# Molecular digitization of a botanical garden: high-depth whole-genome sequencing of 689 vascular plant species from the Ruili Botanical Garden

**DOI:** 10.1093/gigascience/giz007

**Published:** 2019-01-25

**Authors:** Huan Liu, Jinpu Wei, Ting Yang, Weixue Mu, Bo Song, Tuo Yang, Yuan Fu, Xuebing Wang, Guohai Hu, Wangsheng Li, Hongcheng Zhou, Yue Chang, Xiaoli Chen, Hongyun Chen, Le Cheng, Xuefei He, Hechen Cai, Xianchu Cai, Mei Wang, Yang Li, Sunil Kumar Sahu, Jinlong Yang, Yu Wang, Ranchang Mu, Jie Liu, Jianming Zhao, Ziheng Huang, Xun Xu, Xin Liu

**Affiliations:** 1 BGI-Shenzhen, Beishan Industrial Zone, Yantian District, Shenzhen 518083, China; 2 China National GeneBank, Jinsha Road, Dapeng New District, Shenzhen 518120, China; 3 State Key Laboratory of Agricultural Genomics, BGI-Shenzhen, Shenzhen 518083, China; 4 BGI-Yunnan, No. 389 Haiyuan Road, High-tech Development Zone, Kunming, Yunnan 650106, China; 5 Forestry Bureau of Ruili, Yunnan Dehong, Ruili 678600, China

**Keywords:** whole-genome sequencing, vascular plants, phylogeny, voucher specimens, Ruili Botanical Garden

## Abstract

**Background:**

Genome sequencing has been widely used in plant research to construct reference genomes and provide evolutionary insights. However, few plant species have had their whole genome sequenced, thus restraining the utility of these data. We collected 1,093 samples of vascular plant species growing in the Ruili Botanical Garden, located in southwest China. Of these, we sequenced 761 samples and collected voucher specimens stored in the Herbarium of China National GeneBank.

**Results:**

The 761 sequenced samples represented 689 vascular plant species from 137 families belonging to 49 orders. Of these, 257 samples were identified to the species level and 504 to the family level, using specimen and chloroplast sequences. In total, we generated 54 Tb of sequencing data, with an average sequencing depth of 60X per species, as estimated from genome sizes. A reference phylogeny was reconstructed with 78 chloroplast genes for molecular identification and other possible applications.

**Conclusions:**

The large dataset of vascular plant genomes generated in this study, which includes both high-depth whole-genome sequencing data and associated voucher specimens, is valuable for plant genome research and other applications. This project also provides insight into the feasibility and technical requirements for “planetary-scale” projects such as the 10,000 Plant Genomes Project and the Earth BioGenome Project.

## Background

With the advent of next-generation sequencing technologies, enormous efforts have been made to sequence the whole genomes of plant species, thereby providing new insights into plant evolution [1] and new information for improving agriculture yield and stress tolerance [2, 3]. As of November 2018, more than 350 land plant genomes have been sequenced [4], most of which are crops (57.7%), model species and their closely related species (22.3%), and crop wild relatives (17.7%). However, considering the evolutionary history and diversity of the 391,000 known species of plants [5], limited sequence data are currently available. The transcriptome sequences of more than 1,000 plant species have recently been elucidated to better understand plant evolution, thus also providing valuable resources for other plant research [6]. However, considering the high proportion of non-coding regions, studies of plant evolution would benefit from the generation of further whole-genome sequencing data.

As a key part of the Earth BioGenome project [7], a global effort called the 10,000 Plant Genomes Project (10KP) has been initiated to sequence 10,000 plant genomes [8]. The feasibility of large-scale whole-genome sequencing efforts such as this must be determined, as well as establishing technical standards for sampling, sequencing, and data management.

DNA barcoding has emerged as an important molecular tool for ecological studies, particularly for the rapid identification of standard specimens [9]. Although it is well suited for studying historical specimen samples, considering the DNA degradation in those samples [10, 11], a major drawback is that DNA barcoding provides limited genomic information, which is based on only small fragments of the nuclear or chloroplast genome [12]. To overcome this problem, genome skimming, which is whole-genome sequencing using second-generation sequencing technologies, has been proposed [13] to provide more genome sequence information for better species identification [14, 15]. However, previous genome skimming studies have only generated a small amount of sequencing data for individual species. This precludes the re-use of the data to reveal more detailed genome features, including genome sizes (for plants with large genomes), ploidy level, and similar features, or its direct use in further *de novo* genome assembly.

Here, we sequenced the genomes of 761 samples, representing 689 vascular plant species, at high depth (more than 60 Gb per sample, on average). By making these data freely accessible and linking them to voucher details stored in the China National GeneBank (CNGB) herbarium and Ruili Botanical Garden, we provide a valuable genomic resource for evolution and diversity research and applications that may reveal new insights into the evolution of vascular plants.

## Data Description

### Sampling, sequencing, and data summary

**Figure 1:**
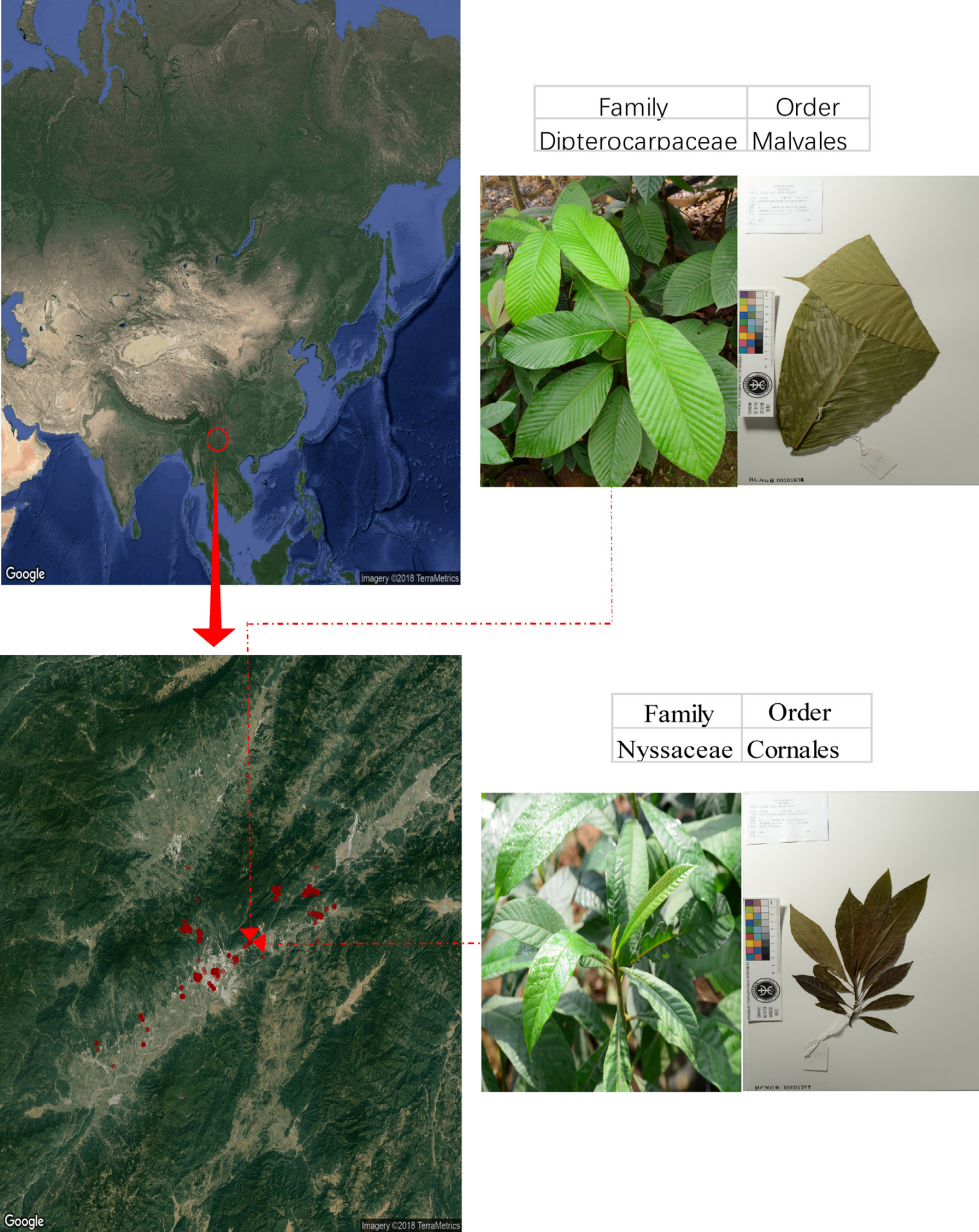
Sampling locations of this project. Sampling was conducted mainly in Ruili Botanical Garden in southwest China, near the China–Myanmar border, shown in red circles.

We sampled almost all of the species growing at the Ruili Botanical Garden, Yunnan, China (97°38′47″ to 98°05′57″ N, 23°52′42″ to 24°09′20″ E, altitude range 738–1,200 m above sea level, as shown in Fig. 1)–1,093 vascular plant samples in total. Young leaves from each sample were used for DNA extraction. Voucher specimens and images were also collected for these samples. All specimens are stored in the CNGB herbarium, and voucher information can be found in Supplementary Table S1 (Additional files). Collected young leaves were shipped to Shenzhen, China, on dry ice, and, using the CTAB method [16], good-quality DNA was extracted from 761 samples.

Whole-genome sequencing libraries were constructed and then sequenced for each of these samples using a BGISEQ-500 desktop sequencer developed by BGI-Shenzhen in 2015, according to the manufacturer's instructions [17]. This machine uses DNA nanoball and combinational probe anchor synthesis technology, developed by Complete Genomics, to generate short reads on a large scale. Sequencing outputs are comparable with the Illumina series [18] and have been successfully utilized to sequence the human genome [19] and metagenomes [20] and for variant identification [21].

Approximately 70 Gb of raw sequencing data (100 bp, paired-end) were generated for each of these samples (Table 1). Raw reads were filtered using SOAPfilter_v2.2 with the following parameters: –y –p –i 180 –M 2 –Q 10. After filtering low-quality reads (reads with more than 10% Ns, ambiguous bases; reads with more than 40% bases having quality lower than 10; reads contaminated by adaptors or polymerase chain reaction duplicates), ∼60 Gb of clean data (high-quality reads >Q35) were obtained for each sample.

**Table 1:** Summary of the sequencing data produced in this study.

Order	Raw base (Gb)	Raw data GC (%)	Raw data Q20	Raw data Q30
Alismatales	66.3873	43.64	95.34	86.48
Apiales	70.0075	35.42	96.40	88.40
Araucariales	74.14	32.87	96.50	88.85
Arecales	68.8318	39.95	95.84	87.20
Asparagales	70.3465	37.97	96.16	87.87
Asterales	67.8382	37.41	95.83	87.20
Brassicales	68.474	37.89	95.99	87.45
Buxales	65.44	42.34	95.38	86.00
Caryophyllales	68.6558	38.04	95.73	87.03
Celastrales	75.8133	38.12	96.56	88.57
Commelinales	65.02	36.80	95.58	86.81
Cornales	76.396	36.49	96.44	88.63
Crossosomatales	60.2	37.17	95.36	86.54
Cucurbitales	65.11	35.73	95.50	86.22
Cupressales	73.54	36.12	96.78	89.01
Cyatheales	75.76	41.32	96.64	88.37
Dioscoreales	78.9	41.47	94.99	85.65
Dipsacales	58.6267	37.58	96.22	87.52
Equisetales	67.3	39.98	94.92	84.77
Ericales	68.1109	38.01	96.46	88.02
Fabales	69.9439	35.50	96.14	87.75
Fagales	68.14	36.81	96.13	87.90
Gentianales	70.1155	36.49	96.36	88.27
Gnetales	71.1267	39.77	96.87	89.24
Lamiales	69.3291	37.47	95.94	87.40
Laurales	71.9425	40.22	96.04	87.83
Liliales	71.4133	41.00	96.73	89.15
Magnoliales	69.0988	38.88	96.12	88.01
Malpighiales	68.1842	35.83	96.40	88.23
Malvales	66.2106	37.19	96.26	88.07
Myrtales	70.7924	38.82	96.23	88.20
Oxalidales	68.3533	34.91	95.61	87.20
Pandanales	72.6733	42.07	96.41	88.31
Pinales	61.04	39.56	93.91	82.96
Piperales	63.2533	40.50	96.23	87.84
Poales	69.6407	44.07	95.56	86.73
Polypodiales	68.588	41.39	96.12	87.69
Proteales	69.0733	39.47	96.49	88.23
Ranunculales	67.5644	38.69	95.68	86.80
Rosales	70.0468	36.72	96.36	88.18
Santalales	69.07	38.11	96.47	88.31
Sapindales	70.5628	36.83	96.14	87.89
Saxifragales	70.84	37.74	96.77	89.36
Schizaeales	62.57	43.84	96.83	89.17
Solanales	72.2389	38.38	96.30	87.93
Vitales	65.235	39.17	95.44	86.71
Zingiberales	67.4956	40.57	95.99	87.51

### Species identification and phylogenetic relationship

Since the specimens collected in this study covered most extant vascular plant lineages, it was not possible to identify each sample to the species level in the short time available. We identified 257 samples to the species level (250 unique species) using specimen morphology, and the remaining 504 samples were identified to the family level using specimen and chloroplast sequences. Thus, we identified 738 samples from 761 sequenced, which belonged to 137 families and 49 orders. Among these families, most species belonged to Fabaceae (71 taxa), Poaceae (45 taxa), and Asteraceae (37 taxa), respectively.

We assembled the chloroplast genomes of each species from clean read data using NOVOPlasty [22], a seed extension-based *de novo* assembler. We used the complete coding sequence of the *rbcL* gene of *Arabidopsis thaliana* (downloaded from the NCBI accession number: U91966) [23] as the seed to conduct the assembly. The NOVOPlasty assembly recovered complete chloroplast genomes of 50 species in a single circular sequence. For the remaining species, the longest contig assembled by NOVOPlasty was BLASTed against the chloroplast database (downloaded from NCBI, including 2,503 non-redundant species) (Supplementary Table S2) and the resulting best-hit sequences (minimum requirement: e-value <10–7 and identity >95%) were used as references for further assembly using MITObim [24]. Complete chloroplast genomes were eventually recovered for all 689 species, ranging from 113,621 to 183,602 bp in size (see Supplemental data in GigaDB) [25]. Assembled chloroplast genomes were annotated using DOGMA [26] and GeneWise [27]. Seventy-two protein-coding genes were found in almost all of these vascular plant families, except the Gnetaceae, Malvaceae, Elaeocarpaceae, and Tectariaceae. For Gnetaceae, we were only able to annotate 52 protein-coding genes in their chloroplast genomes, which is consistent with previous studies [28].

Assembled chloroplast genomes were then compared and a phylogenetic tree constructed using RAxML [29] and IQ_TREE [30]. A total of 78 individual coding genes were identified from 738 samples, most of which were present in 710–738 samples (on average). However, only 18 genes were consistently present among all the plastid genomes; Gnetales and Pinales lost nearly all *ndh* and *rps* genes (Supplementary Table S3).

Each gene was aligned using MAFFT [31], and each alignment was then processed with TrimAL [32] using the gappyout option to remove poorly aligned positions. Gene alignments were then combined, resulting in 59,695 nucleotide positions. Maximum likelihood (ML) species trees were constructed using the RAxML package (version 8.2.4) with the GTRCAT model, 1,000 bootstrap replicates, a random seed number (123 456) selected for parsimony inferences, and 26 fern samples to root the tree. ML analyses were also performed with IQ-TREE using the substitution model GTR+F+R10, which was determined according to the Akaike information criterion and the Bayesian information criterion by IQ-TREE. With the increase in the amount of phylogenetic data, it has become increasingly important to choose different substitution models for variation in rates and patterns of substitution among sites. We partitioned 59,695 nucleotide positions to 78 groups of sites based on gene content, then applied the edge-linked–equal partition model. However, between partitions, a separate model was used with the parameter: -m “GTR+I+G” by IQ-TREE (named IQ-TREE partitions).

**Figure 2:**
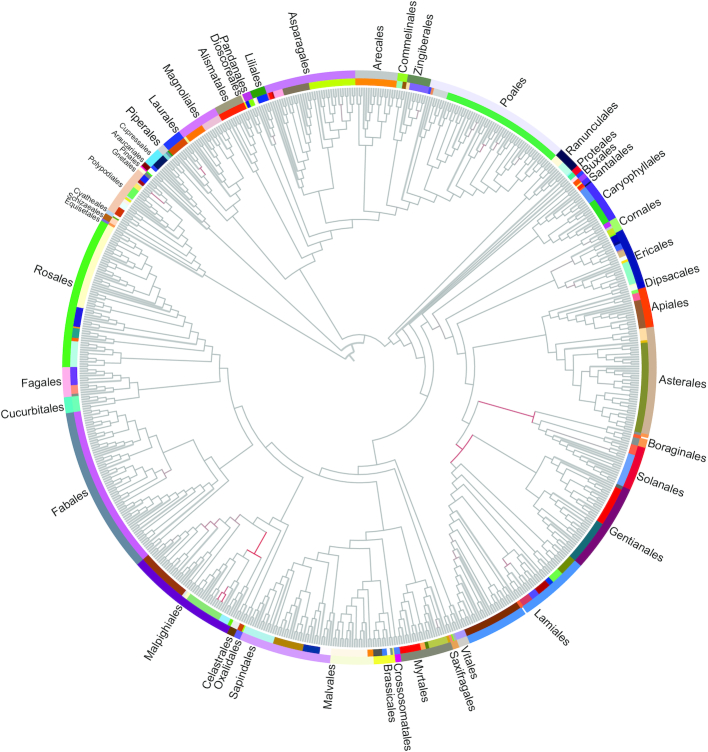
Phylogeny of vascular plants of the Ruili Botanical Garden based on the maximum likelihood analysis tree of 78 chloroplast genes. Colors in the inner circle represent different families, and colors in the outer circle represent different orders.

Both RAxML and IQ_TREE provided consistent phylogenetic reconstructions (Fig. 2 and Supplementary Fig. S1). All nodes in the phylogenetic tree created using the partitioning scheme were the same as those created when no partitioning scheme was used in IQ-TREE. The major lineages can be observed as Fabales, Rosales, Poales, and Malpighiales. Within the Fabids, Celastrales was shown to be a sister group to the Malpighiales, other than Oxalidales in this study (bootstrap support [BS] = 100%). For the Petrosaviidae, the major ordinal relationship was consistent with previous research; like the Liliales, Asparagales, Poales, Arecales, Commelinales, Pandanales, and Zingiberales, the earliest branching lineage was Alismatales [33]. Relationships among Gentianales, Lamiales, and Solanales remained unclear [34, 35].

In this study, the ML tree provided support for the notion that the Gentianales are a sister group to the Lamiales (BS = 83%), which in turn is a sister group to the Solanales and Boraginales (BS = 100%). Fifty-four species of Poales were also analyzed, revealing a close relationship of this group with the Arecales, rather than the Pandanales and Dioscoreales.

### Genome size, repeat content, and heterozygosity

**Figure 3:**
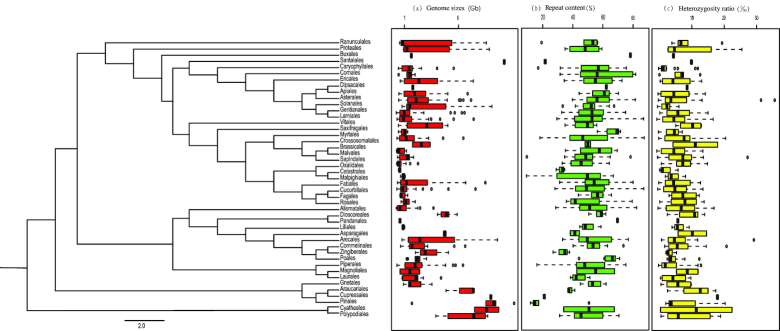
Ordinal phylogeny of vascular plants of the Ruili Botanical Garden based on “drop-tips” from Fig. 2. Based on the species-level phylogenetic tree, we used the drop.tip function in the Ape package (version 5.2) to remove the corresponding internal branches. **(a)** The genome sizes in Gb. **(b)** Repeat content as percentage of total genome (%), and **(c)** the cladogram of the heterozygosity ratio based on 78 chloroplast genes by maximum likelihood phylogeny using only one tip per order.

To ensure the quality and accuracy of the dataset (Table 1), we conducted several analyses to reveal the basic genomic features of the vascular plants sampled. By using GCE [36] and kmergenie [37] software, and clean data for each species, we estimated genome sizes, repeat content, and heterozygosity (Fig. 3 and Supplementary Table S1). The genome sizes of several of the tested species have been previously measured and are publicly available [38] (Supplementary Table S4). We compared these previous estimates to the genome sizes estimated by *k*-mer analysis in this study and found good agreement between them (R2 = 0.63) (Supplementary Fig. S2). Overall, despite there being wide variation in the genome sizes of these plants, most of the families had relatively comparable genome sizes. The most diverse family in terms of genome size was the Cupressaceae, in which genome sizes ranged from 0.18 Gb in *Cunninghamia lanceolata* (Lamb.) Hook. var. *lanceolata* to 19.26 Gb in *Juniperus pingii* var. *wilsonii* (Rehder) Silba. On average, repeat content also varied from 10% to 88% between the species sampled, with several exceptions (Cornaceae, Myrtaceae, and Celastraceae). Myrtaceae (Myrtales) had the most repetitive genomes (∼88% repetitive content), while Celastraceae (Celastrales) had the least repetitive genomes (∼10% repetitive content). There was relatively high heterozygosity in these species, ranging from 0.15% to 36.6% per individual, which probably reflects their nature as wild species.

### Genome assemblies

Despite having constructed only one sequencing library for each species, we were able to assemble preliminary genomes for many of them, reflecting the quality and reuse potential of our data. Based on estimated heterozygosity and repeat content, we initially selected 17 species from 17 families with relatively simple genome content (heterozygosity rate less than 1% and repeat content less than 50%) for genome assembly. We used SOAPdenovo2 [39] (parameters: pregraph-K 35 contig –M 1 scaff) and obtained an average contig N50 of 4.62 kb and an average scaffold N50 of 32.2 kb for these genome assemblies. *Alternanthera sessilis* (L.) R.Br. ex DC was assembled to contig N50 of 15.2 kb and scaffold N50 of 95.5 kb, and *Senna alata* (L.) Roxb. was assembled to a contig N50 of 14 kb and scaffold N50 of 101.1 kb (Supplementary Table S5). We then carried out Benchmarking Universal Single-Copy Orthologs (BUSCO) (version 3.0.1) analysis [40] to find the completeness of these 17 genome assemblies. On average, genome completeness was found to be ∼89.1%; 1,243 BUSCOs were complete and single-copy, and 40 BUSCOs were complete and duplicated (from a total of 1,440 BUSCOs). The average numbers of fragmented and missing BUSCOs were 55 and 101, respectively (Supplementary Table S6).

Our preliminary assemblies were of good quality, providing a useful reference for future efforts to establish complete reference genomes for these plant species. As well as the current genome assembly effort, work continues to finish the preliminary assemblies of the other species; these will be deposited and linked with existing public sequencing data.

### Data access and reuse potential

The data generated here includes images, raw sequencing data, assembled chloroplast genomes, and preliminary nuclear genome assemblies. All data have been organized and linked to a top-level accession in the *GigaScience* GigaDB repository [25], which contains lists of all the species and links to a page for each species. Each species has also been assigned a DOI, linking collection number, a digitized image of the plant taken during sampling, Sequence Read Archive (SRA) accession number for the raw data (filed under SRA project number PRJNA438407 [41]), a data file containing the assembled chloroplast genome sequence in FASTA format (see Supplementary data in GigaDB repository [25]), and a data file containing the preliminary assembled nuclear genome sequence (the latter is only available for some species at present but will be updated as each assembly is completed). Voucher specimens are stored in the herbarium of the CNGB. The data reported in this study are also available in the CNGB Nucleotide Sequence Archive under accession number CNPhis0000538 [42]. With all the metadata indexed and linked via Datacite and GigaDB [25], any future updates made will be traceable records.

The high-depth whole-genome sequencing data, together with images and voucher specimens, can be reused in different ways and will be valuable for future applications. First, future evolutionary analysis may be used to study the evolution of specific genes after assembling them from raw reads, as well as investigating particular features of plant genome evolution, including the evolution of repeats, polyploidization, whole genome duplication, and similar features. Second, the data may be used to improve future genome assemblies of these plant species. For example, the information on repeat content, heterozygosity, and genome sizes provided here may help to tailor new sequencing and genome assembly strategies for these plant genomes. Sequencing data may also be integrated into other genome assemblies. Using the sequencing data obtained from this study would make it easier and more efficient to assemble the remaining sequenced plant genomes. The ∼60 Gb data can be used for genome assembly, in combination with either contig reconstruction of second-generation-based sequence reads or for error correction of third-generation long sequence reads. Finally, this dataset may also be used to develop new methods of species identification based either on sequencing data or plant images and to resolve phylogenetic relationships based on whole-genome sequencing data. At present, we have insufficient information to identify all species, so we are building a living plant database that records the position of species grown in the Ruili Botanical Garden and monitors the status of each species [43].

In combination with information accumulated in the future, deep learning may be applied to this dataset as a training tool to develop plant identification. Indeed, we used data from 175 of the known Ruili species for deep learning, with each sample contributing 1 million reads to build the model. At the first trial stage, 181 species have been successfully identified to the species level using our models. By providing this comprehensive easily and publicly accessible dataset, we believe it would be reused in many ways beyond what has been mentioned here.

## Discussion

Current understanding of the evolution of plants and their diversity in a phylogenomic context is limited because of the lack of genome-scale information across phylogenetically diverse species. In this study, we provide a high-depth whole-genome sequencing dataset comprising 689 vascular plant species with voucher specimens, covering 137 families and 49 orders. These samples were obtained from Ruili Botanical Garden in the Yunnan Province of China, near the border between China and Myanmar, reflecting the rich plant diversity in that region. The data generated here were used to estimate genomic features including genome size, repeat content, and heterozygosity, which will be helpful for future studies aiming to establish reference genomes for these species. The dataset may also be used to assemble chloroplast genomes, as well as some conserved nuclear genes, thus providing useful information for evolution and gene function studies.

In this study, we scaled up a whole-genome sequencing effort to sequence hundreds of plant species. We only constructed a single short insert library (200 bp) for each species and generated ∼70 Gb of whole-genome sequencing data. Although it would be insufficient to assemble high-quality genomes for most species based solely on single library data, the current data have potential uses in analyses such as such as gene finder, plastid, and mitochondrial assembly. We are now using these data, in combination with 10X Genomics, to obtain high-quality genome data for follow-on work including looking at wood development.

This study tested, for the first time, the feasibility of large-scale whole-genome sequencing, which is already underway for the Earth BioGenome Project [8] and the 10KP project [7]. It also provided experience of plant sampling, sample logistics and management, DNA extraction, sequencing library preparation, sequencing and data analysis, and management. Aiming to sequence more than 10,000 plant species, 10KP requires a robust infrastructure for sample and data management, as potentially investigated in this pilot study. We have optimized the DNA extraction protocol and published it via the protocols.io platform [20]. We will soon launch a DNA extraction kit for high-molecular-weight genomic DNA that is suitable for 10X Genomic analysis [16]. We also have just finished writing a guideline on sample submission for 10KP, which includes sample preparation (fresh sample, DNA sample, and RNA sample), sample packing, and shipping. The specific guidelines will be soon available via the 10KP website [44].

## Availability of supporting data

The specimens, leaf samples, and DNA solutions of all collections are stored at the CNGB herbarium. The raw sequencing data described in this article are available in the NCBI SRA repository, under project number PRJNA438407. The data reported in this study are also available in the CNGB Nucleotide Sequence Archive under accession number CNPhis0000538. DNA extraction [16] and BGISEQ-500 whole-genome sequencing library construction protocols can be found via protocols.io [17]. A total of 738 chloroplast genomes and 17 assembled genomes together with raw data supporting the results presented here are available via the *GigaScience* GigaDB repository and will be continuously updated and linked to the GigaDB entries as new assemblies are completed [40].

## Additional files

**Additional file 1: Supplementary Table S1**. List of samples included in this study, with voucher information, current kmer-based estimation of genome sizes, repeat content and heterozygosity. Identified collections were listed with species names, while unidentified collections with only family and order information. Samples with assembled chloroplast genomes (738) are marked with *; 17 samples with assembled unclear genomes are marked with §.

**Supplementary Table S2**. The chloroplast genome list used as references for further assembly by MITObim.

**Supplementary Table S3**. Gene content information for all assembled chloroplast genomes.

**Supplementary Table S4**. Genome information previously measured and publicly available in Plant DNA C-values Database.

**Supplementary Table S5**. Summary of preliminary genome assemblies of 17 species of vascular plant.

**Supplementary Table S6**. Summary of BUSCO analysis for 17 species of vascular plant.

**Additional file 2: Supplementary Fig. S1**. Phylogeny of vascular plants from the Ruili Botanical Garden. Species tree based on the maximum likelihood analysis of 78 chloroplast genes generated by RAxML. Colors of the inner circle and outer circle represent different families and orders. Clade color represents bootstrap values from red to gray (bootstrap range 50–100).

**Supplementary Fig. S2**. A comparison of genome sizes measured by experimental approaches to the k-mer estimated genome sizes in this study.

## Abbreviations

10KP: 10,000 Plant Genome Project; BS: bootstrap support; BUSCO: Benchmarking Universal Single-Copy Orthologs; CNGB: China National GeneBank; ML: maximum likelihood; NCBI: National Center for Biotechnology Information; SRA: Sequence Read Archive.

## Competing interests

The authors declare that they have no competing interests.

## Funding

This work was supported by grants from the Basic Research Program, Shenzhen Municipal Government, China (grants JCYJ20150529150505656 and JCYJ20150831201643396), as well as funding from the Guangdong Provincial Key Laboratory of Genome Read and Write (grant 2017B030301011) and the Construction of China National GeneBank (Yunnan province, 2015DA008, P.R. China).

## Author contributions

X.L. conceived this study. X.L. and H.L. drafted the manuscript. H.L. managed the project. J.P.W., X.B.W., L.C., X.F.H., H.C.C., J.L.Y., Y.W., R.C.M., J.L., and J.M.Z. collected the samples. T.Y. led the identification of voucher specimens. T.Y., W.X.M., B.S., Y.F., Y.C., and H.Y.C. analyzed the data. T.Y., X.L.C., M.W., and Z.H.H. constructed the phylogenetic tree. G.H.H., W.S.L., H.C.Z., H.C.C., and Y.L. extracted DNA and performed genome sequencing. S.K.S. and X.X. revised and edited the manuscript. All the authors read and approved the final manuscript.

## Supplementary Material

GIGA-D-18-00121_Original_Submission.pdf

GIGA-D-18-00121_Revision_1.pdf

GIGA-D-18-00121_Revision_2.pdf

GIGA-D-18-00121_Revision_3.pdf

Response_to_Reviewer_Comments_Original_Submission.pdf

Response_to_Reviewer_Comments_Revision_1.pdf

Response_to_Reviewer_Comments_Revision_2.pdf

Reviewer_1_Report_Original_Submission -- Michael Pirie 9/27/2018 Reviewed

Reviewer_1_Report_Revision_1 -- Michael Pirie 11/26/2018 Reviewed

Reviewer_2_Report_Original_Submission -- Alex Twyford 10/2/2018 Reviewed

Reviewer_2_Report_Revision_1 -- Alex Twyford 12/4/2018 Reviewed

Reviewer_3_Report_Original_Submission -- Monica Carlsen 10/15/2018 Reviewed

Supplemental Files
