# Supplementary material for: Molecular digitization of a botanical garden: high-depth whole-genome sequencing of 689 vascular plant species from the Ruili Botanical Garden
Source: Gigascience. 2019 Jan 25;8(4):giz007. doi: 10.1093/gigascience/giz007 (PMC6441391; doi:10.1093/gigascience/giz007)
Supplement: GIGA-D-18-00121_Original_Submission.pdf [file giz007_giga-d-18-00121_original_submission.pdf]

GigaScience

Digitizing a Botanical Garden: High-depth whole genome sequencing of 689 vascular plants from Ruili Garden

--Manuscript Draft--

**Manuscript Number:**	GIGA-D-18-00121
**Full Title:**	Digitizing a Botanical Garden: High-depth whole genome sequencing of 689 vascular plants from Ruili Garden
**Article Type:**	Data Note
**Funding Information:**	the Shenzhen Municipal Government of china (JCYJ20150529150505656) — Dr. Xin Liu<br>the Shenzhen Municipal Government of China (JCYJ20150831201643396) — Dr. Yue Chang<br>The Construction of China National GeneBank (Yunnan GeneBank) (2015DA008) — Dr. Le Cheng<br>State Key Laboratory of Agricultural Genomics (2011DQ782025) — Dr. Huan Liu<br>Guangdong Provincial Key Laboratory of Genome Read and Write (2017B030301011) — Dr. Wangsheng Li
**Abstract:**	Background<br>Genome sequencing has been widely used in plant research to construct reference genomes and elucidate evolutionary insights. However, only a limited number of plant species have had their whole genome sequenced, and the limited taxon information of these species has further restrained the utility of these data.<br>Findings<br>Here, we comprehensively sampled and sequenced vascular plant species of Ruili Botanical Garden, located in South West China. We sequenced 760 samples out of the total 1,093 collected voucher specimens stored in the Herbarium of China National GeneBank (HCNGB). These 760 samples represented 689 vascular plant species from 134 families belonging to 47 orders. Of these, 254 samples were identified to 232 species by specimen and 506 samples can be identified to families by chloroplast sequences. We generated 54 Tb sequencing data in total, which resulted in an average sequencing depth of 60×for these species, as estimated by the genome size. A reference phylogeny was reconstructed with 78 chloroplast genes for molecular identification and possible applications.<br>Conclusions<br>In this study, we established a large dataset of vascular plants' genomes, with both the high-depth whole genome sequencing data and the voucher specimens, making it valuable dataset for plant genome researches and applications. And providing insight into the feasibility and technical requirements for "planetary scale" projects such as the 10 thousand Plant Genome Project (10KP) and Earth BioGenome Project (EBP).
**Corresponding Author:**	Xin Liu, Ph.D.<br>BGI<br>CHINA
**Corresponding Author Secondary Information:**	
**Corresponding Author's Institution:**	BGI
**Corresponding Author's Secondary Institution:**	
**First Author:**	Huan Liu
**First Author Secondary Information:**	
**Order of Authors:**	Huan Liu

Powered by Editorial Manager® and ProduXion Manager® from Aries Systems Corporation

	jinpu Wei
	Ting Yang
	Weixue Mu
	Bo Song
	Tuo Yang
	Yuan Fu
	Xuebin Wang
	Guohai Hu
	Wangsheng Li
	Hongcheng Zhou
	Yue Chang
	Xiaoli Chen
	Hongyun Chen
	Le Cheng
	Xuefei He
	Hechen Cai
	Xianchu Cai
	Mei Wang
	Yang Li
	Sunil Kumar Sahu, PhD.
	Jinlong Yang
	Yu Wang
	Ranchang Mu
	Jie Liu
	Jianming Zhao
	Ziheng Huang
	Xin Liu, Ph.D.
**Order of Authors Secondary Information:**	
**Additional Information:**	
**Question**	**Response**
Are you submitting this manuscript to a special series or article collection?	No
**Experimental design and statistics**<br><br>Full details of the experimental design and statistical methods used should be given in the Methods section, as detailed in our Minimum Standards Reporting Checklist. Information essential to interpreting the data presented should be made available in the figure legends.	Yes

Powered by Editorial Manager® and ProduXion Manager® from Aries Systems Corporation

Have you included all the information requested in your manuscript?	
**Resources**<br><br>A description of all resources used, including antibodies, cell lines, animals and software tools, with enough information to allow them to be uniquely identified, should be included in the Methods section. Authors are strongly encouraged to cite Research Resource Identifiers (RRIDs) for antibodies, model organisms and tools, where possible.<br><br>Have you included the information requested as detailed in our Minimum Standards Reporting Checklist?	Yes
**Availability of data and materials**<br><br>All datasets and code on which the conclusions of the paper rely must be either included in your submission or deposited in publicly available repositories (where available and ethically appropriate), referencing such data using a unique identifier in the references and in the "Availability of Data and Materials" section of your manuscript.<br><br>Have you have met the above requirement as detailed in our Minimum Standards Reporting Checklist?	Yes

Manuscript
Click here to access/download;Manuscript;Plants_Manuscript_-8.8.docx
Click here to view linked References

Digitizing a Botanical Garden: High-depth whole genome sequencing of 689 vascular plants from Ruili Garden

Authors

Huan Liu[1*], Jinpu Wei[2*], Ting Yang[1*], Weixue Mu[1], Bo Song[1], Tuo Yang[2], Yuan Fu[1], Xuebing Wang[2], Guohai Hu[2], Wangsheng Li[2], Hongcheng Zhou[2], Yue Chang[1], Xiaoli Chen[1], Hongyun Chen[1], Le Cheng[3], Xuefei He[2], Hechen Cai[2], Xianchu Cai[2], Mei Wang[1], Yang Li[2], Sunil Kumar Sahu[1], Jinlong Yang[3], Yu Wang[3], Ranchang Mu[4], Jie Liu[4], Jianming Zhao[4], Ziheng Huang[1], Xin Liu[1#].

Author Affiliations

1. BGI-Shenzhen, Shenzhen 518083, China
2. China National GeneBank, BGI-Shenzhen, Shenzhen 518120, China
3. BGI-Yunnan, BGI-Shenzhen, Kunming, 650106, China
4. Forestry Bureau of Ruili, Ruili, 678600, China

[*] These authors contributed equally to this work.

[#] To whom correspondence should be addressed: Xin Liu (liuxin@genomics.cn)

Abstract

Background

Genome sequencing has been widely used in plant research to construct reference genomes and elucidate evolutionary insights. However, only a limited number of plant species have had their whole genome sequenced, and the limited taxon information of these species has further restrained the utility of these data.

Findings

Here, we comprehensively sampled and sequenced vascular plant species of Ruili Botanical Garden, located in South West China. We sequenced 761 samples out of the total 1,093 collected voucher specimens stored in the Herbarium of China National GeneBank (HCNGB). These 761 samples represented 689 vascular plant species from 134 families belonging to 47 orders. Of these, 254 samples were identified to 232 species by specimen and 506 samples can be identified to families by chloroplast sequences. We generated 54 Tb sequencing data in total, which resulted in an average sequencing depth of 60×for these species, as estimated by the genome size. A reference phylogeny was reconstructed with 78 chloroplast genes for molecular identification and possible applications.

Conclusions

In this study, we established a large dataset of vascular plants' genomes, with

both the high-depth whole genome sequencing data and the voucher specimens, making it valuable dataset for plant genome researches and applications. And providing insight into the feasibility and technical requirements for "planetary scale" projects such as the 10 thousand Plant Genome Project (10KP) and Earth BioGenome Project (EBP).

Keywords: Whole genome sequencing, Vascular plants, Phylogeny, Voucher specimens, Ruili Botanical Garden.

Background

With the advent of sequencing technologies, enormous efforts have been made to sequence whole genome of plant species, thereby providing new insights on the evolution of plants [1] and informations for improving agriculture yield and stress tolerance [2, 3]. As of September 2018, more than 350 land plant's genome are sequenced (https://www.ncbi.nlm.nih.gov/genome), most of which are crops (57.7%), model species along with their related species (22.3%), and wild relatives of crops (17.7%) [4]. However, with approximately 391,000 known species of plants [5], if we consider the evolutionary history and diversity of plants, the currently available sequencing data is very limited. More recently, more than 1,000 plant species have been sequenced at the transcriptome to reveal the evolution of plants, and thus also provide valuable resources for other plant research [6]. However, considering the enormous

gap areas outside of the coding regions, whole genome sequencing data should be generated for further plant evolution studies. Thus, global efforts have been initiated to sequence 10,000 plant genomes (10KP) as a key part of the Earth BioGenome Project (EBP)[7]. For these large-scale whole genome sequencing efforts, we need to prove the feasibility as well as to set up technical routines for sampling, sequencing and data management.

Over the past decade, DNA barcoding has emerged as an important molecular tool for botanical studies, and for the rapid identification of non-routine specimens [8]. Although it is well-suited for studying historical specimen samples, considering the DNA degradation in those samples [9, 10], the major drawback of the technology is that DNA barcoding only provides limited genomic information, which is just based on the small fragments of the nuclear or chloroplast genome [11]. In order to overcome this problem, genome skimming, which is whole genome sequencing by second-generation sequencing technologies, has been proposed [12] to provide more genome sequence information for better species identification [13, 14]. However, previous genome skimming studies have only generated a small amount of sequencing data for the individual species, precluding re-use of the data to reveal more genome features including genome sizes, repeat content, ploidy etc., or its direct usage in the further *de novo* genome assembly. Here, we sequenced vascular plants genomes of 761 samples representing 689 vascular plant species at high depth (more than 60G on an average). Making

all of these data freely accessible and linked to the voucher details in the CNGB herbarium and Ruili botanical garden will provide new insights into the evolution of vascular plants and enable it to be utilized as a valuable genomic resource for evolution and diversity research and applications.

Data Description

Sampling, sequencing and data summary

In order to investigate the diversity of vascular plants in Ruili Botanical Garden and provide genome information for those vascular species for possible conservation, we sampled the vascular species in Ruili Garden and sequenced them using BGISEQ-500 sequencing technology. These samples were collected from Ruili Botanical Garden, Yunnan, China (97°38′47″ to 98°05′57″ N, 23°52′42″ to 24°09′20″ E, altitudes ranging from 738 m to 1,200 m above the sea level, as shown in **Figure 1**). In total, we collected 1,093 vascular plant samples, from which we used the young leaves for DNA extraction. Voucher specimens and images were also collected for these samples accordingly. All the specimens are stored in the Herbarium, China National GeneBank (HCNGB), and the voucher information can be found in **Table S1** (**Additional files**). The collected young leaves were shipped to Shenzhen on dry ice, and then subjected to DNA extraction using the CTAB method[15]. Finally, we succeeded in extracting enough DNA for 761 of those samples.. With the extracted DNA, whole genome sequencing libraries were

constructed for each of these samples according to BGISEQ-500 manufacturer instructions [16], and then pair-end 100 bp sequencing was carried out [17]. Finally, approximately 70 Gb of raw sequencing data (100 bp, paired-end) was generated for each of these samples (**Table 1**). After filtering the low-quality reads (reads with more than 10% Ns, ambiguous bases; reads with more than 40% bases having quality lower than 10; reads contaminated by adaptors or PCR duplications), ~60 Gb clean data were obtained for each of these samples.

Species identification and phylogenetic relationship

The identification of specimens is a time-consuming progress and requires expertise and experience. Here, the collections covering the majority of vascular plant lineages were difficult to identify the species in a short time. We identified 254 samples to 232 species using the specimen morphology and the other 506 samples can be identified to families by chloroplast sequences. Thus, in total, we identified 689 species from those 761 sequenced samples, which belonged to 134 families and 47 orders. Among these families, the majority of the species belonged to Fabaceae (71 species), Poaceae (45 species) and Asteraceae (38 species) respectively.

We assembled the chloroplast genomes using the clean data of each species by using NOVOPlasty, which is a seed-extension-based *de novo* assembler. We used the complete cds *rbcL* gene sequence of *Arabidopsis thaliana* (downloaded from NCBI, accession number: U91966) as the seed to conduct

the assembly. After assembly by NOVOPlasty, chloroplast genomes of 50 species were finally assembled into the single circular sequence. For the remaining species, the longest contig assembled by NOVOPlasty were BLAST against the chloroplast database (download from NCBI, including 2,503 non-redundant species) and the resulted best-hit sequences (minimum requirement: e-value < 10^{-7} and identity > 95%) were used as references for further assembly using MITObim, in this way, we finally recovered complete chloroplast genomes for all 689 species. The assembled chloroplast genome ranged from 113,621 to 183,602 bp in size (**Table S2**). We then annotated the assembled chloroplast genomes using DOGMA [18] and GeneWise [19], and we found 72 protein-coding genes in almost all of these vascular plant families except Gnetaceae, Malvaceae, Elaeocarpaceae, and Tectariaceae. For Gnetaceae, we were only able to annotate 52 protein-coding genes in their chloroplast genomes which is consistent with previous studies [20]. We then compared these assembled chloroplast genomes and constructed the phylogenetic tree using the gene trees and translated to the species tree. We collected 78 coding genes were identified from 738 samples, and majority of them were present in 710 to 738 (on average). However, only 18 genes were commonly found in all the studied samples. Each gene was aligned using Mafft [21] and every alignment was then processed with TrimAL [22] using the gappyout option to remove poorly aligned positions. Maximum likelihood (ML) gene trees were constructed by RAxML package (v8.2.4) with GTRCAT model,

1,000 bootstrap replicates, 5 random number seed for the parsimony inferences was chose and 26 fern samples were used as root. Astral[23] was used to estimate a species tree by combining 78 gene trees (**Figure 3)**. The major lineages can be observed within Fabales, Rosales, Poales, Lamiales and Malpighiales. In Fabids, Celastrales was the sister group to Malpighiales other than Oxalidales in this study. For Petrosaviidae, the major ordinal relationship was consistent with the previous research, such as Liliales, Asparagales, Poales, Arecales, Commelinales, Pandanales, Zingiberales in the same clade and the most early-branched lineage is Alismatales [24]. We also included 54 species of Poales in the phylogenetic tree which revealed its close relationship with Arecales rather than Pandanales and Dioscoreales.

Genome size, repeat content, and heterozygosity

In order to ensure the quality and effectiveness of the dataset (**Table 1**), we conducted several analyses to reveal the basic genomic features of these vascular plants. By using GCE [25] and kmergenie [26] software for the clean data of each species, we estimated the genome sizes, repeat content and heterozygosity (**Figure 2** and **Table S1**). For several of these tested species, the genome sizes have been previously measured by experimental approaches and are publicly available (http://data.kew.org/cvalues/) (**Table S3**). We compared the estimations from these datasets to the genome sizes estimated by k-mer analysis in this study, to find good consistency between them (R^2=0.63) **(Figure S1).** We found that despite overall high variations in

the genome sizes of these plants, most of the families had relatively comparable genome sizes. However, the most diversified family was found to be Cupressaceae, in which the genome sizes ranging from 0.18 Gb in *Cunninghamia lanceolata* (Lamb.) Hook. var. *lanceolata* to 19.26 Gb in *Juniperus pingii* var*. wilsonii* (Rehder) Silba. In addition, the repeat content varied from 10% to 88% in vascular species, with several exceptions in Cornaceae and Myrtaceae. For instance, Myrtaceae in Myrtales was found to have the most repetitive genomes (~88%), while Celastraceae in Celastrales was found to have the least repetitive genomes (~10%). We also found relatively high heterozygosity in these species ranging from 0.15‰ to 36.6‰, which probably reflected their nature as wild species.

Genome assemblies

Despite the limitation of having only one sequencing library constructed for each species, we were able to conduct preliminary genome assembly for many of these species, which reflected the data quality and reuse potential. Here, based on the estimated heterozygosity and repeat content, we initially selected 17 species from 17 families with relatively simple genome content (heterozygosity rate less than 1% and repeat content less than 50%) for the genome assembly. We used SOAPdenovo2 [27] (parameters: pregraph-K 35 contig -M 1 scaff). We obtained an average contig N50 of 4.62 kb, and an average scaffold N50 of 32.2 kb for these genome assemblies. Two species of *Alternanthera sessilis* (L.) R.Br. ex DC. and *Senna alata* (L.) Roxb., were

assembled to contig N50 of 15.2 kb, scaffold N50 of 95.5 kb and contig N50 of 14 kb and the scaffold N50 of 101.1 kb respectively (**Table S4**). We then carried out Benchmarking Universal Single-Copy Orthologs (BUSCO)(version 3.0.1) analysis [28] to find the completeness to be ~89.1% for all the 17 genome assemblies (in average 1243 BUSCOs to be complete and single-copy and in average 40 BUSCOs to be complete and duplicated, of the total 1440 BUSCOs). The average numbers of fragmented and missing BUSCOs were 55 and 101, respectively (**Table S5**). Our preliminary assemblies were of good quality, providing a useful reference for future efforts to establish reference genomes for all these plant species. In addition to the current attempt of genome assembly, continuing efforts are being carried out to finish the preliminary assemblies of the other species and these are being deposited with and linked with existing the already public sequencing data.

Data access and reuse potential

The data generated here included the images, raw sequencing data, assembled chloroplast and preliminary assemblies. All the data have been organized and linked to a top level accession in the *GigaScience* GigaDB repository (DOI:XXXX), containing the lists of all the species and the links to the page of each species. Linked to this, each species has a DOI assigned to them containing the information of collection number, image of the plant during sampling, SRA accession number of the raw data, the data file containing the assembled chloroplast sequence (the current chloroplast sequences can also

be found in **Table S2**), the data file containing the preliminary assembled genome sequence (available only for some species and will continue to be updated when each assembly is completed). The specimens are stored in the Herbarium, China National GeneBank (HCNGB), and digitized images for every sheets are also being made available in GigaDB alongside the sequencing data. All the raw data are stored in the NCBI SRA repository under the project number PRJNA43840. In addition to the description in SRA, the SRA accession number of raw data is also included in the GigaDB entries, thus the raw data of specified species can be traced from GigaDB. Datacite and GigaDB metadata is all linked, and any future updates made on the GigaDB dataset provides traceable records.

The high-depth whole genome sequencing data together with the image and voucher specimen can be reused in different ways and will be valuable for future applications. First of all, in addition to the phylogenetic analysis carried out here based on the assembled chloroplast, future evolutionary analysis can be carried out to depict evolution of specific genes (after assembly of these genes), figure out plant genome evolution features including evolution of repeats, polyploidization, whole genome duplication, etc. (with optimization or developing of suitable methods). Secondly, the data can be used for future genome assembly of these plant species. Other than utilizing the information on repeat content, heterozygosity and genome size estimation provided here to tailor sequencing and genome assembly strategies of plant genomes, the

sequencing data itself can also be integrated in further genome assemblies. By directly using the obtained sequencing data from this study, it would be more easy and efficient to assemble remaining sequenced plant genomes. The ~70 Gb data can either be used for contig construction of the second generation sequencing based genome assembly, or error correction for the third generation long reads based genome assembly. Last but not least, this dataset can also be used for developing new methods for species identification either based on sequencing data or based on images of plants, resolving the phylogenetic relationships based on whole genome sequencing data, etc. For example, deep learning can be applied to develop plant identification using this dataset as a good training set. Providing this comprehensive dataset which can be easily accessed by the researchers and also public, we think it would be reused in many ways beyond what had been mentioned here.

Discussion

The evolution of vasculature was a major event in plant history. In this study, we provide a dataset of high-depth whole genome sequencing of 689 vascular plant species with voucher specimens, covering 134 families and 47 orders. These samples were obtained from Ruili Botanical Garden in Yunnan Province of China, near the border between China and Myanmar, reflecting the rich plant diversity in that region. The high-depth whole genome sequencing data generated here have been used to estimate genomic features including genome size, repeat content, and heterozygosity, which can provide guidance to the future studies aiming at establishing reference genomes for these species. The high-depth whole genome data can be used in assembling the chloroplast genomes, as well as some conserved nuclear genes, thus providing useful information for evolution and gene function studies.

In this study, we scaled up the plant whole genome sequencing effort to sequence hundreds of plant species. We only constructed a single short insert size library for each of the species and generated ~60 Gb whole genome sequencing data. It would be insufficient to assemble good draft genomes for majority of the species just based on single library data, because previous efforts to assemble reference genomes based on second generation sequencing data would require multiple short insert size libraries and also mate pair (large insert size) libraries. However, in addition to future reuse of the current data, our study, for the first time, tested the feasibility of large-scale

whole genome sequencing, which is already underway for the Earth BioGenome Project (EBP) [7] and 10 thousand Plant Genome Projects (10KP) [29]. With particular relevance for the 10KP, this study provided experiences for plant sampling, sample logistics, sample management, DNA extraction, sequencing library preparation, sequencing, data analysis and data management. Aiming at sequencing more than 10,000 plant species, 10KP would require and establish a robust infrastructure for sample and data management, as potentially investigated at a pilot scale by this study.

Availability of Supporting Data

The specimens, leaf samples and DNA solutions of all collections are maintained at the China National GeneBank (CNGB) Herbarium. The raw sequencing data described in this article are available in the NCBI SRA repository, under the project number PRJNA43840. DNA Extraction [27] and BGISEQ-500 WGS library construction protocols can be found in protocols.io [28].

738 chloroplast genomes and 17 assembly genomes together with data supporting the results of this article is available via the *GigaScience* GigaDB repository, and will be continued to be updated and linked to the GigaDB entries as the assemblies are completed.

Additional files

Additional file 1

Table S1. List of the samples with voucher information, current kmer based estimation of genome sizes, repeat content and heterozygosity. Identified

collections were listed with species names, while unidentified ones with only family and order information. 738 samples with assembled chloroplast genome were marked with *, whereas 17 samples with assembled genome were marked with §.

Table S2. All the assembled chloroplast genomes and their lengths.

Table S3. The genome information has been previously measured and are publicly available on the database.

Table S4. Summary of preliminary genome assemblies of 17 species from 17 families.

Table S5. Summary of BUSCO analysis for 17 species.

Additional file 2

Figure S1. The genome sizes in datasets compared to k-mer estimate in this study.

Abbreviations

10 KP: 10 thousand Plant genome Project

bp: base pair

BUSCO: Benchmarking Universal Single-Copy Orthologs.

EBP: Earth BioGenome Project.

Gb: Gigabase pair

HCNGB: Herbarium, China National GeneBank.

ML: Maximum likelihood.

WGS: Whole Genome Sequencing.

Competing interests

All authors declare that they have no competing interests.

Funding

This work was supported by the grants of Basic Research Program, the Shenzhen Municipal Government, China (No.JCYJ20150529150505656) and (No.JCYJ20150831201643396), as well as the funding to State Key Laboratory of Agricultural Genomics (No.2011DQ782025), and Guangdong Provincial Key Laboratory of Genome Read and Write(No.2017B030301011), The Construction of China National GeneBank (Yunnan GeneBank) (Yunnan province, 2015DA008, P.R. China)

Author contributions

XL conceived this study. XL and HL drafted the manuscript. HL managed the project. JPW, XBW, LC, XFH, HCC, JLY, YW, RCM, JL, JMZ collected the samples. TY leaded identification of voucher specimens. TY, WXM, BS, YF, YC, HYC analyzed the data. TY, XLC, MW, ZHH constructed the phylogenetic

tree. GHH, WSL, HCZ, HCC, YL extracted DNA and performed genome sequencing. SKS revised and edited the manuscript. All the authors have read and approved the final manuscript.

Acknowledgments

The authors would like to express their sincere thanks to the local people and Government of Yunnan province for their help in sample collection. They would also like to thank the taxonomic experts in PE for identification. Finally, we are thankful to the production team of China National GeneBank, Shenzhen, China.

References

1. Pennisi E. Plant biology. Green genomes. Science. 2011;332 6036:1372-5. doi:10.1126/science.332.6036.1372.
2. Bolger ME, Weisshaar B, Scholz U, Stein N, Usadel B and Mayer KF. Plant genome sequencing - applications for crop improvement. Curr Opin Biotechnol. 2014;26:31-7. doi:10.1016/j.copbio.2013.08.019.
3. Desta ZA and Ortiz R. Genomic selection: genome-wide prediction in plant improvement. Trends Plant Sci. 2014;19 9:592-601. doi:10.1016/j.tplants.2014.05.006.
4. Leitch I, Coker T and Leitch A. Plant genomes - progress and prospects. 2017.
5. Kew RBG. The state of the world’s plants report–2016. Royal Botanic Gardens, Kew. 2016.
6. Matasci N, Hung L-H, Yan Z, Carpenter EJ, Wickett NJ, Mirarab S, et al. Data access for the 1,000 Plants (1KP) project. Gigascience. 2014;3 1:17.
7. Lewin HA, Robinson GE, Kress WJ, Baker WJ, Coddington J, Crandall KA, et al. Earth BioGenome Project: Sequencing life for the future of life.

Proc Natl Acad Sci U S A. 2018;115 17:4325-33.

8. de Vere N, Rich TC, Trinder SA and Long C. DNA barcoding for plants. Methods Mol Biol. 2015;1245:101-18. doi:10.1007/978-1-4939-1966-6_8.

9. Staats M, Erkens RH, van de Vossenberg B, Wieringa JJ, Kraaijeveld K, Stielow B, et al. Genomic treasure troves: complete genome sequencing of herbarium and insect museum specimens. PLoS One. 2013;8 7:e69189. doi:10.1371/journal.pone.0069189.

10. Osmundson TW, Robert VA, Schoch CL, Baker LJ, Smith A, Robich G, et al. Filling gaps in biodiversity knowledge for macrofungi: contributions and assessment of an herbarium collection DNA barcode sequencing project. PLoS One. 2013;8 4:e62419. doi:10.1371/journal.pone.0062419.

11. Li X, Yang Y, Henry RJ, Rossetto M, Wang Y and Chen S. Plant DNA barcoding: from gene to genome. Biol Rev Camb Philos Soc. 2015;90 1:157-66. doi:10.1111/brv.12104.

12. Straub SC, Parks M, Weitemier K, Fishbein M, Cronn RC and Liston A. Navigating the tip of the genomic iceberg: Next-generation sequencing for plant systematics. Am J Bot. 2012;99 2:349-64. doi:10.3732/ajb.1100335.

13. Male PJ, Bardon L, Besnard G, Coissac E, Delsuc F, Engel J, et al. Genome skimming by shotgun sequencing helps resolve the phylogeny of a pantropical tree family. Mol Ecol Resour. 2014;14 5:966-75. doi:10.1111/1755-0998.12246.

14. Besnard G, Christin PA, Male PJ, Coissac E, Ralimanana H and Vorontsova MS. Phylogenomics and taxonomy of Lecomtelleae (Poaceae), an isolated panicoid lineage from Madagascar. Ann Bot. 2013;112 6:1057-66. doi:10.1093/aob/mct174.

15. Wu C and Yang T. DNA Extraction for plant samples by CTAB. Gigascience. 2018; doi:10.17504/protocols.io.pzqdp5w.

16. Gao S, Mu F, Yang Z, Liu X, Jiang H, Liao S, et al. BGISEQ-500 WGS library construction. 2018; doi:10.17504/protocols.io.ps5dng6.

17. Gao S, Mu F, Yang Z, Liu X, Jiang H, Liao S, et al. BGISEQ-500

Sequencing. doi:10.17504/protocols.io.pq7dmzn.
18. Wyman SK, Jansen RK and Boore JL. Automatic annotation of organellar genomes with DOGMA. Bioinformatics. 2004;20 17:3252-5. doi:10.1093/bioinformatics/bth352.
19. Birney E, Clamp M and Durbin R. GeneWise and Genomewise. Genome Res. 2004;14 5:988-95. doi:10.1101/gr.1865504.
20. Hsu CY, Wu CS, Surveswaran S and Chaw SM. The complete plastome sequence of Gnetum ula (Gnetales: Gnetaceae). Mitochondrial DNA A DNA Mapp Seq Anal. 2016;27 5:3721-2. doi:10.3109/19401736.2015.1079874.
21. Katoh K, Misawa K, Kuma K and Miyata T. MAFFT: a novel method for rapid multiple sequence alignment based on fast Fourier transform. Nucleic Acids Res. 2002;30 14:3059-66.
22. Capella-Gutiérrez S, Silla-Martínez JM and Gabaldón T. trimAl: a tool for automated alignment trimming in large-scale phylogenetic analyses. Bioinformatics. 2009;25 15:1972-3.
23. Mirarab S, Reaz R, Bayzid MS, Zimmermann T, Swenson MS and Warnow T. ASTRAL: genome-scale coalescent-based species tree estimation. Bioinformatics. 2014;30 17:i541-i8.
24. Chase MW. Monocot relationships: an overview. Am J Bot. 2004;91 10:1645-55. doi:10.3732/ajb.91.10.1645.
25. Liu B SY, Yuan J, Hu X, Zhang H, Li N, Li Z, Chen Y, Mu D, Fan W. Estimation of genomic characteristics by analyzing k-mer frequency in de novo genome projects. arXiv preprint. 2013; doi:arXiv:1308.2012.
26. Chikhi R and Medvedev P. Informed and automated k-mer size selection for genome assembly. Bioinformatics. 2014;30 1:31-7. doi:10.1093/bioinformatics/btt310.
27. Luo R, Liu B, Xie Y, Li Z, Huang W, Yuan J, et al. SOAPdenovo2: an empirically improved memory-efficient short-read de novo assembler. Gigascience. 2012;1 1:18.
28. Simão FA, Waterhouse RM, Ioannidis P, Kriventseva EV and Zdobnov EM. BUSCO: assessing genome assembly and annotation completeness with single-copy orthologs. Bioinformatics. 2015;31

19:3210-2.

29. Cheng S, Melkonian M, Smith SA, Brockington S, Archibald JM, Delaux P-M, et al. 10KP: A Phylodiverse Genome Sequencing Plan. GigaScience. 2018.

Figure legends

Figure 1. Sampling localities of this project. Sampling was conducted mainly in Ruili Botanical Garden in Southwest China, near the China-Myanmar border, and shown in red rectangles.

Figure 2. The genome sizes, repeat content and heterozygosity statistics. (a) genome sizes (b) repeat content (c) heterozygosity ration for samples in this study. Ordinal cladogram was generated from the 78 chloroplast gene phylogeny.

Figure 3. Phylogeny of vascular plants of Ruili Botanical Garden. The tree shows the coalescent tree based on 78 chloroplast genes. Here the colors represent different orders.

Tables

Table 1 Summary of the sequencing data in this study.

Table

Click here to access/download;Table;Table 1.xlsx

Order	Raw base(Gb)	Raw data G	Raw data C	Raw data Q30
Alismatales	66.3873	43.64	95.34	86.48
Apiales	70.0075	35.42	96.40	88.40
Araucariales	74.14	32.87	96.50	88.85
Arecales	68.8318	39.95	95.84	87.20
Asparagales	70.3465	37.97	96.16	87.87
Asterales	67.8382	37.41	95.83	87.20
Brassicales	68.474	37.89	95.99	87.45
Buxales	65.44	42.34	95.38	86.00
Caryophyllales	68.6558	38.04	95.73	87.03
Celastrales	75.8133	38.12	96.56	88.57
Commelinales	65.02	36.80	95.58	86.81
Cornales	76.396	36.49	96.44	88.63
Crossosomatales	60.2	37.17	95.36	86.54
Cucurbitales	65.11	35.73	95.50	86.22
Cupressales	73.54	36.12	96.78	89.01
Cyatheales	75.76	41.32	96.64	88.37
Dioscoreales	78.9	41.47	94.99	85.65
Dipsacales	58.6267	37.58	96.22	87.52
Equisetales	67.3	39.98	94.92	84.77
Ericales	68.1109	38.01	96.46	88.02
Fabales	69.9439	35.50	96.14	87.75
Fagales	68.14	36.81	96.13	87.90
Gentianales	70.1155	36.49	96.36	88.27
Gnetales	71.1267	39.77	96.87	89.24
Lamiales	69.3291	37.47	95.94	87.40
Laurales	71.9425	40.22	96.04	87.83
Liliales	71.4133	41.00	96.73	89.15
Magnoliales	69.0988	38.88	96.12	88.01
Malpighiales	68.1842	35.83	96.40	88.23
Malvales	66.2106	37.19	96.26	88.07
Myrtales	70.7924	38.82	96.23	88.20
Oxalidales	68.3533	34.91	95.61	87.20
Pandanales	72.6733	42.07	96.41	88.31
Pinales	61.04	39.56	93.91	82.96
Piperales	63.2533	40.50	96.23	87.84
Poales	69.6407	44.07	95.56	86.73
Polypodiales	68.588	41.39	96.12	87.69
Proteales	69.0733	39.47	96.49	88.23
Ranunculales	67.5644	38.69	95.68	86.80
Rosales	70.0468	36.72	96.36	88.18
Santalales	69.07	38.11	96.47	88.31
Sapindales	70.5628	36.83	96.14	87.89
Saxifragales	70.84	37.74	96.77	89.36
Schizaeales	62.57	43.84	96.83	89.17
Solanales	72.2389	38.38	96.30	87.93
Vitales	65.235	39.17	95.44	86.71
Zingiberales	67.4956	40.57	95.99	87.51

Click here to access/download;Table;Table S5.xlsx

Voucher	Complete B	Complete a	Complete a	Fragmente	Missing BU	Total BUSCO groups searched
RL0207	1376	1352	24	25	39	1440
RL0211	1259	1181	78	58	123	1440
RL0004	1277	1257	20	73	90	1440
RL0521	1367	1350	17	35	38	1440
RL0526	1247	1231	16	91	102	1440
RL0573	1351	1248	103	18	71	1440
RL0712	1279	1260	19	49	112	1440
RL0779	1246	1229	17	81	113	1440
RL0760	1264	1210	54	55	121	1440
RL0090	1309	1256	53	46	85	1440
RL0125	1253	1217	36	70	117	1440
RL0202	1332	1309	23	46	62	1440
RL0229	1287	1271	16	56	97	1440
RL0235	1251	1195	56	60	129	1440
RL0248	1305	1236	69	46	89	1440
RL0276	1229	1209	20	66	145	1440
RL0425	1171	1139	32	82	187	1440

 Click here to access/download;Table;Table S1.xlsx

Voucher	Species
RL0001	
RL0002	
RL0003	
RL0004	
RL0006	Cordyline fruticosa (L.) A.Chev.
RL0008	
RL0010	
RL0012	
RL0015	Elaeagnus conferta Roxb.
RL0017	
RL0020	
RL0021	Toona ciliata M.Roem.
RL0022	
RL0023	
RL0024	
RL0026	Dalbergia sissoo DC.
RL0027	
RL0030	
RL0031	Murraya paniculata (L.) Jack
RL0036	
RL0037	Bauhinia purpurea L.
RL0041	
RL0043	
RL0044	
RL0045	
RL0046	
RL0048	
RL0053	
RL0055	
RL0057	
RL0059	Morus macroura Miq.
RL0060	Tephrosia kerrii J.R.Drumm. & Craib
RL0061	
RL0062	
RL0065	Cassia fistula L.
RL0066	
RL0069	
RL0070	
RL0071	
RL0072	
RL0074	
RL0075	Artocarpus lacucha Buch.-Ham.
RL0076	
RL0077	
RL0078	Crotalaria pallida Aiton
RL0079	
RL0080	
RL0081	
RL0085	

RL0088	
RL0089	
RL0091	
RL0092	
RL0095	
RL0097	
RL0098	
RL0099	
RL0100	
RL0103	
RL0104	
RL0106	
RL0107	
RL0108	
RL0109	Clerodendrum × speciosum Dombrain
RL0110	
RL0111	
RL0112	
RL0113	
RL0114	
RL0115	
RL0116	Ageratina adenophora (Spreng.) R.M.King & H.Rob.
RL0117	
RL0118	
RL0119	Morus australis Poir.
RL0120	
RL0121	
RL0122	
RL0123	
RL0124	
RL0125	
RL0126	
RL0127	
RL0128	
RL0130	Buddleja davidii Franch.
RL0131	
RL0133	
RL0134	
RL0135	
RL0136	
RL0137	
RL0138	Millettia velutina Dunn
RL0140	
RL0141	
RL0142	
RL0143	
RL0145	
RL0146	Tibouchina semidecandra (Mart. & Schrank ex DC.) Cogn.
RL0147	
RL0148	

RL0149		
RL0150		
RL0151	Cyclosorus jinghongensis Ching	
RL0152		
RL0153		
RL0154		
RL0155		
RL0156	Blumea martiniana Vaniot	
RL0157		
RL0158	Acacia auriculiformis Benth.	
RL0159		
RL0160	Solanum chrysotrichum Schltdl.	
RL0161		
RL0164		
RL0165		
RL0166		
RL0168		
RL0169		
RL0170		
RL0171		
RL0172		
RL0175		#N/A
RL0176	Alternanthera brasiliana (L.) Kuntze	
RL0177	Bougainvillea glabra Choisy	
RL0178	Litchi chinensis Sonn.	
RL0179		
RL0181		
RL0182	Trevesia palmata (Roxb. ex Lindl.) Vis.	
RL0183		
RL0184		
RL0186	Macadamia integrifolia Maiden & Betche	
RL0187		
RL0188		
RL0189		
RL0190	Crassocephalum crepidioides (Benth.) S.Moore	
RL0191	Bidens pilosa L.	
RL0192		
RL0193	Sonchus wightianus DC.	
RL0194		
RL0195	Blumea oxyodonta DC.	
RL0196		
RL0197		
RL0198	Dichanthium caricosum (L.) A.Camus	
RL0199	Laggera crispata (Vahl) Hepper & J.R.I.Wood	
RL0200	Neyraudia reynaudiana (Kunth) Keng ex Hitchc.	
RL0201	Urena procumbens L.	
RL0202	Ricinus communis L.	
RL0203		
RL0204		
RL0205		

RL0206	Chenopodium ficifolium Sm.
RL0207	
RL0208	
RL0210	Ageratum houstonianum Mill.
RL0211	Alternanthera sessilis (L.) R.Br. ex DC.
RL0212	
RL0213	Scoparia dulcis L.
RL0214	Amaranthus spinosus L.
RL0216	Vicia hirsuta (L.) Gray
RL0217	Youngia japonica (L.) DC.
RL0218	
RL0220	Alternanthera philoxeroides (Mart.) Griseb.
RL0221	Ranunculus cantoniensis DC.
RL0222	
RL0224	
RL0225	Eclipta prostrata (L.) L.
RL0226	Sonchus oleraceus (L.) L.
RL0227	
RL0228	
RL0229	
RL0230	Chloris barbata Sw.
RL0232	
RL0233	
RL0234	Verbena officinalis L.
RL0235	Aster subulatus (Michx.) Hort. ex Michx.
RL0236	Hyparrhenia rufa (Nees) Stapf
RL0237	
RL0238	
RL0240	
RL0241	
RL0242	Leonotis nepetifolia (L.) R.Br.
RL0243	Phragmites australis (Cav.) Trin. ex Steud.
RL0244	Eragrostis atrovirens (Desf.) Trin. ex Steud.
RL0245	
RL0246	Pteris vittata L.
RL0247	Pseudognaphalium affine (D.Don) Anderb.
RL0248	
RL0249	Cyanthillium cinereum (L.) H.Rob.
RL0251	Cyclosorus parasiticus (L.) Farw.
RL0252	Diplazium esculentum (Retz.) Sw.
RL0255	
RL0256	Cleome rutidosperma DC.
RL0258	
RL0259	
RL0261	
RL0262	Eleusine indica (L.) Gaertn.
RL0263	
RL0264	Lolium perenne L.
RL0265	Melilotus albus Medik.
RL0266	

RL0267	Cyphomandra betacea (Cav.) Sendtn.
RL0268	Coffea canephora Pierre ex A.Froehner
RL0269	Mytilaria laosensis Lecomte
RL0270	
RL0271	
RL0272	
RL0273	
RL0274	Catharanthus roseus (L.) G.Don
RL0275	
RL0276	Moringa oleifera Lam.
RL0277	
RL0278	
RL0279	
RL0280	
RL0281	
RL0282	
RL0283	
RL0284	
RL0285	
RL0286	
RL0288	
RL0289	
RL0290	
RL0294	
RL0295	
RL0296	
RL0297	
RL0298	
RL0299	
RL0301	
RL0302	
RL0304	Bletilla striata (Thunb.) Rchb.f.
RL0306	
RL0308	
RL0309	
RL0310	Gomphocarpus fruticosus (L.) W.T.Aiton
RL0312	
RL0313	
RL0314	
RL0317	
RL0321	
RL0322	
RL0323	
RL0325	Angiopteris caudatiformis Hieron.
RL0328	
RL0329	
RL0330	
RL0332	
RL0333	
RL0334	

RL0335	Tarlmounia elliptica (DC.) H.Rob., S.C.Keeley, Skvarla & R.Chan
RL0336	
RL0337	
RL0339	
RL0340	
RL0341	
RL0342	Solanum chrysotrichum Schltdl.
RL0343	Manihot esculenta Crantz
RL0345	Solanum erianthum D. Don
RL0346	Solanum spirale Roxb.
RL0348	
RL0351	Cajanus cajan (L.) Millsp.
RL0353	
RL0354	
RL0359	Dendrobium heterocarpum Wall. ex Lindl.
RL0360	
RL0361	Dichrocephala integrifolia (L.f.) Kuntze
RL0362	
RL0364	Dendrobium lituiflorum Lindl.
RL0366	Pseudognaphalium affine (D.Don) Anderb.
RL0367	
RL0368	Juncus bufonius L.
RL0371	
RL0372	
RL0373	Lablab purpureus (L.) Sweet
RL0374	Lablab purpureus (L.) Sweet
RL0375	
RL0377	
RL0379	Blumea balsamifera (L.) DC.
RL0380	Tecoma stans (L.) Juss. ex Kunth
RL0381	
RL0382	
RL0383	
RL0384	
RL0385	Alocasia cucullata (Lour.) G.Don
RL0387	
RL0388	
RL0389	Platycladus orientalis (L.) Franco
RL0390	Laggera crispata (Vahl) Hepper & J.R.I.Wood
RL0393	
RL0394	
RL0395	Amaranthus viridis L.
RL0396	
RL0399	
RL0400	
RL0401	Dendrobium sp.
RL0402	
RL0404	Cyathocline purpurea (Buch.-Ham. ex D.Don) Kuntze
RL0408	Lycopersicon esculentum Mill. cv. Yingtaofanqie
RL0410	Citrus limon (L.) Osbeck

RL0411	
RL0412	
RL0413	
RL0414	
RL0416	Pteris vittata L.
RL0417	
RL0418	
RL0421	Themeda triandra Forssk.
RL0423	Senna occidentalis (L.) Link
RL0424	Morus alba L.
RL0425	Kyllinga brevifolia Rottb.
RL0426	
RL0427	
RL0428	
RL0429	
RL0430	
RL0433	
RL0434	Buxus harlandii Hance
RL0435	
RL0438	Solanum americanum Mill.
RL0440	
RL0441	
RL0443	Vanda cf. brunnea
RL0445	
RL0446	
RL0447	
RL0448	Clausena lansium (Lour.) Skeels
RL0449	Vernonia sp.
RL0450	
RL0453	
RL0455	Vernonia amygdalina Delile
RL0457	Grevillea robusta A.Cunn. ex R.Br.
RL0459	Paspalum scrobiculatum L.
RL0462	
RL0463	Nicotiana tabacum L.
RL0465	Anisodus acutangulus C.Y.Wu & C.Chen
RL0468	Bryophyllum delagoense (Eckl. & Zeyh.) Druce
RL0469	
RL0471	
RL0472	Cyperus involucratus Rottb.
RL0473	Engelhardia spicata Leschen ex Blume
RL0474	Cunninghamia lanceolata (Lamb.) Hook. var. lanceolata
RL0475	
RL0476	
RL0477	
RL0478	
RL0479	
RL0480	
RL0482	
RL0483	

RL0484	Pteris biaurita L.
RL0485	
RL0486	Cyclosorus gymnopteridifrons (Hayata) C.M. Kuo
RL0487	Embelia sessiliflora Kurz
RL0488	Pogostemon nigrescens Dunn
RL0489	Rubus ellipticus Sm.
RL0490	Blechnum orientale L.
RL0492	Odontosoria chinensis (L.) J. Sm.
RL0493	
RL0494	Amischotolype hispida (A.Rich.) D.Y.Hong
RL0495	Desmodium styracifolium (Osbeck) Merr.
RL0496	Lygodium salicifolium C.Presl is
RL0497	
RL0498	
RL0499	
RL0500	Flemingia strobilifera (L.) W.T.Aiton
RL0501	Lindsaea ensifolia Sw.
RL0502	
RL0503	
RL0504	
RL0505	Microsorum punctatum (L.) Copel.
RL0506	
RL0508	
RL0511	
RL0512	
RL0513	Argyreia pierreana Bois
RL0514	Codariocalyx motorius (Houtt.) H.Ohashi
RL0515	
RL0517	Lobelia seguinii H.Lév. & Vaniot
RL0518	Prunus salicina Lindl.
RL0519	
RL0520	Pyrus pseudopashia T.T.Yu
RL0521	Prunus persica (L.) Batsch
RL0522	Euphorbia pulcherrima Willd. ex Klotzsch
RL0523	
RL0524	
RL0525	Plantago asiatica L.
RL0526	Carica papaya L.
RL0527	Maesa indica (Roxb.) A. DC.
RL0528	
RL0530	
RL0531	
RL0532	
RL0533	
RL0534	Carex baccans Nees
RL0535	Pseudodrynaria coronans (Wall. ex Mett.) Ching
RL0536	Embelia sessiliflora Kurz
RL0537	
RL0538	Pteridium revolutum (Blume) Nakai
RL0539	Coix lacryma-jobi L.

RL0541	
RL0542	Vicia sativa L.
RL0543	Helixanthera pulchra (DC.) Danser
RL0544	
RL0545	
RL0546	Phyllodium pulchellum (L.) Desv.
RL0547	
RL0548	
RL0549	
RL0550	Elatostema macintyrei Dunn
RL0551	Odontosoria chinensis (L.) J. Sm.
RL0552	Imperata cylindrica (L.) Raeusch.
RL0553	
RL0554	
RL0555	Acmella calva (DC.) R.K.Jansen
RL0556	
RL0557	
RL0559	
RL0560	Wahlenbergia marginata (Thunb.) A.DC.
RL0562	
RL0563	
RL0564	
RL0566	Polycarpon prostratum (Forssk.) Asch. & Schweinf.
RL0567	Gnaphalium polycaulon Pers.
RL0568	Fimbristylis aestivalis Vahl
RL0570	
RL0571	
RL0572	
RL0573	Senna alata (L.) Roxb.
RL0574	
RL0575	
RL0576	Meliosma simplicifolia (Roxb.) Walp.
RL0577	
RL0578	Callerya pachyloba (Drake) H. Sun
RL0579	
RL0580	
RL0581	
RL0582	Molineria capitulata (Lour.) Herb.
RL0583	Stemona tuberosa Lour.
RL0585	
RL0586	
RL0587	
RL0588	
RL0589	
RL0590	
RL0591	Fissistigma minuticalyx (McGregor & W.W.Sm.) Chatterjee
RL0592	Atalantia dasycarpa C.C.Huang
RL0593	
RL0594	Lepisanthes senegalensis(Poir.) Leenh.
RL0595	Sterculia monosperma Vent.

RL0596	
RL0597	
RL0598	
RL0599	Atalantia dasycarpa C.C.Huang
RL0600	Flemingia kweichowensis Y.T.Wei & S.K.Lee
RL0601	
RL0602	
RL0603	
RL0604	
RL0605	
RL0606	
RL0607	
RL0609	
RL0610	Aristolochia chlamydophylla C.Y.Wu
RL0612	
RL0613	
RL0614	
RL0616	
RL0618	Gmelina arborea Roxb.
RL0620	Pseudocaryopteris paniculata (C.B.Clarke) P.D.Cantino
RL0621	Cyathea gigantea (Wall. ex Hook.) Holttum
RL0622	
RL0627	
RL0628	
RL0630	Cibotium barometz (L.) J.Sm.
RL0631	
RL0632	
RL0633	
RL0634	
RL0635	
RL0636	Ipomoea cairica var. gracillima (Collett & Hemsl.) C.Y. Wu
RL0637	Zeuxine strateumatica (L.) Schltr.
RL0638	Bauhinia variegata L.
RL0639	
RL0640	Codonopsis javanica (Blume) Hook.f. & Thomson
RL0641	
RL0642	Medicago sativa L.
RL0643	
RL0644	Acilepis saligna (DC.) H.Rob.
RL0645	
RL0646	
RL0647	
RL0648	
RL0649	
RL0650	
RL0651	Diplospora mollissima Hutch.
RL0652	
RL0653	
RL0655	
RL0657	

RL0659	
RL0660	Disporum calcaratum D.Don
RL0661	
RL0662	
RL0664	Pteris aspericaulis Wall. ex J. Agardh
RL0665	Tetrastigma tsaianum C.Y. Wu
RL0667	Lygodium japonicum (Thunb.) Sw.
RL0668	Mucuna pruriens (L.) DC.
RL0669	Pennisetum qianningense S.L.Zhong
RL0671	Cymbidium ensifolium (L.) Sw.
RL0672	
RL0674	
RL0676	
RL0677	
RL0680	
RL0682	
RL0683	
RL0684	Clematis wissmanniana Hand.-Mazz.
RL0686	Toona sureni (Blume) Merr.
RL0687	
RL0688	
RL0689	
RL0691	Hiptage candicans Hook. f.
RL0692	
RL0693	
RL0696	
RL0697	
RL0698	
RL0699	
RL0700	Syzygium forrestii Merr. & L.M.Perry
RL0701	
RL0702	
RL0704	Engelhardia spicata Leschen ex Blume
RL0705	Engelhardia spicata Leschen ex Blume
RL0708	Alocasia odora (Lindl.) K.Koch
RL0709	
RL0710	
RL0711	
RL0712	
RL0713	Catunaregam spinosa (Thunb.) Tirveng.
RL0714	
RL0715	Schima argentea E.Pritz. ex Diels
RL0717	Bridelia tomentosa Blume
RL0718	
RL0720	Mecardonia procumbens (Mill.) Small
RL0721	
RL0722	Melilotus albus Medik.
RL0723	
RL0725	
RL0726	

RL0727	
RL0729	Broussonetia papyrifera (L.) L'Hér. ex Vent.
RL0730	Colebrookea oppositifolia Sm.
RL0731	
RL0732	Cipadessa baccifera (Roth) Miq.
RL0734	Tetrastigma pachyphyllum (Hemsl.) Chun
RL0735	
RL0737	
RL0738	Pseudocaryopteris paniculata (C.B.Clarke) P.D.Cantino
RL0739	Brassaiopsis hainla (Buch.-Ham.) Seem.
RL0740	
RL0741	
RL0742	Colona floribunda (Kurz) Craib
RL0743	
RL0744	
RL0745	Boehmeria glomerulifera Miq.
RL0746	
RL0748	Congea tomentosa Roxb.
RL0749	
RL0750	Saccharum longisetosum (Andersson) V.Naray. ex Bor
RL0752	
RL0753	
RL0754	
RL0755	Buddleja officinalis Maxim.
RL0756	Pogostemon brevicorollus Y.Z.Sun
RL0757	Toddalia asiatica (L.) Lam.
RL0758	Lobelia nummularia Lam.
RL0759	
RL0760	Scutellaria shweliensis W.W.Sm.
RL0761	Itea yunnanensis Franch.
RL0762	Millettia pulchra Kurz
RL0763	
RL0765	
RL0766	Brassaiopsis fatsioides Harms
RL0767	
RL0768	
RL0769	Lonicera hildebrandiana Collett & Hemsl.
RL0770	
RL0771	
RL0772	Archidendron balansae (Oliv.) I.C.Nielsen
RL0773	
RL0774	Firmiana colorata (Roxb.) R.Br.
RL0775	
RL0776	
RL0777	Beaumontia khasiana Hook.f.
RL0778	
RL0779	
RL0781	Erigeron sumatrensis Retz.
RL0783	
RL0784	Pistacia weinmanniifolia J. Poiss. ex Franch.

RL0786	
RL0787	Gnetum montanum Markgr.
RL0788	Micromelum integerrimum (Buch.-Ham. ex DC.) Wight & Arn. ex M. Roem.
RL0790	
RL0794	
RL0801	Crotalaria ferruginea Benth.
RL0806	
RL0807	
RL0808	
RL0812	
RL0813	
RL0814	
RL0822	
RL0824	
RL0826	Morus wittiorum Hand.-Mazz.
RL0827	
RL0828	
RL0831	
RL0832	Centratherum punctatum subsp. fruticosum K.Kirkman
RL0834	Achillea alpina L.
RL0838	
RL0840	
RL0842	
RL0846	Alangium barbatum subsp. faberi (Oliv.) Bloemb.
RL0850	
RL0855	
RL0856	
RL0858	
RL0859	Microsorum punctatum (L.) Copel.
RL0864	
RL0866	
RL0867	Agrimonia pilosa Ledeb.
RL0868	Oenothera rosea L'Hér. ex Aiton
RL0869	Zanthoxylum bungeanum Maxim.
RL0871	
RL0872	Hamelia patens Jacq.
RL0873	
RL0874	
RL0875	Dalbergia assamica Benth.
RL0876	Vernicia montana Lour.
RL0877	Maclura tricuspidata Carrière
RL0879	
RL0881	
RL0882	
RL0883	
RL0890	Coreopsis lanceolata L.
RL0891	
RL0892	
RL0896	Capparis urophylla F.Chun
RL0900	Girardinia diversifolia (Link) Friis

RL0901	
RL0902	
RL0905	
RL0907	
RL0908	
RL0909	
RL0911	
RL0912	
RL0914	
RL0915	
RL0917	
RL0918	
RL0919	
RL0921	Macrosolen cochinchinensis(Lour.) Tiegh.
RL0925	
RL0927	
RL0928	Asplenium nidus L.
RL0929	
RL0931	
RL0933	Stachyphrynium placentarium (Lour.) Clausager & Borchs.
RL0941	Cleidion brevipetiolatum Pax & K.Hoffm.
RL0942	
RL0948	
RL0949	Alphonsea monogyna Merr. & Chun
RL0959	Peliosanthes macrostegia Hance
RL0961	
RL0963	
RL0964	
RL0969	Pyrrosia costata Tagawa & K. Iwats.
RL0976	Pteridrys cnemidaria (Christ) C. Chr. & Ching
RL0977	
RL0978	Dysoxylum excelsum Blume
RL0981	Crocosmia × crocosmiiflora (Lemoine) N.E.Br.
RL0984	
RL0985	
RL0986	
RL0989	
RL0990	
RL0992	
RL0994	
RL0995	
RL0996	
RL0998	
RL1002	
RL1004	Asplenium antrophyoides Christ
RL1005	
RL1007	Pterospermum truncatolobatum Gagnep.
RL1009	
RL1011	
RL1013	

RL1014	
RL1016	
RL1018	
RL1019	
RL1020	
RL1024	
RL1025	
RL1026	
RL1027	
RL1028	
RL1029	
RL1031	
RL1033	
RL1034	
RL1035	
RL1036	
RL1038	Lobelia zeylanica L.
RL1039	
RL1041	
RL1042	
RL1043	
RL1044	
RL1045	
RL1047	
RL1049	
RL1050	
RL1051	
RL1052	Styrax dasyanthus Perkins
RL1053	
RL1054	
RL1058	
RL1059	
RL1061	
RL1062	
RL1063	
RL1064	
RL1065	Kalimeris indica (L.) Sch.Bip.
RL1067	Dendrobium thyrsiflorum B.S.Williams
RL1069	Erythrina crista-galli L.
RL1070	
RL1071	
RL1072	
RL1073	
RL1074	
RL1075	
RL1076	
RL1077	
RL1078	
RL1079	
RL1080	

RL1081	
RL1082	
RL1083	
RL1084	
RL1085	
RL1086	
RL1087	
RL1089	
RL1090	
RL1091	
RL1093	Flemingia stricta Roxb.

Family	Order	Genome as	Chloroplas	kmer based estimatio	repeat content (%
Moraceae	Rosales		*	688188000	56.46
Rutaceae	Sapindales		*	826374000	63.47
Solanaceae	Solanales		*	5534290000	48.06
Rutaceae	Sapindales	§	*	281368000	40.38
Asparagaceae	Asparagales		*	1207040000	68.67
Lauraceae	Laurales		*	2624380000	60.78
Araliaceae	Apiales		*	2031010000	61.64
Moraceae	Rosales		*	636790000	49.26
Elaeagnaceae	Rosales		*	779908000	56.22
Moraceae	Rosales		*	677153000	49.27
Phyllanthaceae	Malpighiales		*		
Meliaceae	Sapindales		*	868761000	41.93
Boraginaceae	Gentianales		*	1152150000	75.56
Araceae	Alismatales		*		
Apocynaceae	Gentianales		*	1907390000	44.4
Fabaceae	Fabales		*	756564000	44.07
Passifloraceae	Malpighiales		*	2763740000	81
Passifloraceae	Malpighiales		*	1983300000	73.46
Rutaceae	Sapindales		*	307088000	35.18
Moraceae	Rosales		*	618795000	50.66
Fabaceae	Fabales		*	296409000	35.8
Euphorbiaceae	Malpighiales		*	779899000	55.16
Fabaceae	Fabales		*	1364210000	33.94
Boraginaceae	Gentianales		*	6312650000	62.53
Arecaceae	Arecales		*		
Euphorbiaceae	Malpighiales		*	1120040000	59.28
Magnoliaceae	Magnoliales		*	4382180000	42.56
Oleaceae	Lamiales		*	4296950000	52.92
Moraceae	Rosales		*	328309000	30.47
Fabaceae	Fabales		*	2706870000	68.22
Moraceae	Rosales		*	1355270000	72.55
Fabaceae	Fabales		*		
Passifloraceae	Malpighiales		*	3843890000	32.26
Gentianaceae	Gentianales		*	5273210000	73.29
Fabaceae	Fabales		*	467098000	39.58
Araceae	Alismatales		*	4864390000	56.72
Meliaceae	Sapindales		*	406501000	42.6
Poaceae	Poales		*	2577040000	35.69
Poaceae	Poales		*		
Euphorbiaceae	Malpighiales		*		
Poaceae	Poales		*	1988650000	55.36
Moraceae	Rosales		*	775268000	35.27
Phyllanthaceae	Malpighiales		*	1989250000	74.94
Poaceae	Poales		*	2712150000	64.18
Fabaceae	Fabales		*	1332120000	69.71
Moraceae	Rosales		*	377682000	48.2
Zingiberaceae	Zingiberales		*	2195670000	69.61
Amaryllidaceae	Asparagales		*		
Magnoliaceae	Magnoliales		*	2230980000	42.75

Apocynaceae	Gentianales		*	527753000	41.33
Myrtaceae	Myrtales		*	602693000	44.81
Moraceae	Rosales		*	784806000	52.67
Moraceae	Rosales		*	323003000	38.26
Euphorbiaceae	Malpighiales		*	611067000	51.04
Magnoliaceae	Magnoliales		*	2752160000	39.4
Lamiaceae	Lamiales		*	309006000	31.6
Calophyllaceae	Malpighiales		*	533683000	36.3
Salicaceae	Malpighiales		*	341982000	39.34
Sapindaceae	Sapindales		*	481363000	46.58
Nephrolepidaceae	Polypodiales		*	8703890000	38.27
Myrtaceae	Myrtales		*	494887000	59.33
Phyllanthaceae	Malpighiales		*	511669000	56.92
Capparaceae	Brassicales		*	1026520000	66.67
Lamiaceae	Lamiales		*	1237710000	60.67
Moraceae	Rosales		*	319363000	44.22
Salicaceae	Malpighiales		*	2394970000	62.69
Bignoniaceae	Lamiales		*	683260000	53.76
Combretaceae	Myrtales		*	2769320000	40.66
Fabaceae	Fabales		*	861003000	48.05
Zingiberaceae	Zingiberales		*	1213110000	45.09
Asteraceae	Asterales		*	2561470000	82.54
Zingiberaceae	Zingiberales		*	2905990000	69.29
Solanaceae	Solanales		*		
Moraceae	Rosales		*	307620000	49.79
Oxalidaceae	Oxalidales		*	426424000	29.94
Rutaceae	Sapindales		*	392239000	53.28
Sapindaceae	Sapindales		*	450393000	37.65
Rosaceae	Rosales		*	631734000	49.91
Zingiberaceae	Zingiberales		*	2170170000	61.62
Meliaceae	Sapindales	§	*	320656000	38.67
Fabaceae	Fabales		*	930693000	40.97
Fabaceae	Fabales		*	647437000	40.6
Fabaceae	Fabales		*		
Scrophulariaceae	Lamiales		*	909389000	51.88
Oleaceae	Lamiales		*	728054000	40.42
Cupressaceae	Cupressales		*	9455000000	12.83
Asparagaceae	Asparagales		*	771523000	55.41
Meliaceae	Sapindales		*	500179000	33.95
Arecaceae	Arecales		*	641346000	41.21
Moraceae	Rosales		*	854744000	51.21
Fabaceae	Fabales		*	972991000	45.62
Fabaceae	Fabales		*	670839000	39.06
Fabaceae	Fabales		*	1348490000	63.67
Euphorbiaceae	Malpighiales		*	4500590000	35.54
Lauraceae	Laurales		*	1485160000	63.08
Arecaceae	Arecales		*	1360620000	51.23
Melastomataceae	Myrtales		*	806539000	69.18
Podocarpaceae	Araucariales		*		
Fabaceae	Fabales		*	858323000	46.17

Poaceae	Poales		*	2077210000	41.75
Pandanaceae	Pandanales		*	764880000	45.63
Thelypteridaceae	Polypodiales		*	6645030000	45.68
Lauraceae	Laurales		*	1479540000	55.99
Bignoniaceae	Lamiales		*	433512000	39.44
Arecaceae	Arecales		*	806685000	49.84
Euphorbiaceae	Malpighiales		*	3897770000	51.35
Asteraceae	Asterales		*	2528550000	44.08
Moraceae	Rosales		*	1287460000	81.05
Fabaceae	Fabales		*	663003000	36.84
Lamiaceae	Lamiales		*	548544000	47.06
Solanaceae	Solanales		*	1281770000	53.26
Araceae	Alismatales		*	5094600000	60.76
Annonaceae	Magnoliales		*	837469000	46.89
Pandanaceae	Pandanales		*	1028160000	59.72
Euphorbiaceae	Malpighiales		*	1016740000	52.23
Araliaceae	Apiales		*	1642840000	52.14
Fabaceae	Fabales		*	592580000	39.21
Poaceae	Poales		*	2717950000	62.21
Myrtaceae	Myrtales		*	317648000	38.23
Araliaceae	Apiales		*	1999070000	64.61
Rhamnaceae	Rosales		*	1577570000	45.45
Amaranthaceae	Caryophyllales		*		
Nyctaginaceae	Caryophyllales		*	5557760000	32.7
Sapindaceae	Sapindales		*	1043140000	51.14
Arecaceae	Arecales		*	6622670000	47.89
Asparagaceae	Asparagales		*	7458450000	57.05
Araliaceae	Apiales		*	5018030000	62.89
Rubiaceae	Gentianales		*	1057130000	55.71
Fabaceae	Fabales		*	1203880000	55.44
Proteaceae	Proteales		*	1465790000	60.39
Asteraceae	Asterales		*	2080100000	65.58
Rosaceae	Rosales		*	636040000	53.77
Asteraceae	Asterales		*	6450490000	45.74
Asteraceae	Asterales		*	6061980000	50.36
Asteraceae	Asterales		*	2782760000	65.9
Rosaceae	Rosales		*	1370890000	63.06
Asteraceae	Asterales		*	1412060000	70
Cucurbitaceae	Cucurbitales		*	573416000	61.05
Asteraceae	Asterales		*	6202320000	56.09
Verbenaceae	Lamiales		*	5552450000	79.58
Moraceae	Rosales		*	488796000	54.67
Poaceae	Poales		*	2318080000	69.99
Asteraceae	Asterales		*	2244950000	53.47
Poaceae	Poales		*	1024600000	44.25
Malvaceae	Malvales		*	1516520000	53.19
Euphorbiaceae	Malpighiales	§	*	392193000	47.76
Araliaceae	Apiales		*	1704380000	46.99
Polygonaceae	Caryophyllales		*	1233090000	66.6
Polygonaceae	Caryophyllales		*	726313000	55.04

Amaranthaceae	Caryophyllales		*	785464000	66.02
Brassicaceae	Brassicales	§	*	252856000	36.06
Polygonaceae	Caryophyllales		*	1246040000	58.64
Asteraceae	Asterales		*	1231400000	62.38
Amaranthaceae	Caryophyllales	§	*	446974000	17.97
Menispermaceae	Ranunculales		*	721740000	57.08
Plantaginaceae	Lamiales		*	680850000	58.13
Amaranthaceae	Caryophyllales		*	432061000	38.85
Fabaceae	Fabales		*	3499590000	52.02
Asteraceae	Asterales		*	1295470000	61.15
Myrtaceae	Myrtales		*	1815300000	65.37
Amaranthaceae	Caryophyllales		*	1634010000	57.93
Ranunculaceae	Ranunculales		*	5385460000	52.26
Apiaceae	Apiales		*	642303000	67.47
Fabaceae	Fabales		*	819973000	55.74
Asteraceae	Asterales		*	1149250000	58.48
Asteraceae	Asterales		*	1508580000	66.04
Solanaceae	Solanales		*	1617990000	44.63
Malvaceae	Malvales		*	1363240000	49.81
Sapindaceae	Sapindales	§	*	319313000	48.63
Poaceae	Poales		*	1051720000	50.48
Acanthaceae	Lamiales		*	1464930000	60.59
Myrtaceae	Myrtales		*	286687000	25.13
Verbenaceae	Lamiales		*	1265280000	74.91
Asteraceae	Asterales	§	*	480773000	40.7
Poaceae	Poales		*	2222300000	73.27
Araliaceae	Apiales		*	1990100000	67.64
Asteraceae	Asterales		*	1440180000	62.92
Lamiaceae	Lamiales		*	1264720000	59.8
Boraginaceae	Gentianales		*	613039000	51.39
Lamiaceae	Lamiales		*	572470000	51.05
Poaceae	Poales		*	948707000	32.43
Poaceae	Poales		*		
Rubiaceae	Gentianales		*	1179730000	66.27
Pteridaceae	Polypodiales				
Asteraceae	Asterales		*	1515640000	70.47
Apiaceae	Apiales	§	*	477211000	45.2
Asteraceae	Asterales		*	1306630000	67.28
Thelypteridaceae	Polypodiales		*	8261430000	46.11
Athyriaceae	Polypodiales		*		
Lythraceae	Myrtales		*	333115000	19.08
Cleomaceae	Brassicales		*	454672000	65.72
Equisetaceae	Equisetales		*		
Asteraceae	Asterales		*		
Commelinaceae	Commelinales		*	5069250000	35.64
Poaceae	Poales		*	596750000	52.23
Fabaceae	Fabales		*	1218100000	61.95
Poaceae	Poales		*		
Fabaceae	Fabales				
Lauraceae	Laurales		*	901309000	46.06

Solanaceae	Solanales		*	9104870000	34.28
Rubiaceae	Gentianales		*	1257980000	74.86
Hamamelidaceae	Saxifragales		*	1300410000	70.25
Magnoliaceae	Magnoliales		*	2059600000	39.78
Fabaceae	Fabales		*	499591000	29.12
Magnoliaceae	Magnoliales		*	2052520000	40.96
Annonaceae	Magnoliales		*	828908000	43.61
Apocynaceae	Gentianales		*	653032000	46.61
Meliaceae	Sapindales		*	295656000	35.46
Moringaceae	Brassicales	§	*	293006000	48.8
Ebenaceae	Ericales		*	1153130000	54.86
Fabaceae	Fabales		*	688393000	47.18
Dipterocarpaceae	Malvales		*	351064000	45.26
Fabaceae	Fabales		*	926329000	39.67
Fabaceae	Fabales		*	7576470000	88.2
Lauraceae	Laurales		*	3550800000	51.32
Fabaceae	Fabales		*	1436210000	44.6
Podocarpaceae	Araucariales		*	8996120000	22.01
Myrtaceae	Myrtales		*	4579120000	88.43
Combretaceae	Myrtales		*		
Dipterocarpaceae	Malvales		*	403869000	42.38
Nyssaceae	Cornales		*	1227880000	46.02
Magnoliaceae	Magnoliales		*	2073310000	46.67
Sapindaceae	Sapindales		*	348041000	41.07
Fabaceae	Fabales		*	677229000	62.1
Fabaceae	Fabales		*	689428000	44.63
Fagaceae	Fagales		*	1725110000	67.09
Theaceae	Ericales		*	1359600000	51.6
Bignoniaceae	Lamiales		*	558759000	47.75
Annonaceae	Magnoliales		*		
Fabaceae	Fabales		*	441649000	36.28
Orchidaceae	Asparagales		*	2550320000	39.86
Apocynaceae	Gentianales		*	506929000	47.28
Apocynaceae	Gentianales		*	1190460000	38.7
Apocynaceae	Gentianales		*	1083300000	41.6
Apocynaceae	Gentianales		*	303407000	59.62
Malvaceae	Malvales		*	1245390000	53.48
Celastraceae	Celastrales		*	941073000	10.06
Solanaceae	Solanales		*	3003390000	65.23
Celastraceae	Celastrales		*	623145000	67.93
Fabaceae	Fabales		*	1521930000	66.18
Staphyleaceae	Crossosomatales		*	1743370000	52.75
Lamiaceae	Lamiales		*	548961000	47.85
Marattiaceae	Marattiales				
Apocynaceae	Gentianales		*	567093000	54.28
Bignoniaceae	Lamiales		*	757442000	40.08
Fabaceae	Fabales		*		
Magnoliaceae	Magnoliales		*	2474230000	50.44
Magnoliaceae	Magnoliales		*		
Combretaceae	Myrtales		*	1578740000	56.08

Asteraceae	Asterales	*	3660930000	59.71
Berberidaceae	Ranunculales			
Apocynaceae	Gentianales	*	733368000	63.29
Nyssaceae	Cornales	*	457452000	32.54
Phyllanthaceae	Malpighiales	*	2786300000	51.61
Araliaceae	Apiales	*	2103470000	61.02
Solanaceae	Solanales			
Euphorbiaceae	Malpighiales	*	1270480000	71.31
Solanaceae	Solanales	*	1491290000	48.86
Solanaceae	Solanales	*	1207570000	54.33
Combretaceae	Myrtales	*	1971230000	39.81
Fabaceae	Fabales	*	817271000	54.29
Lamiaceae	Lamiales	*	1659140000	74.09
Sapotaceae	Ericales	*	606133000	35.27
Orchidaceae	Asparagales	*	1257660000	52.98
Orchidaceae	Asparagales	*	2302100000	50.67
Asteraceae	Asterales	*	1465090000	59.58
Brassicaceae	Brassicales	*		
Orchidaceae	Asparagales	*	1145260000	34.26
Asteraceae	Asterales	*	1045640000	63.92
Plantaginaceae	Lamiales	*	1197160000	53.76
Juncaceae	Poales	*	1045320000	63.12
Polygonaceae	Caryophyllales	*	2566240000	76.59
Polygonaceae	Caryophyllales	*	1560290000	66.89
Fabaceae	Fabales	*	524682000	45.23
Fabaceae	Fabales	*	692240000	71.28
Typhaceae	Poales	*	926045000	62.43
Rutaceae	Sapindales	*	366033000	46.5
Asteraceae	Asterales	*	2370880000	50.41
Bignoniaceae	Lamiales	*	346304000	41.49
Moraceae	Rosales	*	394999000	38.76
Sapotaceae	Ericales	*	984635000	47.37
Amaryllidaceae	Asparagales	*		
Annonaceae	Magnoliales	*	831890000	40.16
Araceae	Alismatales	*	5884430000	60.08
Asparagaceae	Asparagales	*	9609970000	68.91
Apocynaceae	Gentianales	*	720843000	51.21
Cupressaceae	Cupressales	*	8541070000	14.65
Asteraceae	Asterales	*	4926290000	41.47
Malvaceae	Malvales	*	1625160000	61.02
Acanthaceae	Lamiales			
Amaranthaceae	Caryophyllales	*	425602000	41.96
Polygonaceae	Caryophyllales	*	2087670000	42.06
Orchidaceae	Asparagales	*	1364960000	67.94
Orchidaceae	Asparagales	*	1342190000	57.99
Orchidaceae	Asparagales	*	1178680000	44.46
Orchidaceae	Asparagales	*	1244910000	41.04
Asteraceae	Asterales	*	2186780000	64.86
Solanaceae	Solanales	*	872758000	42.6
Rutaceae	Sapindales	*	406461000	47.26

Orchidaceae	Asparagales		*	7122220000	40.77
Acanthaceae	Lamiales		*	7402710000	54.86
Orchidaceae	Asparagales		*	5953520000	50.39
Asparagaceae	Asparagales		*	5621580000	70.42
Pteridaceae	Polypodiales		*	3899840000	71.67
Menispermaceae	Ranunculales		*	975669000	57.57
Vitaceae	Vitales		*	427760000	35.79
Poaceae	Poales		*	4651410000	17.15
Fabaceae	Fabales		*	597726000	59.27
Moraceae	Rosales		*	1154760000	72.67
Cyperaceae	Poales	§	*	298040000	35.88
Plantaginaceae	Lamiales		*	1831550000	75.72
Taxaceae	Cupressales		*		
Polygonaceae	Caryophyllales		*	1421220000	62.9
Polygonaceae	Caryophyllales		*	1128750000	63.31
Lauraceae	Laurales		*	1269620000	58.4
Asteraceae	Asterales		*	1673040000	43.58
Buxaceae	Buxales		*	1631410000	79.31
Asparagaceae	Asparagales		*		
Solanaceae	Solanales		*	1217260000	68.41
Fabaceae	Fabales		*	633743000	52.99
Primulaceae	Ericales		*	2866920000	73.74
Orchidaceae	Asparagales		*	4244320000	44.45
Moraceae	Rosales		*	364767000	44.63
Ericaceae	Ericales		*	4062840000	65.34
Actinidiaceae	Ericales				
Rutaceae	Sapindales		*	294134000	45.4
Asteraceae	Asterales		*	2782540000	41.21
Cupressaceae	Cupressales		*	11151500000	18.3
Araceae	Alismatales		*		
Asteraceae	Asterales		*	4481460000	53.43
Proteaceae	Proteales		*	901217000	56.25
Poaceae	Poales		*	2425110000	50
Acanthaceae	Lamiales		*	2528750000	71.19
Solanaceae	Solanales		*	4124680000	62.89
Solanaceae	Solanales		*	1292880000	49.34
Crassulaceae	Saxifragales		*	1019180000	72.73
Polygonaceae	Caryophyllales		*	759190000	54.06
Cupressaceae	Cupressales		*	9257800000	15.72
Cyperaceae	Poales		*	304131000	27
Juglandaceae	Fagales		*	1249080000	38.13
Cupressaceae	Cupressales		*		
Rosaceae	Rosales		*	1010810000	52.21
Rubiaceae	Gentianales		*	639314000	44.27
Apocynaceae	Gentianales		*	232603000	45.49
Primulaceae	Ericales		*	4057080000	66.8
Pentaphylacaceae	Ericales		*	1937490000	50.02
Fagaceae	Fagales		*	1238250000	48.81
Rosaceae	Rosales		*	1893970000	79.63
Araliaceae	Apiales		*	1299320000	63.9

Pteridaceae	Polypodiales		*		
Moraceae	Rosales		*	324851000	78.41
Thelypteridaceae	Polypodiales		*	8712070000	46.84
Primulaceae	Ericales		*	1831240000	71.07
Lamiaceae	Lamiales		*	451912000	53.94
Rosaceae	Rosales		*	316685000	38.16
Blechnaceae	Polypodiales		*		
Lindsaeaceae	Polypodiales		*	4616640000	61.15
Cupressaceae	Cupressales		*		
Commelinaceae	Commelinales		*		
Fabaceae	Fabales				
Lygodiaceae	Schizaeales		*		
Rubiaceae	Gentianales		*	423361000	50.25
Asphodelaceae	Asparagales		*	1918350000	66.65
Ranunculaceae	Ranunculales		*		
Fabaceae	Fabales		*	939369000	52.14
Lindsaeaceae	Polypodiales		*		
Fagaceae	Fagales		*	894762000	41.32
Smilacaceae	Liliales		*	4849950000	45.36
Vitaceae	Vitales		*	2084620000	52.21
Polypodiaceae	Polypodiales		*		
Zingiberaceae	Zingiberales		*	2416810000	66.33
Orchidaceae	Asparagales		*	8391010000	45.04
Gnetaceae	Gnetales		*	7372860000	38.14
Fabaceae	Fabales		*	707302000	62.74
Convolvulaceae	Solanales		*	6674600000	53.66
Fabaceae	Fabales		*	1238610000	67.43
Fagaceae	Fagales		*	898455000	55.05
Campanulaceae	Asterales		*	1153590000	47.98
Rosaceae	Rosales		*	296863000	52.9
Poaceae	Poales		*	1670430000	62.63
Rosaceae	Rosales		*	519425000	41.98
Rosaceae	Rosales	§	*	241918000	45.59
Euphorbiaceae	Malpighiales		*	2687230000	69.6
Menispermaceae	Ranunculales		*	680819000	56.26
Araliaceae	Apiales		*	525375000	45.31
Plantaginaceae	Lamiales		*	1693790000	74.1
Caricaceae	Brassicales	§	*	492239000	70.52
Primulaceae	Ericales		*	702969000	48.28
Malvaceae	Malvales		*	668597000	37.85
Primulaceae	Ericales		*	4152790000	65.02
Phyllanthaceae	Malpighiales		*	1065010000	55.19
Rubiaceae	Gentianales		*	6559920000	47.17
Rosaceae	Rosales		*	1379720000	78.79
Cyperaceae	Poales		*	505064000	47.7
Polypodiaceae	Polypodiales		*		
Primulaceae	Ericales		*	3630840000	67.07
Anacardiaceae	Sapindales		*	416496000	49.21
Dennstaedtiaceae	Polypodiales		*	8176600000	32.23
Poaceae	Poales		*	1658430000	76.32

Rutaceae	Sapindales		*	513000000	58.62
Fabaceae	Fabales		*	1855360000	69.72
Loranthaceae	Santalales		*	10238300000	22.63
Euphorbiaceae	Malpighiales		*	6670760000	54.24
Moraceae	Rosales		*	290690000	29.67
Fabaceae	Fabales		*		
Rubiaceae	Gentianales		*	1071700000	59.99
Orchidaceae	Asparagales		*	3833140000	46.25
Solanaceae	Solanales		*	1161080000	47.84
Urticaceae	Rosales		*	1954650000	75.64
Lindsaeaceae	Polypodiales		*	5439090000	60.83
Poaceae	Poales		*	586183000	53.92
Cyperaceae	Poales		*	973469000	60
Pontederiaceae	Commelinales		*	2027320000	38.76
Asteraceae	Asterales		*	2168470000	70.06
Araceae	Alismatales		*	4406250000	62.86
Cucurbitaceae	Cucurbitales		*	1302730000	56.82
Euphorbiaceae	Malpighiales		*	1427910000	46.39
Campanulaceae	Asterales		*	462251000	52.75
Anacardiaceae	Sapindales		*	729917000	59.69
Phyllanthaceae	Malpighiales		*	3711820000	77.27
Menyanthaceae	Asterales		*	604648000	54.14
Caryophyllaceae	Caryophyllales		*		
Asteraceae	Asterales		*	519835000	51.51
Cyperaceae	Poales		*	434009000	39.24
Lamiaceae	Lamiales		*	316114000	36.09
Euphorbiaceae	Malpighiales		*	1097200000	49.47
Phyllanthaceae	Malpighiales		*	1292370000	64.04
Fabaceae	Fabales	§	*	435187000	29.58
Actinidiaceae	Ericales		*	4061370000	42.5
Meliaceae	Sapindales		*	482459000	42.9
Sabiaceae	Proteales		*	8860730000	35.81
Betulaceae	Fagales		*	1997410000	80.39
Fabaceae	Fabales		*	952186000	46.71
Pinaceae	Pinales		*		
Pittosporaceae	Apiales		*	565537000	56.21
Zingiberaceae	Zingiberales		*	3037730000	68.24
Hypoxidaceae	Asparagales		*	1475320000	69.21
Stemonaceae	Pandanales		*	866785000	49.26
Ranunculaceae	Ranunculales		*		
Arecaceae	Arecales		*	3134170000	58.48
Fagaceae	Fagales		*	774261000	43.88
Malvaceae	Malvales		*	443715000	47.82
Euphorbiaceae	Malpighiales		*		
Fabaceae	Fabales		*	2071950000	55.87
Annonaceae	Magnoliales		*	778362000	52.43
Rutaceae	Sapindales				
Phyllanthaceae	Malpighiales		*	3558940000	61.35
Sapindaceae	Sapindales		*	1528090000	65.24
Malvaceae	Malvales		*	1457080000	65.81

Cucurbitaceae	Cucurbitales	*	655776000	58.02
Rubiaceae	Gentianales	*	1595620000	56.81
Actinidiaceae	Ericales	*	4921180000	56.02
Rutaceae	Sapindales	*	484862000	54.67
Fabaceae	Fabales	*	1207280000	55.98
Rubiaceae	Gentianales	*	1676400000	57.49
Primulaceae	Ericales	*	5310850000	63.61
Capparaceae	Brassicales	*	338496000	43.42
Sapindaceae	Sapindales	*	456439000	39.38
Apocynaceae	Gentianales	*	1194010000	57.23
Acanthaceae	Lamiales	*	2624150000	72.67
Salicaceae	Malpighiales	*	458262000	48.97
Anacardiaceae	Sapindales	*	413430000	37.8
Aristolochiaceae	Piperales	*		
Rutaceae	Sapindales	*	320181000	36.4
Fabaceae	Fabales	*	645290000	56.41
Capparaceae	Brassicales	*	974911000	66.98
Fabaceae	Fabales	*	724375000	45.82
Lamiaceae	Lamiales	*	407398000	47.63
Lamiaceae	Lamiales	*	1657400000	52.72
Cyatheaceae	Cyatheales	*	9729680000	34.91
Symplocaceae	Ericales	*	2339570000	67.99
Phyllanthaceae	Malpighiales	*	1054110000	53.9
Sabiaceae	Proteales	*	988067000	42.2
Dicksoniaceae	Cyatheales	*	7485160000	68.59
Convolvulaceae	Solanales	*	6888150000	53.53
Urticaceae	Rosales	*	721467000	58
Euphorbiaceae	Malpighiales	*	1037040000	59.15
Urticaceae	Rosales	*	375519000	55.52
Cyperaceae	Poales	*	1247170000	51.97
Convolvulaceae	Solanales	*	772156000	56.9
Orchidaceae	Asparagales	*		
Fabaceae	Fabales	*	387120000	38.58
Melanthiaceae	Liliales	*		
Campanulaceae	Asterales	*		
Oleaceae	Lamiales	*	397769000	36.64
Fabaceae	Fabales	*	592235000	44.05
Ranunculaceae	Ranunculales	*		
Asteraceae	Asterales	*	3972590000	47.93
Primulaceae	Ericales	*	2985340000	72.52
Rubiaceae	Gentianales	*		
Piperaceae	Piperales	*	591256000	43.67
Cucurbitaceae	Cucurbitales	*	708320000	44.62
Zingiberaceae	Zingiberales	*	1998150000	62.45
Staphyleaceae	Crossosomatales	*	3376800000	49.15
Rubiaceae	Gentianales	*	971859000	46.78
Araliaceae	Apiales	*	4424500000	59.12
Rubiaceae	Gentianales	*	932737000	63.3
Asteraceae	Asterales	*	3062020000	65.74
Zingiberaceae	Zingiberales	*	2187570000	68.94

Asparagaceae	Asparagales		*		
Colchicaceae	Liliales		*		
Rubiaceae	Gentianales		*	598024000	34.36
Orchidaceae	Asparagales		*	3212790000	48.07
Pteridaceae	Polypodiales		*		
Vitaceae	Vitales		*	5015510000	57.01
Lygodiaceae	Schizaeales		*		
Fabaceae	Fabales		*	617522000	42.48
Poaceae	Poales		*	2061290000	51.55
Orchidaceae	Asparagales		*	2755570000	50.46
Amaryllidaceae	Asparagales		*		
Asparagaceae	Asparagales		*		
Araliaceae	Apiales		*	3820320000	71.29
Amaryllidaceae	Asparagales		*		
Hydrangeaceae	Cornales		*	1980750000	57.15
Lamiaceae	Lamiales		*	584558000	50.86
Combretaceae	Myrtales				
Ranunculaceae	Ranunculales				
Meliaceae	Sapindales				
Fabaceae	Fabales				
Fabaceae	Fabales				
Rhamnaceae	Rosales				
Malpighiaceae	Malpighiales				
Ebenaceae	Ericales				
Fabaceae	Fabales				
Combretaceae	Myrtales		*	6449170000	62.63
Fabaceae	Fabales		*	523304000	53.09
Fabaceae	Fabales		*	533158000	46.17
Phyllanthaceae	Malpighiales		*	1122480000	69.71
Myrtaceae	Myrtales		*	1736990000	72.95
Theaceae	Ericales		*	792310000	54.92
Phyllanthaceae	Malpighiales		*	3685600000	77.68
Juglandaceae	Fagales		*	1293590000	36.67
Juglandaceae	Fagales		*	1558520000	38.17
Araceae	Alismatales		*		
Apocynaceae	Gentianales		*	592138000	39.16
Symplocaceae	Ericales		*	7365240000	73.03
Phyllanthaceae	Malpighiales		*	3812660000	64.28
Hypericaceae	Malpighiales	§	*	378401000	41.04
Rubiaceae	Gentianales		*	851932000	56.67
Malvaceae	Malvales		*	538123000	10.42
Theaceae	Ericales		*	802529000	38.72
Phyllanthaceae	Malpighiales		*	661306000	58.59
Rubiaceae	Gentianales		*	558599000	35.03
Plantaginaceae	Lamiales		*	523265000	41.63
Fabaceae	Fabales		*	677407000	57.19
Fabaceae	Fabales		*	1201420000	69.83
Euphorbiaceae	Malpighiales		*	8490870000	47.31
Annonaceae	Magnoliales		*	780335000	49.13
Cactaceae	Caryophyllales		*	1603090000	63.75

Zingiberaceae	Zingiberales		*	2360770000	66.19
Moraceae	Rosales		*	428745000	47.41
Lamiaceae	Lamiales		*	451230000	36.07
Cucurbitaceae	Cucurbitales		*	632049000	53.34
Meliaceae	Sapindales		*	450766000	50.66
Vitaceae	Vitales		*	4048710000	49.39
Lauraceae	Laurales		*	2380510000	51.55
Asteraceae	Asterales		*	3326930000	60.62
Lamiaceae	Lamiales		*	1571400000	58.98
Araliaceae	Apiales		*	6846210000	65.87
Lamiaceae	Lamiales		*	1384470000	65.72
Cornaceae	Cornales		*	1488970000	80.84
Malvaceae	Malvales		*		
Urticaceae	Rosales		*	1052590000	82.27
Phyllanthaceae	Malpighiales		*	1091910000	71.11
Urticaceae	Rosales		*	1573380000	54.9
Smilacaceae	Liliales		*		
Lamiaceae	Lamiales		*	1028020000	41.4
Smilacaceae	Liliales		*		
Poaceae	Poales		*	2009230000	48.6
Lamiaceae	Lamiales		*	785799000	59.01
Lamiaceae	Lamiales		*		
Ranunculaceae	Ranunculales		*	8581950000	20.21
Scrophulariaceae	Lamiales		*	805659000	51.86
Lamiaceae	Lamiales		*	1294170000	49.17
Rutaceae	Sapindales		*	1144230000	55.4
Campanulaceae	Asterales		*		
Lamiaceae	Lamiales		*	2044960000	68.82
Lamiaceae	Lamiales	§	*	313559000	30.34
Iteaceae	Saxifragales		*	433362000	57.27
Fabaceae	Fabales		*	2011480000	71.96
Rhamnaceae	Rosales		*	275433000	47.76
Oleaceae	Lamiales		*	2453170000	53.77
Araliaceae	Apiales		*		
Solanaceae	Solanales		*		
Rosaceae	Rosales		*	3684740000	67.78
Caprifoliaceae	Dipsacales		*	1765230000	63.25
Moraceae	Rosales		*	571654000	42.16
Poaceae	Poales		*		
Fabaceae	Fabales		*	1204200000	63.94
Convolvulaceae	Solanales		*		
Malvaceae	Malvales		*	1838280000	79.34
Lamiaceae	Lamiales		*	3465300000	60.27
Urticaceae	Rosales		*		
Apocynaceae	Gentianales		*	316944000	40.66
Moraceae	Rosales		*	1157700000	65.52
Apocynaceae	Gentianales	§	*	499540000	46.54
Asteraceae	Asterales		*	2077680000	56.84
Fabaceae	Fabales		*	863424000	50.45
Anacardiaceae	Sapindales		*	531361000	59.29

Anacardiaceae	Sapindales	*	451881000	63.17
Gnetaceae	Gnetales	*	4844220000	42.29
Rutaceae	Sapindales	*	266909000	35.34
Lythraceae	Myrtales	*	286328000	27.04
Moraceae	Rosales	*		
Fabaceae	Fabales	*	5068350000	51.28
Moraceae	Rosales	*	2245400000	70.55
Acanthaceae	Lamiales	*	987492000	68.44
Fabaceae	Fabales	*	368757000	38.39
Rhamnaceae	Rosales	*	313089000	34.55
Fabaceae	Fabales	*	861183000	49.61
Fabaceae	Fabales	*	1110800000	62.32
Combretaceae	Myrtales	*	3241930000	49.59
Celastraceae	Celastrales	*	1076290000	50.72
Moraceae	Rosales	*	1290330000	83.46
Salicaceae	Malpighiales	*	415170000	45.75
Meliaceae	Sapindales	*		
Euphorbiaceae	Malpighiales	*	867156000	54.03
Asteraceae	Asterales	*	1558400000	55.46
Asteraceae	Asterales	*	4966430000	47.78
Boraginaceae	Gentianales	*	5651530000	65.26
Rhamnaceae	Rosales	*	307192000	46.25
Lamiaceae	Lamiales	*	3077840000	88.07
Cornaceae	Cornales	*	1663270000	82.76
Myrtaceae	Myrtales	*		
Combretaceae	Myrtales	*	1859440000	47.58
Moraceae	Rosales	*	365675000	51.44
Orchidaceae	Asparagales	*	9212440000	47.08
Polypodiaceae	Polypodiales	*		
Moraceae	Rosales	*	1471430000	64.9
Smilacaceae	Liliales	*	4615390000	39.32
Rosaceae	Rosales	*	2235070000	57.03
Onagraceae	Myrtales	*	1160210000	78.05
Rutaceae	Sapindales	*	2252790000	60.22
Euphorbiaceae	Malpighiales	*	1254670000	63.76
Rubiaceae	Gentianales	*	2788090000	76.08
Moraceae	Rosales	*	429521000	52.76
Euphorbiaceae	Malpighiales	*	524631000	51.96
Fabaceae	Fabales	*	1409810000	42.81
Euphorbiaceae	Malpighiales	*	1301990000	65.8
Moraceae	Rosales	*	2447600000	68.98
Bignoniaceae	Lamiales	*	396924000	42.12
Arecaceae	Arecales	*	1697330000	54.42
Asparagaceae	Asparagales	*		
Arecaceae	Arecales	*	1560170000	53.88
Asteraceae	Asterales	*		
Oleaceae	Lamiales	*	625902000	58.44
Euphorbiaceae	Malpighiales	*	5737440000	39.23
Capparaceae	Brassicales	*	1184570000	51.56
Urticaceae	Rosales	*	566851000	64.16

Adoxaceae	Dipsacales	*		
Polygonaceae	Caryophyllales	*	1623200000	57.34
Moraceae	Rosales	*	423035000	46.21
Acanthaceae	Lamiales	*	789891000	58.43
Moraceae	Rosales	*	426343000	54.91
Phyllanthaceae	Malpighiales	*	600709000	61.61
Araceae	Alismatales	*	4054850000	51.6
Dioscoreaceae	Dioscoreales	*	587797000	70.69
Combretaceae	Myrtales	*	954909000	37.02
Pontederiaceae	Commelinales	*	2921620000	27.91
Lauraceae	Laurales	*	1501280000	52.14
Euphorbiaceae	Malpighiales	*	619970000	50.79
Moraceae	Rosales	*	315893000	45.63
Loranthaceae	Santalales	*		
Simaroubaceae	Sapindales			
Malvaceae	Malvales	*		
Aspleniaceae	Polypodiales	*		
Moraceae	Rosales	*	310469000	34.2
Meliaceae	Sapindales	*	642653000	43.3
Marantaceae	Zingiberales	*	1309660000	72.14
Euphorbiaceae	Malpighiales			
Combretaceae	Myrtales	*	992432000	39.86
Araceae	Alismatales	*		
Annonaceae	Magnoliales	*	981013000	51.47
Asparagaceae	Asparagales	*		
Cannabaceae	Rosales	*	551396000	43.03
Myristicaceae	Magnoliales	*		
Annonaceae	Magnoliales	*		
Polypodiaceae	Polypodiales	*		
Tectariaceae	Polypodiales	*		
Annonaceae	Magnoliales			
Meliaceae	Sapindales	*	1068860000	48.6
Iridaceae	Asparagales	*	1903790000	47.47
Apocynaceae	Gentianales	*	1397980000	70.8
Araliaceae	Apiales	*	1875850000	62.26
Juglandaceae	Fagales	*	679573000	41.12
Malvaceae	Malvales	*	834025000	54.23
Fabaceae	Fabales	*	710645000	47.6
Lamiaceae	Lamiales	*	3231850000	38.36
Phyllanthaceae	Malpighiales	*	3458820000	73.89
Rubiaceae	Gentianales	*	424589000	33.62
Fabaceae	Fabales	*	923942000	63.42
Araceae	Alismatales	*		
Adoxaceae	Dipsacales	*		
Aspleniaceae	Polypodiales	*		
Gnetaceae	Gnetales	*	7398880000	38.3
Malvaceae	Malvales	*	1305450000	35.51
Elaeocarpaceae	Oxalidales	*	573689000	35.56
Rubiaceae	Gentianales	*	2009140000	53.76
Fagaceae	Fagales	*	943280000	41.13

Arecaceae	Arecales	*	5610760000	53.9
Magnoliaceae	Magnoliales	*	2064330000	42.81
Rubiaceae	Gentianales			
Meliaceae	Sapindales	*		
Malvaceae	Malvales	*		
Menispermaceae	Ranunculales	*	546554000	48.33
Arecaceae	Arecales	*	2176380000	64.02
Arecaceae	Arecales	*	1635870000	74.62
Arecaceae	Arecales	*	2558650000	61.57
Meliaceae	Sapindales	*	1019120000	46.17
Malvaceae	Malvales	*	1320430000	49.36
Fagaceae	Fagales	*	1006700000	61.99
Moraceae	Rosales	*	714993000	52.05
Arecaceae	Arecales	*	1574930000	57.12
Araceae	Alismatales	*		
Elaeocarpaceae	Oxalidales	*	651235000	34.22
Campanulaceae	Asterales	*	880213000	57.09
Urticaceae	Rosales	*	485813000	59.99
Orchidaceae	Asparagales	*	1354940000	61.51
Rubiaceae	Gentianales	*	1608250000	57.92
Poaceae	Poales	*	1397340000	44.98
Poaceae	Poales	*	1398210000	43.36
Poaceae	Poales	*	1372700000	49.26
Poaceae	Poales	*	1307140000	64.98
Poaceae	Poales	*	1373230000	54.65
Poaceae	Poales	*	2368280000	63.77
Poaceae	Poales	*	1403000000	43.45
Styracaceae	Ericales	*	879915000	47.88
Poaceae	Poales	*	2611270000	42.17
Primulaceae	Ericales	*		
Arecaceae	Arecales	*		
Arecaceae	Arecales	*	2186900000	59.14
Arecaceae	Arecales	*	3144450000	57.15
Arecaceae	Arecales	*	1093330000	46.8
Asparagaceae	Asparagales	*		
Moraceae	Rosales	*	424149000	50.48
Asteraceae	Asterales	*	7162030000	79.57
Orchidaceae	Asparagales	*	4687340000	77.91
Fabaceae	Fabales	*	831880000	48.98
Poaceae	Poales	*	1349100000	43.77
Poaceae	Poales	*	2734970000	62.12
Poaceae	Poales	*	1993150000	41.23
Poaceae	Poales	*	2533260000	42.32
Poaceae	Poales	*	1531140000	48.92
Poaceae	Poales	*	2642270000	42.74
Cyperaceae	Poales	*	4805250000	48.81
Taxaceae	Cupressales	*	1688360000	51.69
Polygonaceae	Caryophyllales	*	4019060000	51.04
Poaceae	Poales	*	5733010000	67.01
Poaceae	Poales	*	2842680000	47.62

Poaceae	Poales	*		
Poaceae	Poales	*	1041810000	55.53
Poaceae	Poales	*	2169130000	62.6
Poaceae	Poales	*	6416680000	46.84
Poaceae	Poales	*	2874470000	47.68
Poaceae	Poales	*	5501390000	42.15
Poaceae	Poales	*	2811400000	47.68
Saururaceae	Piperales	*	2401780000	68.73
Polygonaceae	Caryophyllales	*	1824350000	67.74
Cucurbitaceae	Cucurbitales	*	1043280000	66.66
Fabaceae	Fabales	*	1238480000	55.36

heterozygosity (%)
5.88
1.92
3.81
8.05
6.1
3.91
7.22
3.1
11.5
3.38
3.75
2.14
3.91
6.85
1.93
1.58
9.82
4.45
3.25
8.54
2.71
10.26
4.92
4.21
3.82
14.85
1.9
1.36
6.23
1.36
5.84
7.22
7.27
3.84
5.33
12.26
0.75
1.73
1.03
12.64
8.84
9.83

8.29
8.57
3.01
11.95
12.92
3.48
7.22
8.24
13.69
13.3
7.57
21.45
10.37
1.84
1.17
10.5
1.3
12.64
4.83
11.43
13.29
10.34
4.46
16.76
6.72
10.89
10.42
9.24
4.11
5.86
5.56
6.35
9.96
12.98
4.55
12.72
8.8
3.57
2.5
7.32
5.26
10.68
9.21
10.66
3.69
0.74
9.33

1.09
10.17
14.65
10.81
8.06
4.45
4.02
8.42
2.12
8.92
8.84
0.34
12.85
1.29
6.54
5.2
8.65
2.78
1.93
9.46
0.87
2.71
2.55
1.53
21.72
3.39
4.3
3.3
7.16
3.59
1.76
4.61
32.36
3.88
1.61
1.42
1.87
13.26
8.08
8.44
11.75
1.54
3.12
8.85
2.29
2.56
5.83
1.3
1.52

2.48
0.15
1.4
10.23
0.69
8.46
4.51
3.15
3.39
3.69
1.48
12.83
4.82
11.19
2.7
1.09
0.91
7.01
28.22
6.38
2.18
14.97
9.51
0.25
1.57
0.68
0.55
14.15
1.37
2.08
1.55
11.66
12.03
4.13
1.2
4.45
4.11
10.83
8.01
3.61
2.18
0.44
9.43

3.49
1.05
5.58
10.69
10.63
2.44
1.56
1.68
7.46
9.55
12.07
10.43
14.55
6.92
7.03
3.11
8.49
18.89
2.28
12.71
7.9
9.01
9.56
14.44
6.03
11.76
6.81
6.97
2.68
8.77
6.25
3.3
13.33
5.04
3.89
2.8
1.83
9.03
7
18.96
10.08
9.61
4.42
7.54
12.81

1.4
12.77
8.42
3.42
10.58
4.5
2.52
1.24
0.99
1.39
1.92
1.3
5.14
5.76
4.52
5.45
11.99
1.38
1.76
2.13
1.58
1.29
9.72
2.98
8.15
9.26
2.08
13.14
9.66
1.85
15
1.58
1.29
2.9
6.68
0.38
2.21
5.67
12.79
8.96
11.94
10.92
3.8
1
6.23

4.61
8.82
5.71
4.96
1.47
6.75
6.03
5.68
2.05
1.39
1.55
0.18
1.83
1.63
1.44
0.62
5.31
3.86
1.13
1.9
3.65
9.7
1.68
8.82
7.29
21.3
4.38
3.73
5.14
1.97
1.57
3.32
0.66
1.51
3.92
1.25
5.37
0.49
7.06
9.3
4.25
1.8
5.12
3.91
5.05

15.01
19.88
14.07
4.55
1.52
6.01
9.47
2.62
3.75
14.77
15.55
8.9
5.88
30.15
18.41
12.68
4.68
1.34
12.68
8.18
8.14
1.66
14.26
9.72
1.54
9.8
1.35
0.38
8.05
10.07
10.04
5.14
11.27
3.17
3.9
2.69
3.36
8.18
18.65
1.29

11.1
0.61
10.86
6.16
17.87
4.25
6.26
3.1
1.06
3.01
12.9
1.62
2.96
1.92
3.37
3.92
7.03
2.45
10.06
2.82
11.79
4.6
6.66
5.57
2.78
2.69
2.26
2.84
12.95
26.48
3.06
10.41
9.65
3.88
11.59
4.85
11.06
12.02
8
3.23
6.09
1.72
3.02
5.81

11.33
5.47
1.22
4.44
4.95
13.75
10.42
14.41
12.17
1.61
1.68
9.18
15.82
5.66
0.7
1.19
0.82
7.85
0.65
23.41
11.4
11.61
7.47
1.3
4.49
5.75
7.08
10.42
1.01
6.79
5.66
10.48
1.53
9.09
1.19
6.21
5.05
3.34
5.17
8.43
15.05
10.22
10
4.45

15.98
8.61
13.52
9.77
10.06
3.23
1.52
13.57
1.67
4.25
12.83
6.1
11.88
1.1
13.03
2.09
4.05
1.05
6.67
3.55
1.01
3.81
5.09
1.32
12.53
11.61
7.86
1.59
13.77
1.39
12.47
1.83
1.6

5.89
7.22
7.24
12.33
6.36
13.97
3.46
4
6.14
3.06
5.42
0.97
4.38
12.18
2.6
11.68
9.93
5.77
20.33
9.24
4.78
13.23
4.77
6.2
8.03
2.16
14.59
8.14
1.96
9.52
4.89
5.28
6.76
3.25
13.13
3.05
5.25
4.1
12.72
10.54

10.61
13.58
11.17
11.17
2.88
5.28
4.76
6.33
11.8
8.73
9.16
3.09
4.6
2.91
13.18
11.53
5.74
12.94
15.98
8.9
4.68
5.81
13.21
10.04
3.78
10.2
6.73
2.92
4.42
6.83
3.31
1.22
12.75
9.94
1.28
5.5
3.32
17.59
6.49
3.67
8.97
3.94
2.81
6.98

6.66
10.53
11.81
10.17
10.59
11.84
6.68
8.91
6.23
10.64
5.17
15.26
13.31
7.87
3.61
9.94
10.65
8.42
9.69
2.06
15.43
15.16
5.96
8.38
10.28
5.05
3.65
13.2
13.71
3.47
10.93
1.93
4.56
13.86

15.46
7.64
6.79
7.75
10.05
6.24
4.3
10.66
12.14
7.11
2.61
0.97
9.49
14.73
2.92
7.15
4.53
4.77
5.87
6.83
8.34
2.03
5.04
8.91
5.11
9.55
6.46
4.01
12.35
36.6
4.71
9.35
9.85
1.69
1.65
2.05
6.21
2.41
2.78
12.05
2.97
1.31
13.8

1.91
1.59
6.31
3.02
9.25
2.65
12.9
12.05
4.75
2.49

Figure
Click here to access/download;Figure;Figure 1.pdf

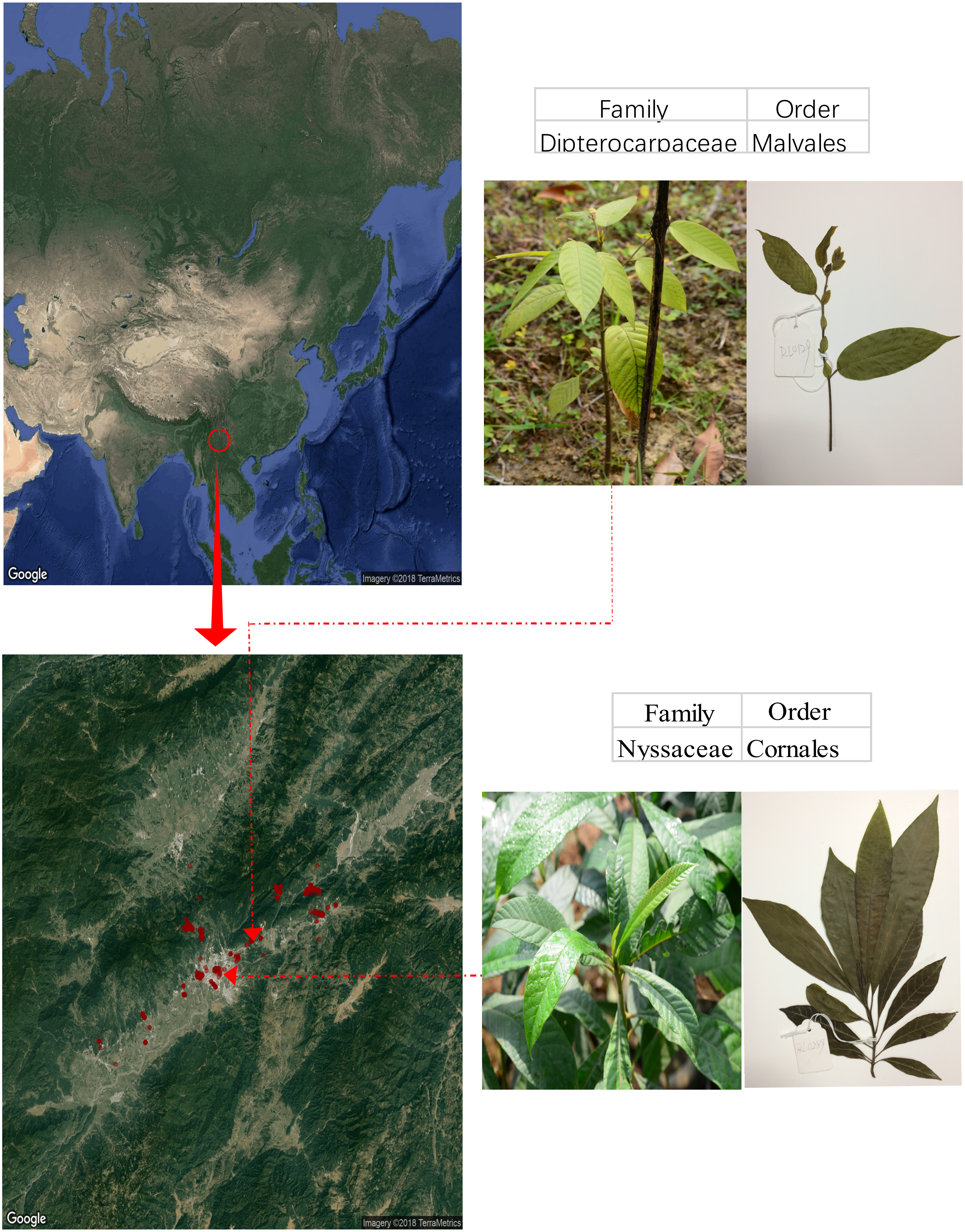

Figure

Click here to access/download;Figure;Figure 2.pdf

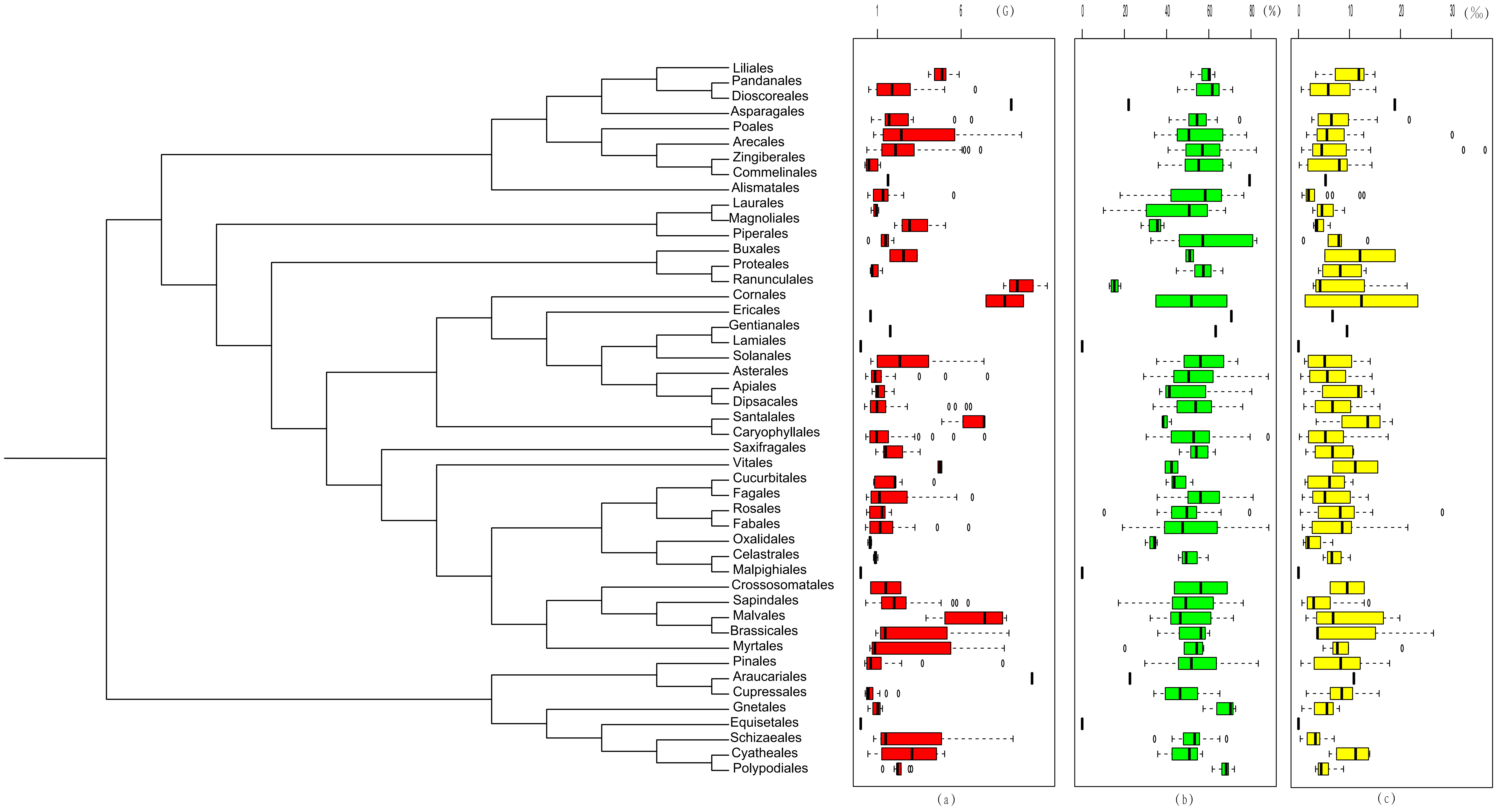

Figure

Click here to access/download;Figure;Figure 3.pdf

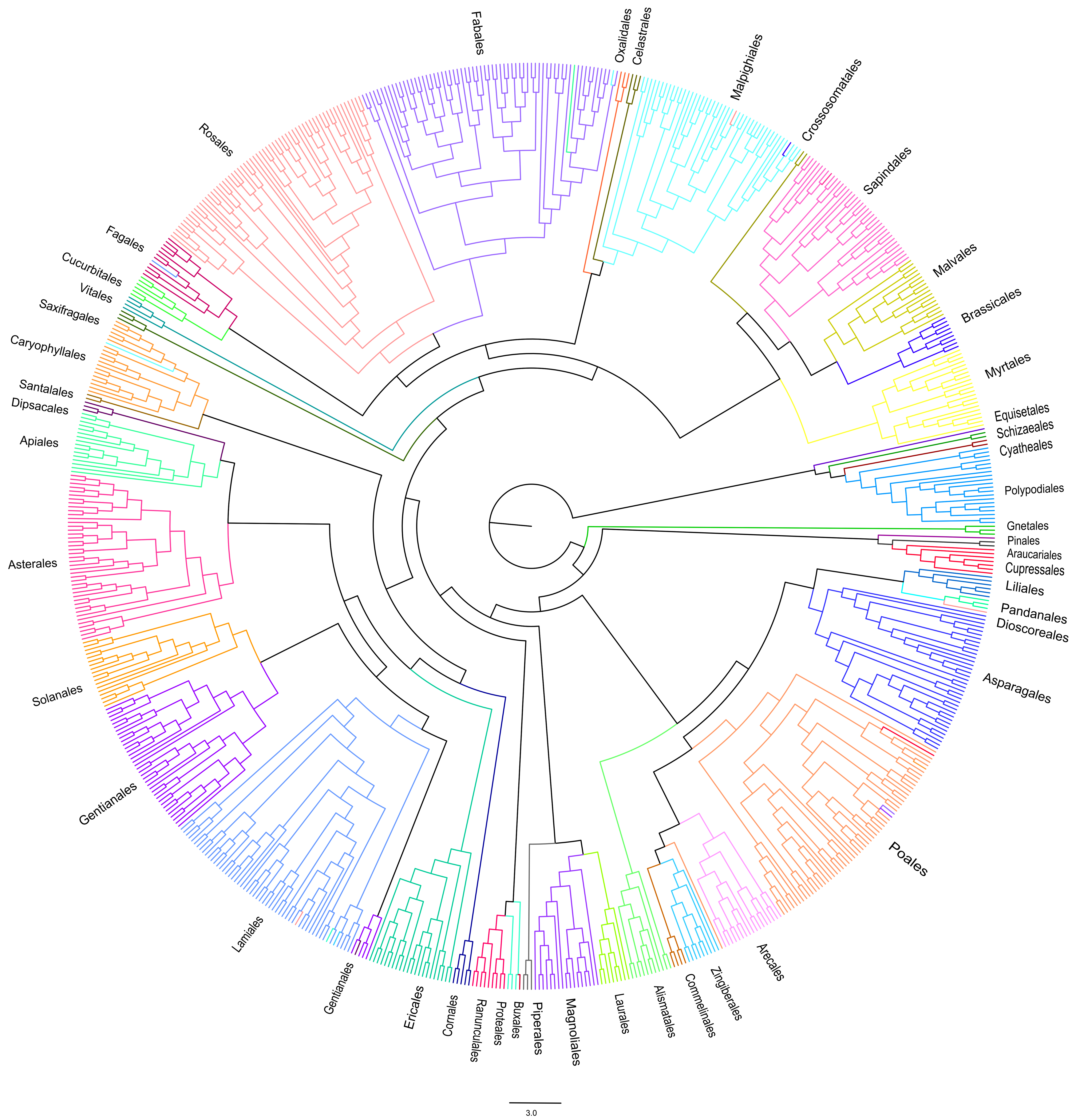

Figure

Click here to access/download;Figure;Figure S1.pdf

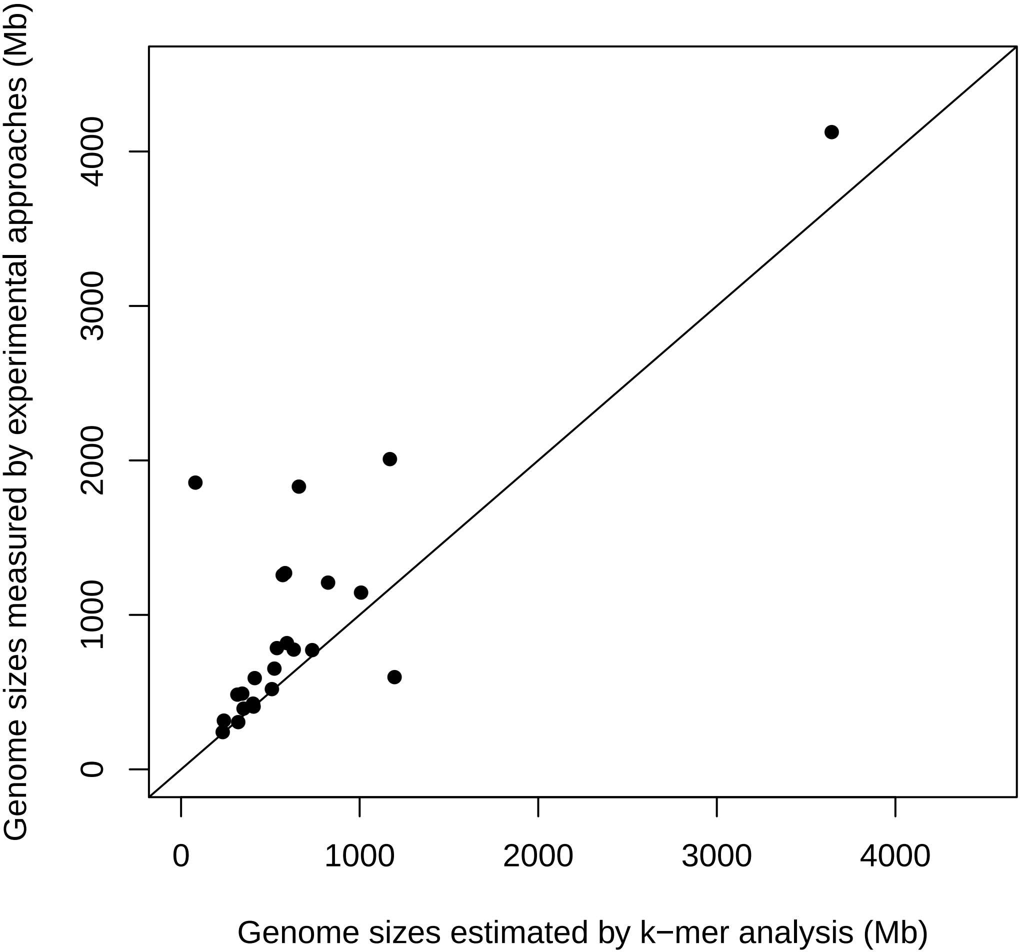

Click here to access/download
Supplementary Material
Table S2.xlsx

Click here to access/download
Supplementary Material
Table S3.xlsx

Click here to access/download
Supplementary Material
Table S4.xlsx
