# Supplementary material for: Molecular digitization of a botanical garden: high-depth whole-genome sequencing of 689 vascular plant species from the Ruili Botanical Garden
Source: Gigascience. 2019 Jan 25;8(4):giz007. doi: 10.1093/gigascience/giz007 (PMC6441391; doi:10.1093/gigascience/giz007)
Supplement: GIGA-D-18-00121_Revision_1.pdf [file giz007_giga-d-18-00121_revision_1.pdf]

GigaScience

Molecular Digitization of a Botanical Garden: High-depth whole genome sequencing of 689 vascular plants from the Ruili Botanical Garden

--Manuscript Draft--

**Manuscript Number:**	GIGA-D-18-00121R1
**Full Title:**	Molecular Digitization of a Botanical Garden: High-depth whole genome sequencing of 689 vascular plants from the Ruili Botanical Garden
**Article Type:**	Data Note
**Funding Information:**	the Shenzhen Municipal Government of china (JCYJ20150529150505656) — Dr. Xin Liu<br>the Shenzhen Municipal Government of China (JCYJ20150831201643396) — Dr. Yue Chang<br>The Construction of China National GeneBank (Yunnan GeneBank) (2015DA008) — Dr. Le Cheng<br>State Key Laboratory of Agricultural Genomics (2011DQ782025) — Dr. Huan Liu<br>Guangdong Provincial Key Laboratory of Genome Read and Write (2017B030301011) — Dr. Wangsheng Li
**Abstract:**	Background<br>Genome sequencing has been widely used in plant research to construct reference genomes and elucidate evolutionary insights. However, only a limited number of plant species have had their whole genome sequenced, and the limited taxon information of these species has further restrained the utility of these data.<br><br>Findings<br>Here, we comprehensively sampled and sequenced vascular plant species of growing in the Ruili Botanical Garden, located in South West China. We sequenced 761 samples out of the total 1,093 collected voucher specimens stored in the Herbarium of China National GeneBank (HCNGB). These 761 samples represented 689 vascular plant species from 134 families belonging to 47 orders. Of these, 254 samples were identified to species level and 506 samples were identified to families using chloroplast sequences. In total, we generated 54 Tb of sequencing data, which resulted in an average sequencing depth of 60X per species, as estimated by the genome size. A reference phylogeny was reconstructed with 78 chloroplast genes for molecular identification and other possible applications.<br><br>Conclusions<br>In this study, we generated a large dataset of vascular plant genomes, with both high-depth whole genome sequencing data and associated voucher specimens, making it a valuable dataset for plant genome research and other applications. This project also provides insight into the feasibility and technical requirements for “planetary scale” projects such as the 10 thousand Plant Genome Project (10KP) and the Earth BioGenome Project (EBP).
**Corresponding Author:**	Xin Liu, Ph.D.<br>BGI<br>CHINA
**Corresponding Author Secondary Information:**	
**Corresponding Author's Institution:**	BGI
**Corresponding Author's Secondary Institution:**	
**First Author:**	Huan Liu

Powered by Editorial Manager® and ProduXion Manager® from Aries Systems Corporation

First Author Secondary Information:	
Order of Authors:	Huan Liu
	jinpu Wei
	Ting Yang
	Weixue Mu
	Bo Song
	Tuo Yang
	Yuan Fu
	Xuebin Wang
	Guohai Hu
	Wangsheng Li
	Hongcheng Zhou
	Yue Chang
	Xiaoli Chen
	Hongyun Chen
	Le Cheng
	Xuefei He
	Hechen Cai
	Xianchu Cai
	Mei Wang
	Yang Li
	Sunil Kumar Sahu, PhD.
	Jinlong Yang
	Yu Wang
	Ranchang Mu
	Jie Liu
	Jianming Zhao
	Ziheng Huang
	Xun Xu, PhD
	Xin Liu, Ph.D.
Order of Authors Secondary Information:	
Response to Reviewers:	Reviewer reports:<br><br>Reviewer #1: The authors present an awesome quantity of whole genome sequence data representing hundreds of plant species. That the data is being made openly available represents a substantial contribution to the research community. Clearly the analysis of the data is a work in progress. Only 17 of the samples are subjected to whole genome assembly; for 738 just the chloroplast genomes were assembled. The sampling strategy was not exactly ideal: the plants were collected from a single botanic garden, and as such you might expect a somewhat arbitrary selection of species; given the numbers involved and the intent of the study this is perhaps not so important. What is more surprising is that the identifications of the plants is so limited. If these are part of a curated collection, you might expect them to be determined at least to genus; if it represents wild-collected specimens, you would ideally consult (local) botanical expertise for more precise determinations. Certainly for this kind of study it would

increase the value of the data markedly to know from what organisms the sequences originated. Since there are voucher specimens lodged in herbaria, and it seems considerable resources will be available - images etc. - the identifications can presumably still be achieved and the database updated. It just seems a bit odd; but if the paper is viewed as the presentation of a vast quantity of usable semi-raw data then perhaps this is OK too. I have not assessed the data itself and have no great insight to provide into the bioinformatic methods - they look superficially pretty standard. The authors have compared previously known genome sizes to that inferred from their data and it appears similar; although only 17 of the genomes were assembled (those with lower heterozygosity and repeats; apparently assemblies of more will be added later) those each included around 90% of benchmarking universal single-copy orthologs. Since the data, from raw reads
onwards should be available, the community will be in a position to assess it in detail post publication.

1. "We then compared these assembled chloroplast genomes and constructed the phylogenetic tree using the gene trees and translated to the species tree"
You have used astral to infer a species tree from the plastid data? This seems inappropriate in principle; the data ought to be linked and could be combined e.g. under ML to infer a tree without using a coalescence-based method. Plus, if there is conflict in this dataset, coalescence is not the best way to model it I would argue.
Response:
Thank you for the nice suggestion. Yes, we used ASTRAL to infer a species tree in the earlier version of this article. We also combined all genes to super data and used two methods for tree construction, one is by RAxML using GTRCAT model, another one is by IQ_TREE using the best model (GTR+F+R10) to construct the species tree. We have compared these two results in the revised article.

2. The conclusion towards the end of the data description (although it instead feels like discussion material): "deep learning can be applied to develop plant identification using this dataset as a good training set" seems a little far-fetched in this context. Huge as it is, I'm not convinced that this dataset represents useful input for this kind of application - the plants would need to be reliably determined, at the least, and I believe image-based IDs need a great deal more training data.

Response:
Thank you for the suggestion. We have now revised the sentence as per your suggestion as follows:

3.Discussion:
"The evolution of vasculature was a major event in plant history."
This is not directly relevant - I'd suggest to consider an alternative leading sentence here.

Response:
Thank you for the suggestion. We have removed this sentence from the main text and replaced with “The current understanding on the evolution of plants and its diversity in a phylogenomic context is limited due to the non-availability of genome-scale information across phylogenetically diverse species.”

4. "We only constructed a single short insert size library for each of the species and generated ~60 Gb whole genome sequencing data. It would be insufficient to assemble good draft genomes for majority of the species just based on single library data, because previous efforts to assemble reference genomes based on second generation sequencing data would require multiple short insert size libraries and also mate pair (large insert size) libraries. However,"
I don't understand what you're saying here. Are you saying that it would not have been possible to assemble genomes from much of the current data (surely not?)? Or are you suggesting that something has changed and that although in the past it would not have been possible, now it is? The lack of long reads marks a difference between this effort and that proposed in the 10KP project; how are the data likely to be comparable?
Response:
Yes, we do agree with your comments. Though a single library can be used to

assemble a genome, but may not yield a high-quality genome assembly. However, the current data was potentially used for several analyses such as gene finder, plastid and mitochondrial assembly. At present, we are using these data in combination with 10x genomics to get high quality genome data.

5. "With particular relevance for the 10KP, this study provided experiences for plant sampling, sample logistics, sample management, DNA extraction, sequencing library preparation, sequencing, data analysis and data management."
What exactly was learnt about all this? Qualification needed here - I have no doubt the authors gained a great deal of insight in the process of generating this huge dataset and it would be well worth sharing.
Response:
We have optimized the DNA extraction protocol and also published the protocol (Wu and Yang, 2018). Soon we will also launch the DNA extraction kit.
Wu C and Yang T. DNA Extraction for plant samples by CTAB. Gigascience. 2018; doi:10.17504/protocols.io.pzqdp5w

We also have just finished a guideline about sample submission for 10KP which including sample preparation (fresh sample, DNA sample and RNA sample), sample packing and shipping. The specific guidelines will be soon available in our 10KP website (https://db.cngb.org/10kp/).

6. "DNA Extraction [27] and BGISEQ-500 WGS library construction protocols can be found in protocols.io [28]."
Are these the appropriate references...?
Response: Yes, these are valid and appropriate references.

Table 1: does it really make sense to summarize this by order? Surely the quantities of raw data are only comparable if you break that down to families/genera/species/individuals?
Response:
Thank you for raising this question. For every sample, the sequencing quality and results were highly similar. Hence, we summarized the data by “Order” in Table 1. However, the detailed breakdown of the data at families/genera/species/ level will presented in our subsequent manuscript with higher quality genome assemblies for the selected species.

Reviewer #2: This paper sequences and assembles the genomes of diverse plant species found at the Ruili Botanical Garden, China. The authors generate approximately 60 Gb short sequence read data from 760 samples, and perform a range of analyses suitable for low-coverage draft genomes such as repeat content characterization and plastid assembly. These analyses show considerable variation in many genomic properties across plants. While the paper is descriptive, and is limited by using the same sequencing approach and assembly parameters for all samples, I do think it is impressive in terms of the scale of sequencing, and will serve as a good test case for large-scale sequencing of diverse sample sets.

Major comments
1. This paper is likely to be used as a test case for sequencing diverse samples across a flora. As such, I'm sure the reader would like to know the relationship between coverage and contiguity in the assemblies. Also, were mitochondrial genome assemblies attempted, and were these successful? More generally, it would be useful to explain why the same sequencing effort was allocated to all samples regardless of known genome size (and would you suggest others follow this approach in the future?).
Response
In this article, we have mentioned that ~70 Gb of raw sequencing data was generated for each of the sample. Combined with estimated genome size by GCE and kmergenie we can infer the genome coverage. For mitochondrial genome, we completed the initial test run, and successfully assembled the mitochondrial genome of five Species. Based on these positive assemblies, we have now actually initiated the mitochondrial genome assembly of remaining species.

Powered by Editorial Manager® and ProduXion Manager® from Aries Systems Corporation

	To calculate the genome size, only 10-30X data is usually enough, that's why we sequenced ~70 Gb as the preliminary step. Of course, for some samples with large genome size, we couldn't estimate the genome size, but their sequencing data was used in assembling the chloroplast genomes, as well as some conserved nuclear genes. Hence, once we obtained the genome size information, then we selected the appropriate method for the genome assembly. Overall, based on our experience, the sequencing method employed in this study can be recommended for the smaller genomes only.<br><br>2. As far as I'm aware, Astral is a species tree approach that should be used on many independent loci. I don't think it's suitable for the plastid as all loci are linked. An alternative phylogenetic approach should be used (such as partitioned analysis in IQ-TREE).<br>Response<br>Yes, we agree with your suggestion that Astral is not a suitable method for our analysis. We now combined all genes to super data and used two different methods for the tree construction, one is by RAxML using GTRCAT model, another one is by IQ_TREE using the best model to construct the species tree. We have compared these two results in the revised article.<br><br>3. The quality of the writing and clarity of some sentences could be improved.<br>Response:<br>Thank you for the suggestion. We have thoroughly revised the manuscript, and also followed the specific suggestions given by Reviewer#3 on writing part. In addition, our manuscript was proof read by a native English speaker.<br><br>4. This is the first time I've seen repeat content and heterozygosity summarised across such a broad sample set, and I'd be interested in more interpretation of these results. Presumably you'd consider these to be extremely labile across plants? Given that lability, I wonder the suitability of summarising this across species within a family (as shown in the box plots)?<br>Response:<br>We have summarized the information on repeat content and heterozygosity for every individual, both in the result section as well in the Table S1.<br><br>Minor comments<br><br>5. Line 52. Next generation sequencing technologies?<br>Response: We have revised the sentence as "With the advent of next generation sequencing technologies, enormous efforts have been made to sequence whole genomes of plant species, thereby providing new insights on plant evolution [1]"<br><br>6. Line 81. Genome size and repeat content estimates are possible from genome skim data (for example, see RepeatExplorer), so this critique doesn't seem fair.<br>Response: Thank you for the suggestion. We do agree with your comment that "repeat content estimates are possible from the genome skim data via RepeatExplorer program". However, Genome size estimation is only possible for the plants with small genome size. Anyway, we have revised the sentence to avoid the confusion as follows: "However, previous genome skimming studies have only generated a small amount of sequencing data for the individual species, precluding the re-use of the data to reveal more detailed genome features including genome sizes (for plant with large genome size), ploidy level etc., or its direct usage in the further de novo genome assembly."<br><br><br>7. Line 85: A major challenge for diverse genome sequencing projects like this one is choosing whether to sequence samples in accordance to their genome size, or whether to use a 'one size fits all' approach with similar amounts of data for all samples. It would be valuable to justify this approach, and say the range of coverage this amount of data generates (see main comment 1, above).<br>Response: It's really a good question indeed. As you mentioned, it was a major question cum challenge for us to select the best sequencing approach for all the samples. In continuation to our response to the "comment 1", this project is part of 10KP project, and being the starting point of this mega project, we wanted to first evaluate our existing sequencing strategy according to 'one size fits all' approach. On

Powered by Editorial Manager® and ProduXion Manager® from Aries Systems Corporation

an average we generated 60X data per sample.

8. Line 106: The details of DNA extraction are very brief. Was the same DNA extraction protocol used for all species? Were there any modifications?
Response: We first optimized the CTAB DNA extraction protocol and found it suitable for most of the tested species. That's the reason we used the same DNA extraction (Wu and Yang, 2018) method for all the species. The detailed protocol is available via GigaScience protocols.

Wu C and Yang T. DNA Extraction for plant samples by CTAB. GigaScience. 2018;doi:10.17504/protocols.io.pzqdp5w

9. Line 107: BGISEQ is not a very established sequencing platform relative to other platforms (such as Illumina). A brief description of this technology would be useful.
Response: BGISEQ-500 is a desktop sequencer developed by our Institute BGI-Shenzhen in 2015. Using DNA nanoball and combinational probe anchor synthesis developed from Complete Genomics™ sequencing technologies, it generates short reads at a large scale. The sequencing outputs are comparable with the Illumina series (Mak et al 2017), and has been successfully utilized to sequence the plant genome (Chang et al 2018), human genome (Huang et al 2017), and metagenomes (Fang et al 2017)

Mak, S. S. T., Gopalakrishnan, S., Carøe, C., Geng, C., Liu, S., Sinding, M. H. S., ... & Germonpré, M. (2017). Comparative performance of the BGISEQ-500 vs Illumina HiSeq2500 sequencing platforms for palaeogenomic sequencing. GigaScience, 6(8), 1-13.

Huang, J., Liang, X., Xuan, Y., Geng, C., Li, Y., Lu, H., ... & Sun, N. (2017). A reference human genome dataset of the BGISEQ-500 sequencer. Gigascience, 6(5), 1-9.

Fang, C., Zhong, H., Lin, Y., Chen, B., Han, M., Ren, H., ... & Stein, S. (2017). Assessment of the cPAS-based BGISEQ-500 platform for metagenomic sequencing. GigaScience, 7(3), gix133.

10. Line 108: What type of libraries were prepared? What was the insert size?
Response: The 100bp pair end libraries were prepared for the sequencing, and the insert size was 200 bp.

11. Line 110. Repetition of 100bp PE from the previous line.
Response: We have now deleted the repetitive information.

12. Line 166: Define which repeats are being counted.
Response: We didn't assemble all the genomes, and doesn't used the RepeatExplorer program for our analysis. That's why we just used the Kmer data plot to identify the repeat ratio in the sequenced genomes. The specific repeat types will be classified in our subsequent manuscript, once we finish the assembly of all the genomes.

Line 183: It's unclear to me how heterozygosity was scored. Were reads mapped back to the initial assembly then heterozygous sites scored?
Response: The heterozygosity was scored based on the Kmer distribution plot according to the method described by Liu et al (2013). The same description is now mentioned in the main text also.

13. Line 255: Evolution of vasculature is not really relevant to this study.
Figure legends lack detail, e.g. details of the phylogenetic reconstruction used in Figure 2.
Response:
The ordinal phylogeny in Figure 2 was "drop-tips" from Figure 3.

14. Reference 4 seems incomplete.
Response: Thanks for your careful observation. We have now updated the reference

	for the book chapter.<br><br><br>Reviewer #3: This manuscript reports the whole genome sequencing of more than 600 hundred vascular plant samples collected at the Ruili Botanical Garden in China. The data generated here makes an enormous contribution to the study of plant genomes of non-model plants by generating raw sequencing data, along with images, voucher information, complete chloroplast genomes and several partial nuclear genomes assembled. The supplementary data is essential to understand the scope of this project, and make processed data (i.e. genome assemblies) available.<br>My main comments, suggestions and edits are included in the attached word document. They include a few questions to clarify the analyses performed, as well as suggestions to make the language clearer.<br><br>Response:<br>Thank you for your great suggestions and kind recommendation. We have thoroughly updated the manuscript (with track changes) as per your suggestions. Kindly refer the enclosed manuscript.
Additional Information:	
**Question**	**Response**
Are you submitting this manuscript to a special series or article collection?	No
**Experimental design and statistics**<br><br><br>Full details of the experimental design and statistical methods used should be given in the Methods section, as detailed in our Minimum Standards Reporting Checklist. Information essential to interpreting the data presented should be made available in the figure legends.<br><br><br>Have you included all the information requested in your manuscript?	Yes
**Resources**<br><br><br>A description of all resources used, including antibodies, cell lines, animals and software tools, with enough information to allow them to be uniquely identified, should be included in the Methods section. Authors are strongly encouraged to cite Research Resource Identifiers (RRIDs) for antibodies, model organisms and tools, where possible.<br><br><br>Have you included the information requested as detailed in our Minimum	Yes

Standards Reporting Checklist?	
**Availability of data and materials**<br><br>All datasets and code on which the conclusions of the paper rely must be either included in your submission or deposited in publicly available repositories (where available and ethically appropriate), referencing such data using a unique identifier in the references and in the "Availability of Data and Materials" section of your manuscript.<br><br>Have you have met the above requirement as detailed in our Minimum Standards Reporting Checklist?	Yes

Manuscript clean
Click here to access/download;Manuscript;GIGA-D-18-00121_clean.docx
Click here to view linked References

Molecular Digitization of a Botanical Garden: High-depth whole genome sequencing of 689 vascular plants from the Ruili Botanical Garden

Authors

Huan Liu[1*], Jinpu Wei[2*], Ting Yang[1*], Weixue Mu[1], Bo Song[1], Tuo Yang[2], Yuan Fu[1], Xuebing Wang[2], Guohai Hu[2], Wangsheng Li[2], Hongcheng Zhou[2], Yue Chang[1], Xiaoli Chen[1], Hongyun Chen[1], Le Cheng[3], Xuefei He[2], Hechen Cai[2], Xianchu Cai[2], Mei Wang[1], Yang Li[2], Sunil Kumar Sahu[1], Jinlong Yang[3], Yu Wang[3], Ranchang Mu[4], Jie Liu[4], Jianming Zhao[4], Ziheng Huang[1], Xun Xu[1], Xin Liu[1#].

Author Affiliations

1. BGI-Shenzhen, Shenzhen 518083, China
2. China National GeneBank, BGI-Shenzhen, Shenzhen 518120, China
3. BGI-Yunnan, BGI-Shenzhen, Kunming, 650106, China
4. Forestry Bureau of Ruili, Ruili, 678600, China

[*] These authors contributed equally to this work.

[#] To whom correspondence should be addressed: Xin Liu (liuxin@genomics.cn)

Abstract

Background

Genome sequencing has been widely used in plant research to construct reference genomes and elucidate evolutionary insights. However, only a limited number of plant species have had their whole genome sequenced, and the limited taxon information of these species has further restrained the utility of these data.

Findings

Here, we comprehensively sampled and sequenced vascular plant species of growing in the Ruili Botanical Garden, located in South West China. We sequenced 761 samples out of the total 1,093 collected voucher specimens stored in the Herbarium of China National GeneBank (HCNGB). These 761 samples represented 689 vascular plant species from 134 families belonging to 47 orders. Of these, 254 samples were identified to species level and 506 samples were identified to families using chloroplast sequences. In total, we generated 54 Tb of sequencing data, which resulted in an average sequencing depth of 60X per species, as estimated by the genome size. A reference phylogeny was reconstructed with 78 chloroplast genes for molecular identification and other possible applications.

Conclusions

In this study, we generated a large dataset of vascular plant genomes, with both high-depth whole genome sequencing data and associated voucher specimens, making it a valuable dataset for plant genome research and other applications. This project

also provides insight into the feasibility and technical requirements for “planetary scale” projects such as the 10 thousand Plant Genome Project (10KP) and the Earth BioGenome Project (EBP).

Keywords: Whole genome sequencing, Vascular plants, Phylogeny, Voucher specimens, Ruili Botanical Garden.

Background

With the advent of next generation sequencing technologies, enormous efforts have been made to sequence whole genomes of plant species, thereby providing new insights on plant evolution [1] and new information for improving agriculture yield and stress tolerance [2, 3]. As of September 2018, more than 350 land plant’s genome has been sequenced (https://www.ncbi.nlm.nih.gov/genome), most of which are crops (57.7%), model species along with their closely-related species (22.3%), and wild relatives of crops (17.7%). However, with approximately 391,000 known species of plants [4], if we consider the evolutionary history and diversity of plants, the currently available sequencing data is very limited. More recently, more than 1,000 plant species have been sequenced at the transcriptome to reveal the evolution of plants, and thus also provide valuable resources for other plant research [5]. However, considering the enormous gap areas outside of the coding regions, whole genome sequencing data should be generated for further plant evolution studies. Thus, global

efforts have been initiated to sequence 10,000 plant genomes (10KP) as a key part of the Earth BioGenome Project (EBP)[6]. For these large-scale whole genome sequencing efforts, we need to prove the feasibility as well as to set up technical standards for sampling, sequencing and data management.

Over the past decade, DNA barcoding has emerged as an important molecular tool for ecological studies, and specially for the rapid identification of non-routine specimens [7]. Although it is well-suited for studying historical specimen samples, considering the DNA degradation in those samples [8, 9], the major drawback of the technology is that DNA barcoding only provides limited genomic information, which is just based on the small fragments of the nuclear or chloroplast genome [10]. In order to overcome this problem, genome skimming, which is whole genome sequencing by second-generation sequencing technologies, has been proposed [11] to provide more genome sequence information for better species identification [12, 13]. However, previous genome skimming studies have only generated a small amount of sequencing data for the individual species, precluding the re-use of the data to reveal more detailed genome features including genome sizes (for a plant with large genome size), ploidy level etc., or its direct usage in the further de novo genome assembly. Here, we sequenced vascular plants genomes of 761 samples representing 689 vascular plant species at high depth (more than 60Gb on an average per sample). Making all of these data freely accessible and linked to their voucher details in the CNGB herbarium and Ruili Botanical Garden will provide new insights into the

evolution of vascular plants and enable it to be utilized as a valuable genomic resource for evolution and diversity research and applications.

Data Description

Sampling, sequencing and data summary

In order to investigate the diversity of vascular plants in Ruili Botanical Garden and provide genome information for these species, we sampled almost all the vascular plant species in Ruili Garden and sequenced them using BGISEQ-500 sequencing technology. These samples were collected from Ruili Botanical Garden, Yunnan, China (97°38′47″ to 98°05′57″ N, 23°52′42″ to 24°09′20″ E, ranging in altitude from 738 m to 1,200 m above the sea level, as shown in Figure 1). In total, we collected 1,093 vascular plant samples, from which we used the young leaves for DNA extraction. Voucher specimens and images were also collected for these samples. All the specimens are stored in the Herbarium, of China National GeneBank (HCNGB), and voucher information can be found in Table S1 (Additional files). The collected young leaves were shipped to Shenzhen on dry ice, and DNA was extracted using the CTAB method [14]. We were successful at extracting enough DNA for 761 of those samples. whole genome sequencing libraries were constructed for each of these samples according to BGISEQ-500 manufacturer instructions [15], and then sequenced [16]. Approximately 70 Gb of raw sequencing data (100 bp, paired-end) was generated for each of these samples (Table 1). Raw reads were filtered using SOAPfilter_v2.2 by following the command: -y –p –i 180 –M 2 –Q 10. After filtering

the low-quality reads (reads with more than 10% Ns, ambiguous bases; reads with more than 40% bases having quality lower than 10; reads contaminated by adaptors or PCR duplicates), ~60 Gb clean data were obtained for each of these samples and data showed high-quality reads (>Q35).

Species identification and phylogenetic relationship

The taxonomic identification of specimens is a time-consuming process and requires expertise and experience. In this study, the collections covering the majority of vascular plant lineages were difficult to identify to species level in a short time. We were able to identify 254 samples to species level (from a total of 232) using the specimen morphology and the other 506 samples were identified to families using their chloroplast sequences. Thus, in total, we identified 689 samples from those 761 sequenced, which belonged to 134 families and 47 orders. Among these families, the majority of the species belonged to Fabaceae (71 taxa), Poaceae (45 taxa) and Asteraceae (38 taxa), respectively. We assembled the chloroplast genomes of each species from clean read data using NOVOPlasty[17], a seed-extension-based de novo assembler. We used the complete cds rbcL gene sequence of *Arabidopsis thaliana* (downloaded from NCBI, accession number: U91966) as the seed to conduct the assembly. The NOVOPlasty assembly recovered complete chloroplast genomes of 50 species in a single circular sequence. For the remaining species, the longest contig assembled by NOVOPlasty was BLASTed against the chloroplast database (downloaded from NCBI, including 2,503 non-redundant species) and the resulted

best-hit sequences (minimum requirement: e-value < 10-7 and identity > 95%) were used as references for further assembly using MITObim[18], in this way, we finally recovered complete chloroplast genomes for all 689 species. The assembled chloroplast genomes ranged from 113,621 to 183,602 bp in size (Table S2). We then annotated the assembled chloroplast genomes using DOGMA [19] and GeneWise [20], and we found 72 protein-coding genes in almost all of these vascular plant families except Gnetaceae, Malvaceae, Elaeocarpaceae, and Tectariaceae. For Gnetaceae, we were only able to annotate 52 protein-coding genes in their chloroplast genomes which is consistent with previous studies [21]. We then compared these assembled chloroplast genomes and constructed the phylogenetic tree using RAxML[22] and IQ_TREE[23]. A total of 78 individual coding genes were identified from 738 samples, the majority of them were present in 710 to 738 (on average). However, only 18 genes were commonly found in all of the samples studied. Each gene was aligned using MAFFT[24] and every alignment was then processed with TrimAL[25] using the gappyout option to remove poorly aligned positions. Then gene alignments were combined which resulted in 46235 nucleotide positions. Maximum likelihood (ML) species trees were constructed by RAxML package (v8.2.4) with GTRCAT model, 1,000 bootstrap replicates, 5 random seed number were selected for the parsimony inferences and 26 fern samples were used to root the tree. At the same time, ML analyses were performed with IQ-TREE under the substitution model GTR+F+R10 which was determined according to the Akaike information criterion (AIC) and the

Bayesian information criterion (BIC) by IQ-TREE. Both RAxML and IQ_TREE provided concordant phylogenetic reconstruction (Figure 3 and Figure S2). The major lineages can be observed within Fabales, Rosales, Poales and Malpighiales. In Fabids, Celastrales was the sister group to Malpighiales other than Oxalidales in this study (BS=100%). For Petrosaviidae, the major ordinal relationship was consistent with the previous research, as well as for Liliales, Asparagales, Poales, Arecales, Commelinales, Pandanales, Zingiberales in the same clade, the earliest-branching lineage is Alismatales [26]. Relationships between Gentianales, Lamiales and Solanales remained unclear [27, 28], ML tree provided support for Gentianales sister to Lamiales (BS=83%) with sister group to Solanales and Boraginales (BS=100%). We also included 54 species of Poales in the phylogenetic tree which revealed its close relationship with Arecales rather than Pandanales and Dioscoreales.

Genome size, repeat content, and heterozygosity

In order to ensure the quality and effectiveness of the dataset (Table 1), we conducted several analyses to reveal the basic genomic features of the vascular plants sampled. By using GCE [29] and kmergenie [30] software and the clean data of each species, we estimated the genome sizes, repeat content and heterozygosity (Figure 2 and Table S1). For several of these tested species, the genome sizes have been previously measured by experimental approaches and are publicly available (http://data.kew.org/cvalues/) (Table S3). We compared the previous estimations to the genome sizes estimated by k-mer analysis in this study, and found good agreement

between them (R2=0.63) (Figure S1). We found that despite overall wide variation in the genome sizes of these plants, most of the families had relatively comparable genome sizes. The most diversed family in terms of genome size was found to be Cupressaceae, in which genome sizes ranging from 0.18 Gb in *Cunninghamia lanceolata* (Lamb.) Hook. var. *lanceolata* to 19.26 Gb in Juniperus pingii var. wilsonii (Rehder) Silba. In addition, repeat content varied from 10% to 88% on average among species sampled, with several exceptions in Cornaceae, Myrtaceae and Celastraceae. For instance, Myrtaceae(Myrtales) was found to have the most repetitive genomes (~88% of repetitive content), while Celastraceae (Celastrales) was found to have the least repetitive genomes (~10% repetitive content). We also found relatively high heterozygosity in these species ranging from 0.15% to 36.6% individually, which probably reflected their nature as wild species.

Genome assemblies

Despite the limitation of having only one sequencing library constructed for each species, we were able to conduct preliminary genome assemblies for many of these species, which reflected the quality and reuse potential of the data. Based on the estimated heterozygosity and repeat content, we initially selected 17 species from 17 families with relatively simple genome content (heterozygosity rate less than 1% and repeat content less than 50%) for genome assembly. We used SOAPdenovo2 [31] (parameters: pregraph-K 35 contig -M 1 scaff). We obtained an average contig N50 of 4.62 kb, and an average scaffold N50 of 32.2 kb for these genome assemblies. Two

species *Alternanthera sessilis* (L.) R.Br. ex DC. and *Senna alata* (L.) Roxb., were assembled to contig N50 of 15.2 kb, scaffold N50 of 95.5 kb and contig N50 of 14 kb and the scaffold N50 of 101.1 kb respectively (Table S4). We then carried out Benchmarking Universal Single-Copy Orthologs (BUSCO)(version 3.0.1) analysis [32] to find the completeness of all these 17 genome assemblies. On average, genome completeness was found to be ~89.1%, 1243 BUSCOs were complete and single-copy and 40 BUSCOs were complete and duplicated (from a total of 1440 BUSCOs). The average number of fragmented and missing BUSCOs were 55 and 101, respectively (Table S5). Our preliminary assemblies were of good quality, providing a useful reference for future efforts to establish complete reference genomes for all these plant species. In addition to the current attempt of genome assembly, continuing efforts are being carried out to finish the preliminary assemblies of the other species and these are being deposited and linked with existing already public sequencing data.

Data access and reuse potential

The data generated here includes images, raw sequencing data, assembled chloroplast genomes and preliminary nuclear genome assemblies. All the data have been organized and linked to a top-level accession in the GigaScience GigaDB repository (http://doi.org/10.5524/100502), containing the lists of all the species and the links to each species page. In addition, each species has a DOI assigned to them containing information on collection number, an image of the plant during sampling, SRA accession number for the raw data, a data file containing the assembled chloroplast

sequence (these chloroplast sequences can also be found in Table S2), a data file containing the preliminary assembled genome sequence (available only for some species and will continue to be updated when each assembly is completed). Voucher specimens are stored in the Herbarium of China National GeneBank (HCNGB), and digitized images for every sheet are also being made available in GigaDB alongside the sequencing data. All raw data are stored in the NCBI SRA repository under the project number PRJNA43840. In addition to the description in SRA, the SRA accession number of raw data is also included in the GigaDB entries, thus the raw data of specific species can be traced from GigaDB. Datacite and GigaDB (http://doi.org/10.5524/100502) metadata are all linked, and any future updates made on the GigaDB dataset provides traceable records.

The high-depth whole genome sequencing data together with images and voucher specimens can be reused in different ways and will be valuable for future applications. First of all, in addition to the phylogenetic analysis carried out here using on the assembled chloroplast genomes, future evolutionary analysis can be carried out to study the evolution of specific genes after assembling them from raw reads, as well as investigating particular features of plant genome evolution including evolution of repeats, polyploidization, whole genome duplication, etc. Secondly, the data can be used to improve future genome assemblies of these plant species. For example, utilizing the information on repeat content, heterozygosity and genome size estimation provided here to tailor new sequencing and genome assembly strategies of

these plant genomes, as well as integrating the sequencing data itself in other genome assemblies. By directly using the sequencing data obtained from this study, it would be easier and more efficient to assemble the remaining sequenced plant genomes. The ~70 Gb data can be used for genome assembly in combination with either contig reconstruction of the second generation based sequence reads, or for error correction of the third generation long sequence reads. Last but not least, this dataset can also be used for developing new methods for species identification either based on sequencing data or based on images of plants and to resolve phylogenetic relationships based on whole genome sequencing data among others. For example, deep learning can be applied to develop plant identification using this dataset as a good training set. Providing this comprehensive dataset which can be easily accessed by researchers and also the general public, we beleive it would be reused in many ways beyond what has been mentioned here.

Discussion

The current understanding on the evolution of plants and its diversity in a phylogenomic context is limited due to the non-availability of genome-scale information across phylogenetically diverse species [33]. In this study, we provide a dataset of high-depth whole genome sequencing of 689 vascular plant species with voucher specimens, covering 134 families and 47 orders. These samples were obtained from Ruili Botanical Garden in Yunnan Province of China, near the border between China and Myanmar, reflecting the rich plant diversity in that region. The

high-depth whole genome sequencing data generated here have been used to estimate genomic features including genome size, repeat content, and heterozygosity, which can provide guidance to the future studies aiming at establishing reference genomes for these species. The high-depth whole genome data can be also used in assembling chloroplast genomes, as well as some conserved nuclear genes, thus providing useful information for evolution and gene function studies.

In this study, we scaled up the plant whole genome sequencing effort to sequence hundreds of plant species. We only constructed a single short insert library (200 bp) for each of the species and generated ~60 Gb of whole genome sequencing data. It would be insufficient to assemble good draft genomes for the majority of the species just based on single library data, because previous efforts to assemble reference genomes based on second generation sequencing data have required multiple short insert libraries and also mate pair (large insert size) libraries. In addition, our study tested for the first time, the feasibility of large-scale whole genome sequencing, which is already underway for the Earth BioGenome Project (EBP) [6] and 10 thousand Plant Genome Projects (10KP) [34]. This study provided experiences for plant sampling, sample logistics and management, DNA extraction, sequencing library preparation, sequencing and data analysis and management. Aiming at sequencing more than 10,000 plant species, 10KP would require to establish a robust infrastructure for sample and data management, as potentially investigated in this pilot study.

Availability of Supporting Data

The specimens, leaf samples and DNA solutions of all collections are stored at the China National GeneBank (CNGB) Herbarium. The raw sequencing data described in this article are available in the NCBI SRA repository, under the project number PRJNA43840. DNA Extraction [27] and BGISEQ-500 WGS library construction protocols can be found in protocols.io [28]. A total of 738 chloroplast genomes and 17 assembled genomes together with raw data supporting the results of this article are available via the GigaDB repository of GigaScience, and will be continuously updated and linked to the GigaDB entries as new assemblies are completed.

Additional files

Additional file 1

Table S1. List of samples included in this study with voucher information, current kmer based estimation of genome sizes, repeat content and heterozygosity. Identified collections were listed with species names, while unidentified ones with only family and order information. 738 samples with assembled chloroplast genome were marked with *, whereas 17 samples with assembled genomes were marked with §.

Table S2. All the assembled chloroplast genomes and their lengths.

Table S3. Genome information previously measured and publicly available on the database.

Table S4. Summary of preliminary genome assemblies of 17 species of vascular plants families.

Table S5. Summary of BUSCO analysis for 17 species of vascular plants families.

Additional file 2

Figure S1. A comparison of genome sizes measured by the experimental approaches to the k-mer estimated genome sizes in this study.

Figure S2. Phylogeny of vascular plants of the Ruili Botanical Garden. The tree shows the species tree based on 78 chloroplast genes by RAxML. The inner circle and the outer circus colors represent different families and orders. The clade color represents bootstrap values from red to gray (bootstrap range from 50 to 100).

Abbreviations

10 KP: 10 thousand Plant Genome Project

bp: base pair

BUSCO: Benchmarking Universal Single-Copy Orthologs.

EBP: Earth BioGenome Project.

Gb: Gigabase pair

HCNGB: Herbarium, China National GeneBank.

ML: Maximum likelihood.

WGS: Whole Genome Sequencing.

Competing interests

All authors declare that they have no competing interests.

Funding

This work was supported by grants of Basic Research Program, the Shenzhen Municipal Government, China (No.JCYJ20150529150505656) and (No.JCYJ20150831201643396), as well as funding from State Key Laboratory of Agricultural Genomics (No.2011DQ782025), Guangdong Provincial Key Laboratory of Genome Read and Write (No.2017B030301011) , The Construction of China National GeneBank (Yunnan GeneBank) (Yunnan province, 2015DA008, P.R. China)

Author contributions

XL conceived this study. XL and HL drafted the manuscript. HL managed the project. JPW, XBW, LC, XFH, HCC, JLY, YW, RCM, JL, JMZ collected the samples. TY lead identification of voucher specimens. TY, WXM, BS, YF, YC, HYC analyzed the data. TY, XLC, MW, ZHH constructed the phylogenetic tree. GHH, WSL, HCZ, HCC, YL extracted DNA and performed genome sequencing. SKS and XX revised and edited the manuscript. All the authors have read and approved the final manuscript.

Acknowledgments

The authors would like to express their sincere thanks to the local people and

Government of Yunnan province, and Forestry Institute of Dehong Prefecture for their kind help in sample collections. We would also like to thank the taxonomic experts in PE (Herbarium, Institute of Botany, Chinese Academy of Sciences) for identification. Finally, we are thankful to the production team of China National GeneBank, Shenzhen, China.

References

1. Pennisi E. Plant biology. Green genomes. Science. 2011;332 6036:1372-5. doi:10.1126/science.332.6036.1372.
2. Bolger ME, Weisshaar B, Scholz U, Stein N, Usadel B and Mayer KF. Plant genome sequencing - applications for crop improvement. Curr Opin Biotechnol. 2014;26:31-7. doi:10.1016/j.copbio.2013.08.019.
3. Desta ZA and Ortiz R. Genomic selection: genome-wide prediction in plant improvement. Trends Plant Sci. 2014;19 9:592-601. doi:10.1016/j.tplants.2014.05.006.
4. Kew RBG. The state of the world's plants report–2016. Royal Botanic Gardens, Kew. 2016.
5. Matasci N, Hung L-H, Yan Z, Carpenter EJ, Wickett NJ, Mirarab S, et al. Data access for the 1,000 Plants (1KP) project. Gigascience. 2014;3 1:17.
6. Lewin HA, Robinson GE, Kress WJ, Baker WJ, Coddington J, Crandall KA, et al. Earth BioGenome Project: Sequencing life for the future of life. Proc Natl Acad Sci U S A. 2018;115 17:4325-33.
7. de Vere N, Rich TC, Trinder SA and Long C. DNA barcoding for plants. Methods Mol Biol. 2015;1245:101-18. doi:10.1007/978-1-4939-1966-6_8.
8. Staats M, Erkens RH, van de Vossenberg B, Wieringa JJ, Kraaijeveld K, Stielow B, et al. Genomic treasure troves: complete genome sequencing of herbarium and insect museum specimens. PLoS One. 2013;8 7:e69189. doi:10.1371/journal.pone.0069189.
9. Osmundson TW, Robert VA, Schoch CL, Baker LJ, Smith A, Robich G, et al. Filling gaps in biodiversity knowledge for macrofungi: contributions and

assessment of an herbarium collection DNA barcode sequencing project. PLoS One. 2013;8 4:e62419. doi:10.1371/journal.pone.0062419.

10. Li X, Yang Y, Henry RJ, Rossetto M, Wang Y and Chen S. Plant DNA barcoding: from gene to genome. Biol Rev Camb Philos Soc. 2015;90 1:157-66. doi:10.1111/brv.12104.

11. Straub SC, Parks M, Weitemier K, Fishbein M, Cronn RC and Liston A. Navigating the tip of the genomic iceberg: Next-generation sequencing for plant systematics. Am J Bot. 2012;99 2:349-64. doi:10.3732/ajb.1100335.

12. Male PJ, Bardon L, Besnard G, Coissac E, Delsuc F, Engel J, et al. Genome skimming by shotgun sequencing helps resolve the phylogeny of a pantropical tree family. Mol Ecol Resour. 2014;14 5:966-75. doi:10.1111/1755-0998.12246.

13. Besnard G, Christin PA, Male PJ, Coissac E, Ralimanana H and Vorontsova MS. Phylogenomics and taxonomy of Lecomtelleae (Poaceae), an isolated panicoid lineage from Madagascar. Ann Bot. 2013;112 6:1057-66. doi:10.1093/aob/mct174.

14. Wu C and Yang T. DNA Extraction for plant samples by CTAB. Gigascience. 2018; doi:10.17504/protocols.io.pzqdp5w.

15. Gao S, Mu F, Yang Z, Liu X, Jiang H, Liao S, et al. BGISEQ-500 WGS library construction. 2018; doi:10.17504/protocols.io.ps5dng6.

16. Gao S, Mu F, Yang Z, Liu X, Jiang H, Liao S, et al. BGISEQ-500 Sequencing. doi:10.17504/protocols.io.pq7dmzn.

17. Dierckxsens N, Mardulyn P and Smits G. NOVOPlasty: de novo assembly of organelle genomes from whole genome data. Nucleic acids research. 2016;45 4:e18-e.

18. Hahn C, Bachmann L and Chevreux B. Reconstructing mitochondrial genomes directly from genomic next-generation sequencing reads—a baiting and iterative mapping approach. Nucleic acids research. 2013;41 13:e129-e.

19. Wyman SK, Jansen RK and Boore JL. Automatic annotation of organellar genomes with DOGMA. Bioinformatics. 2004;20 17:3252-5. doi:10.1093/bioinformatics/bth352.

20. Birney E, Clamp M and Durbin R. GeneWise and Genomewise. Genome Res.

2004;14 5:988-95. doi:10.1101/gr.1865504.

21. Hsu CY, Wu CS, Surveswaran S and Chaw SM. The complete plastome sequence of Gnetum ula (Gnetales: Gnetaceae). Mitochondrial DNA A DNA Mapp Seq Anal. 2016;27 5:3721-2. doi:10.3109/19401736.2015.1079874.

22. Stamatakis A. RAxML version 8: a tool for phylogenetic analysis and post-analysis of large phylogenies. Bioinformatics. 2014;30 9:1312-3.

23. Nguyen L-T, Schmidt HA, von Haeseler A and Minh BQ. IQ-TREE: a fast and effective stochastic algorithm for estimating maximum-likelihood phylogenies. Molecular biology and evolution. 2014;32 1:268-74.

24. Katoh K, Misawa K, Kuma K and Miyata T. MAFFT: a novel method for rapid multiple sequence alignment based on fast Fourier transform. Nucleic Acids Res. 2002;30 14:3059-66.

25. Capella-Gutiérrez S, Silla-Martínez JM and Gabaldón T. trimAl: a tool for automated alignment trimming in large-scale phylogenetic analyses. Bioinformatics. 2009;25 15:1972-3.

26. Chase MW. Monocot relationships: an overview. Am J Bot. 2004;91 10:1645-55. doi:10.3732/ajb.91.10.1645.

27. Bremer K, Backlund A, Sennblad B, Swenson U, Andreasen K, Hjertson M, et al. A phylogenetic analysis of 100+ genera and 50+ families of euasterids based on morphological and molecular data with notes on possible higher level morphological synapomorphies. Plant Systematics and Evolution. 2001;229 3-4:137-69.

28. Refulio- Rodriguez NF and Olmstead RG. Phylogeny of lamiidae. American Journal of Botany. 2014;101 2:287-99.

29. Liu B SY, Yuan J, Hu X, Zhang H, Li N, Li Z, Chen Y, Mu D, Fan W. Estimation of genomic characteristics by analyzing k-mer frequency in de novo genome projects. arXiv preprint. 2013; doi:arXiv:1308.2012.

30. Chikhi R and Medvedev P. Informed and automated k-mer size selection for genome assembly. Bioinformatics. 2014;30 1:31-7. doi:10.1093/bioinformatics/btt310.

31. Luo R, Liu B, Xie Y, Li Z, Huang W, Yuan J, et al. SOAPdenovo2: an empirically improved memory-efficient short-read de novo assembler.

Gigascience. 2012;1 1:18.

32. Simão FA, Waterhouse RM, Ioannidis P, Kriventseva EV and Zdobnov EM. BUSCO: assessing genome assembly and annotation completeness with single-copy orthologs. Bioinformatics. 2015;31 19:3210-2.

33. Cheng S, Melkonian M, Smith SA, Brockington S, Archibald JM, Delaux P-M, et al. 10KP: A phylodiverse genome sequencing plan. Gigascience. 2018;7 3:giy013.

34. Cheng S, Melkonian M, Smith SA, Brockington S, Archibald JM, Delaux P-M, et al. 10KP: A Phylodiverse Genome Sequencing Plan. GigaScience. 2018.

Figure legends

Figure 1. Sampling localities of this project. Sampling was conducted mainly in Ruili Botanical Garden in Southwest China, near the China-Myanmar border, and shown in red rectangles.

Figure 2. The ordinal phylogeny is based on “drop-tips” from Figure 3. Genome size, repeat content and heterozygosity statistics of the nuclear genomes assembled in this study. (a) Genome sizes in GB, (b) repeat content as percentage of total genome (%), (c) heterozygosity ratio Cladogram was generated from the 78 chloroplast gene phylogeny.

Figure 3. Phylogeny of vascular plants of the Ruili Botanical Garden. The tree shows the coalescent tree based on 78 chloroplast genes. In inner circus, Colors represent different families and out circus colors represent different orders.

Tables

Table 1 Summary of the sequencing data in this study.

Manuscript with track changes

Click here to access/download;Manuscript;GIGA-D-18-00121_with track changes.docx

Click here to view linked References

GigaScience

Digitizing a Botanical Garden: High-depth whole genome sequencing of 689 vascular plants from Ruili Garden

--Manuscript Draft--

Manuscript Number:	GIGA-D-18-00121
Full Title:	Digitizing a Botanical Garden: High-depth whole genome sequencing of 689 vascular plants from Ruili Garden
Article Type:	Data Note
Abstract:	Background<br>Genome sequencing has been widely used in plant research to construct reference genomes and elucidate evolutionary insights. However, only a limited number of plant species have had their whole genome sequenced, and the limited taxon information of these species has further restrained the utility of these data.<br>Findings<br>Here, we comprehensively sampled and sequenced vascular plant species of Ruili Botanical Garden, located in South West China. We sequenced 760 samples out of the total 1,093 collected voucher specimens stored in the Herbarium of China National GeneBank (HCNGB). These 760 samples represented 689 vascular plant species from 134 families belonging to 47 orders. Of these, 254 samples were identified to 232 species by specimen and 506 samples can be identified to families by chloroplast sequences. We generated 54 Tb sequencing data in total, which resulted in an average sequencing depth of 60×for these species, as estimated by the genome size. A reference phylogeny was reconstructed with 78 chloroplast genes for molecular identification and possible applications.<br>Conclusions<br>In this study, we established a large dataset of vascular plants' genomes, with both the high-depth whole genome sequencing data and the voucher specimens, making it valuable dataset for plant genome researches and applications. And providing insight into the feasibility and technical requirements for "planetary scale" projects such as the 10 thousand Plant Genome Project (10KP) and Earth BioGenome Project (EBP).
Additional Information:	
Question	Response
Are you submitting this manuscript to a special series or article collection?	No
Experimental design and statistics<br><br>Full details of the experimental design and statistical methods used should be given in the Methods section, as detailed in our Minimum Standards Reporting Checklist. Information essential to interpreting the data presented should be made available in the figure legends.	Yes

Have you included all the information requested in your manuscript?	
Resources	Yes

Powered by Editorial Manager® and ProduXion Manager® from Aries Systems Corporation

A description of all resources used, including antibodies, cell lines, animals and software tools, with enough information to allow them to be uniquely identified, should be included in the Methods section. Authors are strongly encouraged to cite Research Resource Identifiers (RRIDs) for antibodies, model organisms and tools, where possible.<br><br>Have you included the information requested as detailed in our Minimum Standards Reporting Checklist?	
Availability of data and materials<br><br>All datasets and code on which the conclusions of the paper rely must be either included in your submission or deposited in publicly available repositories (where available and ethically appropriate), referencing such data using a unique identifier in the references and in the “Availability of Data and Materials” section of your manuscript.<br><br>Have you have met the above requirement as detailed in our Minimum Standards Reporting Checklist?	Yes

Powered by Editorial Manager® and ProduXion Manager® from Aries Systems Corporation

Manuscript

Click here to view linked References

Click here to access/download;Manuscript;Plants_Manuscript_-8.8.docx

Molecular Digitization of a Botanical Garden: High-depth whole genome sequencing of 689 vascular plants from the Ruili Botanical Garden

Authors

Huan Liu[1*], Jinpu Wei[2*], Ting Yang[1*], Weixue Mu[1], Bo Song[1], Tuo Yang[2], Yuan Fu[1], Xuebing Wang[2], Guohai Hu[2], Wangsheng Li[2], Hongcheng Zhou[2], Yue Chang[1], Xiaoli Chen[1], Hongyun Chen[1], Le Cheng[3], Xuefei He[2], Hechen Cai[2], Xianchu Cai[2], Mei Wang[1], Yang Li[2], Sunil Kumar Sahu[1], Jinlong Yang[3], Yu Wang[3], Ranchang Mu[4], Jie Liu[4], Jianming Zhao[4], Ziheng Huang[1], Xun Xu[1], Xin Liu[1#].

Formatted: Superscript

Author Affiliations

1. BGI-Shenzhen, Shenzhen 518083, China
2. China National GeneBank, BGI-Shenzhen, Shenzhen 518120, China
3. BGI-Yunnan, BGI-Shenzhen, Kunming, 650106, China
4. Forestry Bureau of Ruili, Ruili, 678600, China

[*] These authors contributed equally to this work.

[#] To whom correspondence should be addressed: Xin Liu (liuxin@genomics.cn)

Abstract

Background

Genome sequencing has been widely used in plant research to construct reference genomes and elucidate evolutionary insights. However, only a limited number of plant species have had their whole genome sequenced, and the limited taxon information of these species has further restrained the utility of these data.

Findings

Here, we comprehensively sampled and sequenced vascular plant species growing in theof Ruili Botanical Garden, located in South West China. We sequenced 761 -samples out of the total 1,093 collected voucher specimens stored in the Herbarium of China National GeneBank (HCNGB). These 761 samples represented 689 vascular plant species from 134 families belonging to 47 orders. Of these, 254 samples were identified to 232 species level by specimen and 506 samples can bewere identified to families by using chloroplast sequences. In total, wWe generated 54-Tb of sequencing data, in total, which resulted in an average sequencing depth of 60× for theseper species, as estimated by the genome size. A reference phylogeny was reconstructed with 78 chloroplast

Conclusions

In this study, we established generated a large dataset of vascular plants' genomes, with

Commented [Office1]: What do you mean by this? I believe the taxa sequenced are well-known, do you mean there are no voucher specimens associated? Or something else? Please clarify.

Commented [SS2R1]: Here we are not talking about our sequenced species. Here the "limited taxon information " refers to species whose genome information is not available. This is just a background information.

genes for molecular identification and other possible applications.

both the high-depth whole genome sequencing data and the associated voucher specimens, making it a valuable dataset for plant genome researches and other applications. And This project also providesing insight into the feasibility and technical requirements for “planetary scale” projects such as the 10 thousand Plant Genome Project (10KP) and the Earth BioGenome Project (EBP).

Keywords: Whole genome sequencing, Vascular plants, Phylogeny, Voucher specimens, Ruili Botanical Garden.

Background

With the advent of next generation sequencing technologies, enormous efforts have been made to sequence whole genomes of plant species, thereby providing new insights on the plant evolution of plants [1] and new informations for improving agriculture yield and stress tolerance [2, 3]. As of September 2018, more than 350 land plant’s genomes are have been sequenced (https://www.ncbi.nlm.nih.gov/genome), most of which are crops (57.7%), model species along with their closely-related species (22.3%), and wild relatives of crops (17.7%) [4]. However, with approximately 391,000 known species of plants [5], if we consider the evolutionary history and diversity of

However, considering the enormous plants, the currently available sequencing data is very limited. More recently, more than 1,000 plant species have been sequenced at the transcriptome to reveal the evolution of plants, and thus also provide valuable resources for other plant research [6].

gap areas outside of the coding regions, whole genome sequencing data should be generated for further plant evolution studies. Thus, global efforts have been initiated to sequence 10,000 plant genomes (10KP) as a key part of the Earth BioGenome Project (EBP)[7]. For these large-scale whole genome sequencing efforts, we need to prove the feasibility as well as to set up technical ~~routines~~ standards for sampling, sequencing and data management. Over the past decade, DNA barcoding has emerged as an important molecular tool for ~~botanical~~ ecological studies, and specially for the rapid identification of non-routine specimens [8]. Although it is well-suited for studying historical specimen samples, considering the DNA degradation in those samples [9, 10], the major drawback of the technology is that DNA barcoding only provides limited genomic information, which is just based on ~~the~~ small fragments of the nuclear or chloroplast genome [11]. In order to overcome this problem, genome skimming, which is whole genome sequencing by second-generation sequencing technologies, has been proposed [12] to provide more genome sequence information for better species identification [13, 14]. However, previous genome skimming studies have only generated a small amount of sequencing data for the individual species, precluding the re-use of the data to reveal more detailed genome features including genome sizes (for plant with large genome size), ploidy level etc., or

Commented [Office3]: Perhaps “ecological” would be a better term. In Botany, DNA barcodes are rarely used.

Commented [SS4R3]: Ok we agree “ecological” is a better term

its direct usage in the further *de novo* genome assembly. Here, we sequenced vascular plants genomes of 761 samples representing 689 vascular plant species at high depth (more than 60GBb on an average per sample). Making

all of these data freely accessible and linked to their voucher details in the CNGB herbarium and Ruili B~~b~~otanical ~~g~~Garden will provide new insights into the evolution of vascular plants and enable it to be utilized as a valuable genomic resource for evolution and diversity research and applications.

Data Description

Sampling, sequencing and data summary

In order to investigate the diversity of vascular plants in Ruili Botanical Garden and provide genome information for ~~those vascular~~these species~~ for possible conservation~~, we sampled ~~the ~~almost all the vascular plant species in Ruili Garden and sequenced them using BGISEQ-500 sequencing technology. These samples were collected from Ruili Botanical Garden, Yunnan, China (97°38′47″ to 98°05′57″ N, 23°52′42″ to 24°09′20″ E, ranging in altitude~~s~~ ~~ranging ~~from 738 m to 1,200 m above the sea level, as shown in Figure 1). In total, we collected 1,093 vascular plant samples, from which we used the young leaves for DNA extraction. Voucher specimens and images were also collected for these samples~~ accordingly~~. All the specimens are stored in the Herbarium~~,~~ of China National GeneBank (HCNGB), and ~~the ~~voucher information can be found in Table S1 (Additional files). The collected young leaves were shipped to

Commented [Office5]: “almost” or “all”?

Commented [SS6R5]: We tried to cover the maxium species available in Ruili garden

Shenzh and ~~then subjected to~~ DNA was extractedion using the CTAB method[15].

en on ~~Finally,~~ WWwe were successsful ateded ~~in~~ extracting enough DNA for 761 of those

dry ice,

samples.. ~~With the extracted DNA, w~~Whole genome sequencing libraries were

constructed for each of these samples according to BGISEQ-500 manufacturer instructions [16], and then ~~pair-end 100 bp~~ sequenc~~eding -~~

~~was carried out~~ [17]. ~~Finally, a~~Approximately 70 Gb of raw sequencing data (100 bp, paired-end) was generated for each of these samples (Table 1). Raw reads were filtered using SOAPfilter_v2.2 ~~with~~by following the command: -y –p –i 180 –M 2 –Q 10. After filtering the low-quality reads (reads with more than 10% Ns, ambiguous bases; reads with more than 40% bases having quality lower than 10; reads contaminated by adaptors or PCR duplicat~~eion~~s), ~60 Gb clean data were obtained for each of these samples and data showed a ~~high quality~~high-quality reads (>Q35)~~.~~

Species identification and phylogenetic ~~-~~relationship

The taxonomic identification of specimens is a time-consuming pro~~cg~~ress and requires expertise and experience. ~~Here~~In this study, the collections covering the majority of vascular plant lineages were difficult to identify ~~the~~ to species level in a short time. We were able to identif~~yied~~ 254 samples to ~~232~~ species level (from a total of 232) using the specimen morphology and the other 506 samples ~~can be~~were identified to families ~~by~~ using their chloroplast sequences. Thus, in total, we identified 689 ~~species~~ samples from those 761 sequenced ~~samples~~, which belonged to 134 families and 47 orders. Among these families, the majority of the species belonged to Fabaceae (71 ~~species~~taxa), Poaceae (45 ~~species~~taxa) and Asteraceae (38 ~~species~~taxa), respectively.

Commented [Office7]: This table shows figures at the order level not the taxon level, so it would be better to cite a different table or supplementary material that shows numbers per sample

Commented [SS8R7]: Thank you for raising this question. For every sample, the sequencing quality and results were highly similar. Hence, we summarized the data by "Order" in Table 1. However, the detailed breakdown of the data at families/genera/species/ level will presented in our subsequent manuscript with higher quality genome assemblies for the selected species.

Commented [Office9]: What software was used, and what parameters?

Commented [SS10R9]: Raw reads were filtered using SOAPfilter_v2.2 by following the command: -y –p –i 180 –M 2 –Q 10.

Commented [Office11]: What scale are you using for quality? A score of 10 in illumina sequencing is pretty low, it should be more like 20 at the minimum. Could you specify the quality scale used here?

Commented [SS12R11]: By default BGISEQ500 performs the sequencing task with high quality data as an output. Actually 10 or 20 cut off score doesn't have significant difference. After data filter, we checked the quality distribution for clean data and found average quality were more than 35.

We assembled the chloroplast genomes of each species ~~using~~ ~~the~~from clean read data ~~of each species~~ ~~by~~ using NOVOPlasty[2], ~~which is~~ a seed-extension-based *de novo* assembler. We used the complete cds *rbcL* gene sequence of *Arabidopsis thaliana* (downloaded from NCBI, accession number: U91966) as the seed to conduct

Commented [Office13]: Please cite reference

Commented [SS14R13]: Added now

129

130

131

132

133

134

135

136

137

138

139

140

141

142

143

144

145

146

147

148

149

150

the assembly ~~After~~ The NOVOPlasty assembly recovered completely ~~NOVOPlasty,~~ chloroplast genomes of 50

species ~~were finally assembled into the~~in a single circular sequence. For the remaining species, the longest contig assembled by NOVOPlasty ~~were~~ was BLASTed against the chloroplast database (downloaded from NCBI, including 2,503 non-redundant species) and the resulted best-hit sequences (minimum requirement: e-value < 10^{-7} and identity > 95%) were used as references for further assembly using MITObim[1], in this way, we finally recovered complete chloroplast genomes for all 689 species. The assembled chloroplast genomes ranged from 113,621 to 183,602 bp in size (Table S2). We then annotated the assembled chloroplast genomes using DOGMA [18] and GeneWise [19], and we found 72 protein-coding genes in almost all of these vascular plant families except Gnetaceae, Malvaceae, Elaeocarpaceae, and Tectariaceae. For Gnetaceae, we were only able to annotate 52 protein-coding genes in their chloroplast genomes which is consistent with previous studies [20]. We then compared these assembled chloroplast genomes and constructed the phylogenetic tree using ~~—~~RAxML[3] and IQ_TREE[4].~~the gene trees and translated to the species tree~~. ~~We collected~~A total of 78 individual coding genes were identified from 738 samples, ~~and~~ the majority of them were present in 710 to 738 (on average). However, only 18 genes were commonly found in all of the

Commented [Office15]: Add literature reference for this software

Commented [SS16R15]: Added now

Commented [Office17]: At what step did you get rid off mitochondrial reads, which could be pretty similar to chloroplast reads and actually influence the assemblies

Commented [SS18R17]: Generally we get rid off mitochondrial reads first. But, in this study, we first performed the assembly by NOVOPlasty and identified the plasmid reference, then MITObim was used to extract reads that precisely match the reference to create new reference sequences

Commented [Office19]: Please clarify. A chloroplast based tree could only be done with a single concatenated alignment of the genes included. Each individual chloroplast gene should not be use to create an individual gene tree, as chloroplast genes and linked and non-recombinant

Commented [SS20R19]: Thank you for the nice suggestion. We used ASTRAL to infer a species tree in the earlier version of this article. Now we combined all genes to super data and used two methods for tree construction, one is by RAxML using GTRCAT model, another one is by IQ_TREE using the best model to construct the species tree. We have compared these two results in the revised article.

samples studied ~~samples~~. Each gene was aligned using MAFFT ~~afft~~ [21] and every alignment was then processed with TrimAL [22] using the gappyout option to remove poorly aligned positions. Then gene aligments were combined ~~and got total of~~which resulted in 46235 nucleotide positions. Maximum likelihood (ML) ~~gene~~ species trees were constructed ~~by~~ with RAxML ~~package~~ (v8.2.4)[3] with GTRCAT model,

Commented [Office21]: Include literature reference

Commented [SS22R21]: Added now

151

152

153

154

155

156

157

158

159

160

161

162

163

164

165

166

167

168

169

170

171

172

1,000 bootstrap replicates, 5 random ~~seed~~ number ~~were~~ ~~chose~~ ~~seed~~ for the parsimony inference ~~was~~ ~~chose~~ and 26 fern

samples were used ~~as~~ to root the tree. At the same time, ML analyses were performed with IQ-TREE under the substitution model GTR+F+R10 which was determined according to the Akaike information criterion (AIC) and the Bayesian information criterion (BIC) by IQ-TREE. Both RAxML and IQ_TREE ~~gived~~provided ~~the same phylogentic~~concordant phylogentic ~~Astral[23] was~~ major lineages can be observed within Fabales, Rosales, Poales, ~~Lamiales~~ and Malpighiales. In Fabids, Celastrales was the sister group to Malpighiales other than Oxalidales in this study (BS=100%). For Petrosaviidae, the major ordinal relationship was consistent with the previous research, ~~such~~ as well as for Liliales, Asparagales, Poales, Arecales, Commelinales, Pandanales, Zingiberales in —the same clade, ~~and~~ the ~~most~~ earliest~~y~~-branchin~~g~~ed lineage is Alismatales [24]. Relationships between Gentianales, Lamiales and Solanales remained unclear [5, 6], ML tree provided support for Gentianales sister to Lamiales(BS=83%) with sister group to Solanales and Boraginales (BS=100%). We also included 54 species of Poales in the phylogenetic tree which revealed its close rel_ationship with Arecales rather than Pandanales and Dioscoreales.

Genome size, repeat content, and heterozygosity

In order to ensure the quality and effectiveness of the dataset (Table 1), we

conducted several analyses to reveal the basic genomic features of these vascular plants sampled. By using GCE [25] and kmergenie [26] software ~~for~~ and the clean data of each species, we estimated the genome sizes, repeat content and heterozygosity (Figure 2 and Table S1). For several of these ~~tested~~ species, their genome sizes have been previously measured by experimental approaches and are publicly available (http://data.kew.org/cvalues/) (Table S3). We compared these previous estimations ~~from these datasets~~ to the genome sizes estimated by k-mer analysis in this study, ~~to~~ and fo~~u~~ui nd good ~~consistency~~ agreement between them (R^2=0.63) (Figure S1). We found that despite overall ~~high~~ wide variation~~s~~ in

the genome sizes of these plants, most of the families - had relatively compara ble genome sizes. However , Tthe most diversifie d family in terms of genome size was found to be Cupressaceae, in which the genome sizes rangeding from 0.18 Gb in *Cunninghamia lanceolata* (Lamb.) Hook. var. *lanceolata* to 19.26 Gb in *Juniperus pingii* var. *wilsonii* (Rehder) Silba. In addition, the repeat content varied from 10% to 88% on average among in vascular species sampled, with several exceptions in Cornaceae and ,Myrtaceae and Celastraceae. For instance, Myrtaceae (in Myrtales) was found to have the most repetitive genomes (~88% of repetitive content), while Celastraceae (in Celastrales) was found to have the least repetitive genomes (~10% repetitive content). We also found relatively high heterozygosity in these species, ranging from 0.15%‰ to 36.6%‰individually, which probably reflected their nature as wild species.

Commented [Office23]: Do you mean "Celastraceae"? The next sentence talks about Celastraceae not about Cornaceae

Commented [杨婷(Ting24R23]: Both Cornaceae and Myrtaceae contains high repeat elements. We have also listed Celastraceae here now.

Commented [Office25]: Are these averages across several species within the family. Please clarify

Commented [SS26R25]: It's at individually level

Genome assemblies

Despite the limitation of having only one sequencing library constructed for each species, we were able to conduct preliminary genome assembliesy for many of these species, which reflected the the data quality and reuse potential of the data. The heterozygosity was scored based on the Kmer distribution plot according to the method described by Li.Here, Bbased on the estimated heterozygosity and repeat content, we initially selected 17 species from 17 families with relatively simple genome content (heterozygosity rate less than

1% and M 1 scaff). We obtained an average contig N50 of 4.62 kb, and an average

repeat scaffold N50 of 32.2 kb for these genome assemblies. Two species, ~~of~~

content *Alternanthera sessilis* (L.) R.Br. ex DC. and *Senna alata* (L.) Roxb., were

less than

50%) for

~~the~~

genome

assembl

y. We

used

SOAPde

novo2

[27]

(paramet

ers:

pregraph

-K 35

contig -

assembled to contig N50 of 15.2 kb, scaffold N50 of 95.5 kb and contig N50 of 14 kb and the scaffold N50 of 101.1 kb, respectively (Table S4). We then carried out Benchmarking Universal Single-Copy Orthologs (BUSCO)(version 3.0.1) analysis [28] to find the completeness ~~to be ~89.1% for~~of all these 17 genome assemblies. O~~(i~~n average, genome completeness was found to be ~89.1%, 1243 BUSCOs ~~to be~~were complete and single-copy and ~~in average~~ 40 BUSCOs ~~to be~~were complete and duplicated~~,~~ (from a~~of the~~ total of 1440 BUSCOs). The average number~~s~~ of fragmented and missing BUSCOs were 55 and 101, respectively (Table S5). Our preliminary assemblies were of good quality, providing a useful reference for future efforts to establish complete reference genomes for all these plant species. In addition to the current attempt of genome assembly, continuing efforts are being carried out to finish the preliminary assemblies of the other species and these are being deposited ~~with~~ and linked with existing, ~~the~~ already public, sequencing data.

Data access and reuse potential

The data generated here includes~~d~~ ~~the~~ images, raw sequencing data, assembled chloroplast genomes and preliminary nuclear genome assemblies. All the data have been organized and linked to a top level accession in the *GigaScience* GigaDB repository (DOI:XXXX), containing the lists of all the species and the links to

the each species page of each species. Linked to thisIn addition, each species has a DOI assigned to them containing the information onf

collection number, an image of the plant during sampling, SRA accession number ofor the raw data, the a data file containing the assembled chloroplast sequence (these current chloroplast sequences can also

Commented [Office27]: In what format? Does it contain annotations?

Commented [杨婷(Ting28R27]: It is in fasta format

be found in Table S2), ~~the~~ a data file containing the preliminary assembled genome sequence (available only for some species and will continue to be updated when each assembly is completed). ~~The~~ Voucher specimens are stored in the Herbarium of~~,~~ China National GeneBank (HCNGB), and digitized images for every sheets are

also being made available in GigaDB alongside the sequencing data. All ~~the~~ raw data are stored in the NCBI SRA repository under the project number PRJNA43840. In addition to the description in SRA, the SRA accession number of raw data is also included in the GigaDB entries, thus the raw data of specific~~ced~~ species can be traced from GigaDB. Datacite and GigaDB (http://doi.org/10.5524/100502) metadata is all linked, and any future updates made on the GigaDB dataset provides traceable records.

Commented [Office29]: Could you provide doi for Datacite?

Commented [SS30R29]: Liu et al (2018): Supporting data for "Digitizing a Botanical Garden: High-depth whole genome sequencing of 689 vascular plants from Ruili Garden" GigaScience Database. http://doi.org/10.5524/100502

The high-depth whole genome sequencing data together with ~~the~~ images and voucher specimens can be reused in different ways and will be valuable for future applications. First of all, in addition to the phylogenetic analysis carried out here ~~based on~~using the assembled chloroplast genomes, future evolutionary analysis can be carried out to ~~depict~~ study the evolution of specific genes ~~(~~after assembling~~y of these genes~~ them from raw reads~~)~~, ~~figure out~~as well as investigating particular ~~-~~features of plant genome evolution, ~~features including evolution of repeats, polyploidization, whole genome duplication, etc. (with optimization or developing of suitable methods).~~ Secondly, the data can be used ~~for~~ to improve future genome assembl~~y~~ies of these plant species. For example, ~~Other than~~ utilizing the information on repeat content, heterozygosity and genome size estimation provided here to tailor new sequencing and genome assembly strategies of these plant genomes, ~~the~~as well as

integrating the sequencing data itself ~~can also be integrated~~ in ~~further~~ other genome assemblies. By directly using the sequencing data obtained

~~sequencing data~~ from this study, it would be ~~— more~~ easier~~y~~ and more efficient to assemble the remaining sequenced plant genomes. The ~70 Gb data can ~~either~~ be used for genome assembly in combination with either contig reconstruction of the second generation based sequenc~~eing~~ing reads~~based genome assembly~~, or for error correction ~~for~~ of the third generation long sequence reads ~~based genome assembly~~. Last but not least, this dataset can also be used for developing new methods for species identification either based on sequencing data or based on images of plants and to~~,~~ resolv~~eing~~e ~~the~~ phylogenetic relationships based on whole genome sequencing data~~,~~ ~~etc~~among others. For example, deep learning can be applied to develop plant identification using this dataset as a good training set. Providing this comprehensive dataset which can be easily accessed by ~~the~~ researchers and also the general public, we think it would be reused in many ways beyond what ha~~ved~~ been mentioned here.

1
2

254
255
256
257
258
259
260
261
262
263
264
265
266
267
268
269
270
271
272
273
274
275

Discussion

~~The evolution of vasculature was a major event in plant history.~~ The current understanding on the evolution of plants and its diversity in a phylogenomic context is limited due to the non-availability of genome-scale information across phylogenetically diverse species [29]. In this study, we provide a dataset of high-depth whole genome sequencing of 689 vascular plant species with voucher specimens, covering 134 families and 47 orders. These samples were obtained from Ruili Botanical Garden in Yunnan Province of China, near the border between China and Myanmar, reflecting the rich plant diversity in that region. The high-depth whole genome sequencing data generated here have been used to estimate genomic features including genome size, repeat content, and heterozygosity, which can provide guidance to the future studies aiming at establishing reference genomes for these species. The high-depth whole genome data can also be used in assembling ~~the~~ chloroplast genomes, as well as some conserved nuclear genes, thus providing useful information for evolution and gene function studies.

In this study, we scaled up the plant whole genome sequencing effort to sequence hundreds of plant species. We only constructed a single short insert ~~size~~ library (200 bp) for each of the species and generated ~60 Gb of whole genome sequencing data. It would be insufficient to assemble good draft genomes for the majority of the species just based on single library data,

because multiple short insert ~~size~~ libraries and also mate pair (large insert size)

previous libraries. ~~However,~~ In addition ~~to future reuse of the current data~~, our study~~,~~

efforts to tested for the first time, ~~tested~~ the feasibility of large-scale

assemble reference genomes based on second generation sequencing data ~~would~~ have required

whole genome sequencing, which is already underway for the Earth BioGenome Project (EBP) [7] and 10 thousand Plant Genome Projects (10KP) [29]. ~~With particular relevance for the 10KP, T~~This study provided experiences for plant sampling, sample logistics~~,~~ ~~sample~~ and management, DNA extraction, sequencing library preparation, sequencing, and data analysis and ~~data~~ management. Aiming at sequencing more than 10,000 plant species, 10KP would require ~~and~~ to establish a robust infrastructure for sample and data management, as potentially investigated ~~at a pilot scale by~~in this pilot study.

Availability of Supporting Data

The specimens, leaf samples and DNA solutions of all collections are ~~maintained~~ stored at the China National GeneBank (CNGB) Herbarium. The raw sequencing data described in this article are available in the NCBI SRA repository~~,~~ under the project number PRJNA43840. DNA Extraction [27] and BGISEQ-500 WGS library construction protocols can be found in protocols.io [28].

738 chloroplast genomes and 17 assembl~~y~~edy genomes together with raw data supporting the results of this article ~~is~~ are available via the *GigaScience* GigaDB repository, and will be continu~~ed~~ouslyed ~~to be~~ updated and linked to the GigaDB entries as ~~the~~ new assemblies are completed.

Additional file 1

Table S1. List of ~~the~~ samples included in this study with voucher information, current kmer based estimation of genome sizes, repeat content and heterozygosity. Identified

Additional files

300

collections were listed with species names, while unidentified ones with only family and order information. 738 samples with assembled chloroplast genome were marked with *, whereas 17 samples with assembled genomes were marked with §.

Table S2. All the assembled chloroplast genomes and their lengths.

Table S3. G~~The g~~enome information ~~has been~~ previously measured and ~~are~~ publicly available on the database.

Table S4. Summary of preliminary genome assemblies of 17 species of vascular plants ~~from 17~~

Table S5. Summary of BUSCO analysis for 17 species of vascular plant families.

families.

Additional file 2

Figure S1. ~~The~~ A comparison of genome sizes measured ~~with~~by the experimental approaches ~~in datasets compared~~ to the k-mer estimated genome sizes in this study.

Figure S2. Phylogeny of vascular plants of the Ruili Botanical Garden. The tree by RAxML. The inner circle and the outer circus colors represent different families and orders. The clade color represents bootstrap values from red to gray (bootstrap range from 50 to 100).

~~le~~

Abbreviations

EBP: Earth BioGenome Project.

Gb: Gigabase pair

10 KP: 10 thousand Plant genome Project

Commented [Office31]: Chloroplast genes violate the assumptions of coalescence analyses, as they are grouped and linked into a single molecule that is inherited as a single unit. They can have different evolutionary rates, but per definition, they all have the same evolutionary history, and thus coalescence analyses should not be used with chloroplast genes. I suggest instead doing a ML reconstruction with the concatenated matrix of all chloroplast genes.

Commented [SS32R31]: Yes, we agree with your suggestion that Astral is not a suitable method for our analysis. We now combined all genes to super data and used two different methods for the tree construction, one is by RAxML using GTRCAT model, another one is by IQ_TREE using the best model to construct the species tree. We have compared these two results in the revised article.

bp: base pair

BUSCO: Benchmarking Universal Single-Copy Orthologs.

HCNGB: Herbarium~~,~~ of China National GeneBank. ML: Maximum likelihood. WGS: Whole Genome Sequencing.

Competing interests

All authors declare that they have no competing interests.

Funding

This work was supported by ~~the~~ grants of Basic Research Program, the Shenzhen Municipal Government, China (No.JCYJ20150529150505656~~)~~ and ~~(~~No.JCYJ20150831201643396), as well as ~~the~~ funding ~~to~~ from State Key Laboratory of Agricultural Genomics (No.2011DQ782025), ~~and~~ Guangdong Provincial Key Laboratory of Genome Read and Write(No.2017B030301011), The Construction of China National GeneBank (Yunnan GeneBank) (Yunnan and ~~pro~~vince, 2015DA008, P.R. China)

Author contributions

XL conceived this study. XL and HL drafted the manuscript. HL managed the project. JPW, XBW, LC, XFH, HCC, JLY, YW, RCM, JL, JMZ collected the samples. TY lead~~ed~~ identification of voucher specimens. TY, WXM, BS, YF,

YC, HYC analyzed the data. TY, XLC, MW, ZHH constructed the phylogenetic

tree. GHH, WSL, HCZ, HCC, YL extracted DNA and performed genome sequencing. SKS revised and edited the manuscript. All the authors have read and approved the final manuscript.

Acknowledgments

The authors would like to express their sincere thanks to the local people and Government of Yunnan province, and Forestry Institute of Dehong Prefecture for their help in sample collection. We ~~They~~

6.

7.

would also like to thank the taxonomic experts in PE (Herbarium Institute of Botany, Chinese Academy of Sciences) for identification.

Finally, we are thankful to the production team of China National GeneBank, Shenzhen, China.

References

1. Pennisi E. Plant biology. Green genomes. Science. 2011;332 6036:1372-5. doi:10.1126/science.332.6036.1372.
2. Bolger ME, Weisshaar B, Scholz U, Stein N, Usadel B and Mayer KF. Plant genome sequencing - applications for crop improvement. Curr Opin Biotechnol. 2014;26:31-7. doi:10.1016/j.copbio.2013.08.019.
3. Desta ZA and Ortiz R. Genomic selection: genome-wide prediction in plant improvement. Trends Plant Sci. 2014;19 9:592-601. doi:10.1016/j.tplants.2014.05.006.
4. Leitch I, Coker T and Leitch A. Plant genomes - progress and prospects. 2017.
5. Kew RBG. The state of the world's plants report–2016. Royal Botanic Gardens, Kew. 2016.

Matasci N, Hung L-H, Yan Z, Carpenter EJ, Wickett NJ, Mirarab S, et al. Data access for the 1,000 Plants (1KP) project. Gigascience. 2014;3 1:17.

Lewin HA, Robinson GE, Kress WJ, Baker WJ, Coddington J, Crandall KA, et al. Earth BioGenome Project: Sequencing life for the future of life.

Commented [Office33]: Are you referring to a herbarium? Please clarify

15.

16.

Proc Natl Acad Sci U S A. 2018;115 17:4325-33.

8. de Vere N, Rich TC, Trinder SA and Long C. DNA barcoding for plants. Methods Mol Biol. 2015;1245:101-18. doi:10.1007/978-1-4939-1966-6_8.

9. Staats M, Erkens RH, van de Vossenberg B, Wieringa JJ, Kraaijeveld K, Stielow B, et al. Genomic treasure troves: complete genome sequencing of herbarium and insect museum specimens. PLoS One. 2013;8 7:e69189. doi:10.1371/journal.pone.0069189.

10. Osmundson TW, Robert VA, Schoch CL, Baker LJ, Smith A, Robich G, et al. Filling gaps in biodiversity knowledge for macrofungi: contributions and assessment of an herbarium collection DNA barcode sequencing project. PLoS One. 2013;8 4:e62419. doi:10.1371/journal.pone.0062419.

11. Li X, Yang Y, Henry RJ, Rossetto M, Wang Y and Chen S. Plant DNA barcoding: from gene to genome. Biol Rev Camb Philos Soc. 2015;90 1:157-66. doi:10.1111/brv.12104.

12. Straub SC, Parks M, Weitemier K, Fishbein M, Cronn RC and Liston A. Navigating the tip of the genomic iceberg: Next-generation sequencing for plant systematics. Am J Bot. 2012;99 2:349-64. doi:10.3732/ajb.1100335.

13. Male PJ, Bardon L, Besnard G, Coissac E, Delsuc F, Engel J, et al. Genome skimming by shotgun sequencing helps resolve the phylogeny of a pantropical tree family. Mol Ecol Resour. 2014;14 5:966-75. doi:10.1111/1755-0998.12246.

14. Besnard G, Christin PA, Male PJ, Coissac E, Ralimanana H and Vorontsova MS. Phylogenomics and taxonomy of Lecomtelleae (Poaceae), an isolated panicoid lineage from Madagascar. Ann Bot. 2013;112 6:1057-66. doi:10.1093/aob/mct174.

Wu C and Yang T. DNA Extraction for plant samples by CTAB.

s.io.pzqdp5w.

Gao S, Mu F, Yang Z, Liu X, Jiang H, Liao S, et al. BGISEQ-500 WGS library construction. 2018; doi:10.17504/protocols.io.ps5dng6.

17. Gao S, Mu F, Yang Z, Liu X, Jiang H, Liao S, et al. BGISEQ-500

Gi g a sc ie n c e. 2 0 1 8; d oi :1 0. 1 7 5 0 4/ pr ot o c ol

403
404
405
406
407
408
409
410
411
412
413
414
415
416
417
418
419
420
421
422
423
424
425
426
427
428
429
430
431
432
433
434
435

Sequencing. doi:10.17504/protocols.io.pq7dmzn.

18. Wyman SK, Jansen RK and Boore JL. Automatic annotation of organellar genomes with DOGMA. Bioinformatics. 2004;20 17:3252-5. doi:10.1093/bioinformatics/bth352.

19. Birney E, Clamp M and Durbin R. GeneWise and Genomewise. Genome Res. 2004;14 5:988-95. doi:10.1101/gr.1865504.

20. Hsu CY, Wu CS, Surveswaran S and Chaw SM. The complete plastome sequence of Gnetum ula (Gnetales: Gnetaceae). Mitochondrial DNA A DNA Mapp Seq Anal. 2016;27 5:3721-2. doi:10.3109/19401736.2015.1079874.

21. Katoh K, Misawa K, Kuma K and Miyata T. MAFFT: a novel method for rapid multiple sequence alignment based on fast Fourier transform. Nucleic Acids Res. 2002;30 14:3059-66.

22. Capella-Gutiérrez S, Silla-Martínez JM and Gabaldón T. trimAl: a tool for automated alignment trimming in large-scale phylogenetic analyses. Bioinformatics. 2009;25 15:1972-3.

23. Mirarab S, Reaz R, Bayzid MS, Zimmermann T, Swenson MS and Warnow T. ASTRAL: genome-scale coalescent-based species tree estimation. Bioinformatics. 2014;30 17:i541-i8.

24. Chase MW. Monocot relationships: an overview. Am J Bot. 2004;91 10:1645-55. doi:10.3732/ajb.91.10.1645.

25. Liu B SY, Yuan J, Hu X, Zhang H, Li N, Li Z, Chen Y, Mu D, Fan W. Estimation of genomic characteristics by analyzing k-mer frequency in de novo genome projects. arXiv preprint. 2013; doi:arXiv:1308.2012.

26. Chikhi R and Medvedev P. Informed and automated k-mer size selection for genome assembly. Bioinformatics. 2014;30 1:31-7. doi:10.1093/bioinformatics/btt310.

27. Luo R, Liu B, Xie Y, Li Z, Huang W, Yuan J, et al. SOAPdenovo2: an

empirically improved memory-efficient short-read de novo assembler. Gigascience. 2012;1 1:18.

28. Simão FA, Waterhouse RM, Ioannidis P, Kriventseva EV and Zdobnov EM. BUSCO: assessing genome assembly and annotation completeness with single-copy orthologs. Bioinformatics. 2015;31

19:3210-2.

29. Cheng S, Melkonian M, Smith SA, Brockington S, Archibald JM, Delaux P-M, et al. 10KP: A Phylodiverse Genome Sequencing Plan. GigaScience. 2018.

Figure legends

Figure 1. Sampling localities of this project. Sampling was conducted mainly in Ruili Botanical Garden in Southwest China, near the China-Myanmar border, and shown in red rectangles.

Commented [Office34]: This figures shows red circles for the sampling localities. Please clarify.

Figure 2. **The ordinal phylogeny was "drop-tips" from Figure 3.** GThe genome sizes, repeat content and heterozygosity statistics of the nuclear genomes assembled in

this study. (a)

Ggenome sizes in GB, (b) repeat content as percentage of total genome (%), (c) heterozygosity ration for samples in this study. Ordinal Ccladogram was generated from the 78 chloroplast gene phylogeny.

Figure 3. Phylogeny of vascular plants of the Ruili Botanical Garden. The tree shows the coalescent tree based on 78 chloroplast genes. In inner circus, CHere the colors represent different ordersfamilies and out circus colors represent different orders., as labelled..

Commented [Office35]: In this figure, some branches within the orders have different colors, does it mean that the sample was misidentified, or that the color coding is not correct. Please verify and modify or explain accordingly.

Commented [Office36]: Chloroplast genes violate the assumptions of coalescence analyses, as they are grouped and linked into a single molecule that is inherited as a single unit. They can have different evolutionary rates, but per definition, they all have the same evolutionary history, and thus coalescence analyses should not be used with chloroplast genes. I suggest instead doing a ML reconstruction with the concatenated matrix of all chloroplast genes.

Tables

Table 1 Summary of the sequencing data in this study.

New references added

1. Hahn, C., L. Bachmann, and B. Chevreux, *Reconstructing mitochondrial genomes directly from genomic next-generation sequencing reads—a baiting and iterative mapping approach.* Nucleic acids research, 2013. **41**(13): p. e129-e129.
2. Dierckxsens, N., P. Mardulyn, and G. Smits, *NOVOPlasty: de novo assembly of organelle genomes from whole genome data.* Nucleic acids research, 2016. **45**(4): p. e18-e18.
3. Stamatakis, A., *RAxML version 8: a tool for phylogenetic analysis and post-analysis of large phylogenies.* Bioinformatics, 2014. **30**(9): p. 1312-1313.
4. Nguyen, L.-T., et al., *IQ-TREE: a fast and effective stochastic algorithm for estimating maximum-likelihood phylogenies.* Mol Biol Evol, 2014. **32**(1): p. 268-274.

5. Bremer, K., et al., *A phylogenetic analysis of 100+ genera and 50+ families of euasterids based on morphological and molecular data with notes on possible higher level morphological synapomorphies.* Plant Systematics and Evolution, 2001. **229**(3-4): p. 137-169.

6. Refulio-Rodriguez, N.F. and R.G. Olmstead, *Phylogeny of lamiidae.* American Journal of Botany, 2014. **101**(2): p. 287-299.

 Click here to access/download;Table;Table 1.docx

Table 1. Summary of the sequencing data in this study

Order	Raw base (Gb)	Raw data GC (%)	Raw data Q20	Raw data Q30
Alismatales	66.3873	43.64	95.34	86.48
Apiales	70.0075	35.42	96.40	88.40
Araucariales	74.14	32.87	96.50	88.85
Arecales	68.8318	39.95	95.84	87.20
Asparagales	70.3465	37.97	96.16	87.87
Asterales	67.8382	37.41	95.83	87.20
Brassicales	68.474	37.89	95.99	87.45
Buxales	65.44	42.34	95.38	86.00
Caryophyllales	68.6558	38.04	95.73	87.03
Celastrales	75.8133	38.12	96.56	88.57
Commelinales	65.02	36.80	95.58	86.81
Cornales	76.396	36.49	96.44	88.63
Crossosomatales	60.2	37.17	95.36	86.54
Cucurbitales	65.11	35.73	95.50	86.22
Cupressales	73.54	36.12	96.78	89.01
Cyatheales	75.76	41.32	96.64	88.37
Dioscoreales	78.9	41.47	94.99	85.65
Dipsacales	58.6267	37.58	96.22	87.52
Equisetales	67.3	39.98	94.92	84.77
Ericales	68.1109	38.01	96.46	88.02
Fabales	69.9439	35.50	96.14	87.75
Fagales	68.14	36.81	96.13	87.90
Gentianales	70.1155	36.49	96.36	88.27
Gnetales	71.1267	39.77	96.87	89.24
Lamiales	69.3291	37.47	95.94	87.40
Laurales	71.9425	40.22	96.04	87.83
Liliales	71.4133	41.00	96.73	89.15
Magnoliales	69.0988	38.88	96.12	88.01
Malpighiales	68.1842	35.83	96.40	88.23
Malvales	66.2106	37.19	96.26	88.07
Myrtales	70.7924	38.82	96.23	88.20
Oxalidales	68.3533	34.91	95.61	87.20
Pandanales	72.6733	42.07	96.41	88.31
Pinales	61.04	39.56	93.91	82.96
Piperales	63.2533	40.50	96.23	87.84
Poales	69.6407	44.07	95.56	86.73
Polypodiales	68.588	41.39	96.12	87.69
Proteales	69.0733	39.47	96.49	88.23
Ranunculales	67.5644	38.69	95.68	86.80

Rosales	70.0468	36.72	96.36	88.18
Santalales	69.07	38.11	96.47	88.31
Sapindales	70.5628	36.83	96.14	87.89
Saxifragales	70.84	37.74	96.77	89.36
Schizaeales	62.57	43.84	96.83	89.17
Solanales	72.2389	38.38	96.30	87.93
Vitales	65.235	39.17	95.44	86.71
Zingiberales	67.4956	40.57	95.99	87.51

Figure 1

Click here to access/download;Figure;Figure 1.pdf

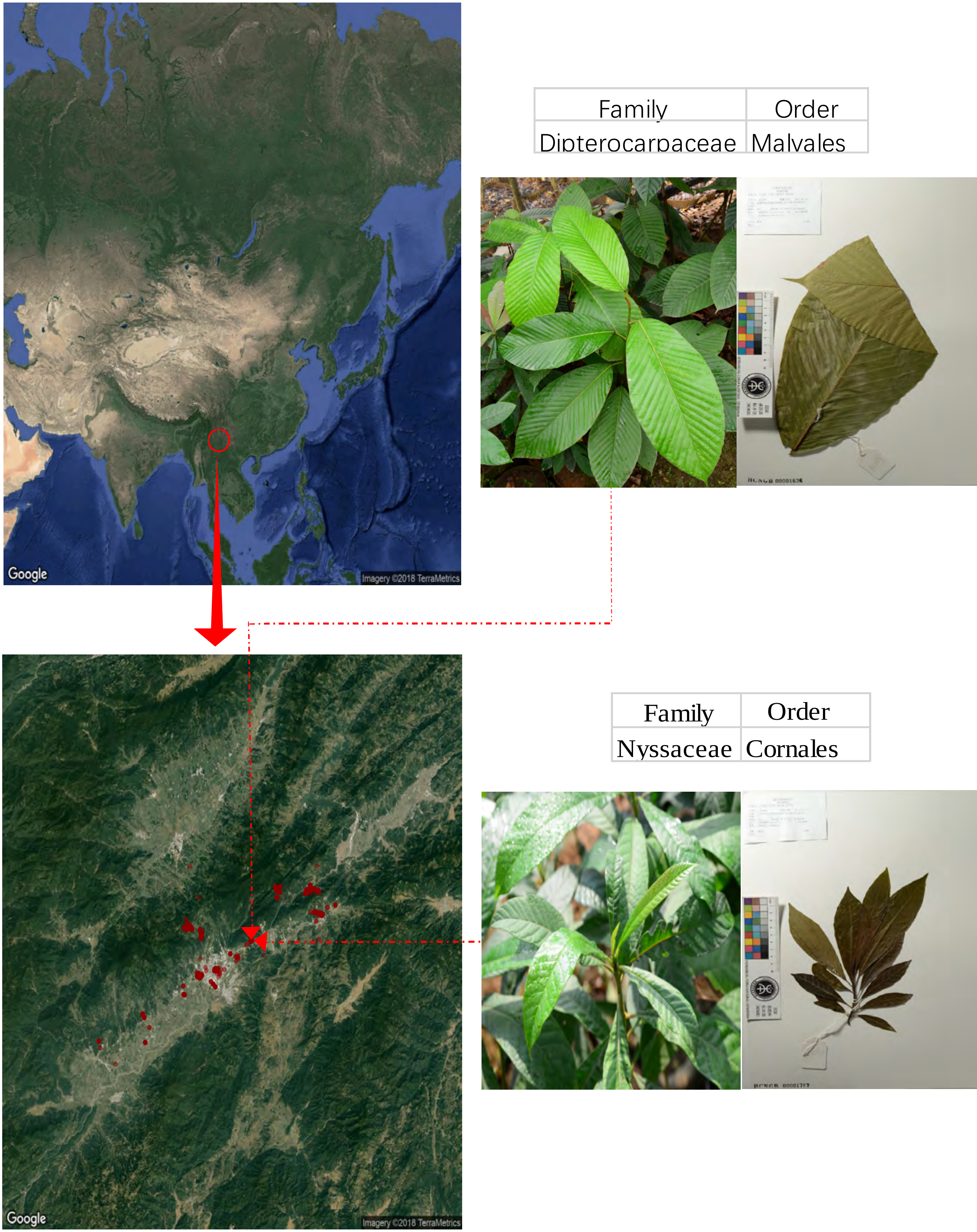

Family	Order
Dipterocarpaceae	Malvales

Family	Order
Nyssaceae	Cornales

Figure 2

Click here to access/download;Figure;Figure 2.pdf

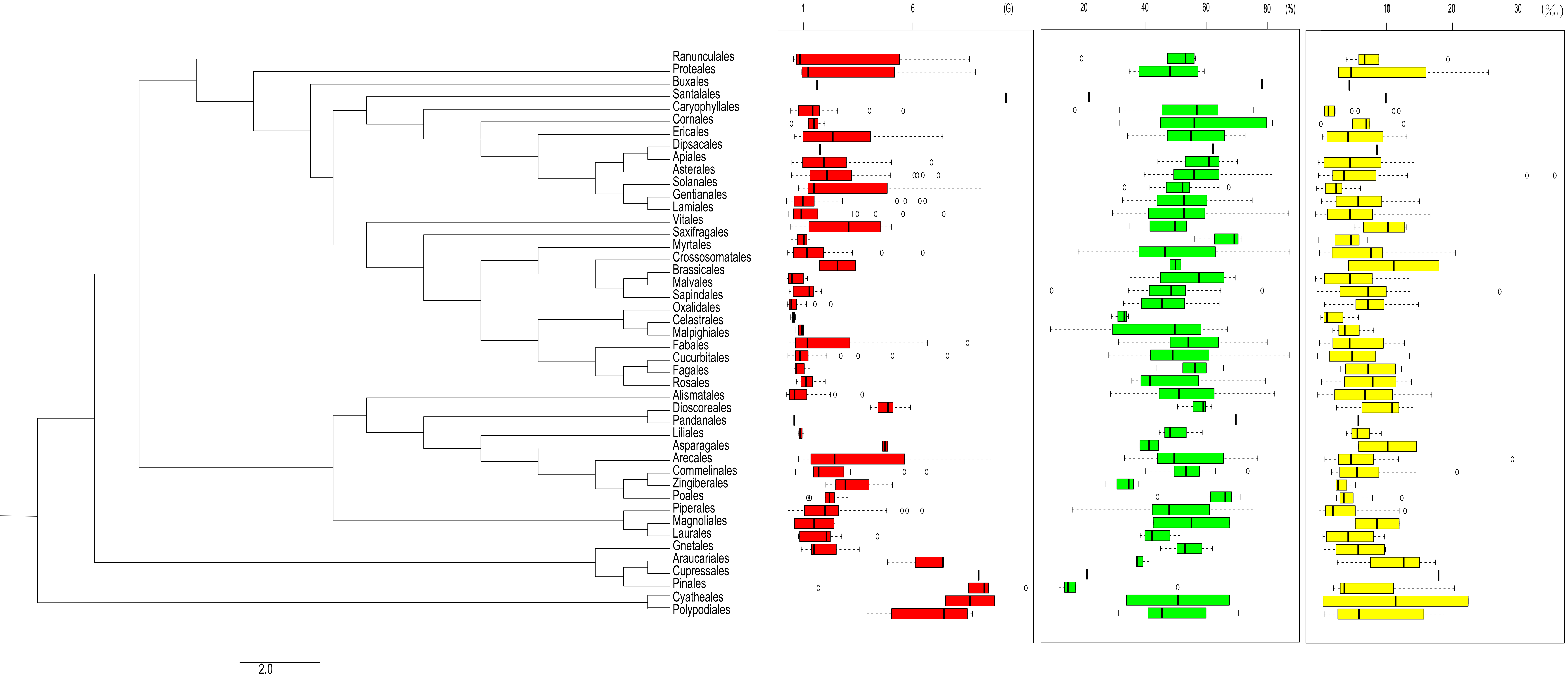

Figure 3

Click here to access/download;Figure;Figure 3.pdf

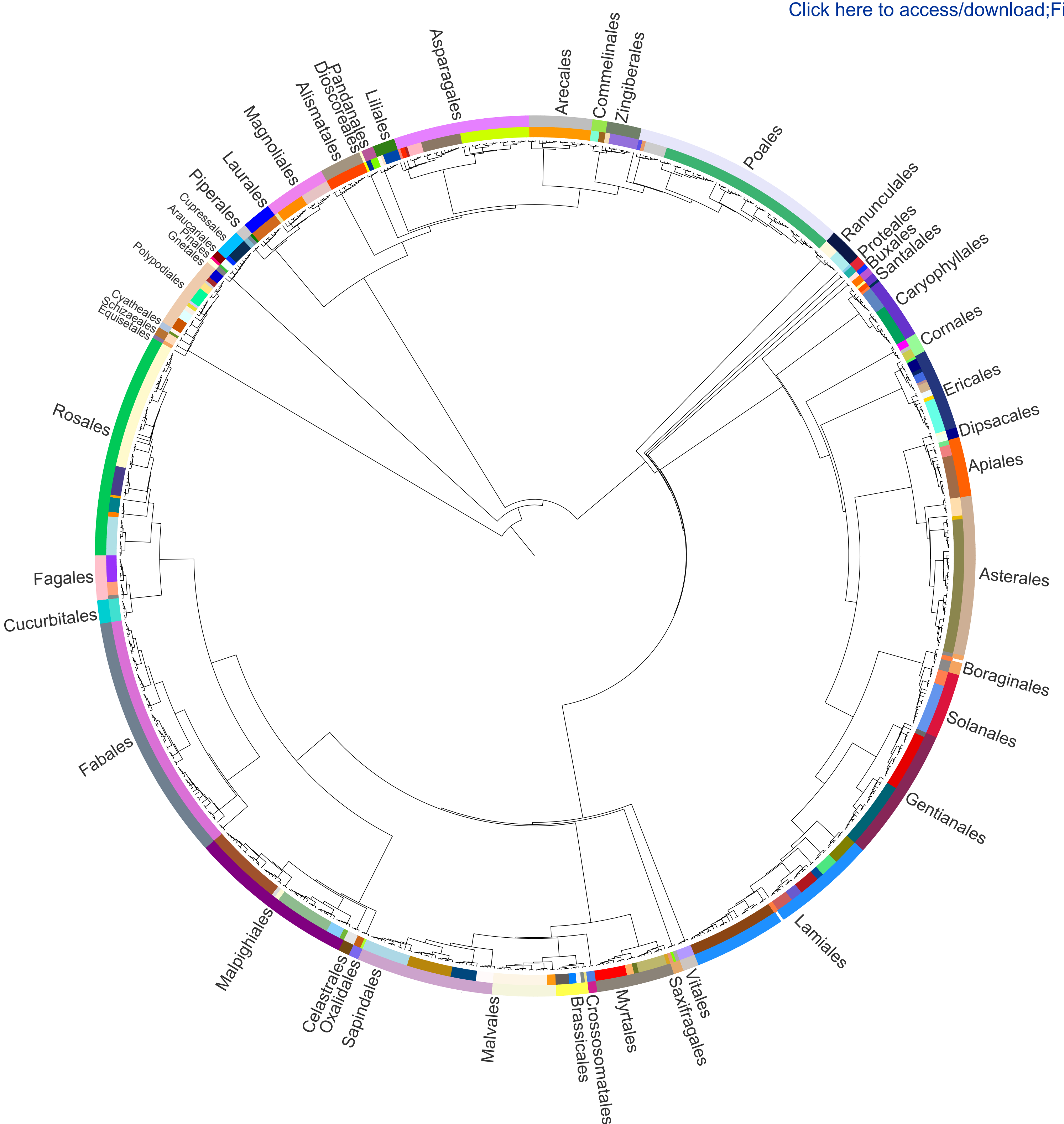

Supplementary Figure S1

Click here to access/download
Supplementary Material
Figure S1.pdf

Supplementary Figure S2

Click here to access/download
Supplementary Material
Figure S2.pdf

Supplementary Table S1

Click here to access/download
Supplementary Material
Table S1.xlsx

Supplementary Table S2

Click here to access/download
Supplementary Material
Table S2.xlsx

Supplementary Table S3

Click here to access/download
Supplementary Material
Table S3.xlsx

Supplementary Table S4

Click here to access/download
Supplementary Material
Table S4.xlsx

Supplementary Table S5

Click here to access/download
Supplementary Material
Table S5.xlsx

 Click here to access/download;Personal Cover;cover_letter.docx

Dear Dr. Scott,

Sub: Submission of the revised manuscript GIGA-D-18-00121.

We are glad to submit the thoroughly revised version of our manuscript entitled "Molecular Digitization of a Botanical Garden: High-depth whole genome sequencing of 689 vascular plants from the Ruili Botanical Garden"

The comments of the reviewers were highly insightful and enabled us to greatly improve the quality of our manuscript. According to their advice, we have carefully revised our manuscript. At the same time, we have added Dr. Xun Xu (the CEO of BGI) as co-author due to his immense support and suggestions to improve and revise our manuscript.

The following are the major revisions made in the manuscript:

Both two reviewers mentioned that to infer a species, plastid data should be combined and ML method should be used to infer a tree. Therefore;

For Figure 1, we have now updated the figure with more clear and better images of the specimen

For Figure 3, we combined plastid genes and constructed the species tree by IQ_TREE using the best model (GTR+F+R10).

For Figure S2, we used the same gene data by RAxML using GTRCAT model and constructed a concordant tree.

In the reviewer reports, we present the point-by-point responses to each of the comments and suggestions of the reviewer, we also made substantial revision in the manuscript with track changes. However, we have uploaded both clean and track changes version for your kind perusal. We strongly believe that these revisions in the manuscript and our accompanying responses are sufficient to make our manuscript suitable for publication in GigaScience.

We look forward to hearing from you at your earliest convenience.

Yours sincerely,

Xin Liu

Reviewer reports:

Reviewer #1: The authors present an awesome quantity of whole genome sequence data representing hundreds of plant species. That the data is being made openly available represents a substantial contribution to the research community. Clearly the analysis of the data is a work in progress. Only 17 of the samples are subjected to whole genome assembly; for 738 just the chloroplast genomes were assembled. The sampling strategy was not exactly ideal: the plants were collected from a single botanic garden, and as such you might expect a somewhat arbitrary selection of species; given the numbers involved and the intent of the study this is perhaps not so important. What is more surprising is that the identifications of the plants is so limited. If these are part of a curated collection, you might expect them to be determined at least to genus; if it represents wild-collected specimens, you would ideally consult (local) botanical expertise for more precise determinations. Certainly for this kind of study it would increase the value of the data markedly to know from what organisms the sequences originated. Since there are voucher specimens lodged in herbaria, and it seems considerable resources will be available - images etc. - the identifications can presumably still be achieved and the database updated. It just seems a bit odd; but if the paper is viewed as the presentation of a vast quantity of usable semi-raw data then perhaps this is OK too. I have not assessed the data itself and have no great insight to provide into the bioinformatic methods - they look superficially pretty standard. The authors have compared previously known genome sizes to that inferred from their data and it appears similar; although only 17 of the genomes were assembled (those with lower heterozygosity and repeats; apparently assemblies of more will be added later) those each included around 90% of benchmarking universal single-copy orthologs. Since the data, from raw reads onwards should be available, the community will be in a position to assess it in detail post publication.

1. "We then compared these assembled chloroplast genomes and constructed the phylogenetic tree using the gene trees and translated to the species tree"
You have used astral to infer a species tree from the plastid data? This seems inappropriate in principle; the data ought to be linked and could be combined e.g. under ML to infer a tree without using a coalescence-based method. Plus, if there is conflict in this dataset, coalescence is

not the best way to model it I would argue.

Response:

Thank you for the nice suggestion. Yes, we used ASTRAL to infer a species tree in the earlier version of this article. We also combined all genes to super data and used two methods for tree construction, one is by RAxML using GTRCAT model, another one is by IQ_TREE using the best model (GTR+F+R10) to construct the species tree. We have compared these two results in the revised article.

2. The conclusion towards the end of the data description (although it instead feels like discussion material): "deep learning can be applied to develop plant identification using this dataset as a good training set" seems a little far-fetched in this context. Huge as it is, I'm not convinced that this dataset represents useful input for this kind of application - the plants would need to be reliably determined, at the least, and I believe image-based IDs need a great deal more training data.

Response:

Thank you for the suggestion. We have now revised the sentence as per your suggestion as follows:

3.Discussion:

"The evolution of vasculature was a major event in plant history."

This is not directly relevant - I'd suggest to consider an alternative leading sentence here.

Response:

Thank you for the suggestion. We have removed this sentence from the main text and replaced with “The current understanding on the evolution of plants and its diversity in a phylogenomic context is limited due to the non-availability of genome-scale information across phylogenetically diverse species.”

4. "We only constructed a single short insert size library for each of the species and generated

~60 Gb whole genome sequencing data. It would be insufficient to assemble good draft genomes for majority of the species just based on single library data, because previous efforts to assemble reference genomes based on second generation sequencing data would require multiple short insert size libraries and also mate pair (large insert size) libraries. However,"
I don't understand what you're saying here. Are you saying that it would not have been possible to assemble genomes from much of the current data (surely not?)? Or are you suggesting that something has changed and that although in the past it would not have been possible, now it is? The lack of long reads marks a difference between this effort and that proposed in the 10KP project; how are the data likely to be comparable?
Response:
Yes, we do agree with your comments. Though a single library can be used to assemble a genome, but may not yield a high-quality genome assembly. However, the current data was potentially used for several analyses such as gene finder, plastid and mitochondrial assembly. At present, we are using these data in combination with 10x genomics to get high quality genome data.

5. "With particular relevance for the 10KP, this study provided experiences for plant sampling, sample logistics, sample management, DNA extraction, sequencing library preparation, sequencing, data analysis and data management."
What exactly was learnt about all this? Qualification needed here - I have no doubt the authors gained a great deal of insight in the process of generating this huge dataset and it would be well worth sharing.
Response:
We have optimized the DNA extraction protocol and also published the protocol (Wu and Yang, 2018). Soon we will also launch the DNA extraction kit.
Wu C and Yang T. DNA Extraction for plant samples by CTAB. Gigascience. 2018; doi:10.17504/protocols.io.pzqdp5w

We also have just finished a guideline about sample submission for 10KP which including sample preparation (fresh sample, DNA sample and RNA sample), sample packing and shipping. The specific guidelines will be soon available in our 10KP website (https://db.cngb.org/10kp/).

6. "DNA Extraction [27] and BGISEQ-500 WGS library construction protocols can be found in protocols.io [28]."

Are these the appropriate references...?

Response: Yes, these are valid and appropriate references.

Table 1: does it really make sense to summarize this by order? Surely the quantities of raw data are only comparable if you break that down to families/genera/species/individuals?

Response:

Thank you for raising this question. For every sample, the sequencing quality and results were highly similar. Hence, we summarized the data by "Order" in Table 1. However, the detailed breakdown of the data at families/genera/species/ level will presented in our subsequent manuscript with higher quality genome assemblies for the selected species.

Reviewer #2: This paper sequences and assembles the genomes of diverse plant species found at the Ruili Botanical Garden, China. The authors generate approximately 60 Gb short sequence read data from 760 samples, and perform a range of analyses suitable for low-coverage draft genomes such as repeat content characterization and plastid assembly. These analyses show considerable variation in many genomic properties across plants. While the paper is descriptive, and is limited by using the same sequencing approach and assembly parameters for all samples, I do think it is impressive in terms of the scale of sequencing, and will serve as a good test case for large-scale sequencing of diverse sample sets.

Major comments

1. This paper is likely to be used as a test case for sequencing diverse samples across a flora. As such, I'm sure the reader would like to know the relationship between coverage and contiguity in the assemblies. Also, were mitochondrial genome assemblies attempted, and were these successful? More generally, it would be useful to explain why the same sequencing effort was allocated to all samples regardless of known genome size (and would you suggest others

follow this approach in the future?).

Response

In this article, we have mentioned that ~70 Gb of raw sequencing data was generated for each of the sample. Combined with estimated genome size by GCE and kmergenie we can infer the genome coverage. For mitochondrial genome, we completed the initial test run, and successfully assembled the mitochondrial genome of five Species. Based on these positive assemblies, we have now actually initiated the mitochondrial genome assembly of remaining species.

To calculate the genome size, only 10-30X data is usually enough, that's why we sequenced ~70 Gb as the preliminary step. Of course, for some samples with large genome size, we couldn't estimate the genome size, but their sequencing data was used in assembling the chloroplast genomes, as well as some conserved nuclear genes. Hence, once we obtained the genome size information, then we selected the appropriate method for the genome assembly. Overall, based on our experience, the sequencing method employed in this study can be recommended for the smaller genomes only.

2. As far as I'm aware, Astral is a species tree approach that should be used on many independent loci. I don't think it's suitable for the plastid as all loci are linked. An alternative phylogenetic approach should be used (such as partitioned analysis in IQ-TREE).

Response

Yes, we agree with your suggestion that Astral is not a suitable method for our analysis. We now combined all genes to super data and used two different methods for the tree construction, one is by RAxML using GTRCAT model, another one is by IQ_TREE using the best model to construct the species tree. We have compared these two results in the revised article.

3. The quality of the writing and clarity of some sentences could be improved.

Response:

Thank you for the suggestion. We have thoroughly revised the manuscript, and also followed the specific suggestions given by Reviewer#3 on writing part. In addition, our manuscript was proof read by a native English speaker.

4. This is the first time I've seen repeat content and heterozygosity summarised across such a

broad sample set, and I'd be interested in more interpretation of these results. Presumably you'd consider these to be extremely labile across plants? Given that lability, I wonder the suitability of summarising this across species within a family (as shown in the box plots)?

Response:

We have summarized the information on repeat content and heterozygosity for every individual, both in the result section as well in the Table S1.

Minor comments

5. Line 52. Next generation sequencing technologies?

Response: We have revised the sentence as "With the advent of next generation sequencing technologies, enormous efforts have been made to sequence whole genomes of plant species, thereby providing new insights on plant evolution [1]"

6. Line 81. Genome size and repeat content estimates are possible from genome skim data (for example, see RepeatExplorer), so this critique doesn't seem fair.

Response: Thank you for the suggestion. We do agree with your comment that "repeat content estimates are possible from the genome skim data via RepeatExplorer program". However, Genome size estimation is only possible for the plants with small genome size. Anyway, we have revised the sentence to avoid the confusion as follows:

"However, previous genome skimming studies have only generated a small amount of sequencing data for the individual species, precluding the re-use of the data to reveal more detailed genome features including genome sizes (for plant with large genome size), ploidy level etc., or its direct usage in the further *de novo* genome assembly."

7. Line 85: A major challenge for diverse genome sequencing projects like this one is choosing whether to sequence samples in accordance to their genome size, or whether to use a 'one size fits all' approach with similar amounts of data for all samples. It would be valuable to justify this approach, and say the range of coverage this amount of data generates (see main comment 1, above).

Response: It’s really a good question indeed. As you mentioned, it was a major question cum challenge for us to select the best sequencing approach for all the samples. In continuation to our response to the “comment 1”, this project is part of 10KP project, and being the starting point of this mega project, we wanted to first evaluate our existing sequencing strategy according to 'one size fits all' approach. On an average we generated 60X data per sample.

8. Line 106: The details of DNA extraction are very brief. Was the same DNA extraction protocol used for all species? Were there any modifications?

Response: We first optimized the CTAB DNA extraction protocol and found it suitable for most of the tested species. That’s the reason we used the same DNA extraction (Wu and Yang, 2018) method for all the species. The detailed protocol is available via GigaScience protocols.

Wu C and Yang T. DNA Extraction for plant samples by CTAB. GigaScience. 2018; doi:10.17504/protocols.io.pzqdp5w

9. Line 107: BGISEQ is not a very established sequencing platform relative to other platforms (such as Illumina). A brief description of this technology would be useful.

Response: BGISEQ-500 is a desktop sequencer developed by our Institute BGI-Shenzhen in 2015. Using DNA nanoball and combinational probe anchor synthesis developed from Complete Genomics™ sequencing technologies, it generates short reads at a large scale. The sequencing outputs are comparable with the Illumina series (Mak et al 2017), and has been successfully utilized to sequence the plant genome (Chang et al 2018), human genome (Huang et al 2017), and metagenomes (Fang et al 2017)

Mak, S. S. T., Gopalakrishnan, S., Carøe, C., Geng, C., Liu, S., Sinding, M. H. S., ... & Germonpré, M. (2017). Comparative performance of the BGISEQ-500 vs Illumina HiSeq2500 sequencing platforms for palaeogenomic sequencing. *GigaScience*, *6*(8), 1-13.

Huang, J., Liang, X., Xuan, Y., Geng, C., Li, Y., Lu, H., ... & Sun, N. (2017). A reference human genome dataset of the BGISEQ-500 sequencer. *Gigascience*, *6*(5), 1-9.

Fang, C., Zhong, H., Lin, Y., Chen, B., Han, M., Ren, H., ... & Stein, S. (2017). Assessment of the cPAS-based BGISEQ-500 platform for metagenomic sequencing. *GigaScience*, *7*(3), gix133.

10. Line 108: What type of libraries were prepared? What was the insert size?

Response: The 100bp pair end libraries were prepared for the sequencing, and the insert size was 200 bp.

11. Line 110. Repetition of 100bp PE from the previous line.

Response: We have now deleted the repetitive information.

12. Line 166: Define which repeats are being counted.

Response: We didn't assemble all the genomes, and doesn't used the RepeatExplorer program for our analysis. That's why we just used the Kmer data plot to identify the repeat ratio in the sequenced genomes. The specific repeat types will be classified in our subsequent manuscript, once we finish the assembly of all the genomes.

Line 183: It's unclear to me how heterozygosity was scored. Were reads mapped back to the initial assembly then heterozygous sites scored?

Response: The heterozygosity was scored based on the Kmer distribution plot according to the method described by Liu et al (2013). The same description is now mentioned in the main text also.

13. Line 255: Evolution of vasculature is not really relevant to this study.

Figure legends lack detail, e.g. details of the phylogenetic reconstruction used in Figure 2.

Response:

The ordinal phylogeny in Figure 2 was "drop-tips" from Figure 3.

14. Reference 4 seems incomplete.

Response: Thanks for your careful observation. We have now updated the reference for the book

chapter.

Reviewer #3: This manuscript reports the whole genome sequencing of more than 600 hundred vascular plant samples collected at the Ruili Botanical Garden in China. The data generated here makes an enormous contribution to the study of plant genomes of non-model plants by generating raw sequencing data, along with images, voucher information, complete chloroplast genomes and several partial nuclear genomes assembled. The supplementary data is essential to understand the scope of this project, and make processed data (i.e. genome assemblies) available. My main comments, suggestions and edits are included in the attached word document. They include a few questions to clarify the analyses performed, as well as suggestions to make the language clearer.

Response:

Thank you for your great suggestions and kind recommendation. We have thoroughly updated the manuscript (with track changes) as per your suggestions. Kindly refer the enclosed manuscript.
