# Supplementary material for: Molecular digitization of a botanical garden: high-depth whole-genome sequencing of 689 vascular plant species from the Ruili Botanical Garden
Source: Gigascience. 2019 Jan 25;8(4):giz007. doi: 10.1093/gigascience/giz007 (PMC6441391; doi:10.1093/gigascience/giz007)
Supplement: GIGA-D-18-00121_Revision_2.pdf [file giz007_giga-d-18-00121_revision_2.pdf]

GigaScience

Molecular Digitization of a Botanical Garden: High-depth whole genome sequencing of 689 vascular plants from the Ruili Botanical Garden

--Manuscript Draft--

Manuscript Number:	GIGA-D-18-00121R2
Full Title:	Molecular Digitization of a Botanical Garden: High-depth whole genome sequencing of 689 vascular plants from the Ruili Botanical Garden
Article Type:	Data Note

Funding Information:

the Shenzhen Municipal Government of china (JCYJ20150529150505656)	Dr. Xin Liu
the Shenzhen Municipal Government of China (JCYJ20150831201643396)	Dr. Yue Chang
The Construction of China National GeneBank (Yunnan GeneBank) (2015DA008)	Dr. Le Cheng
State Key Laboratory of Agricultural Genomics (2011DQ782025)	Dr. Huan Liu
Guangdong Provincial Key Laboratory of Genome Read and Write (2017B030301011)	Dr. Wangsheng Li

Abstract:

Background

Genome sequencing has been widely used in plant research to construct reference genomes and provide evolutionary insights. However, only a limited number of plant species have had their whole genome sequenced, thus further restraining the utility of these data.

Findings

Here, we comprehensively sampled and sequenced vascular plant species growing in the Ruili Botanical Garden, located in South West China. We sequenced 761 samples, out of the total of 1,093, and collected voucher specimens that were stored in the Herbarium of China National GeneBank (HCNGB). These 761 samples represented 689 vascular plant species from 134 families belonging to 47 orders. Of these, 254 samples were identified to species level and 506 samples were identified to families using chloroplast sequences. In total, we generated 54 Tb of sequencing data, which resulted in an average sequencing depth of 60X per species, as estimated from genome sizes. A reference phylogeny was reconstructed with 78 chloroplast genes for molecular identification and other possible applications.

Conclusions

In this study, we generated a large dataset of vascular plant genomes, with both high-depth whole genome sequencing data and associated voucher specimens, making it a valuable dataset for plant genome research and other applications. This project also provides insight into the feasibility and technical requirements for “planetary scale” projects such as the 10 thousand Plant Genome Project (10KP) and the Earth BioGenome Project (EBP).

Corresponding Author:	Xin Liu, Ph.D.<br>BGI<br>CHINA
Corresponding Author Secondary Information:	
Corresponding Author's Institution:	BGI

Powered by Editorial Manager® and ProduXion Manager® from Aries Systems Corporation

**Corresponding Author's Secondary Institution:**	
**First Author:**	Huan Liu
**First Author Secondary Information:**	
**Order of Authors:**	Huan Liu
	jinpu Wei
	Ting Yang
	Weixue Mu
	Bo Song
	Tuo Yang
	Yuan Fu
	Xuebin Wang
	Guohai Hu
	Wangsheng Li
	Hongcheng Zhou
	Yue Chang
	Xiaoli Chen
	Hongyun Chen
	Le Cheng
	Xuefei He
	Hechen Cai
	Xianchu Cai
	Mei Wang
	Yang Li
	Sunil Kumar Sahu, PhD.
	Jinlong Yang
	Yu Wang
	Ranchang Mu
	Jie Liu
	Jianming Zhao
	Ziheng Huang
	Xun Xu, PhD
	Xin Liu, Ph.D.
**Order of Authors Secondary Information:**	
**Response to Reviewers:**	Dear Dr. Scott,<br><br>We are glad to submit the thoroughly revised version of our manuscript entitled "Molecular Digitization of a Botanical Garden: High-depth whole genome sequencing of 689 vascular plants from the Ruili Botanical Garden".<br><br>The comments of the reviewers were highly insightful and enabled us to greatly improve the quality of our manuscript. According to their advice, we have carefully revised our manuscript.

Powered by Editorial Manager® and ProduXion Manager® from Aries Systems Corporation

We present the point-by-point responses to each of the comments and suggestions of the reviewer, we also made a substantial revision in the manuscript with track changes. However, we have uploaded both clean and track changes version for your kind perusal. We strongly believe that these revisions in the manuscript and our accompanying responses are sufficient to make our manuscript suitable for publication in GigaScience.

Reviewer reports:
Reviewer #1:
1)I reviewed the previous version of this submission and am pleased to see that most of the reviewers' comments have been used effectively. My only reservations would remain the low proportion of precise identifications of the samples used, and I trust that the available resources - images; specimens - will be sufficient to address this as the data are further processed and used.
Response: Your comments in the earlier version of the manuscript were really useful, and enabled us to greatly improve the quality of our manuscript. Yes, the genomics data generated in this study makes an enormous contribution to the study of plant genomes of non-model plants along with images, voucher information, complete chloroplast genomes and nuclear genomes. We agree with your concerns, but as mentioned in the manuscript, all the information will be continuously updated and linked to the GigaDB repository as new assemblies are completed.

2) I have been through the text and made a number of suggestions for minor revisions and a few comments which are included in a tracked changes version of the word document. I hope these will be useful and look forward to seeing this impressive piece of work published in due course.

Response:
Thank you for your great suggestions and kind recommendation. We have thoroughly updated the manuscript (with track changes) as per your suggestions. Kindly refer the enclosed manuscript.

Reviewer #2:
The authors have done the right thing and removed the Astral analysis, which was inappropriate for this data. Their new analysis is better, though personally I would raise the following questions:

1.It is very unusual that only 18 conserved genes are found across plastids. In general plastid gene content is highly conserved across plant species. I would suggest the few samples without a standard plastid gene complement are removed and the phylogeny rerun with as many loci as possible. Alternatively, more details could be given as to this issue, perhaps in the Supplementary Information. For example, is it actually gene loss, or are there issues aligning divergent loci?
Response：Yes, we do agree that the plastid gene content of green plant is highly conserved, but the gene loss was observed in several clades especially in parasitic plants and gymnosperm. For instance, in our study, Gnetales, Pinales lost all ndh and rps genes, Gnetaceae only contained 45 genes. To avoid the confusion, we have now changed the sentence “only 18 conserved genes are found across plastids” to “only 18 genes were found to be consistently present among all the plastid genomes”. The gene content information is listed in supplementary table 3.

2.A single model of molecular evolution is used across loci. It would be much better to use model testing on each locus and rerun the analysis using partitions.

Response: To refer the phylogeny of green plants, Brad et al. (2014) [1] used 360 plastid genomes and partitioned the nucleotide data into four data sets with the GTR+Γ model. They found that the average support values among all internal nodes in the ML trees were slightly higher in the ntAll phylogeny, and ntAll phylogeny also had the most clades resolved with ≥70% BS. In our study, we used IQ-TREE with best model GTR+F+R10 to construct the tree.
According to your advice, we partitioned all nt data set to 76 groups of sites based on

	gene content, IQ-TREE edge-linked-equal partition model. But, separate models between partitions were used with command -m “GTR+I+G”. However, to our surprise, the phylogenetic tree was the same as our previous result. Please see the enclosed phylogenetic tree for your kind perusal.<br><br>We look forward to hearing from you at your earliest convenience.<br>Yours sincerely,<br>Xin Liu
**Additional Information:**	
**Question**	**Response**
Are you submitting this manuscript to a special series or article collection?	No
**Experimental design and statistics**<br><br>Full details of the experimental design and statistical methods used should be given in the Methods section, as detailed in our Minimum Standards Reporting Checklist. Information essential to interpreting the data presented should be made available in the figure legends.<br><br>Have you included all the information requested in your manuscript?	Yes
**Resources**<br><br>A description of all resources used, including antibodies, cell lines, animals and software tools, with enough information to allow them to be uniquely identified, should be included in the Methods section. Authors are strongly encouraged to cite Research Resource Identifiers (RRIDs) for antibodies, model organisms and tools, where possible.<br><br>Have you included the information requested as detailed in our Minimum Standards Reporting Checklist?	Yes
**Availability of data and materials**<br><br>All datasets and code on which the conclusions of the paper rely must be either included in your submission or	Yes

Powered by Editorial Manager® and ProduXion Manager® from Aries Systems Corporation

deposited in publicly available repositories (where available and ethically appropriate), referencing such data using a unique identifier in the references and in the "Availability of Data and Materials" section of your manuscript.<br><br>Have you have met the above requirement as detailed in our Minimum Standards Reporting Checklist?	

Powered by Editorial Manager® and ProduXion Manager® from Aries Systems Corporation

Manuscript
Click here to access/download;Manuscript;GIGA-D-18-00121_clean_R1_clean_final.docx
Click here to view linked References

Molecular Digitization of a Botanical Garden: High-depth whole genome sequencing of 689 vascular plants from the Ruili Botanical Garden

Huan Liu[1,2,5*], Jinpu Wei[1,2*], Ting Yang[1,2,5*], Weixue Mu[1,2,5], Bo Song[1,2], Tuo Yang[1,2], Yuan Fu[1,2], Xuebing Wang[1,2], Guohai Hu[1,2], Wangsheng Li[1,2], Hongcheng Zhou[1,2], Yue Chang[1,2], Xiaoli Chen[1,2,5], Hongyun Chen[1,2,5], Le Cheng[3], Xuefei He[1,2], Hechen Cai[1,2], Xianchu Cai[1,2], Mei Wang[1,2,5], Yang Li[1,2], Sunil Kumar Sahu[1,2,5], Jinlong Yang[3], Yu Wang[3], Ranchang Mu[4], Jie Liu[4], Jianming Zhao[4], Ziheng Huang[1,2,5], Xun Xu[1,2,5], Xin Liu[1,2,5#].

Author Affiliations

1. BGI-Shenzhen, Shenzhen 518083, China
2. China National GeneBank, BGI-Shenzhen, Shenzhen 518120, China
3. BGI-Yunnan, BGI-Shenzhen, Kunming, 650106, China
4. Forestry Bureau of Ruili, Ruili, 678600, China
5. State Key Laboratory of Agricultural Genomics, BGI-Shenzhen, Shenzhen 518083, China

[*] These authors contributed equally to this work.

[#] To whom correspondence should be addressed: Xin Liu (liuxin@genomics.cn)

ORCID: Xin Liu: 0000-0003-3256-2940; Ting Yang: 0000-0002-2872-4954; Huan Liu: 0000-0002-6902-9931; Yue Chang: 0000-0003-3909-0931; Sunil Kumar Sahu: 0000-0002-4742-9870

Abstract

Background

Genome sequencing has been widely used in plant research to construct reference genomes and provide evolutionary insights. However, only a limited number of plant species have had their whole genome sequenced, thus further restraining the utility of these data.

Findings

Here, we comprehensively sampled and sequenced vascular plant species growing in the Ruili Botanical Garden, located in South West China. We sequenced 761 samples, out of the total of 1,093, and collected voucher specimens that were stored in the Herbarium of China National GeneBank (HCNGB). These 761 samples represented 689 vascular plant species from 134 families belonging to 47 orders. Of these, 254 samples were identified to species level and 506 samples were identified to families using chloroplast sequences. In total, we generated 54 Tb of sequencing data, which resulted in an average sequencing depth of 60X per species, as estimated from genome sizes. A reference phylogeny was reconstructed with 78 chloroplast genes for molecular identification and other possible applications.

Conclusions

In this study, we generated a large dataset of vascular plant genomes, with both high-depth whole genome sequencing data and associated voucher specimens, making it a valuable dataset for plant genome research and other applications. This project also

provides insight into the feasibility and technical requirements for "planetary scale" projects such as the 10 thousand Plant Genome Project (10KP) and the Earth BioGenome Project (EBP).

Keywords: Whole genome sequencing, Vascular plants, Phylogeny, Voucher specimens, Ruili Botanical Garden.

Background

With the advent of next generation sequencing technologies, enormous efforts have been made to sequence whole genomes of plant species, thereby providing new insights into plant evolution [1] and new information for improving agriculture yield and stress tolerance [2, 3]. As of November 2018, more than 350 land plant genomes have been sequenced (https://www.ncbi.nlm.nih.gov/genome), most of which are crops (57.7%), model species along with their closely-related species (22.3%), and wild relatives of crops (17.7%). However, with approximately 391,000 known species of plants [4], if we consider the evolutionary history and diversity of plants, the currently available sequence data is very limited. More recently, more than 1,000 plant species have had their transcriptome sequenced to better understand the evolution of plants, and thus also provide valuable resources for other plant research [5]. However, considering the high proportion of non-coding regions, whole genome sequencing data should be generated for further plant evolutionary studies. Thus, global efforts have been initiated to

sequence 10,000 plant genomes (10KP) [6] as a key part of the Earth BioGenome project (https://www.earthbiogenome.org/) [7]. For these large-scale whole genome sequencing efforts, we need to prove feasibility, as well as to set up technical standards for sampling, sequencing and data management.

Over the past decade, DNA barcoding has emerged as an important molecular tool for ecological studies, and specially for the rapid identification of standard specimens [8]. Although it is well-suited for studying historical specimen samples, considering the DNA degradation in those samples [9, 10], the major drawback of the technology is that DNA barcoding only provides limited genomic information, which is just based on small fragments of the nuclear or chloroplast genome [11]. In order to overcome this problem, genome skimming, which is whole genome sequencing by second-generation sequencing technologies, has been proposed [12] to provide more genome sequence information for better species identification [13, 14]. However, previous genome skimming studies have only generated a small amount of sequencing data for the individual species, precluding the re-use of the data to reveal more detailed genome features including genome sizes (for plants with large genomes), ploidy level etc., or its direct usage in the further de novo genome assembly. Here, we sequenced vascular plants genomes of 761 samples representing 689 vascular plant species at high depth (more than 60Gb on an average per sample). Making all of these data freely accessible and linked to their voucher details in the CNGB herbarium and Ruili Botanical Garden will provide new insights into the evolution of vascular plants and enable the data to be

utilized as a valuable genomic resource for evolution and diversity research and applications.

Data Description

Sampling, sequencing and data summary

In order to investigate the diversity of vascular plants in Ruili Botanical Garden and provide genome information for these species, we sampled almost all of the species growing at the Garden and sequenced them using BGISEQ-500 sequencing technology. BGISEQ-500 is a desktop sequencer developed by our Institute BGI-Shenzhen in 2015. Using DNA nanoball and combinational probe anchor synthesis developed from Complete Genomics™ sequencing technologies, it generates short reads at a large scale. The sequencing outputs are comparable with the Illumina series [15], and has been successfully utilized to sequence human genome [16], metagenomes [17] and variant identification [18]. The samples were collected from Ruili Botanical Garden, Yunnan, China (97°38′47″ to 98°05′57″ N, 23°52′42″ to 24°09′20″ E, ranging in altitude from 738 m to 1,200 m above the sea level, as shown in Figure 1). In total, we collected 1,093 vascular plant samples, from which we used the young leaves for DNA extraction. The voucher specimens and images were also collected for these samples. All the specimens are stored in the Herbarium, of China National GeneBank (HCNGB), and voucher information can be found in Table S1 (Additional files). The collected young leaves were shipped to Shenzhen on dry ice, and DNA was extracted using the CTAB method [19]. We were successful at extracting good quality DNA from 761 samples.

Whole genome sequencing libraries were constructed for each of these samples according to BGISEQ-500 manufacturer instructions, and then sequenced [20]. Approximately 70 Gb of raw sequencing data (100 bp, paired-end) was generated for each of these samples (Table 1). Raw reads were filtered using SOAPfilter_v2.2 with the following parameters: -y –p –i 180 –M 2 –Q 10. After filtering the low-quality reads (reads with more than 10% Ns, ambiguous bases; reads with more than 40% bases having quality lower than 10; reads contaminated by adaptors or PCR duplicates), ~60 Gb of clean data (high-quality reads >Q35) were obtained for each of these samples.

Species identification and phylogenetic relationship

The taxonomic identification of specimens is a time-consuming process and requires expertise and experience. In this study, covering the majority of extant vascular plant lineages, the collections were difficult to identify to species level in a short time. We were able to identify 254 samples to species level (for a total of 232 unique species) using the specimen morphology and the other 506 samples were identified to families using their chloroplast sequences. Thus, in total, we identified 689 samples from those 761 sequenced, which belonged to 134 families and 47 orders. Among these families, the majority of the species belonged to Fabaceae (71 taxa), Poaceae (45 taxa) and Asteraceae (38 taxa), respectively. We assembled the chloroplast genomes of each species from clean read data using NOVOPlasty [21], a seed-extension-based de novo assembler. We used the complete cds rbcL gene sequence of *Arabidopsis thaliana* (downloaded from NCBI, accession number: U91966) as the seed to conduct the

assembly. The NOVOPlasty assembly recovered complete chloroplast genomes of 50 species in a single circular sequence. For the remaining species, the longest contig assembled by NOVOPlasty was BLASTed against the chloroplast database (downloaded from NCBI, including 2,503 non-redundant species) and the resulted best-hit sequences (minimum requirement: e-value < 10-7 and identity > 95%) were used as references for further assembly using MITObim [22]. In this way, we finally recovered complete chloroplast genomes for all 689 species. The assembled chloroplast genomes ranged from 113,621 to 183,602 bp in size (Table S2). We then annotated the assembled chloroplast genomes using DOGMA [23] and GeneWise [24], and we found 72 protein-coding genes in almost all of these vascular plant families except Gnetaceae, Malvaceae, Elaeocarpaceae, and Tectariaceae. For Gnetaceae, we were only able to annotate 52 protein-coding genes in their chloroplast genomes, which is consistent with previous studies [25]. We then compared these assembled chloroplast genomes and constructed the phylogenetic tree using RAxML [26] and IQ_TREE [27]. A total of 78 individual coding genes were identified from 738 samples, the majority of which were present in 710 to 738 samples (on average). However, only 18 genes were found to be consistently present among all the plastid genomes, Gnetales and Pinales nearly lost all ndh and rps genes (Table S3). Each gene was aligned using MAFFT [28] and every alignment was then processed with TrimAL [29] using the gappyout option to remove poorly aligned positions. Then gene alignments were combined, which resulted in 59695 nucleotide positions. Maximum likelihood (ML) species trees were constructed using the RAxML

package (v8.2.4) with the GTRCAT model, 1,000 bootstrap replicates, a random seed number:123456 were selected for the parsimony inferences and 26 fern samples were used to root the tree. ML analyses were also performed with IQ-TREE under the substitution model GTR+F+R10 which was determined according to the Akaike information criterion (AIC) and the Bayesian information criterion (BIC) by IQ-TREE. With the increase in amount of phylogenetic data, it has become increasingly important to choose different substitution model for variation in rates and patterns of substitution among sites. We partitioned 59,695 nucleotide positions to 76 groups of sites based on the gene content, then edge-linked-equal partition model. But, separate models between partitions were used with the parameter: -m "GTR+I+G" by IQ-TREE (named as IQ-TREE partitions). Both RAxML and IQ_TREE provided consistent phylogenetic reconstructions (Figure 2 and Figure S1). All nodes in the phylogenetic tree with partitioning scheme were same with no partitioning scheme in IQ-TREE. The major lineages can be observed within Fabales, Rosales, Poales and Malpighiales. In Fabids, Celastrales was the sister group to Malpighiales other than Oxalidales in this study (BS=100%). For Petrosaviidae, the major ordinal relationship was consistent with the previous research, as well as for Liliales, Asparagales, Poales, Arecales, Commelinales, Pandanales, and Zingiberales; the earliest-branching lineage is Alismatales [30]. Relationships between Gentianales, Lamiales and Solanales remained unclear [31, 32]. In our study, the ML tree provided support for Gentianales sister to Lamiales (BS=83%) with sister group to Solanales and Boraginales (BS=100%). We also included 54

species of Poales in the analyses, which revealed its close relationship with Arecales rather than Pandanales and Dioscoreales.

Genome size, repeat content, and heterozygosity

In order to ensure the quality and effectiveness of the dataset (Table 1), we conducted several analyses to reveal the basic genomic features of the vascular plants sampled. By using GCE [33] and kmergenie [34] software and the clean data of each species, we estimated the genome sizes, repeat content and heterozygosity (Figure 3 and Table S1). For several of these tested species, the genome sizes have been previously measured by experimental approaches and are publicly available (http://data.kew.org/cvalues/) (Table S4). We compared the previous estimations to the genome sizes estimated by k-mer analysis in this study, and found good agreement between them (R2=0.63) (Figure S2). We found that despite overall wide variation in the genome sizes of these plants, most of the families had relatively comparable genome sizes. The most diverse family in terms of genome size was found to be Cupressaceae, in which genome sizes ranged from 0.18 Gb in *Cunninghamia lanceolata* (Lamb.) Hook. var. *lanceolata* to 19.26 Gb in *Juniperus pingii* var. *wilsonii* (Rehder) Silba. In addition, repeat content varied from 10% to 88% on average among species sampled, with several exceptions in Cornaceae, Myrtaceae and Celastraceae. For instance, Myrtaceae (Myrtales) were found to have the most repetitive genomes (~88% of repetitive content), while Celastraceae (Celastrales) waere found to have the least repetitive genomes (~10% repetitive content). We also found relatively high heterozygosity in these species ranging from

0.15% to 36.6% per individual, which probably reflected their nature as wild species.

Genome assemblies

Despite the limitation of having only one sequencing library constructed for each species, we were able to conduct preliminary genome assemblies for many of them, which reflected the quality and reuse potential of the data. Based on the estimated heterozygosity and repeat content, we initially selected 17 species from 17 families with relatively simple genome content (heterozygosity rate less than 1% and repeat content less than 50%) for genome assembly. We used SOAPdenovo2 [35] (parameters: pregraph-K 35 contig -M 1 scaff). We obtained an average contig N50 of 4.62 kb, and an average scaffold N50 of 32.2 kb for these genome assemblies. Two species *Alternanthera sessilis* (L.) R.Br. ex DC. and *Senna alata* (L.) Roxb., were assembled to contig N50 of 15.2 kb, scaffold N50 of 95.5 kb and contig N50 of 14 kb and scaffold N50 of 101.1 kb respectively (Table S5). We then carried out Benchmarking Universal Single-Copy Orthologs (BUSCO) (version 3.0.1) analysis [36] to find the completeness of all these 17 genome assemblies. On average, genome completeness was found to be ~89.1%, 1,243 BUSCOs were complete and single-copy and 40 BUSCOs were complete and duplicated (from a total of 1,440 BUSCOs). The average numbers of fragmented and missing BUSCOs were 55 and 101, respectively (Table S6). Our preliminary assemblies were of good quality, providing a useful reference for future efforts to establish complete reference genomes for all these plant species. In addition to the current attempt of genome assembly, continuing efforts are being carried out to

finish the preliminary assemblies of the other species and these are being deposited and linked with existing already public sequencing data.

Data access and reuse potential

The data generated here includes images, raw sequencing data, assembled chloroplast genomes, and preliminary nuclear genome assemblies. All the data have been organized and linked to a top-level accession in the GigaScience GigaDB repository (http://doi.org/10.5524/100502), containing the lists of all the species and the links to each species page. In addition, each species has a DOI assigned to them containing information on collection number, an image of the plant during sampling, SRA accession number for the raw data, a data file containing the assembled chloroplast genome sequence in FASTA format (these chloroplast sequences can also be found in Table S2), a data file containing the preliminary assembled nuclear genome sequence (available only for some species and will continue to be updated when each assembly is completed). Voucher specimens are stored in the Herbarium of China National GeneBank (HCNGB), and digitized images for every sheet are also being made available in GigaDB alongside the sequencing data. The data reported in this study are also available in the CNGB Nucleotide Sequence Archive (CNSA: https://db.cngb.org/cnsa ; accession number CNPhis0000538). All raw data are also deposited in the NCBI SRA repository under the project number PRJNA43840. In addition to the description in SRA, the SRA accession number of raw data is also included in the GigaDB entries, thus the raw data of specific species can be traced from

GigaDB. Datacite and GigaDB (http://doi.org/10.5524/100502) metadata are all linked, and any future updates made on the GigaDB dataset provides traceable records.

The high-depth whole genome sequencing data together with images and voucher specimens can be reused in different ways and will be valuable for future applications. First of all, in addition to the phylogenetic analysis carried out here using the assembled chloroplast genomes, future evolutionary analysis can be carried out to study the evolution of specific genes after assembling them from raw reads, as well as investigating particular features of plant genome evolution including evolution of repeats, polyploidization, whole genome duplication, etc. Secondly, the data can be used to improve future genome assemblies of these plant species. For example, utilizing the information on repeat content, heterozygosity and genome size estimation provided here to tailor new sequencing and genome assembly strategies of these plant genomes, as well as integrating the sequencing data itself in other genome assemblies. By directly using the sequencing data obtained from this study, it would be easier and more efficient to assemble the remaining sequenced plant genomes. The ~60 Gb data can be used for genome assembly in combination with either contig reconstruction of the second generation-based sequence reads, or for error correction of the third-generation long sequence reads. Last but not least, this dataset can also be used for developing new methods for species identification either based on sequencing data or based on images of plants and to resolve phylogenetic relationships based on whole genome sequencing data, among others. At present, we do not have enough information to identify all the

species, so we are building a living plant database which records the position of Ruili species and monitor the status of each species, the database can be visited through the URL http://720yunnan.com/tour/a2b8096d43d7226d?scene=scene_d3627cc2a43314d8. Deep learning could also be applied to develop plant identification using this dataset as a good training set in combination with other accumulating information in future. We used 175 known species from Ruili data for deep learning, every sample extracted 1M reads to build the model. At the first trial stage, 181 species have been successfully identified to species level using our models. Providing this comprehensive dataset which can be easily accessed by researchers and also the general public, we believe it would be reused in many ways beyond what has been mentioned here.

Discussion

The current understanding of the evolution of plants and its diversity in a phylogenomic context is limited due to the lack of genome-scale information across phylogenetically diverse species. In this study, we provide a dataset of high-depth whole genome sequencing of 689 vascular plant species with voucher specimens, covering 134 families and 47 orders. These samples were obtained from Ruili Botanical Garden in Yunnan Province of China, near the border between China and Myanmar, reflecting the rich plant diversity in that region. The high-depth whole genome sequencing data generated here have been used to estimate genomic features including genome size, repeat content and heterozygosity, which can provide guidance to future studies aiming

at establishing reference genomes for these species. The high-depth whole genome data can also be used in assembling chloroplast genomes, as well as some conserved nuclear genes, thus providing useful information for evolution and gene function studies.

In this study, we scaled up whole genome sequencing effort to sequence hundreds of plant species. We only constructed a single short insert library (200 bp) for each of the species and generated ~60 Gb of whole genome sequencing data. It would be insufficient to assemble high-quality genomes for the majority of the species just based on single library data, however, the current data could potentially be used for several analyses such as such as gene finder, plastid and mitochondrial assembly. At present, we are using these data in combination with 10x genomics to get high quality genome data. In addition, our study tested for the first time, the feasibility of large-scale whole genome sequencing, which is already underway for the Earth BioGenome Project (EBP) [7] and 10 thousand Plant Genome Projects (10KP)[6]. This study provided experience for plant sampling, sample logistics and management, DNA extraction, sequencing library preparation, sequencing and data analysis and management. Aiming at sequencing more than 10,000 plant species, 10KP requires the establishment of a robust infrastructure for sample and data management, as potentially investigated in this pilot study. Now, we have optimized the DNA extraction protocol and published the protocol [19]. Soon we will launch the DNA extraction kit. We also have just finished a guideline about sample submission for 10KP which includes sample preparation (fresh sample, DNA sample and RNA sample), sample packing and shipping. The specific guidelines

will be soon available in our 10KP website (https://db.cngb.org/10kp/).

Availability of Supporting Data

The specimens, leaf samples and DNA solutions of all collections are stored at the China National GeneBank (CNGB) Herbarium. The raw sequencing data described in this article are available in the NCBI SRA repository, under the project number PRJNA43840. DNA Extraction [19] and BGISEQ-500 WGS library construction protocols can be found in protocols.io [20]. A total of 738 chloroplast genomes and 17 assembled genomes together with raw data supporting the results of this article are available via the GigaDB repository of GigaScience, and will be continuously updated and linked to the GigaDB entries as new assemblies are completed.

Additional files

Additional file 1

Table S1. List of samples included in this study with voucher information, current kmer based estimation of genome sizes, repeat content and heterozygosity. Identified collections were listed with species names, while unidentified ones with only family and order information. 738 samples with assembled chloroplast genome were marked with *, whereas 17 samples with assembled unclear genomes were marked with §.

Table S2. All the assembled chloroplast genomes and their lengths.

Table S3. The gene content information for all the assembled chloroplast genomes.

Table S4. Genome information previously measured and publicly available on the

database.

Table S5. Summary of preliminary genome assemblies of 17 species of vascular plants families.

Table S6. Summary of BUSCO analysis for 17 species of vascular plant families.

Additional file 2

Figure S1. Phylogeny of vascular plants of the Ruili Botanical Garden. Species tree based on 78 the ML analysis of chloroplast genes generated by RAxML. Colors of the inner circle and outer circle represent different families and orders. The clade color represents bootstrap values from red to gray (bootstrap ranges from 50 to 100).

Figure S2. A comparison of genome sizes measured by experimental approaches to the k-mer estimated genome sizes in this study.

Abbreviations

10KP: 10 thousand Plant Genome Project

bp: base pair

BUSCO: Benchmarking Universal Single-Copy Orthologs.

EBP: Earth BioGenome Project.

Gb: Gigabase

HCNGB: Herbarium, China National GeneBank.

ML: Maximum likelihood.

WGS: Whole Genome Sequencing.

Competing interests

All authors declare that they have no competing interests.

Funding

This work was supported by grants of Basic Research Program, the Shenzhen Municipal Government, China (No. JCYJ20150529150505656) and (No. JCYJ20150831201643396), as well as funding from Guangdong Provincial Key Laboratory of Genome Read and Write（No.2017B030301011）, and The Construction of China National GeneBank (Yunnan GeneBank) (Yunnan province, 2015DA008, P.R. China).

Author contributions

XL conceived this study. XL and HL drafted the manuscript. HL managed the project. JPW, XBW, LC, XFH, HCC, JLY, YW, RCM, JL, JMZ collected the samples. TY lead identification of voucher specimens. TY, WXM, BS, YF, YC, HYC analyzed the data. TY, XLC, MW, ZHH constructed the phylogenetic tree. GHH, WSL, HCZ, HCC, YL extracted DNA and performed genome sequencing. SKS and XX revised and edited the manuscript. All the authors have read and approved the final manuscript.

Acknowledgments

The authors would like to express their sincere thanks to the local people and Government of Yunnan province, and the Forestry Institute of Dehong Prefecture for their kind help in sample collections. We would also like to thank the taxonomic experts in PE (Herbarium, Institute of Botany, Chinese Academy of Sciences) for identification. Finally, we are thankful to the production team of China National GeneBank, Shenzhen, China.

References

1. Pennisi E. Plant biology. Green genomes. Science. 2011;332 6036:1372-5. doi:10.1126/science.332.6036.1372.
2. Bolger ME, Weisshaar B, Scholz U, Stein N, Usadel B and Mayer KF. Plant genome sequencing - applications for crop improvement. Curr Opin Biotechnol. 2014;26:31-7. doi:10.1016/j.copbio.2013.08.019.
3. Desta ZA and Ortiz R. Genomic selection: genome-wide prediction in plant improvement. Trends Plant Sci. 2014;19 9:592-601. doi:10.1016/j.tplants.2014.05.006.
4. Kew RBG. The state of the world's plants report–2016. Royal Botanic Gardens, Kew. 2016.
5. Matasci N, Hung L-H, Yan Z, Carpenter EJ, Wickett NJ, Mirarab S, et al. Data access for the 1,000 Plants (1KP) project. Gigascience. 2014;3 1:17.
6. Cheng S, Melkonian M, Smith SA, Brockington S, Archibald JM, Delaux P-M, et al. 10KP: A phylodiverse genome sequencing plan. Gigascience. 2018;7 3:giy013.
7. Lewin HA, Robinson GE, Kress WJ, Baker WJ, Coddington J, Crandall KA, et al. Earth BioGenome Project: Sequencing life for the future of life. Proc Natl Acad Sci U S A. 2018;115 17:4325-33.

8. de Vere N, Rich TC, Trinder SA and Long C. DNA barcoding for plants. Methods Mol Biol. 2015;1245:101-18. doi:10.1007/978-1-4939-1966-6_8.

9. Staats M, Erkens RH, van de Vossenberg B, Wieringa JJ, Kraaijeveld K, Stielow B, et al. Genomic treasure troves: complete genome sequencing of herbarium and insect museum specimens. PLoS One. 2013;8 7:e69189. doi:10.1371/journal.pone.0069189.

10. Osmundson TW, Robert VA, Schoch CL, Baker LJ, Smith A, Robich G, et al. Filling gaps in biodiversity knowledge for macrofungi: contributions and assessment of an herbarium collection DNA barcode sequencing project. PLoS One. 2013;8 4:e62419. doi:10.1371/journal.pone.0062419.

11. Li X, Yang Y, Henry RJ, Rossetto M, Wang Y and Chen S. Plant DNA barcoding: from gene to genome. Biol Rev Camb Philos Soc. 2015;90 1:157-66. doi:10.1111/brv.12104.

12. Straub SC, Parks M, Weitemier K, Fishbein M, Cronn RC and Liston A. Navigating the tip of the genomic iceberg: Next-generation sequencing for plant systematics. Am J Bot. 2012;99 2:349-64. doi:10.3732/ajb.1100335.

13. Male PJ, Bardon L, Besnard G, Coissac E, Delsuc F, Engel J, et al. Genome skimming by shotgun sequencing helps resolve the phylogeny of a pantropical tree family. Mol Ecol Resour. 2014;14 5:966-75. doi:10.1111/1755-0998.12246.

14. Besnard G, Christin PA, Male PJ, Coissac E, Ralimanana H and Vorontsova MS. Phylogenomics and taxonomy of Lecomtelleae (Poaceae), an isolated panicoid lineage from Madagascar. Ann Bot. 2013;112 6:1057-66. doi:10.1093/aob/mct174.

15. Mak SST, Gopalakrishnan S, Carøe C, Geng C, Liu S, Sinding M-HS, et al. Comparative performance of the BGISEQ-500 vs Illumina HiSeq2500 sequencing platforms for palaeogenomic sequencing. Gigascience. 2017;6 8:1-13.

16. Huang J, Liang X, Xuan Y, Geng C, Li Y, Lu H, et al. A reference human genome dataset of the BGISEQ-500 sequencer. Gigascience. 2017;6 5:1-9.

17. Fang C, Zhong H, Lin Y, Chen B, Han M, Ren H, et al. Assessment of the cPAS-based BGISEQ-500 platform for metagenomic sequencing. Gigascience. 2017;7 3:gix133.

18. Patch A-M, Nones K, Kazakoff SH, Newell F, Wood S, Leonard C, et al. Germline and somatic variant identification using BGISEQ-500 and HiSeq X Ten whole genome sequencing. PloS one. 2018;13 1:e0190264.

19. Wu C and Yang T. DNA Extraction for plant samples by CTAB. Gigascience. 2018; doi:10.17504/protocols.io.pzqdp5w.

20. Gao S, Mu F, Yang Z, Liu X, Jiang H, Liao S, et al. BGISEQ-500 WGS library construction. 2018; doi:10.17504/protocols.io.ps5dng6.

21. Dierckxsens N, Mardulyn P and Smits G. NOVOPlasty: de novo assembly of organelle genomes from whole genome data. Nucleic acids research. 2016;45 4:e18-e.

22. Hahn C, Bachmann L and Chevreux B. Reconstructing mitochondrial genomes directly from genomic next-generation sequencing reads—a baiting and iterative mapping approach. Nucleic acids research. 2013;41 13:e129-e.

23. Wyman SK, Jansen RK and Boore JL. Automatic annotation of organellar genomes with DOGMA. Bioinformatics. 2004;20 17:3252-5. doi:10.1093/bioinformatics/bth352.

24. Birney E, Clamp M and Durbin R. GeneWise and Genomewise. Genome Res. 2004;14 5:988-95. doi:10.1101/gr.1865504.

25. Hsu CY, Wu CS, Surveswaran S and Chaw SM. The complete plastome sequence of Gnetum ula (Gnetales: Gnetaceae). Mitochondrial DNA A DNA Mapp Seq Anal. 2016;27 5:3721-2. doi:10.3109/19401736.2015.1079874.

26. Stamatakis A. RAxML version 8: a tool for phylogenetic analysis and post-analysis of large phylogenies. Bioinformatics. 2014;30 9:1312-3.

27. Nguyen L-T, Schmidt HA, von Haeseler A and Minh BQ. IQ-TREE: a fast and effective stochastic algorithm for estimating maximum-likelihood phylogenies. Molecular biology and evolution. 2014;32 1:268-74.

28. Katoh K, Misawa K, Kuma K and Miyata T. MAFFT: a novel method for rapid multiple sequence alignment based on fast Fourier transform. Nucleic Acids Res. 2002;30 14:3059-66.

29. Capella-Gutiérrez S, Silla-Martínez JM and Gabaldón T. trimAl: a tool for automated alignment trimming in large-scale phylogenetic analyses. Bioinformatics. 2009;25 15:1972-3.

30. Chase MW. Monocot relationships: an overview. Am J Bot. 2004;91 10:1645-55. doi:10.3732/ajb.91.10.1645.

31. Bremer K, Backlund A, Sennblad B, Swenson U, Andreasen K, Hjertson M, et al. A phylogenetic analysis of 100+ genera and 50+ families of euasterids based on morphological and molecular data with notes on possible higher level morphological synapomorphies. Plant Systematics and Evolution. 2001;229 3-4:137-69.

32. Refulio‐Rodriguez NF and Olmstead RG. Phylogeny of lamiidae. American Journal of Botany. 2014;101 2:287-99.

33. Liu B SY, Yuan J, Hu X, Zhang H, Li N, Li Z, Chen Y, Mu D, Fan W. Estimation of genomic characteristics by analyzing k-mer frequency in de novo genome projects. arXiv preprint. 2013; doi:arXiv:1308.2012.

34. Chikhi R and Medvedev P. Informed and automated k-mer size selection for genome assembly. Bioinformatics. 2014;30 1:31-7. doi:10.1093/bioinformatics/btt310.

35. Luo R, Liu B, Xie Y, Li Z, Huang W, Yuan J, et al. SOAPdenovo2: an empirically improved memory-efficient short-read de novo assembler. Gigascience. 2012;1 1:18.

36. Simão FA, Waterhouse RM, Ioannidis P, Kriventseva EV and Zdobnov EM. BUSCO: assessing genome assembly and annotation completeness with single-copy orthologs. Bioinformatics. 2015;31 19:3210-2.

Figure legends

Figure 1. Sampling localities of this project. Sampling was conducted mainly in Ruili Botanical Garden in Southwest China, near the China-Myanmar border, and shown in red circles.

Figure 2. Phylogeny of vascular plants of the Ruili Botanical Garden based on the ML analysis tree of 78 chloroplast genes. inner circle, colors represent different families and color in outer circle represent different orders.

Figure 3. The ordinal phylogeny is based on "drop-tips" from Figure 2. Based on species phylogenetic tree, we used drop.tip function in ape package (5.2) to remove the corresponding internal branches. (a) Genome sizes in G, (b) repeat content as percentage of total genome (%), (c) heterozygosity ratio Cladogram was generated from the 78 chloroplast gene ML phylogeny using only one tip per orders.

Tables

Table 1 Summary of the sequencing data produced in this study.

Manuscript with track changes

Click here to view linked References

Click here to access/download;Manuscript;GIGA-D-18-00121_clean_R1_with track changes.docx

Molecular Digitization of a Botanical Garden: High-depth whole genome sequencing of 689 vascular plants from the Ruili Botanical Garden

Huan Liu[1,2,5*], Jinpu Wei[1,2*], Ting Yang[1,2,5*], Weixue Mu[1,2,5], Bo Song[1,2], Tuo Yang[1,2], Yuan Fu[1,2], Xuebing Wang[1,2], Guohai Hu[1,2], Wangsheng Li[1,2], Hongcheng Zhou[1,2], Yue Chang[1,2], Xiaoli Chen[1,2,5], Hongyun Chen[1,2,5], Le Cheng[3], Xuefei He[1,2], Hechen Cai[1,2], Xianchu Cai[1,2], Mei Wang[1,2,5], Yang Li[1,2], Sunil Kumar Sahu[1,2,5], Jinlong Yang[3], Yu Wang[3], Ranchang Mu[4], Jie Liu[4], Jianming Zhao[4], Ziheng Huang[1,2,5], Xun Xu[1,2], Xin Liu[1,2,5#].

Author Affiliations

1. BGI-Shenzhen, Shenzhen 518083, China
2. China National GeneBank, BGI-Shenzhen, Shenzhen 518120, China
3. BGI-Yunnan, BGI-Shenzhen, Kunming, 650106, China
4. Forestry Bureau of Ruili, Ruili, 678600, China
5. State Key Laboratory of Agricultural Genomics, BGI-Shenzhen, Shenzhen 518083, China

[*] These authors contributed equally to this work.

[#] To whom correspondence should be addressed: Xin Liu (liuxin@genomics.cn)

Abstract

Background

Genome sequencing has been widely used in plant research to construct reference genomes and ~~elucidate~~ provide evolutionary insights. However, only a limited number of plant species have had their whole genome sequenced, ~~and the limited taxon~~omic ~~breadth~~ ~~information of these species has~~thus further restrain~~ed~~ing the utility of these data.

Findings

Here, we comprehensively sampled and sequenced vascular plant species ~~of~~ growing in the Ruili Botanical Garden, located in South West China. We sequenced 761 samples, out of the total of 1,093, and collected voucher specimens that were stored in the Herbarium of China National GeneBank (HCNGB). These 761 samples represented 689 vascular plant species from 134 families belonging to 47 orders. Of these, 254 samples were identified to species level and 506 samples were identified to families using chloroplast sequences. In total, we generated 54 Tb of sequencing data, which resulted in an average sequencing depth of 60X per species, as estimated ~~by the~~from genome sizes. –A reference phylogeny was reconstructed with 78 chloroplast genes for molecular identification and other possible applications.

Conclusions

In this study, we generated a large dataset of vascular plant genomes, with both high-depth whole genome sequencing data and associated voucher specimens, making it a

valuable dataset for plant genome research and other applications. This project also provides insight into the feasibility and technical requirements for "planetary scale" projects such as the 10 thousand Plant Genome Project (10KP) and the Earth BioGenome Project (EBP).

Keywords: Whole genome sequencing, Vascular plants, Phylogeny, Voucher specimens, Ruili Botanical Garden.

Background

With the advent of next generation sequencing technologies, enormous efforts have been made to sequence whole genomes of plant species, thereby providing new insights ~~on~~ into plant evolution [1] and new information for improving agriculture yield and stress tolerance [2, 3]. As of ~~September~~ November 2018, more than 350 land plant~~'s~~ genomes ~~has~~ have been sequenced (https://www.ncbi.nlm.nih.gov/genome), most of which are crops (57.7%), model species along with their closely-related species (22.3%), and wild relatives of crops (17.7%). However, with approximately 391,000 known species of plants [4], if we consider the evolutionary history and diversity of plants, the currently available sequenc~~eing~~ data is very limited. More recently, more than 1,000 plant species have ~~been~~ had their transcriptome sequenced ~~at the transcriptome~~ to ~~reveal~~ better understand the evolution of plants, and thus also provide valuable resources for other plant research [5]. However, considering the ~~enormous gap~~

~~areas outside of the~~ high proportion of non-coding regions, whole genome sequencing data should be generated for further plant evolutionary studies. Thus, global efforts have been initiated to sequence 10,000 plant genomes (10KP) [6] as a key part of the https://www.earthbiogenome.org/ (EBP)[7]. For these large-scale whole genome sequencing efforts, we need to prove ~~the~~ feasibility, as well as to set up technical standards for sampling, sequencing and data management.

Formatted: No underline

Formatted: No underline

Over the past decade, DNA barcoding has emerged as an important molecular tool for ecological studies, and specially for the rapid identification of ~~non routine~~ standard specimens [8]. Although it is well-suited for studying historical specimen samples, considering the DNA degradation in those samples [9, 10], the major drawback of the technology is that DNA barcoding only provides limited genomic information, which is just based on ~~the~~ small fragments of the nuclear or chloroplast genome [11]. In order to overcome this problem, genome skimming, which is whole genome sequencing by second-generation sequencing technologies, has been proposed [12] to provide more genome sequence information for better species identification [13, 14]. However, previous genome skimming studies have only generated a small amount of sequencing data for the individual species, precluding the re-use of the data to reveal more detailed genome features including genome sizes (for ~~a~~ plants with large genomes ~~size~~), ploidy level etc., or its direct usage in the further de novo genome assembly. Here, we sequenced vascular plants genomes of 761 samples representing 689 vascular plant species at high depth (more than 60Gb on an average per sample). Making all of these

data freely accessible and linked to their voucher details in the CNGB herbarium and Ruili Botanical Garden will provide new insights into the evolution of vascular plants and enable ~~it~~ the data to be utilized as a valuable genomic resource for evolution and diversity research and applications.

Data Description

Sampling, sequencing and data summary

In order to investigate the diversity of vascular plants in Ruili Botanical Garden and provide genome information for these species, we sampled almost all of the ~~vascular plant~~ species growing at the ~~in Ruili~~ Garden and sequenced them using BGISEQ-500 sequencing technology. BGISEQ-500 is a desktop sequencer developed by our Institute BGI-Shenzhen in 2015. Using DNA nanoball and combinational probe anchor synthesis developed from Complete Genomics™ sequencing technologies, it generates short reads at a large scale. The sequencing outputs are comparable with the Illumina series [15], and has been successfully utilized to sequence human genome [16], metagenomes [17] and variant identification [18]. The~~se~~ samples were collected from Ruili Botanical Garden, Yunnan, China (97°38′47″ to 98°05′57″ N, 23°52′42″ to 24°09′20″ E, ranging in altitude from 738 m to 1,200 m above the sea level, as shown in Figure 1). In total, we collected 1,093 vascular plant samples, from which we used the young leaves for DNA extraction. The~~V~~ voucher specimens and images were also collected for these samples. All the specimens are stored in the Herbarium, of China National GeneBank (HCNGB), and voucher information can be found in Table S1

(Additional files). The collected young leaves were shipped to Shenzhen on dry ice, and DNA was extracted using the CTAB method [19]. We were successful at extracting ~~enough~~ good quality DNA ~~for~~ from 761 ~~of those~~ samples. W~~w~~hole genome sequencing libraries were constructed for each of these samples according to BGISEQ-500 manufacturer instructions, and then sequenced [20]. Approximately 70 Gb of raw sequencing data (100 bp, paired-end) was generated for each of these samples (Table 1). Raw reads were filtered using SOAPfilter_v2.2 ~~by~~ with the following ~~the command~~parameters: -y –p –i 180 –M 2 –Q 10. After filtering the low-quality reads (reads with more than 10% Ns, ambiguous bases; reads with more than 40% bases having quality lower than 10; reads contaminated by adaptors or PCR duplicates), ~60 Gb of clean data (high-quality reads >Q35) were obtained for each of these samples ~~and data showed high-quality reads (>Q35)~~.

Formatted: Font color: Blue, Pattern: Clear (Custom Color(RGB(239,242,247)))

Species identification and phylogenetic relationship

The taxonomic identification of specimens is a time-consuming process and requires expertise and experience. In this study, ~~the collections~~ covering the majority of extant vascular plant lineages, the collections were difficult to identify to species level in a short time. We were able to identify 254 samples to species level (~~from~~ for a total of 232 unique species) using the specimen morphology and the other 506 samples were identified to families using their chloroplast sequences. Thus, in total, we identified 689 samples from those 761 sequenced, which belonged to 134 families and 47 orders. Among these families, the majority of the species belonged to Fabaceae (71 taxa),

Poaceae (45 taxa) and Asteraceae (38 taxa), respectively. We assembled the chloroplast genomes of each species from clean read data using NOVOPlasty [21], a seed-extension-based de novo assembler. We used the complete cds rbcL gene sequence of *Arabidopsis thaliana* (downloaded from NCBI, accession number: U91966) as the seed to conduct the assembly. The NOVOPlasty assembly recovered complete chloroplast genomes of 50 species in a single circular sequence. For the remaining species, the longest contig assembled by NOVOPlasty was BLASTed against the chloroplast database (downloaded from NCBI, including 2,503 non-redundant species) and the resulted best-hit sequences (minimum requirement: e-value < 10-7 and identity **>** 95%) were used as references for further assembly using MITObim ~~[18],~~ [22]. ~~in~~ In this way, we finally recovered complete chloroplast genomes for all 689 species. The assembled chloroplast genomes ranged from 113,621 to 183,602 bp in size (Table S2). We then annotated the assembled chloroplast genomes using DOGMA [23] and GeneWise [24], and we found 72 protein-coding genes in almost all of these vascular plant families except Gnetaceae, Malvaceae, Elaeocarpaceae, and Tectariaceae. For Gnetaceae, we were only able to annotate 52 protein-coding genes in their chloroplast genomes, which is consistent with previous studies [25]. We then compared these assembled chloroplast genomes and constructed the phylogenetic tree using RAxML [26] and IQ_TREE [27]. A total of 78 individual coding genes were identified from 738 samples, the majority of ~~them~~ which were present in 710 to 738 samples (on average). However, only 18 genes were found to be consistently present among all the plastid genomes,

Gnetales and Pinales nearly lost all ndh and rps genes (Table S3). ~~only 18 genes were commonly found in all of the samples studied~~. Each gene was aligned using MAFFT [28] and every alignment was then processed with TrimAL [29] using the gappyout option to remove poorly aligned positions. Then gene alignments were combined, which resulted in ~~46235~~ 59695 nucleotide positions. Maximum likelihood (ML) species trees were constructed ~~by~~ using the RAxML package (v8.2.4) with the GTRCAT model, 1,000 bootstrap replicates, ~~5~~ a random seed number:123456 were selected for the parsimony inferences and 26 fern samples were used to root the tree. ~~At the same time,~~ ML analyses were also performed with IQ-TREE under the substitution model GTR+F+R10 which was determined according to the Akaike information criterion (AIC) and the Bayesian information criterion (BIC) by IQ-TREE. ~~As~~With the increase in amount of phylogenetic data~~ sets have increased~~, it has become increasingly important to choose different substitution model for variation in rates and patterns of substitution among sites. We partitioned 59,695 nucleotide positions to 76 groups of sites based on the gene content, then edge-linked-equal partition model. ~~b~~But, separate models between partitions were used with the parameter: -m “GTR+I+G” by IQ-TREE (named as IQ-TREE partitions). Both RAxML and IQ_TREE provided ~~concordant~~ consistent phylogenetic reconstructions (Figure 2~~3~~ and Figure ~~S2~~S1). All nodes in the phylogenetic tree with partitioning scheme ~~are~~were ~~the~~ same with no partitioning scheme in~~using~~ IQ-TREE. The major lineages can be observed within Fabales, Rosales, Poales and Malpighiales. In Fabids, Celastrales was the sister group

Commented [MP1]: Parsimony? Only ML methods used. Clarify here.

Commented [杨婷(Ting2R1]: Yes, we used ML method. A random seed is set to guarantee that we will generate a deterministic parsimony starting tree ,so we used command –p 12345

Formatted: Font: (Default) Times New Roman, Font color: Custom Color(RGB(42,42,42)), Pattern: Clear (White)

Formatted: Font color: Custom Color(RGB(42,42,42)), Pattern: Clear (White)

Formatted: Font: (Default) Times New Roman, Font color: Custom Color(RGB(42,42,42)), Pattern: Clear (White)

Formatted: Font: (Default) Times New Roman, Font color: Custom Color(RGB(42,42,42)), Pattern: Clear (White)

Formatted: Font: (Default) Times New Roman, Font color: Custom Color(RGB(42,42,42)), Pattern: Clear (White)

Formatted: Font: (Default) Times New Roman, Font color: Custom Color(RGB(42,42,42)), Pattern: Clear (White)

Formatted: Font: (Default) Times New Roman, Font color: Custom Color(RGB(42,42,42)), Pattern: Clear (White)

Formatted: Font: (Default) Times New Roman, Font color: Custom Color(RGB(42,42,42)), Pattern: Clear (White)

Formatted: Font: (Default) Times New Roman, Font color: Custom Color(RGB(42,42,42)), Pattern: Clear (White)

to Malpighiales other than Oxalidales in this study (BS=100%). For Petrosaviidae, the major ordinal relationship was consistent with the previous research, as well as for Liliales, Asparagales, Poales, Arecales, Commelinales, Pandanales, and Zingiberales ~~in the same clade,~~; the earliest-branching lineage is Alismatales [30]. Relationships between Gentianales, Lamiales and Solanales remained unclear [31, 32]. ~~,~~ ~~and~~ In our study, the ML tree provided support for Gentianales sister to Lamiales (BS=83%) with sister group to Solanales and Boraginales (BS=100%). We also included 54 species of Poales in the ~~phylogenetic tree~~analyses, which revealed its close relationship with Arecales rather than Pandanales and Dioscoreales.

Genome size, repeat content, and heterozygosity

In order to ensure the quality and effectiveness of the dataset (Table 1), we conducted several analyses to reveal the basic genomic features of the vascular plants sampled. By using GCE [33] and kmergenie [34] software and the clean data of each species, we estimated the genome sizes, repeat content and heterozygosity (Figure ~~2~~ 3 and Table S1). For several of these tested species, the genome sizes have been previously measured by experimental approaches and are publicly available (http://data.kew.org/cvalues/) (Table ~~S3~~S4). We compared the previous estimations to the genome sizes estimated by k-mer analysis in this study, and found good agreement between them (R2=0.63) (Figure S2~~1~~). We found that despite overall wide variation in the genome sizes of these plants, most of the families had relatively comparable genome sizes. The most diverse~~d~~ family in terms of genome size was found to be Cupressaceae,

in which genome sizes ranged~~ing~~ from 0.18 Gb in *Cunninghamia lanceolata* (Lamb.) Hook. var. *lanceolata* to 19.26 Gb in *Juniperus pingii* var. *wilsonii* (Rehder) Silba. In addition, repeat content varied from 10% to 88% on average among species sampled, with several exceptions in Cornaceae, Myrtaceae and Celastraceae. For instance, Myrtaceae (Myrtales) were~~as~~ found to have the most repetitive genomes (~88% of repetitive content), while Celastraceae (Celastrales) ~~was~~ waere found to have the least repetitive genomes (~10% repetitive content). We also found relatively high heterozygosity in these species ranging from 0.15% to 36.6% ~~individually~~, per individual, which probably reflected their nature as wild species.

Genome assemblies

Despite the limitation of having only one sequencing library constructed for each species, we were able to conduct preliminary genome assemblies for many of them~~se species~~, which reflected the quality and reuse potential of the data. Based on the estimated heterozygosity and repeat content, we initially selected 17 species from 17 families with relatively simple genome content (heterozygosity rate less than 1% and repeat content less than 50%) for genome assembly. We used SOAPdenovo2 [35] (parameters: pregraph-K 35 contig -M 1 scaff). We obtained an average contig N50 of 4.62 kb, and an average scaffold N50 of 32.2 kb for these genome assemblies. Two species *Alternanthera sessilis* (L.) R.Br. ex DC. and *Senna alata* (L.) Roxb., were assembled to contig N50 of 15.2 kb, scaffold N50 of 95.5 kb and contig N50 of 14 kb and ~~the~~ scaffold N50 of 101.1 kb respectively (Table ~~S4~~S5). We then carried out

Formatted: Font: Italic

Formatted: Font: Italic

Formatted: Font: (Default) Times New Roman, Font color: Auto

Benchmarking Universal Single-Copy Orthologs (BUSCO) (version 3.0.1) analysis [36] to find the completeness of all these 17 genome assemblies. On average, genome completeness was found to be ~89.1%, 1,243 BUSCOs were complete and single-copy and 40 BUSCOs were complete and duplicated (from a total of 1,440 BUSCOs). The average numbers of fragmented and missing BUSCOs were 55 and 101, respectively (Table S5S6). Our preliminary assemblies were of good quality, providing a useful reference for future efforts to establish complete reference genomes for all these plant species. In addition to the current attempt of genome assembly, continuing efforts are being carried out to finish the preliminary assemblies of the other species and these are being deposited and linked with existing already public sequencing data.

Data access and reuse potential

The data generated here includes images, raw sequencing data, assembled chloroplast genomes, and preliminary nuclear genome assemblies. All the data have been organized and linked to a top-level accession in the GigaScience GigaDB repository (http://doi.org/10.5524/100502), containing the lists of all the species and the links to each species page. In addition, each species has a DOI assigned to them containing information on collection number, an image of the plant during sampling, SRA accession number for the raw data, a data file containing the assembled chloroplast genome sequence in FASTA format (these chloroplast sequences can also be found in Table S2), a data file containing the preliminary assembled nuclear genome sequence (available only for some species and will continue to be updated when each assembly

is completed). Voucher specimens are stored in the Herbarium of China National GeneBank (HCNGB), and digitized images for every sheet are also being made available in GigaDB alongside the sequencing data. The data reported in this study are also available in the CNGB Nucleotide Sequence Archive (CNSA: https://db.cngb.org/cnsa ; accession number CNPhis0000538). All raw data are also stored deposited in the NCBI SRA repository under the project number PRJNA43840. In addition to the description in SRA, the SRA accession number of raw data is also included in the GigaDB entries, thus the raw data of specific species can be traced from GigaDB. Datacite and GigaDB (http://doi.org/10.5524/100502) metadata are all linked, and any future updates made on the GigaDB dataset provides traceable records.

The high-depth whole genome sequencing data together with images and voucher specimens can be reused in different ways and will be valuable for future applications. First of all, in addition to the phylogenetic analysis carried out here using on the assembled chloroplast genomes, future evolutionary analysis can be carried out to study the evolution of specific genes after assembling them from raw reads, as well as investigating particular features of plant genome evolution including evolution of repeats, polyploidization, whole genome duplication, etc. Secondly, the data can be used to improve future genome assemblies of these plant species. For example, utilizing the information on repeat content, heterozygosity and genome size estimation provided here to tailor new sequencing and genome assembly strategies of these plant genomes, as well as integrating the sequencing data itself in other genome assemblies. By directly

using the sequencing data obtained from this study, it would be easier and more efficient to assemble the remaining sequenced plant genomes. The ~~~70~~ 60 Gb data can be used for genome assembly in combination with either contig reconstruction of the second generation based sequence reads, or for error correction of the third generation long sequence reads. Last but not least, this dataset can also be used for developing new methods for species identification either based on sequencing data or based on images of plants and to resolve phylogenetic relationships based on whole genome sequencing data, among others. At ~~the~~ present, we ~~have~~do not have enough information to identify all the species, so we are building a living plant database which records the position of ~~r~~Ruili species and ~~keep tracking the state for these~~monitor the status of each species, the database can be visited through the U~~T~~RL ~~position:~~

http://720yunnan.com/tour/a2b8096d43d7226d?scene=scene_d3627cc2a43314d8.

~~For example,~~ ~~D~~deep learning ~~can~~ could also be applied to develop plant identification using this dataset as a good training set in combination with other accumulating information in future. We used 175 known species from ~~r~~Ruili data for deep learning, every sample extracted 1M reads to build the model. At the first trial stage, 181 species have been successfully identified to species level using our these models. Providing this comprehensive dataset which can be easily accessed by researchers and also the general public, we ~~beleive~~believe it would be reused in many ways beyond what has been mentioned here.

Discussion

Commented [MP3]: I don't really buy this: the sampling is too sparse: multiple accessions of species would be needed

Commented [杨婷(Ting4R3]: The URL is in the final stage of modification which could be visit on January

Commented [MP5]: As above

Formatted: Font: (Default) +Body (DengXian)

The current understanding ~~on~~ of the evolution of plants and its diversity in a phylogenomic context is limited due to the ~~non-availability~~lack of genome-scale information across phylogenetically diverse species ~~[33]~~. In this study, we provide a dataset of high-depth whole genome sequencing of 689 vascular plant species with voucher specimens, covering 134 families and 47 orders. These samples were obtained from Ruili Botanical Garden in Yunnan Province of China, near the border between China and Myanmar, reflecting the rich plant diversity in that region. The high-depth whole genome sequencing data generated here have been used to estimate genomic features including genome size, repeat content ~~,~~ and heterozygosity, which can provide guidance to ~~the~~ future studies aiming at establishing reference genomes for these species. The high-depth whole genome data can ~~be~~ also be used in assembling chloroplast genomes, as well as some conserved nuclear genes, thus providing useful information for evolution and gene function studies.

In this study, we scaled up ~~the plant~~ whole genome sequencing effort to sequence hundreds of plant species. We only constructed a single short insert library (200 bp) for each of the species and generated ~60 Gb of whole genome sequencing data. It would be insufficient to assemble ~~good draft~~high-quality genomes for the majority of the species just based on single library data, however, the current data could potentially be used for several analyses such as such as gene finder, plastid and mitochondrial assembly. At present, we are using these data in combination with 10x genomics to get high quality genome data. ~~because previous efforts to assemble reference genomes based on second generation sequencing data have required multiple short insert libraries and also mate pair (large insert size) libraries~~. In addition, our study tested for the first time, the feasibility of large-scale whole genome sequencing, which is already underway for the Earth BioGenome Project

Formatted: Left, Indent: First line: 0", Line spacing: single, Don't adjust space between Latin and Asian text, Don't adjust space between Asian text and numbers

Formatted: Font color: Blue, Check spelling and grammar

Formatted: Font: (Default) Times New Roman, Font color: Blue

Formatted: Font color: Blue

(EBP) [7] and 10 thousand Plant Genome Projects (10KP) [37]. This study provided experience~~s~~ for plant sampling, sample logistics and management, DNA extraction, sequencing library preparation, sequencing and data analysis and management. Aiming at sequencing more than 10,000 plant species, 10KP ~~would~~ requires the ~~to~~ establishment of a robust infrastructure for sample and data management, as potentially investigated in this pilot study. Now, we have optimized the DNA extraction protocol and published the protocol [19]. Soon we will launch the DNA extraction kit. We also have just finished a guideline about sample submission for 10KP which includ~~inges~~ sample preparation (fresh sample, DNA sample and RNA sample), sample packing and shipping. The specific guidelines will be soon available in our 10KP website (https://db.cngb.org/10kp/).

Formatted: Font: (Default) Times New Roman, 12 pt, Font color: Auto

Formatted: Font: (Default) Times New Roman, 12 pt, Font color: Auto

Formatted: Font: (Default) Times New Roman, 12 pt, Font color: Auto

Formatted: Font: (Default) Times New Roman, 12 pt, Font color: Auto

Formatted: Font: (Default) Times New Roman, 12 pt, Font color: Auto

Availability of Supporting Data

The specimens, leaf samples and DNA solutions of all collections are stored at the China National GeneBank (CNGB) Herbarium. The raw sequencing data described in this article are available in the NCBI SRA repository, under the project number PRJNA43840. DNA Extraction [19]~~[27]~~ and BGISEQ-500 WGS library construction protocols can be found in protocols.io ~~[15][33]~~ [20]. A total of 738 chloroplast genomes and 17 assembled genomes together with raw data supporting the results of this article are available via the GigaDB repository of GigaScience, and will be continuously updated and linked to the GigaDB entries as new assemblies are completed.

Additional files

Additional file 1

Table S1. List of samples included in this study with voucher information, current kmer

based estimation of genome sizes, repeat content and heterozygosity. Identified collections were listed with species names, while unidentified ones with only family and order information. 738 samples with assembled chloroplast genome were marked with *, whereas 17 samples with assembled unclear genomes were marked with §.

Table S2. All the assembled chloroplast genomes and their lengths.

Table S3. The gene content information for all the assembled chloroplast genomes.

Table ~~S3~~S4. Genome information previously measured and publicly available on the database.

Table ~~S4~~S5. Summary of preliminary genome assemblies of 17 species of vascular plants families.

Table ~~S5~~S6. Summary of BUSCO analysis for 17 species of vascular plant~~s~~ families.

Additional file 2

~~**Figure S1.** A comparison of genome sizes measured by the experimental approaches to the k-mer estimated genome sizes in this study.~~

Figure S1~~2~~. Phylogeny of vascular plants of the Ruili Botanical Garden. ~~The tree shows the species~~ Species tree based on 78 the ML analysis of chloroplast genes generated by~~by~~ RAxML. ~~The~~ Colors of the inner circle and ~~the~~ outer ~~circus colors~~ circle represent different families and orders. The clade color represents bootstrap values from red to gray (bootstrap ranges from 50 to 100).

Figure S2. A comparison of genome sizes measured by experimental approaches to the

Formatted: Font: Not Bold

Formatted: Font color: Auto

Formatted: Font: Not Bold

Formatted: Font: Not Bold

Formatted: Font: Not Bold

Formatted: Font: Not Bold

Formatted: Font: (Default) Times New Roman, 12 pt, Not Bold, Font color: Auto

Formatted: Font: (Default) Times New Roman, 12 pt, Not Bold, Font color: Auto

k-mer estimated genome sizes in this study.

Formatted: Font: (Default) +Body (DengXian)

Formatted: Normal

Abbreviations

10 KP: 10 thousand Plant Genome Project

bp: base pair

BUSCO: Benchmarking Universal Single-Copy Orthologs.

EBP: Earth BioGenome Project.

Gb: Gigabase ~~pair~~

HCNGB: Herbarium, China National GeneBank.

ML: Maximum likelihood.

WGS: Whole Genome Sequencing.

Competing interests

All authors declare that they have no competing interests.

Funding

This work was supported by grants of Basic Research Program, the Shenzhen Municipal Government, China (No.JCYJ20150529150505656) and (No.JCYJ20150831201643396), as well as funding from ~~State Key Laboratory of Agricultural Genomics (No.2011DQ782025),~~ Guangdong Provincial Key Laboratory

of Genome Read and Write（No.2017B030301011）, and The Construction of China National GeneBank (Yunnan GeneBank) (Yunnan province, 2015DA008, P.R. China)

Author contributions

XL conceived this study. XL and HL drafted the manuscript. HL managed the project. JPW, XBW, LC, XFH, HCC, JLY, YW, RCM, JL, JMZ collected the samples. TY lead identification of voucher specimens. TY, WXM, BS, YF, YC, HYC analyzed the data. TY, XLC, MW, ZHH constructed the phylogenetic tree. GHH, WSL, HCZ, HCC, YL extracted DNA and performed genome sequencing. SKS and XX revised and edited the manuscript. All the authors have read and approved the final manuscript.

Acknowledgments

The authors would like to express their sincere thanks to the local people and Government of Yunnan province, and the Forestry Institute of Dehong Prefecture for their kind help in sample collections. We would also like to thank the taxonomic experts in PE (Herbarium, Institute of Botany, Chinese Academy of Sciences) for identification. Finally, we are thankful to the production team of China National GeneBank, Shenzhen, China.

References

1. Pennisi E. Plant biology. Green genomes. Science. 2011;332 6036:1372-5. doi:10.1126/science.332.6036.1372.
2. Bolger ME, Weisshaar B, Scholz U, Stein N, Usadel B and Mayer KF. Plant genome sequencing - applications for crop improvement. Curr

Opin Biotechnol. 2014;26:31-7. doi:10.1016/j.copbio.2013.08.019.
3. Desta ZA and Ortiz R. Genomic selection: genome-wide prediction in plant improvement. Trends Plant Sci. 2014;19 9:592-601. doi:10.1016/j.tplants.2014.05.006.
4. Kew RBG. The state of the world's plants report–2016. Royal Botanic Gardens, Kew. 2016.
5. Matasci N, Hung L-H, Yan Z, Carpenter EJ, Wickett NJ, Mirarab S, et al. Data access for the 1,000 Plants (1KP) project. Gigascience. 2014;3 1:17.
6. Cheng S, Melkonian M, Smith SA, Brockington S, Archibald JM, Delaux P-M, et al. 10KP: A phylodiverse genome sequencing plan. Gigascience. 2018;7 3:giy013.
7. Lewin HA, Robinson GE, Kress WJ, Baker WJ, Coddington J, Crandall KA, et al. Earth BioGenome Project: Sequencing life for the future of life. Proc Natl Acad Sci U S A. 2018;115 17:4325-33.
8. de Vere N, Rich TC, Trinder SA and Long C. DNA barcoding for plants. Methods Mol Biol. 2015;1245:101-18. doi:10.1007/978-1-4939-1966-6_8.
9. Staats M, Erkens RH, van de Vossenberg B, Wieringa JJ, Kraaijeveld K, Stielow B, et al. Genomic treasure troves: complete genome sequencing of herbarium and insect museum specimens. PLoS One. 2013;8 7:e69189. doi:10.1371/journal.pone.0069189.
10. Osmundson TW, Robert VA, Schoch CL, Baker LJ, Smith A, Robich G, et al. Filling gaps in biodiversity knowledge for macrofungi: contributions and assessment of an herbarium collection DNA barcode sequencing project. PLoS One. 2013;8 4:e62419. doi:10.1371/journal.pone.0062419.
11. Li X, Yang Y, Henry RJ, Rossetto M, Wang Y and Chen S. Plant DNA barcoding: from gene to genome. Biol Rev Camb Philos Soc. 2015;90 1:157-66. doi:10.1111/brv.12104.
12. Straub SC, Parks M, Weitemier K, Fishbein M, Cronn RC and Liston A.

Navigating the tip of the genomic iceberg: Next-generation sequencing for plant systematics. Am J Bot. 2012;99 2:349-64. doi:10.3732/ajb.1100335.

13. Male PJ, Bardon L, Besnard G, Coissac E, Delsuc F, Engel J, et al. Genome skimming by shotgun sequencing helps resolve the phylogeny of a pantropical tree family. Mol Ecol Resour. 2014;14 5:966-75. doi:10.1111/1755-0998.12246.
14. Besnard G, Christin PA, Male PJ, Coissac E, Ralimanana H and Vorontsova MS. Phylogenomics and taxonomy of Lecomtelleae (Poaceae), an isolated panicoid lineage from Madagascar. Ann Bot. 2013;112 6:1057-66. doi:10.1093/aob/mct174.
15. Mak SST, Gopalakrishnan S, Carøe C, Geng C, Liu S, Sinding M-HS, et al. Comparative performance of the BGISEQ-500 vs Illumina HiSeq2500 sequencing platforms for palaeogenomic sequencing. Gigascience. 2017;6 8:1-13.
16. Huang J, Liang X, Xuan Y, Geng C, Li Y, Lu H, et al. A reference human genome dataset of the BGISEQ-500 sequencer. Gigascience. 2017;6 5:1-9.
17. Fang C, Zhong H, Lin Y, Chen B, Han M, Ren H, et al. Assessment of the cPAS-based BGISEQ-500 platform for metagenomic sequencing. Gigascience. 2017;7 3:gix133.
18. Patch A-M, Nones K, Kazakoff SH, Newell F, Wood S, Leonard C, et al. Germline and somatic variant identification using BGISEQ-500 and HiSeq X Ten whole genome sequencing. PloS one. 2018;13 1:e0190264.
19. Wu C and Yang T. DNA Extraction for plant samples by CTAB. Gigascience. 2018; doi:10.17504/protocols.io.pzqdp5w.
20. Gao S, Mu F, Yang Z, Liu X, Jiang H, Liao S, et al. BGISEQ-500 WGS library construction. 2018; doi:10.17504/protocols.io.ps5dng6.
21. Dierckxsens N, Mardulyn P and Smits G. NOVOPlasty: de novo assembly of organelle genomes from whole genome data. Nucleic acids research. 2016;45 4:e18-e.

22. Hahn C, Bachmann L and Chevreux B. Reconstructing mitochondrial genomes directly from genomic next-generation sequencing reads—a baiting and iterative mapping approach. Nucleic acids research. 2013;41 13:e129-e.
23. Wyman SK, Jansen RK and Boore JL. Automatic annotation of organellar genomes with DOGMA. Bioinformatics. 2004;20 17:3252-5. doi:10.1093/bioinformatics/bth352.
24. Birney E, Clamp M and Durbin R. GeneWise and Genomewise. Genome Res. 2004;14 5:988-95. doi:10.1101/gr.1865504.
25. Hsu CY, Wu CS, Surveswaran S and Chaw SM. The complete plastome sequence of Gnetum ula (Gnetales: Gnetaceae). Mitochondrial DNA A DNA Mapp Seq Anal. 2016;27 5:3721-2. doi:10.3109/19401736.2015.1079874.
26. Stamatakis A. RAxML version 8: a tool for phylogenetic analysis and post-analysis of large phylogenies. Bioinformatics. 2014;30 9:1312-3.
27. Nguyen L-T, Schmidt HA, von Haeseler A and Minh BQ. IQ-TREE: a fast and effective stochastic algorithm for estimating maximum-likelihood phylogenies. Molecular biology and evolution. 2014;32 1:268-74.
28. Katoh K, Misawa K, Kuma K and Miyata T. MAFFT: a novel method for rapid multiple sequence alignment based on fast Fourier transform. Nucleic Acids Res. 2002;30 14:3059-66.
29. Capella-Gutiérrez S, Silla-Martínez JM and Gabaldón T. trimAl: a tool for automated alignment trimming in large-scale phylogenetic analyses. Bioinformatics. 2009;25 15:1972-3.
30. Chase MW. Monocot relationships: an overview. Am J Bot. 2004;91 10:1645-55. doi:10.3732/ajb.91.10.1645.
31. Bremer K, Backlund A, Sennblad B, Swenson U, Andreasen K, Hjertson M, et al. A phylogenetic analysis of 100+ genera and 50+ families of euasterids based on morphological and molecular data with notes on possible higher level morphological synapomorphies. Plant Systematics and Evolution. 2001;229 3-4:137-69.

32. Refulio-Rodriguez NF and Olmstead RG. Phylogeny of lamiidae. American Journal of Botany. 2014;101 2:287-99.
33. Liu B SY, Yuan J, Hu X, Zhang H, Li N, Li Z, Chen Y, Mu D, Fan W. Estimation of genomic characteristics by analyzing k-mer frequency in de novo genome projects. arXiv preprint. 2013; doi:arXiv:1308.2012.
34. Chikhi R and Medvedev P. Informed and automated k-mer size selection for genome assembly. Bioinformatics. 2014;30 1:31-7. doi:10.1093/bioinformatics/btt310.
35. Luo R, Liu B, Xie Y, Li Z, Huang W, Yuan J, et al. SOAPdenovo2: an empirically improved memory-efficient short-read de novo assembler. Gigascience. 2012;1 1:18.
36. Simão FA, Waterhouse RM, Ioannidis P, Kriventseva EV and Zdobnov EM. BUSCO: assessing genome assembly and annotation completeness with single-copy orthologs. Bioinformatics. 2015;31 19:3210-2.
37. Cheng S, Melkonian M, Smith SA, Brockington S, Archibald JM, Delaux P-M, et al. 10KP: A Phylodiverse Genome Sequencing Plan. GigaScience. 2018.

Figure legends

Figure 1. Sampling localities of this project. Sampling was conducted mainly in Ruili Botanical Garden in Southwest China, near the China-Myanmar border, and shown in red rectanglescircles.

Figure 2. The ordinal phylogeny is based on "drop-tips" from Figure 3. Genome size, repeat content and heterozygosity statistics of the nuclear genomes assembled in this study. (a) Genome sizes in GB, (b) repeat content as percentage of total genome (%), (c) heterozygosity ratio Cladogram was generated from the 78 chloroplast gene phylogeny.

Figure 32. Phylogeny of vascular plants of the Ruili Botanical Garden based on the ML analysis. The tree of 78 chloroplast genes. In the inner circleus, cColors represent different families and in the outer circleus colors color in outer circle represent different orders.

Figure 3. The ordinal phylogeny is based on "drop-tips" from Figure 2. Based on species phylogenetic tree, we used drop.tip function in ape package (5.2) to remove the corresponding internal branches. Genome size, repeat content and heterozygosity statistics of the nuclear genomes assembled in this study were given. (a) Genome sizes in G, (b) repeat content as percentage of total genome (%), (c) heterozygosity ratio Cladogram was generated from the 78 chloroplast gene ML phylogeny using only one tip per orders.

Tables

Commented [MP6]: Not clear what this means

Commented [MP7]: incomplete sentence

Formatted: Font: (Default) Times New Roman, Font color: Auto

Formatted: Font: (Default) Segoe UI, 10.5 pt

Formatted: Left, Line spacing: single, Don't adjust space between Latin and Asian text, Don't adjust space between Asian text and numbers

Formatted: Font: (Default) Times New Roman, Font color: Auto

Formatted: Strikethrough

Commented [MP8]: incomplete sentence

Commented [SS9R8]: we have revised the sentence

Formatted: Strikethrough

Formatted: Strikethrough

Formatted: Font: (Default) Times New Roman, Font color: Auto

Table 1 Summary of the sequencing data produced in this study.

Table 1

Click here to access/download;Table;Table 1.xlsx

Order	Raw base(Gb)	Raw data G	Raw data C	Raw data Q30
Alismatales	66.3873	43.64	95.34	86.48
Apiales	70.0075	35.42	96.40	88.40
Araucariales	74.14	32.87	96.50	88.85
Arecales	68.8318	39.95	95.84	87.20
Asparagales	70.3465	37.97	96.16	87.87
Asterales	67.8382	37.41	95.83	87.20
Brassicales	68.474	37.89	95.99	87.45
Buxales	65.44	42.34	95.38	86.00
Caryophyllales	68.6558	38.04	95.73	87.03
Celastrales	75.8133	38.12	96.56	88.57
Commelinales	65.02	36.80	95.58	86.81
Cornales	76.396	36.49	96.44	88.63
Crossosomatales	60.2	37.17	95.36	86.54
Cucurbitales	65.11	35.73	95.50	86.22
Cupressales	73.54	36.12	96.78	89.01
Cyatheales	75.76	41.32	96.64	88.37
Dioscoreales	78.9	41.47	94.99	85.65
Dipsacales	58.6267	37.58	96.22	87.52
Equisetales	67.3	39.98	94.92	84.77
Ericales	68.1109	38.01	96.46	88.02
Fabales	69.9439	35.50	96.14	87.75
Fagales	68.14	36.81	96.13	87.90
Gentianales	70.1155	36.49	96.36	88.27
Gnetales	71.1267	39.77	96.87	89.24
Lamiales	69.3291	37.47	95.94	87.40
Laurales	71.9425	40.22	96.04	87.83
Liliales	71.4133	41.00	96.73	89.15
Magnoliales	69.0988	38.88	96.12	88.01
Malpighiales	68.1842	35.83	96.40	88.23
Malvales	66.2106	37.19	96.26	88.07
Myrtales	70.7924	38.82	96.23	88.20
Oxalidales	68.3533	34.91	95.61	87.20
Pandanales	72.6733	42.07	96.41	88.31
Pinales	61.04	39.56	93.91	82.96
Piperales	63.2533	40.50	96.23	87.84
Poales	69.6407	44.07	95.56	86.73
Polypodiales	68.588	41.39	96.12	87.69
Proteales	69.0733	39.47	96.49	88.23
Ranunculales	67.5644	38.69	95.68	86.80
Rosales	70.0468	36.72	96.36	88.18
Santalales	69.07	38.11	96.47	88.31
Sapindales	70.5628	36.83	96.14	87.89
Saxifragales	70.84	37.74	96.77	89.36
Schizaeales	62.57	43.84	96.83	89.17
Solanales	72.2389	38.38	96.30	87.93
Vitales	65.235	39.17	95.44	86.71
Zingiberales	67.4956	40.57	95.99	87.51

Click here to access/download;Figure;Figure 1.pdf

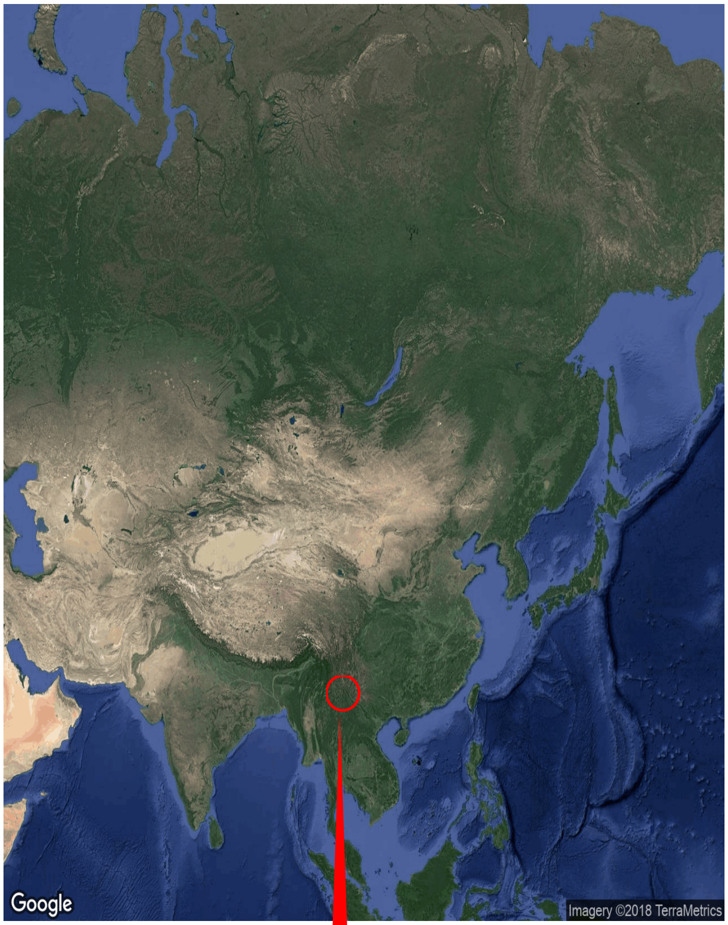

Family	Order
Dipterocarpaceae	Malvales

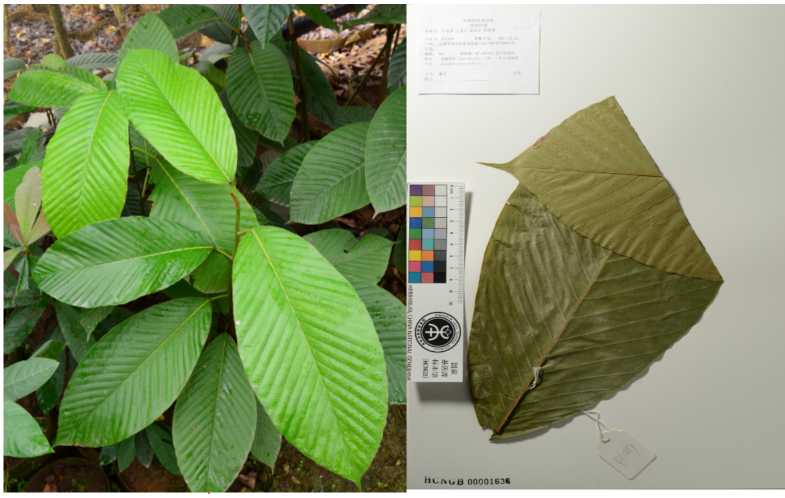

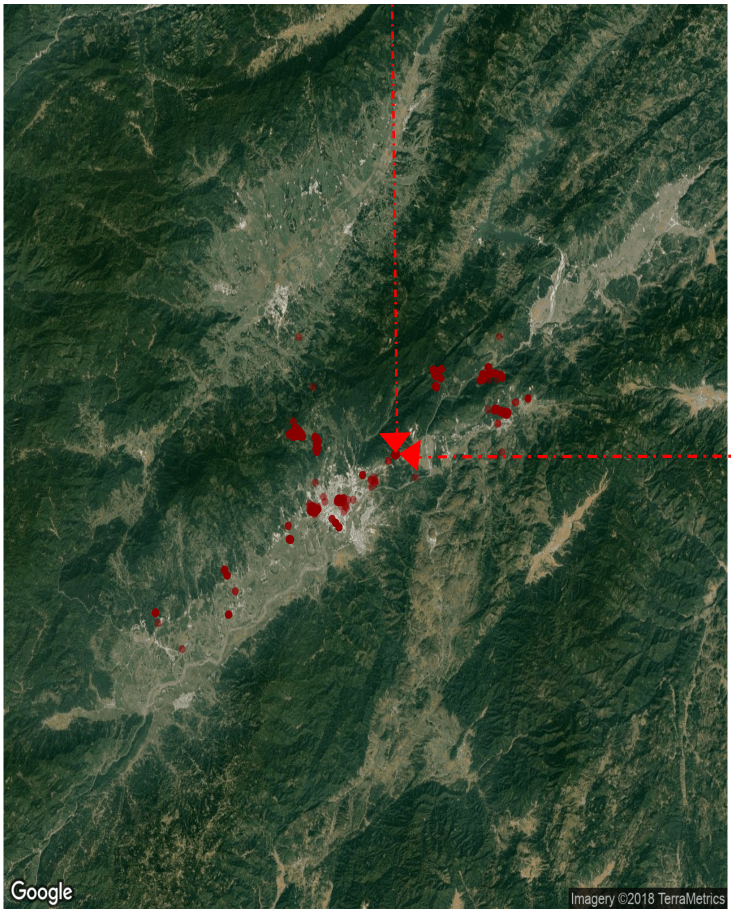

Family	Order
Nyssaceae	Cornales

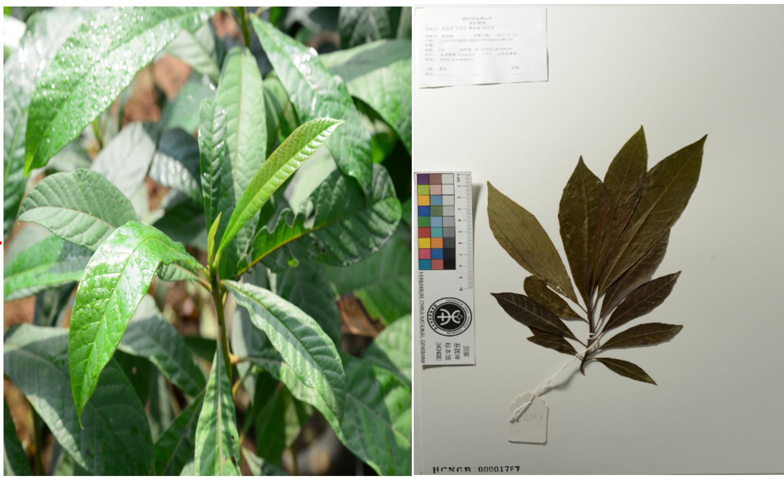

Figure 2

Click here to access/download;Figure;Figure 2.pdf

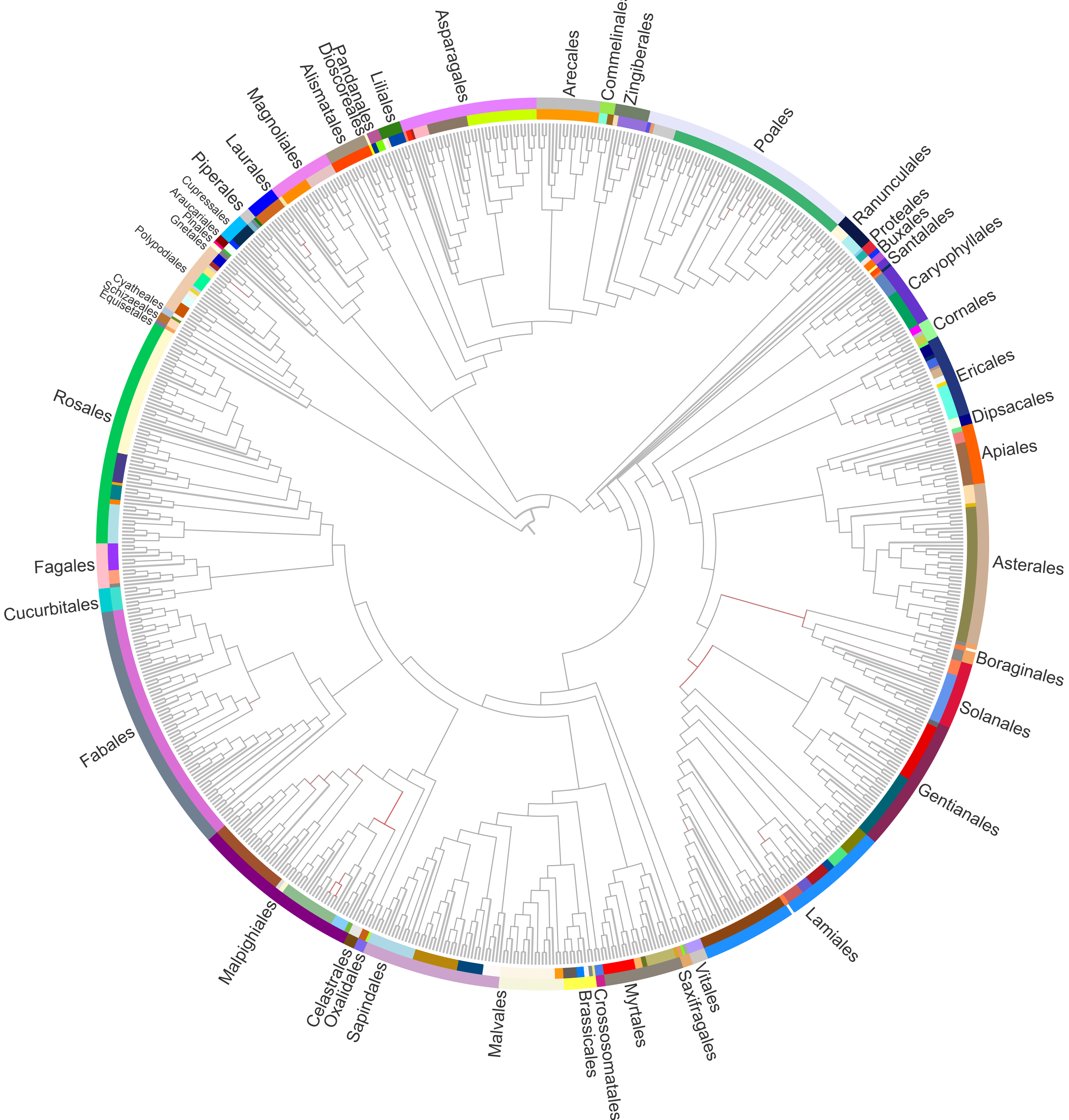

Figure 3

Click here to access/download;Figure;Figure 3.pdf

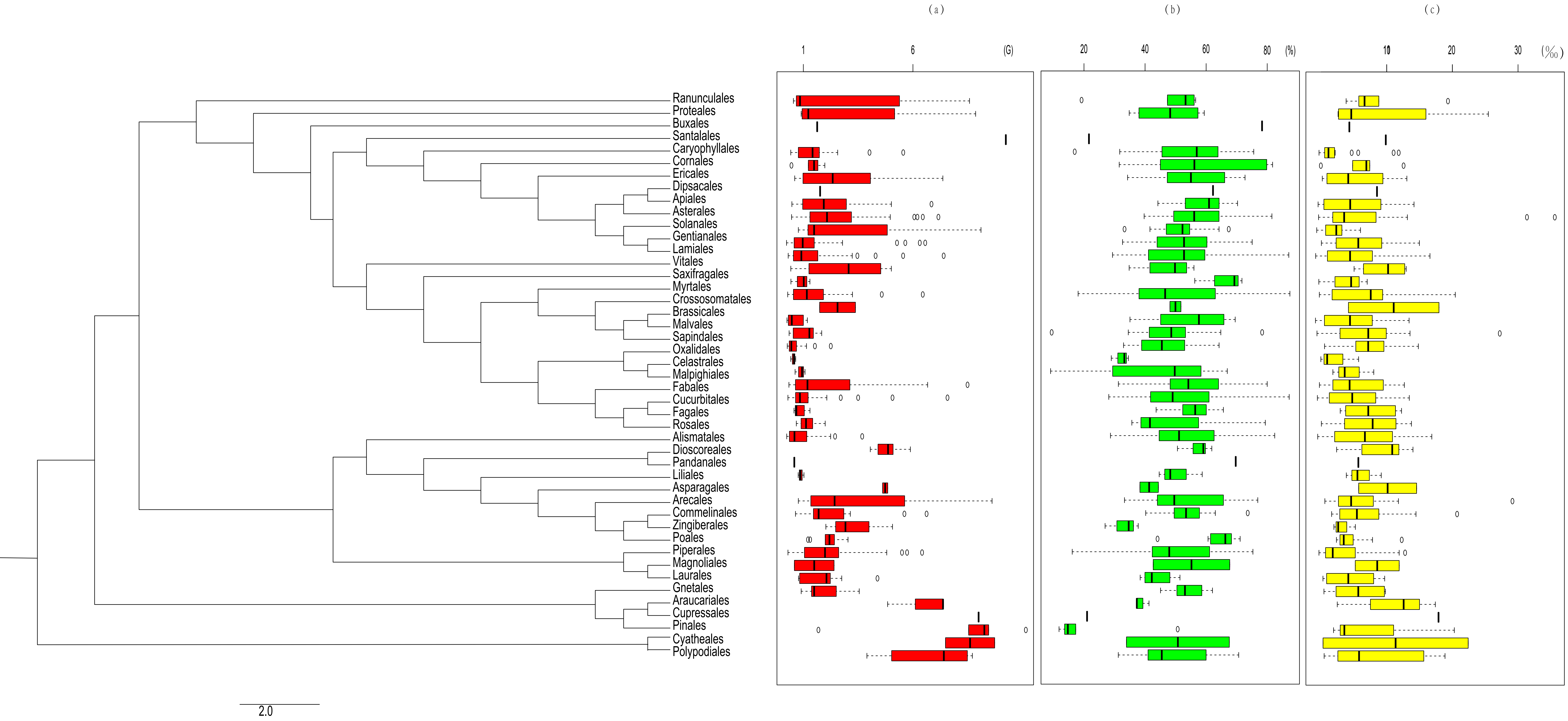

Supplementary Fig S1

Click here to access/download
Supplementary Material
Figure S1.pdf

Supplementary Fig S2

Click here to access/download
Supplementary Material
Figure S2.pdf

Supplementary Table S1

Click here to access/download
Supplementary Material
Table S1.xlsx

Supplementary Table S2

Click here to access/download
Supplementary Material
Table S2.xlsx

Supplementary Table S3

Click here to access/download
Supplementary Material
Table S3.xls

Supplementary Table S4

Click here to access/download
Supplementary Material
Table S4.xlsx

Supplementary Table S5

Click here to access/download
Supplementary Material
Table S5.xlsx

Supplementary Table S6

Click here to access/download
Supplementary Material
Table S6.xlsx

Click here to access/download;Personal Cover;Response to Reviewer.docx

Dear Dr. Scott,

Sub: Submission of the revised manuscript GIGA-D-18-00121R1.

We are glad to submit the thoroughly revised version of our manuscript entitled "Molecular Digitization of a Botanical Garden: High-depth whole genome sequencing of 689 vascular plants from the Ruili Botanical Garden"

The comments of the reviewers were highly insightful and enabled us to greatly improve the quality of our manuscript. According to their advice, we have carefully revised our manuscript.

We present the point-by-point responses to each of the comments and suggestions of the reviewer, we also made a substantial revision in the manuscript with track changes. However, we have uploaded both clean and track changes version for your kind perusal. We strongly believe that these revisions in the manuscript and our accompanying responses are sufficient to make our manuscript suitable for publication in GigaScience.

We look forward to hearing from you at your earliest convenience.

Yours sincerely,

Xin Liu

Reviewer reports:

Reviewer #1:

1) I reviewed the previous version of this submission and am pleased to see that most of the reviewers' comments have been used effectively. My only reservations would remain the low proportion of precise identifications of the samples used, and I trust that the available resources - images; specimens - will be sufficient to address this as the data are further processed and used.

Response: Your comments in the earlier version of the manuscript were really useful, and enabled us to greatly improve the quality of our manuscript. Yes, the genomics data generated in this study makes an enormous contribution to the study of plant genomes of non-model plants along with images, voucher information, complete chloroplast genomes and nuclear genomes. We agree with your concerns, but as mentioned in the manuscript, all the information will be continuously updated and linked to the GigaDB repository as new assemblies are completed.

2) I have been through the text and made a number of suggestions for minor revisions and a few comments which are included in a tracked changes version of the word document. I hope these will be useful and look forward to seeing this impressive piece of work published in due course.

Response:
Thank you for your great suggestions and kind recommendation. We have thoroughly updated the manuscript (with track changes) as per your suggestions. Kindly refer the enclosed manuscript.

Reviewer #2:
The authors have done the right thing and removed the Astral analysis, which was inappropriate for this data. Their new analysis is better, though personally I would raise the following questions:

1. It is very unusual that only 18 conserved genes are found across plastids. In general plastid gene content is highly conserved across plant species. I would suggest the few samples without a standard plastid gene complement are removed and the phylogeny rerun with as many loci as possible. Alternatively, more details could be given as to this issue, perhaps in the Supplementary Information. For example, is it

actually gene loss, or are there issues aligning divergent loci?

Response：Yes, we do agree that the plastid gene content of green plant is highly conserved, but the gene loss was observed in several clades especially in parasitic plants and gymnosperm. For instance, in our study, Gnetales, Pinales lost all ndh and rps genes, Gnetaceae only contained 45 genes. To avoid the confusion, we have now changed the sentence “only 18 conserved genes are found across plastids” to “only 18 genes were found to be consistently present among all the plastid genomes”. The gene content information is listed in supplementary table 3.

2. A single model of molecular evolution is used across loci. It would be much better to use model testing on each locus and rerun the analysis using partitions.

Response: To refer the phylogeny of green plants, Brad et al. (2014) [1] used 360 plastid genomes and partitioned the nucleotide data into four data sets with the GTR+Γ model. They found that the average support values among all internal nodes in the ML trees were slightly higher in the ntAll phylogeny, and ntAll phylogeny also had the most clades resolved with ≥70% BS. In our study, we used IQ-TREE with best model GTR+F+R10 to construct the tree.

According to your advice, we partitioned all nt data set to 76 groups of sites based on gene content, IQ-TREE edge-linked-equal partition model. But, separate models between partitions were used with command -m “GTR+I+G”. However, to our surprise, the phylogenetic tree was the same as our previous result. Please see the enclosed phylogenetic tree for your kind perusal.

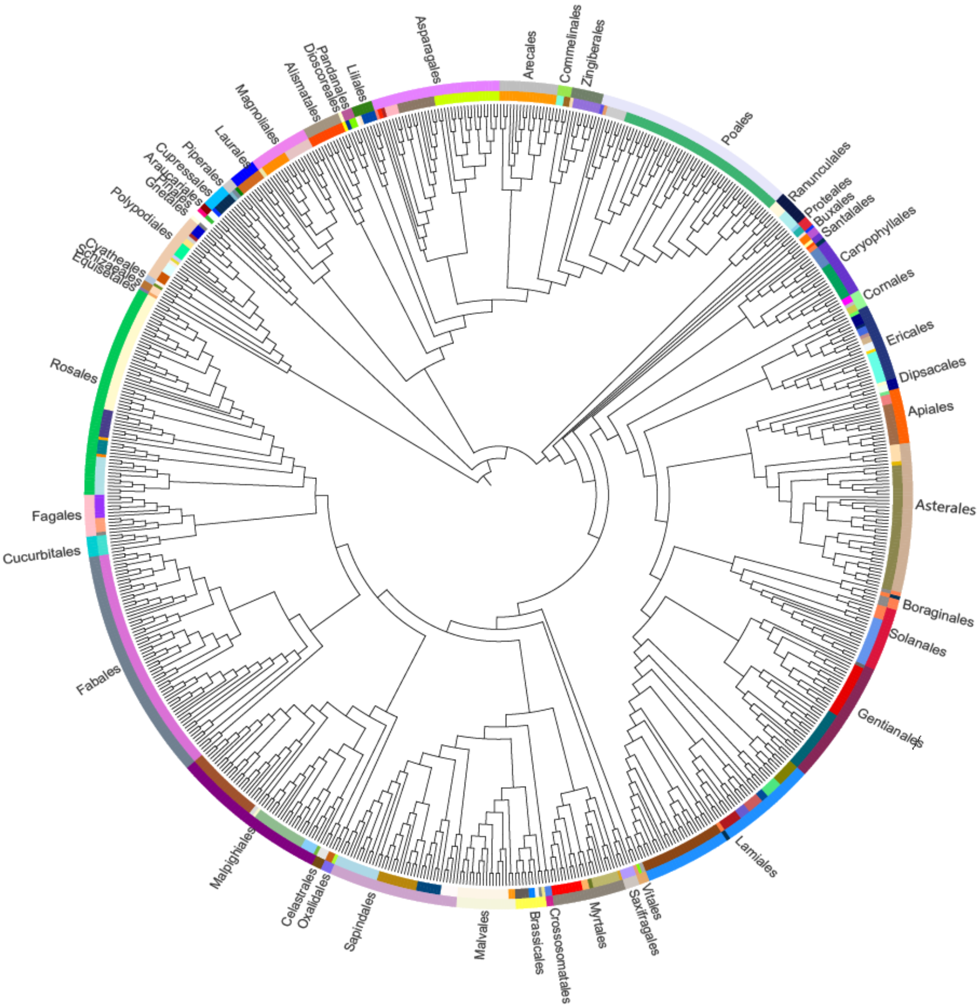

1. Ruhfel BR, Gitzendanner MA, Soltis PS, Soltis DE and Burleigh JG. From algae to angiosperms–inferring the phylogeny of green plants (Viridiplantae) from 360 plastid genomes. BMC evolutionary biology. 2014;14 1:23.
