# Supplementary material for: Molecular digitization of a botanical garden: high-depth whole-genome sequencing of 689 vascular plant species from the Ruili Botanical Garden
Source: Gigascience. 2019 Jan 25;8(4):giz007. doi: 10.1093/gigascience/giz007 (PMC6441391; doi:10.1093/gigascience/giz007)
Supplement: GIGA-D-18-00121_Revision_3.pdf [file giz007_giga-d-18-00121_revision_3.pdf]

GigaScience

Molecular Digitization of a Botanical Garden: High-depth whole genome sequencing of 689 vascular plant species from the Ruili Botanical Garden

--Manuscript Draft--

**Manuscript Number:**	GIGA-D-18-00121R3
**Full Title:**	Molecular Digitization of a Botanical Garden: High-depth whole genome sequencing of 689 vascular plant species from the Ruili Botanical Garden
**Article Type:**	Data Note
**Funding Information:**	the Shenzhen Municipal Government of china (JCYJ20150529150505656) — Dr. Xin Liu<br>the Shenzhen Municipal Government of China (JCYJ20150831201643396) — Dr. Yue Chang<br>The Construction of China National GeneBank (Yunnan GeneBank) (2015DA008) — Dr. Le Cheng<br>State Key Laboratory of Agricultural Genomics (2011DQ782025) — Dr. Huan Liu<br>Guangdong Provincial Key Laboratory of Genome Read and Write (2017B030301011) — Dr. Wangsheng Li
**Abstract:**	Background:<br><br>Genome sequencing has been widely used in plant research to construct reference genomes and provide evolutionary insights. However, few plant species have had their whole genome sequenced, thus restraining the utility of these data. We collected 1,093 samples of vascular plant species growing in the Ruili Botanical Garden, located in South West China. Of these, we sequenced 761 samples, and collected voucher specimens stored in the Herbarium of China National GeneBank.<br><br>Results:<br>The 761 sequenced samples represented 689 vascular plant species from 137 families belonging to 49 orders. Of these, 257 samples were identified to the species level and 504 to the family level, using specimen and chloroplast sequences. In total, we generated 54 Tb of sequencing data, with an average sequencing depth of 60X per species, as estimated from genome sizes. A reference phylogeny was reconstructed with 78 chloroplast genes for molecular identification and other possible applications.<br><br>Conclusions:<br>The large dataset of vascular plant genomes generated by this study, which includes both high-depth whole genome sequencing data and associated voucher specimens, is valuable for plant genome research and other applications. This project also provides insight into the feasibility and technical requirements for ‘planetary scale’ projects such as the 10,000 Plant Genome Project and the Earth BioGenome Project.
**Corresponding Author:**	Xin Liu, Ph.D.<br>BGI<br>CHINA
**Corresponding Author Secondary Information:**	
**Corresponding Author's Institution:**	BGI
**Corresponding Author's Secondary Institution:**	
**First Author:**	Huan Liu
**First Author Secondary Information:**	

Powered by Editorial Manager® and ProduXion Manager® from Aries Systems Corporation

**Order of Authors:**	Huan Liu
	jinpu Wei
	Ting Yang
	Weixue Mu
	Bo Song
	Tuo Yang
	Yuan Fu
	Xuebin Wang
	Guohai Hu
	Wangsheng Li
	Hongcheng Zhou
	Yue Chang
	Xiaoli Chen
	Hongyun Chen
	Le Cheng
	Xuefei He
	Hechen Cai
	Xianchu Cai
	Mei Wang
	Yang Li
	Sunil Kumar Sahu, PhD.
	Jinlong Yang
	Yu Wang
	Ranchang Mu
	Jie Liu
	Jianming Zhao
	Ziheng Huang
	Xun Xu, PhD
	Xin Liu, Ph.D.
**Order of Authors Secondary Information:**	
**Response to Reviewers:**	Dear Dr. Scott,<br><br>We are glad to submit the thoroughly revised version of our manuscript entitled "Molecular Digitization of a Botanical Garden: High-depth whole genome sequencing of 689 vascular plant species from the Ruili Botanical Garden"<br><br>We look forward to hearing from you at your earliest convenience.<br>Yours sincerely,<br>Xin Liu<br><br>Note: No comments by the reviewers
**Additional Information:**	
**Question**	**Response**

Powered by Editorial Manager® and ProduXion Manager® from Aries Systems Corporation

Are you submitting this manuscript to a special series or article collection?	No
**Experimental design and statistics**<br><br>Full details of the experimental design and statistical methods used should be given in the Methods section, as detailed in our Minimum Standards Reporting Checklist. Information essential to interpreting the data presented should be made available in the figure legends.<br><br>Have you included all the information requested in your manuscript?	Yes
**Resources**<br><br>A description of all resources used, including antibodies, cell lines, animals and software tools, with enough information to allow them to be uniquely identified, should be included in the Methods section. Authors are strongly encouraged to cite Research Resource Identifiers (RRIDs) for antibodies, model organisms and tools, where possible.<br><br>Have you included the information requested as detailed in our Minimum Standards Reporting Checklist?	Yes
**Availability of data and materials**<br><br>All datasets and code on which the conclusions of the paper rely must be either included in your submission or deposited in publicly available repositories (where available and ethically appropriate), referencing such data using a unique identifier in the references and in the "Availability of Data and Materials" section of your manuscript.<br><br>Have you have met the above requirement as detailed in our Minimum	Yes

Standards Reporting Checklist?	

Powered by Editorial Manager® and ProduXion Manager® from Aries Systems Corporation

Manuscript
Click here to view linked References
Click here to access/download;Manuscript;GIGA-D-18-00121R3_SCE_Sunil(1).docx

Molecular digitization of a botanical garden: high-depth whole genome sequencing of 689 vascular plant species from the Ruili Botanical Garden

Huan Liu[1,2,3#], Jinpu Wei[1,2#], Ting Yang[1,2,3#], Weixue Mu[1,2,3], Bo Song[1,2], Tuo Yang[1,2], Yuan Fu[1,2], Xuebing Wang[1,2], Guohai Hu[1,2], Wangsheng Li[1,2], Hongcheng Zhou[1,2], Yue Chang[1,2], Xiaoli Chen[1,2,3], Hongyun Chen[1,2,3], Le Cheng[4], Xuefei He[1,2], Hechen Cai[1,2], Xianchu Cai[1,2], Mei Wang[1,2,3], Yang Li[1,2], Sunil Kumar Sahu[1,2,3], Jinlong Yang[4], Yu Wang[4], Ranchang Mu[5], Jie Liu[5], Jianming Zhao[5], Ziheng Huang[1,2,3], Xun Xu[1,2,3], Xin Liu[1,2,3*]

[1]BGI-Shenzhen, Beishan Industrial Zone, Yantian District, Shenzhen 518083, China

[2]China National GeneBank, Jinsha Road, Dapeng New District, Shenzhen 518120, China

[3]State Key Laboratory of Agricultural Genomics, BGI-Shenzhen, Shenzhen 518083, China

[4]BGI-Yunnan, No. 389 Haiyuan Road, High-tech Development Zone, Kunming,

Yunnan 650106, China

[5]Forestry Bureau of Ruili, Yurman Dehong, Ruili 678600, China

[*]Correspondence address: Xin Liu, BGI-Shenzhen, Beishan Industrial Zone, Yantian District, Shenzhen 518083, China; Tel: +86 18025460332; Email: liuxin@genomics.cn

[#]Equal contribution

ORCID: Xin Liu: 0000-0003-3256-2940; Ting Yang: 0000-0002-2872-4954; Huan Liu: 0000-0002-6902-9931; Yue Chang: 0000-0003-3909-0931; Sunil Kumar Sahu: 0000-0002-4742-9870

ABSTRACT

Background: Genome sequencing has been widely used in plant research to construct reference genomes and provide evolutionary insights. However, few plant species have had their whole genome sequenced, thus restraining the utility of these data. We collected 1,093 samples of vascular plant species growing in the Ruili Botanical Garden, located in South West China. Of these, we sequenced 761 samples, and collected voucher specimens stored in the Herbarium of China National GeneBank.

Results: The 761 sequenced samples represented 689 vascular plant species from 137 families belonging to 49 orders. Of these, 257 samples were identified to the species level and 504 to the family level, using specimen and chloroplast sequences. In total, we generated 54 Tb of sequencing data, with an average sequencing depth of 60X per species, as estimated from genome sizes. A reference phylogeny was reconstructed with 78 chloroplast genes for molecular identification and other possible applications.

Conclusions: The large dataset of vascular plant genomes generated by this study, which includes both high-depth whole genome sequencing data and associated voucher specimens, is valuable for plant genome research and other applications. This

project also provides insight into the feasibility and technical requirements for 'planetary scale' projects such as the 10,000 Plant Genome Project and the Earth BioGenome Project.

Keywords: Whole genome sequencing, vascular plants, phylogeny, voucher specimens, Ruili Botanical Garden.

Background

With the advent of next generation sequencing technologies, enormous efforts have been made to sequence the whole genomes of plant species, thereby providing new insights into plant evolution [1] and new information for improving agriculture yield and stress tolerance [2, 3]. As of November 2018, more than 350 land plant genomes have been sequenced [4], most of which are crops (57.7%), model species and their closely related species (22.3%), and crop wild relatives (17.7%). However, considering the evolutionary history and diversity of the 391,000 known species of plants [5], limited sequence data is currently available. The transcriptome sequences of more than 1,000 plant species have recently been elucidated to better understand

plant evolution, thus also providing valuable resources for other plant research [6]. However, considering the high proportion of non-coding regions, studies of plant evolution would benefit from the generation of further whole genome sequencing data.

As a key part of the Earth BioGenome project [7], a global effort called the 10,000 Plant Genomes (10KP) project has been initiated to sequence 10,000 plant genomes [8]. The feasibility of large-scale whole genome sequencing efforts such as this must be determined, as well as establishing technical standards for sampling, sequencing and data management.

DNA barcoding has emerged as an important molecular tool for ecological studies, particularly for the rapid identification of standard specimens [9]. Although it is well-suited for studying historical specimen samples, considering the DNA degradation in those samples [10, 11], a major drawback is that DNA barcoding provides limited genomic information, which is based on only small fragments of the nuclear or chloroplast genome [12]. To overcome this problem, genome skimming, which is whole genome sequencing using second-generation sequencing technologies,

has been proposed [13] to provide more genome sequence information for better species identification [14, 15]. However, previous genome skimming studies have only generated a small amount of sequencing data for individual species. This precludes the re-use of the data to reveal more detailed genome features, including genome sizes (for plants with large genomes), ploidy level, etc., or its direct use in further de novo genome assembly.

Here, we sequenced the genomes of 761 samples, representing 689 vascular plant species, at high depth (more than 60 Gb per sample, on average). By making these data freely accessible and linking them to voucher details stored in the China National GeneBank (CNGB) herbarium and Ruili Botanical Garden, we provide a valuable genomic resource for evolution and diversity research and applications that may reveal new insights into the evolution of vascular plants.

Data description

Sampling, sequencing and data summary

We sampled almost all of the species growing at the Ruili Botanical Garden, Yunnan,

97 China (97°38′47″ to 98°05′57″ N, 23°52′42″ to 24°09′20″ E, altitude range 738–
98 1,200 m above sea level, as shown in Figure 1) – 1,093 vascular plant samples in total.
99 Young leaves from each sample were used for DNA extraction. Voucher specimens
100 and images were also collected for these samples. All specimens are stored in the
101 CNGB herbarium, and voucher information can be found in Table S1 (Additional
102 files). Collected young leaves were shipped to Shenzhen, China, on dry ice, and,
103 using the CTAB method [16], good quality DNA was extracted from 761 samples.
104 Whole genome sequencing libraries were constructed and then sequenced for each of
105 these samples using a BGISEQ-500 desktop sequencer developed by BGI-Shenzhen
106 in 2015, according to the manufacturer's instructions [17]. This machine uses DNA
107 nanoball and combinational probe anchor synthesis technology, developed by
108 Complete Genomics™, to generate short reads on a large scale. Sequencing outputs
109 are comparable with the Illumina series [18], and have been successfully utilized to
110 sequence the human genome [19] and metagenomes [20], and for variant
111 identification [21].
112 Approximately 70 Gb of raw sequencing data (100 bp, paired-end) was generated for

113 each of these samples (Table 1). Raw reads were filtered using SOAPfilter_v2.2 with
114 the following parameters: –y –p –i 180 –M 2 –Q 10. After filtering low-quality reads
115 (reads with more than 10% Ns, ambiguous bases; reads with more than 40% bases
116 having quality lower than 10; reads contaminated by adaptors or PCR duplicates),
117 ~60 Gb of clean data (high-quality reads >Q35) was obtained for each sample.
118

119 **Species identification and phylogenetic relationship**

120 Since the specimens collected in this study covered most extant vascular plant
121 lineages, it was not possible to identify each sample to the species level in the short
122 time available. We identified 257 samples to the species level (250 unique species)
123 using specimen morphology, and the remaining 504 samples were identified to the
124 family level using specimen and chloroplast sequences. Thus, we identified 738
125 samples from 761 sequenced, which belonged to 137 families and 49 orders. Among
126 these families, most species belonged to Fabaceae (71 taxa), Poaceae (45 taxa) and
127 Asteraceae (37 taxa), respectively.

128 We assembled the chloroplast genomes of each species from clean read data using

NOVOPlasty [22], a seed extension-based de novo assembler. We used the complete coding sequence of the *rbcL* gene of *Arabidopsis thaliana* (downloaded from the National Center for Biotechnology Information [NCBI], accession number: U91966) [23] as the seed to conduct the assembly. The NOVOPlasty assembly recovered complete chloroplast genomes of 50 species in a single circular sequence. For the remaining species, the longest contig assembled by NOVOPlasty was BLASTed against the chloroplast database (downloaded from NCBI, including 2,503 non-redundant species) (Table S2) and the resulting best-hit sequences (minimum requirement: e-value < 10-7 and identity >95%) were used as references for further assembly using MITObim [24]. Complete chloroplast genomes were eventually recovered for all 689 species, ranging from 113,621 to 183,602 bp in size (see supplemental data in GigaDB) [25]. Assembled chloroplast genomes were annotated using DOGMA [26] and GeneWise [27]. Seventy-two protein-coding genes were found in almost all of these vascular plant families, except the Gnetaceae, Malvaceae, Elaeocarpaceae, and Tectariaceae. For Gnetaceae, we were only able to annotate 52 protein-coding genes in their chloroplast genomes, which is consistent with previous

145 studies [28].

146 Assembled chloroplast genomes were then compared and a phylogenetic tree
147 constructed using RAxML [29] and IQ_TREE [30]. A total of 78 individual coding
148 genes were identified from 738 samples, most of which were present in 710–738
149 samples (on average). However, only 18 genes were consistently present among all
150 the plastid genomes; Gnetales and Pinales lost nearly all *ndh* and *rps* genes (Table
151 S3).

152 Each gene was aligned using MAFFT [31], and each alignment was then processed
153 with TrimAL [32] using the gappyout option to remove poorly aligned positions.
154 Gene alignments were then combined, resulting in 59,695 nucleotide positions.
155 Maximum likelihood (ML) species trees were constructed using the RAxML package
156 (version 8.2.4) with the GTRCAT model, 1,000 bootstrap replicates, a random seed
157 number (123456) selected for parsimony inferences, and 26 fern samples to root the
158 tree. ML analyses were also performed with IQ-TREE using the substitution model
159 GTR+F+R10, which was determined according to the Akaike information criterion
160 and the Bayesian information criterion by IQ-TREE. With the increase in the amount

of phylogenetic data, it has become increasingly important to choose different substitution models for variation in rates and patterns of substitution among sites. We partitioned 59,695 nucleotide positions to 78 groups of sites based on gene content, then applied the edge-linked–equal partition model. However, between partitions, a separate model was used with the parameter: -m “GTR+I+G” by IQ-TREE (named IQ-TREE partitions).

Both RAxML and IQ_TREE provided consistent phylogenetic reconstructions (Figure 2 and Figure S1). All nodes in the phylogenetic tree created using the partitioning scheme were the same as those created when no partitioning scheme was used in IQ-TREE. The major lineages can be observed as Fabales, Rosales, Poales and Malpighiales. Within the Fabids, Celastrales was shown to be a sister group to the Malpighiales, other than Oxalidales in this study (bootstrap support [BS] = 100%). For the Petrosaviidae, the major ordinal relationship was consistent with previous research: like the Liliales, Asparagales, Poales, Arecales, Commelinales, Pandanales, and Zingiberales, the earliest branching lineage was Alismatales [33]. Relationships between Gentianales, Lamiales and Solanales remained unclear [34, 35].

In this study, the ML tree provided support for the notion that the Gentianales are a sister group to the Lamiales (BS = 83%), which in turn is a sister group to the Solanales and Boraginales (BS = 100%). Fifty-four species of Poales were also analysed, revealing a close relationship of this group with the Arecales, rather than the Pandanales and Dioscoreales.

Genome size, repeat content, and heterozygosity

To ensure the quality and accuracy of the dataset (Table 1), we conducted several analyses to reveal the basic genomic features of the vascular plants sampled. By using GCE [36] and kmergenie [37] software, and clean data for each species, we estimated genome sizes, repeat content and heterozygosity (Figure 3 and Table S1). The genome sizes of several of the tested species have been previously measured and are publicly available [38] (Table S4). We compared these previous estimates to the genome sizes estimated by k-mer analysis in this study, and found good agreement between them (R2 = 0.63) (Figure S2). Overall, despite there being wide variation in the genome sizes of these plants, most of the families had relatively comparable genome sizes.

The most diverse family in terms of genome size was the Cupressaceae, in which genome sizes ranged from 0.18 Gb in *Cunninghamia lanceolata* (Lamb.) Hook. var. *lanceolata*, to 19.26 Gb in *Juniperus pingii* var. *wilsonii* (Rehder) Silba. On average, repeat content also varied from 10–88% between the species sampled, with several exceptions (Cornaceae, Myrtaceae and Celastraceae). Myrtaceae (Myrtales) had the most repetitive genomes (~88% repetitive content), while Celastraceae (Celastrales) had the least repetitive genomes (~10% repetitive content). There was relatively high heterozygosity in these species, ranging from 0.15% to 36.6% per individual, which probably reflects their nature as wild species.

Genome assemblies

Despite having constructed only one sequencing library for each species, we were able to assemble preliminary genomes for many of them, reflecting the quality and reuse potential of our data. Based on estimated heterozygosity and repeat content, we initially selected 17 species from 17 families with relatively simple genome content (heterozygosity rate less than 1% and repeat content less than 50%) for genome

assembly. We used SOAPdenovo2 [39] (parameters: pregraph-K 35 contig –M 1 scaff) and obtained an average contig N50 of 4.62 kb, and an average scaffold N50 of 32.2 kb for these genome assemblies. *Alternanthera sessilis* (L.) R.Br. ex DC was assembled to contig N50 of 15.2 kb and scaffold N50 of 95.5 kb, and *Senna alata* (L.) Roxb. was assembled to acontig N50 of 14 kb and scaffold N50 of 101.1 kb (Table S5). We then carried out Benchmarking Universal Single-Copy Orthologs (BUSCO) (version 3.0.1) analysis [40] to find the completeness of these 17 genome assemblies. On average, genome completeness was found to be ~89.1%; 1,243 BUSCOs were complete and single-copy, and 40 BUSCOs were complete and duplicated (from a total of 1,440 BUSCOs). The average numbers of fragmented and missing BUSCOs were 55 and 101, respectively (Table S6).

Our preliminary assemblies were of good quality, providing a useful reference for future efforts to establish complete reference genomes for these plant species. As well as the current genome assembly effort, work continues to finish the preliminary assemblies of the other species; these will be deposited and linked with existing public sequencing data.

Data access and reuse potential

The data generated here includes images, raw sequencing data, assembled chloroplast genomes, and preliminary nuclear genome assemblies. All data have been organized and linked to a top-level accession in the *GigaScience* GigaDB repository [25], which contains lists of all the species and links to a page for each species. Each species has also been assigned a DOI, linking collection number, a digitized image of the plant taken during sampling, Sequence Read Archive (SRA) accession number for the raw data (filed under SRA project number PRJNA438407 [41]), a data file containing the assembled chloroplast genome sequence in FASTA format (see supplementary data in GigaDB repository [25]), and a data file containing the preliminary assembled nuclear genome sequence (the latter is only available for some species at present, but will be updated as each assembly is completed). Voucher specimens are stored in the herbarium of the CNGB. The data reported in this study are also available in the CNGB Nucleotide Sequence Archive under accession number CNPhis0000538 [42]. With all the metadata indexed and linked via Datacite and GigaDB [25], any

future updates made will be traceable records.

The high-depth whole genome sequencing data, together with images and voucher specimens, can be reused in different ways and will be valuable for future applications. First of all, future evolutionary analysis may be used to study the evolution of specific genes after assembling them from raw reads, as well as investigating particular features of plant genome evolution, including the evolution of repeats, polyploidization, whole genome duplication, etc. Secondly, the data may be used to improve future genome assemblies of these plant species. For example, the information on repeat content, heterozygosity and genome sizes provided here may help to tailor new sequencing and genome assembly strategies for these plant genomes. Sequencing data may also be integrated into other genome assemblies. Using the sequencing data obtained from this study would make it easier and more efficient to assemble the remaining sequenced plant genomes. The ~60 Gb data can be used for genome assembly, in combination with either contig reconstruction of second generation-based sequence reads, or for error correction of third-generation long sequence reads. Finally, this dataset may also be used to develop new methods of

257 species identification based either on sequencing data or plant images, and to resolve
258 phylogenetic relationships based on whole genome sequencing data. At present, we
259 have insufficient information to identify all species, so we are building a living plant
260 database that records the position of species grown in the Ruili Botanical Garden and
261 monitors the status of each species [43].
262 In combination with information accumulated in future, deep learning may be applied
263 to this dataset as a training tool to develop plant identification. Indeed, we used data
264 from 175 of the known Ruili species for deep learning, with each sample contributing
265 1 million reads to build the model. At the first trial stage, 181 species have been
266 successfully identified to the species level using our models. By providing this
267 comprehensive, easily and publicly accessible dataset, we believe it would be reused
268 in many ways beyond what has been mentioned here.
269

270 **DISCUSSION**

271 Current understanding of the evolution of plants and their diversity in a phylogenomic
272 context is limited because of the lack of genome-scale information across

phylogenetically diverse species. In this study, we provide a high-depth whole genome sequencing dataset comprising 689 vascular plant species with voucher specimens, covering 137 families and 49 orders. These samples were obtained from Ruili Botanical Garden in Yunnan Province of China, near the border between China and Myanmar, reflecting the rich plant diversity in that region. The data generated here were used to estimate genomic features including genome size, repeat content and heterozygosity, which will be helpful for future studies aiming to establish reference genomes for these species. The dataset may also be used to assemble chloroplast genomes, as well as some conserved nuclear genes, thus providing useful information for evolution and gene function studies.

In this study, we scaled up a whole genome sequencing effort to sequence hundreds of plant species. We only constructed a single short insert library (200 bp) for each species and generated ~60 Gb of whole genome sequencing data. Although it would be insufficient to assemble high-quality genomes for most species based solely on single library data, the current data have potential uses in analyses such as such as gene finder, plastid and mitochondrial assembly. We are now using these data, in

combination with 10x genomics, to obtain high quality genome data for follow on work including looking at wood development.

This study tested, for the first time, the feasibility of large-scale whole genome sequencing, which is already underway for the Earth BioGenome Project [8] and the 10KP project [7]. It also provided experience of plant sampling, sample logistics and management, DNA extraction, sequencing library preparation, sequencing and data analysis and management. Aiming to sequence more than 10,000 plant species, 10KP requires a robust infrastructure for sample and data management, as potentially investigated in this pilot study. We have optimized the DNA extraction protocol and published it via the protocols.io platform [20]. We will soon launch a DNA extraction kit for High Molecular Weight Genomic DNA that is suitable for 10X genomic analysis [16]. We also have just finished writing a guideline on sample submission for 10KP, which includes sample preparation (fresh sample, DNA sample and RNA sample), sample packing and shipping. The specific guidelines will be soon available via the 10KP website [44].

Additional files

Additional file 1

Table S1. List of samples included in this study, with voucher information, current kmer-based estimation of genome sizes, repeat content and heterozygosity. Identified collections were listed with species names, while unidentified collections with only family and order information. Samples with assembled chloroplast genomes (738) are marked with *; 17 samples with assembled unclear genomes are marked with §.

Table S2. The chloroplast genome list used as references for further assembly by MITObim.

Table S3. Gene content information for all assembled chloroplast genomes.

Table S4. Genome information previously measured and publicly available in Plant DNA C-values Database.

Table S5. Summary of preliminary genome assemblies of 17 species of vascular plant.

Table S6. Summary of BUSCO analysis for 17 species of vascular plant.

Additional file 2

Figure S1**.** Phylogeny of vascular plants from the Ruili Botanical Garden. Species tree based on the maximum likelihood analysis of 78 chloroplast genes generated by RAxML. Colors of the inner circle and outer circle represent different families and orders. Clade color represents bootstrap values from red to gray (bootstrap range 50–100).

Figure S2. A comparison of genome sizes measured by experimental approaches to the k-mer estimated genome sizes in this study.

Abbreviations

10KP: 10,000 Plant Genome Project; bp: Base pair; BUSCO: Benchmarking Universal Single-Copy Orthologs; Gb: Gigabase; CNGB: China National GeneBank; ML: Maximum likelihood; WGS: Whole genome sequencing.

Fundings

This work was supported by grants from the Basic Research Program, Shenzhen

Municipal Government, China (grant numbers JCYJ20150529150505656 and JCYJ20150831201643396), as well as funding from the Guangdong Provincial Key Laboratory of Genome Read and Write (grant number 2017B030301011), and The Construction of China National GeneBank (Yunnan province, 2015DA008, P.R. China).

Availability of supporting data

The specimens, leaf samples and DNA solutions of all collections are stored at the CNGB herbarium. The raw sequencing data described in this article are available in the NCBI SRA repository, under the project number PRJNA43840. DNA extraction [16] and BGISEQ-500 WGS library construction protocols can be found via protocols.io [17]. A total of 738 chloroplast genomes and 17 assembled genomes together with raw data supporting the results of this article are available via the *GigaScience* GigaDB repository and will be continuously updated and linked to the GigaDB entries as new assemblies are completed [40].

Competing interests

The authors declare that they have no competing interests.

Author contributions

X.L. conceived this study. X.L. and H.L. drafted the manuscript. H.L. managed the project. J.P.W., X.B.W., L.C., X.F.H., H.C.C., J.L.Y., Y.W., R.C.M., J.L. and J.M.Z. collected the samples. T.Y. lead the identification of voucher specimens. T.Y., W.X.M., B.S., Y.F., Y.C., H.Y.C. analyzed the data. T.Y., X.L.C., M.W., Z.H.H. constructed the phylogenetic tree. G.H.H., W.S.L., H.C.Z., H.C.C., Y.L. extracted DNA and performed genome sequencing. S.K.S. and X.X. revised and edited the manuscript. All the authors have read and approved the final manuscript.

Acknowledgments

The authors would like to express their sincere thanks to the local people and Government of Yunnan province, and the Forestry Institute of Dehong Prefecture for their kind help in sample collections. We would also like to thank the taxonomic

experts at the PE Herbarium (Institute of Botany, Chinese Academy of Sciences) for identification. Finally, we are thankful to the production team of China National GeneBank, Shenzhen, China.

References

1. Pennisi E. Plant biology. Green genomes. Science. 2011;332 6036:1372-5. doi:10.1126/science.332.6036.1372.
2. Bolger ME, Weisshaar B, Scholz U, Stein N, Usadel B and Mayer KF. Plant genome sequencing - applications for crop improvement. Curr Opin Biotechnol. 2014;26:31-7. doi:10.1016/j.copbio.2013.08.019.
3. Desta ZA and Ortiz R. Genomic selection: genome-wide prediction in plant improvement. Trends Plant Sci. 2014;19 9:592-601. doi:10.1016/j.tplants.2014.05.006.
4. NCBI Genomes https://www.ncbi.nlm.nih.gov/genome
5. Kew RBG. The state of the world’s plants report–2016. Royal Botanic Gardens, Kew. 2016.

6. Matasci N, Hung L-H, Yan Z, Carpenter EJ, Wickett NJ, Mirarab S, et al. Data access for the 1,000 Plants (1KP) project. Gigascience. 2014;3 1:17.

7. Lewin HA, Robinson GE, Kress WJ, Baker WJ, Coddington J, Crandall KA, et al. Earth BioGenome Project: Sequencing life for the future of life. Proc Natl Acad Sci U S A. 2018;115 17:4325-33.8.

8. Cheng S, Melkonian M, Smith SA, Brockington S, Archibald JM, Delaux P-M, et al. 10KP: A phylodiverse genome sequencing plan. Gigascience. 2018;7 3:giy013.

9. de Vere N, Rich TC, Trinder SA and Long C. DNA barcoding for plants. Methods Mol Biol. 2015;1245:101-18. doi:10.1007/978-1-4939-1966-6_8.

10. Staats M, Erkens RH, van de Vossenberg B, Wieringa JJ, Kraaijeveld K, Stielow B, et al. Genomic treasure troves: complete genome sequencing of herbarium and insect museum specimens. PLoS One. 2013;8 7:e69189. doi:10.1371/journal.pone.0069189.

11. Osmundson TW, Robert VA, Schoch CL, Baker LJ, Smith A, Robich G, et al. Filling gaps in biodiversity knowledge for macrofungi: contributions and

assessment of an herbarium collection DNA barcode sequencing project. PLoS One. 2013;8 4:e62419. doi:10.1371/journal.pone.0062419.

12. Li X, Yang Y, Henry RJ, Rossetto M, Wang Y and Chen S. Plant DNA barcoding: from gene to genome. Biol Rev Camb Philos Soc. 2015;90 1:157-66. doi:10.1111/brv.12104.

13. Straub SC, Parks M, Weitemier K, Fishbein M, Cronn RC and Liston A. Navigating the tip of the genomic iceberg: Next-generation sequencing for plant systematics. Am J Bot. 2012;99 2:349-64. doi:10.3732/ajb.1100335.

14. Male PJ, Bardon L, Besnard G, Coissac E, Delsuc F, Engel J, et al. Genome skimming by shotgun sequencing helps resolve the phylogeny of a pantropical tree family. Mol Ecol Resour. 2014;14 5:966-75. doi:10.1111/1755-0998.12246.

15. Besnard G, Christin PA, Male PJ, Coissac E, Ralimanana H and Vorontsova MS. Phylogenomics and taxonomy of Lecomtelleae (Poaceae), an isolated panicoid lineage from Madagascar. Ann Bot. 2013;112 6:1057-66. doi:10.1093/aob/mct174.

16. Wu C and Yang T. DNA Extraction for plant samples by CTAB. Protocols.io. 2018; http://dx.doi.org/10.17504/protocols.io.pzqdp5w

17. Gao S, Mu F, Yang Z, Liu X, Jiang H, Liao S, et al. BGISEQ-500 WGS library construction. Protocols.io 2018; http://dx.doi.org/10.17504/protocols.io.ps5dng6

18. Mak SST, Gopalakrishnan S, Carøe C, Geng C, Liu S, Sinding M-HS, et al. Comparative performance of the BGISEQ-500 vs Illumina HiSeq2500 sequencing platforms for palaeogenomic sequencing. Gigascience. 2017;6 8:1-13.

19. Huang J, Liang X, Xuan Y, Geng C, Li Y, Lu H, et al. A reference human genome dataset of the BGISEQ-500 sequencer. Gigascience. 2017;6 5:1-9.

20. Fang C, Zhong H, Lin Y, Chen B, Han M, Ren H, et al. Assessment of the cPAS-based BGISEQ-500 platform for metagenomic sequencing. Gigascience. 2018 Mar 1;7(3):1-8. doi: 10.1093/gigascience/gix133.

21. Patch A-M, Nones K, Kazakoff SH, Newell F, Wood S, Leonard C, et al. Germline and somatic variant identification using BGISEQ-500 and HiSeq X

Ten whole genome sequencing. PloS One. 2018;13 1:e0190264.

22. Dierckxsens N, Mardulyn P and Smits G. NOVOPlasty: de novo assembly of organelle genomes from whole genome data. Nucleic Acids Research. 2016;45 4:e18-e.

23. Arabidopsis thaliana rbcL gene NCBI sequence https://www.ncbi.nlm.nih.gov/nuccore/U91966.1/

24. Hahn C, Bachmann L and Chevreux B. Reconstructing mitochondrial genomes directly from genomic next-generation sequencing reads—a baiting and iterative mapping approach. Nucleic Acids Research. 2013;41 13:e129-e.

25. Liu H, Wei J, Yang T, Mu W, Song B, Yang T, et al. Genomic and Imaging Data Supporting the Digitization of Ruili Botanical Garden. GigaScience Database. 2019; http://dx.doi.org/10.5524/100502

26. Wyman SK, Jansen RK and Boore JL. Automatic annotation of organellar genomes with DOGMA. Bioinformatics. 2004;20 17:3252-5. doi:10.1093/bioinformatics/bth352.

27. Birney E, Clamp M and Durbin R. GeneWise and Genomewise. Genome Res.

2004;14 5:988-95. doi:10.1101/gr.1865504.

28. Hsu CY, Wu CS, Surveswaran S and Chaw SM. The complete plastome sequence of Gnetum ula (Gnetales: Gnetaceae). Mitochondrial DNA A DNA Mapp Seq Anal. 2016;27 5:3721-2. doi:10.3109/19401736.2015.1079874.

29. Stamatakis A. RAxML version 8: a tool for phylogenetic analysis and post-analysis of large phylogenies. Bioinformatics. 2014;30 9:1312-3.

30. Nguyen L-T, Schmidt HA, von Haeseler A and Minh BQ. IQ-TREE: a fast and effective stochastic algorithm for estimating maximum-likelihood phylogenies. Molecular biology and evolution. 2014;32 1:268-74.

31. Katoh K, Misawa K, Kuma K and Miyata T. MAFFT: a novel method for rapid multiple sequence alignment based on fast Fourier transform. Nucleic Acids Res. 2002;30 14:3059-66.

32. Capella-Gutiérrez S, Silla-Martínez JM and Gabaldón T. trimAl: a tool for automated alignment trimming in large-scale phylogenetic analyses. Bioinformatics. 2009;25 15:1972-3.

33. Chase MW. Monocot relationships: an overview. Am J Bot. 2004;91

10:1645-55. doi:10.3732/ajb.91.10.1645.

34. Bremer K, Backlund A, Sennblad B, Swenson U, Andreasen K, Hjertson M, et al. A phylogenetic analysis of 100+ genera and 50+ families of euasterids based on morphological and molecular data with notes on possible higher level morphological synapomorphies. Plant Systematics and Evolution. 2001;229 3-4:137-69.

35. Refulio-Rodriguez NF and Olmstead RG. Phylogeny of lamiidae. American Journal of Botany. 2014;101 2:287-99.

36. Liu B SY, Yuan J, Hu X, Zhang H, Li N, Li Z, Chen Y, Mu D, Fan W. Estimation of genomic characteristics by analyzing k-mer frequency in de novo genome projects. arXiv preprint. 2013; arXiv:1308.2012.

37. Chikhi R and Medvedev P. Informed and automated k-mer size selection for genome assembly. Bioinformatics. 2014;30 1:31-7. doi:10.1093/bioinformatics/btt310.

38. Kew C values website http://data.kew.org/cvalues/

39. Luo R, Liu B, Xie Y, Li Z, Huang W, Yuan J, et al. SOAPdenovo2: an

empirically improved memory-efficient short-read de novo assembler. Gigascience. 2012;1 1:18.

40. Simão FA, Waterhouse RM, Ioannidis P, Kriventseva EV and Zdobnov EM. BUSCO: assessing genome assembly and annotation completeness with single-copy orthologs. Bioinformatics. 2015;31 19:3210-2.

41. Ruili Garden genome project NCBI bioproject's webpage https://www.ncbi.nlm.nih.gov/bioproject/?term=PRJNA438407

42. CNGB Nucleotide Sequence Archive (CNSA) Website https://db.cngb.org/cnsa

43. Ruili Garden website http://720yunnan.com/tour/a2b8096d43d7226d?scene=scene_d3627cc2a43314d

44. 10KP Website https://db.cngb.org/10kp/

Figure legends

Figure 1. Sampling localities of this project.

Sampling was conducted mainly in Ruili Botanical Garden in Southwest China, near

the China–Myanmar border, shown in red circles.

Figure 2. Phylogeny of vascular plants of the Ruili Botanical Garden based on the maximum likelihood (ML) analysis tree of 78 chloroplast genes. Colors in the inner circle represent different families, and colors in the outer circle represent different orders.

Figure 3. Ordinal phylogeny of vascular plants of the Ruili Botanical Garden based on 'drop-tips' from Figure 2. Based on the species-level phylogenetic tree, we used the drop.tip function in the Ape package (version 5.2) to remove the corresponding internal branches. (a) The genome sizes in Gb. (b) Repeat content as percentage of total genome (%), and (c) The cladogram of the heterozygosity ratio based on 78 chloroplast genes by maximum likelihood (ML) phylogeny using only one tip per order.

Tables

Table 1. Summary of the sequencing data produced in this study

Table 1. Summary of the sequencing data in this study

Order	Raw base (Gb)	Raw data GC (%)	Raw data Q20	Raw data Q30
Alismatales	66.3873	43.64	95.34	86.48
Apiales	70.0075	35.42	96.40	88.40
Araucariales	74.14	32.87	96.50	88.85
Arecales	68.8318	39.95	95.84	87.20
Asparagales	70.3465	37.97	96.16	87.87
Asterales	67.8382	37.41	95.83	87.20
Brassicales	68.474	37.89	95.99	87.45
Buxales	65.44	42.34	95.38	86.00
Caryophyllales	68.6558	38.04	95.73	87.03
Celastrales	75.8133	38.12	96.56	88.57
Commelinales	65.02	36.80	95.58	86.81
Cornales	76.396	36.49	96.44	88.63
Crossosomatales	60.2	37.17	95.36	86.54
Cucurbitales	65.11	35.73	95.50	86.22
Cupressales	73.54	36.12	96.78	89.01
Cyatheales	75.76	41.32	96.64	88.37
Dioscoreales	78.9	41.47	94.99	85.65
Dipsacales	58.6267	37.58	96.22	87.52
Equisetales	67.3	39.98	94.92	84.77
Ericales	68.1109	38.01	96.46	88.02
Fabales	69.9439	35.50	96.14	87.75
Fagales	68.14	36.81	96.13	87.90
Gentianales	70.1155	36.49	96.36	88.27
Gnetales	71.1267	39.77	96.87	89.24
Lamiales	69.3291	37.47	95.94	87.40
Laurales	71.9425	40.22	96.04	87.83
Liliales	71.4133	41.00	96.73	89.15
Magnoliales	69.0988	38.88	96.12	88.01
Malpighiales	68.1842	35.83	96.40	88.23
Malvales	66.2106	37.19	96.26	88.07
Myrtales	70.7924	38.82	96.23	88.20
Oxalidales	68.3533	34.91	95.61	87.20
Pandanales	72.6733	42.07	96.41	88.31
Pinales	61.04	39.56	93.91	82.96
Piperales	63.2533	40.50	96.23	87.84
Poales	69.6407	44.07	95.56	86.73
Polypodiales	68.588	41.39	96.12	87.69
Proteales	69.0733	39.47	96.49	88.23
Ranunculales	67.5644	38.69	95.68	86.80

Rosales	70.0468	36.72	96.36	88.18
Santalales	69.07	38.11	96.47	88.31
Sapindales	70.5628	36.83	96.14	87.89
Saxifragales	70.84	37.74	96.77	89.36
Schizaeales	62.57	43.84	96.83	89.17
Solanales	72.2389	38.38	96.30	87.93
Vitales	65.235	39.17	95.44	86.71
Zingiberales	67.4956	40.57	95.99	87.51

Figure 1

Click here to access/download;Figure;Figure 1.pdf

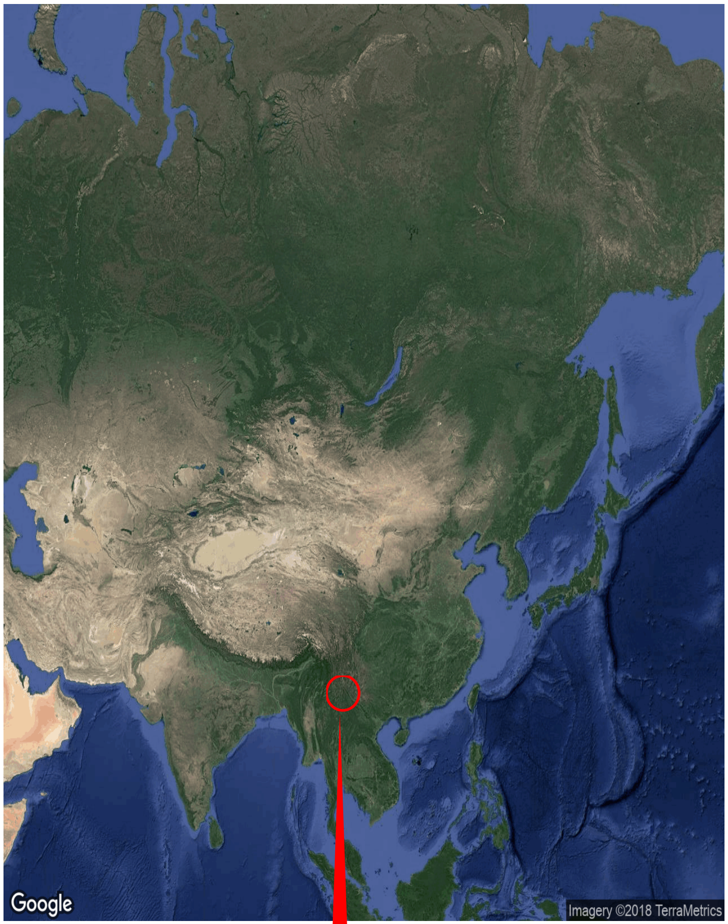

Family	Order
Dipterocarpaceae	Malvales

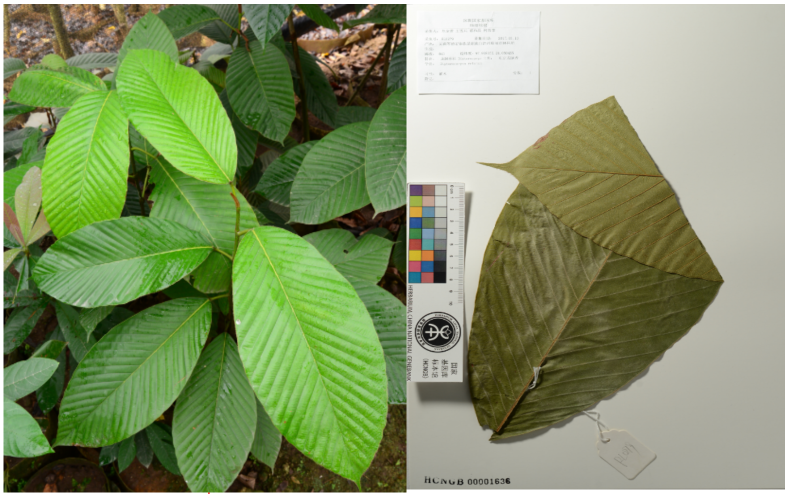

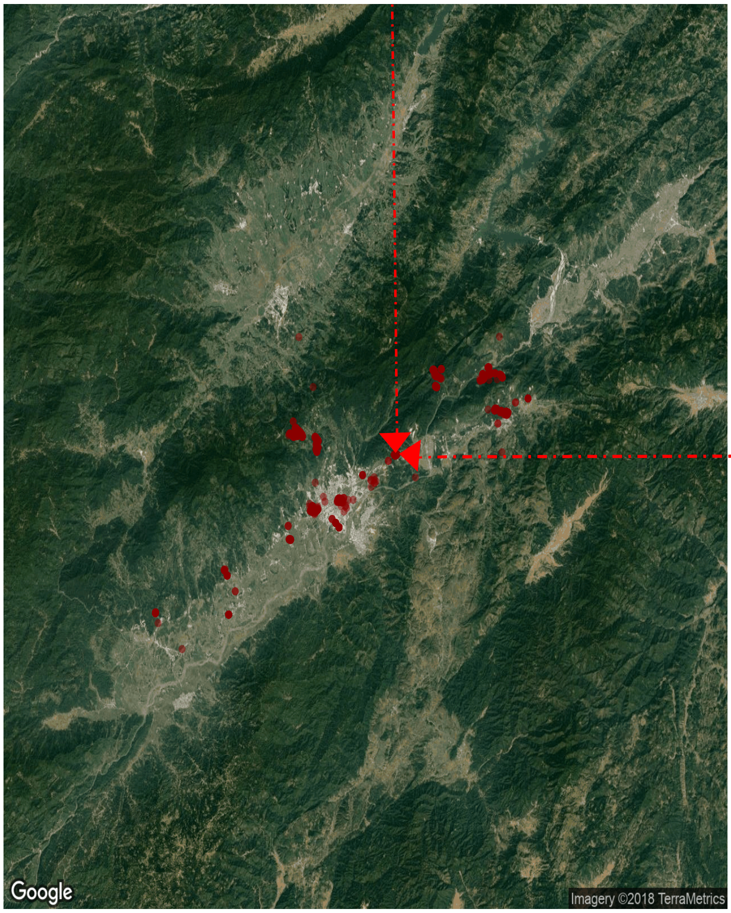

Family	Order
Nyssaceae	Cornales

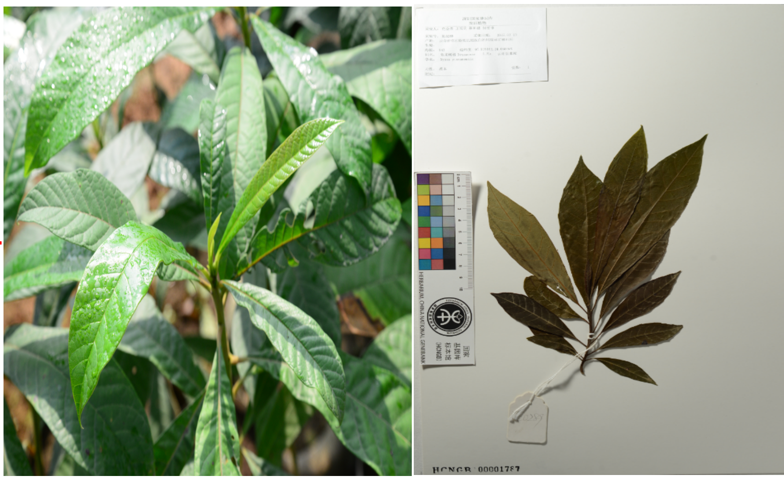

Figure 2

Click here to access/download;Figure;Figure 2.pdf

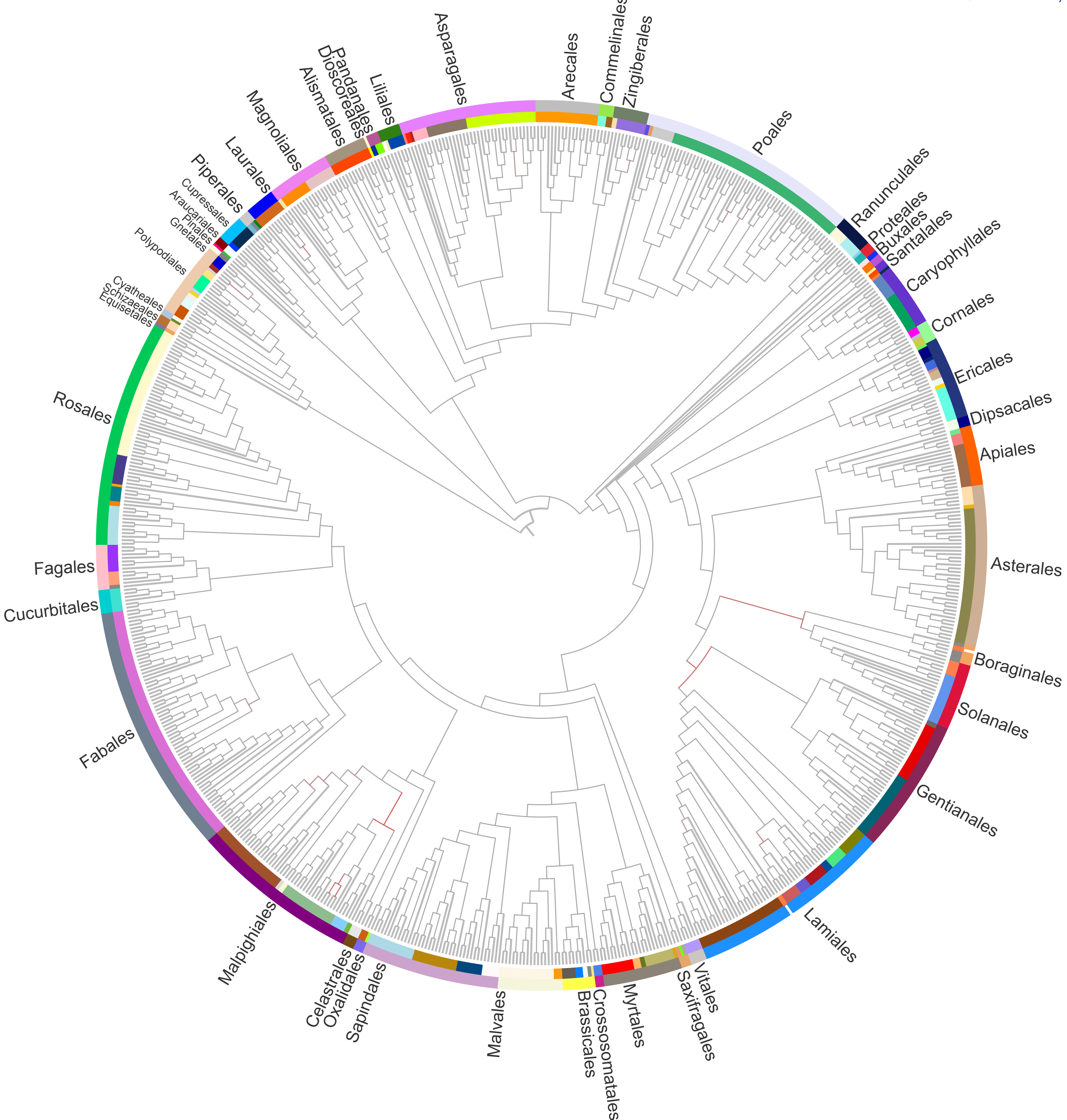

Figure 3

Click here to access/download;Figure;Figure 3.pdf

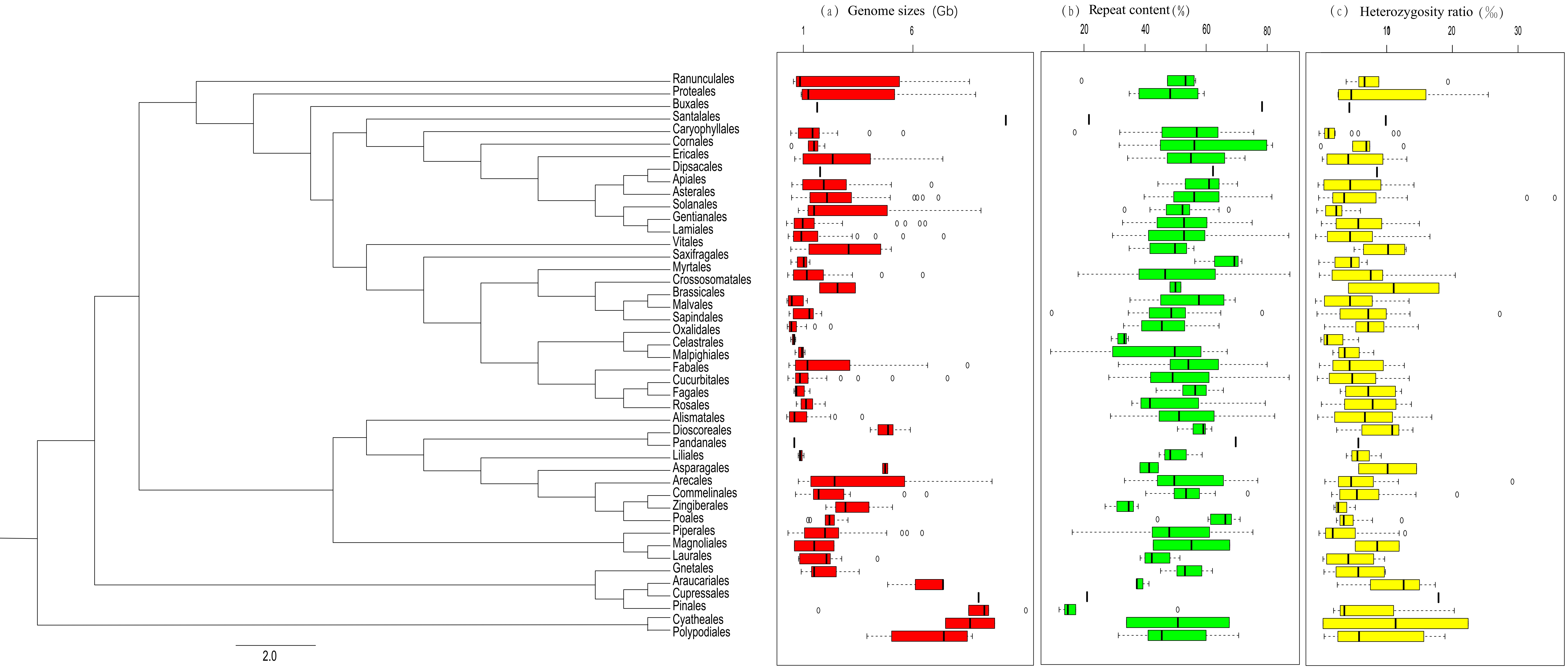

Supplementary Table S1

Click here to access/download
Supplementary Material
Table S1.xlsx

Supplementary Table S2

Click here to access/download
Supplementary Material
Table S2.xlsx

Supplementary Table S3

Click here to access/download
Supplementary Material
Table S4.xls

Supplementary Table S4

Click here to access/download
Supplementary Material
Table S5.xlsx

Supplementary Table S5

Click here to access/download

Supplementary Material

Table S6.xlsx

Supplementary Table S6

Click here to access/download

Supplementary Material

Table S7.xlsx

Supplementary Material Figure S1

Click here to access/download
Supplementary Material
Figure S1.pdf

Supplementary Material Figure S2

Click here to access/download
Supplementary Material
Figure S2.pdf

Personal Cover Click here to access/download;Personal Cover;cover_letter.docx

Dear Dr. Scott,

Sub: Submission of the re-revised manuscript GIGA-D-18-00121R3.

We are glad to submit the thoroughly revised version of our manuscript entitled “Molecular Digitization of a Botanical Garden: High-depth whole genome sequencing of 689 vascular plant species from the Ruili Botanical Garden”

We look forward to hearing from you at your earliest convenience.

Yours sincerely,

Xin Liu
