# Supplementary figures and images for: Molecular digitization of a botanical garden: high-depth whole-genome sequencing of 689 vascular plant species from the Ruili Botanical Garden
Source: Gigascience. 2019 Jan 25;8(4):giz007. doi: 10.1093/gigascience/giz007 (PMC6441391; doi:10.1093/gigascience/giz007)

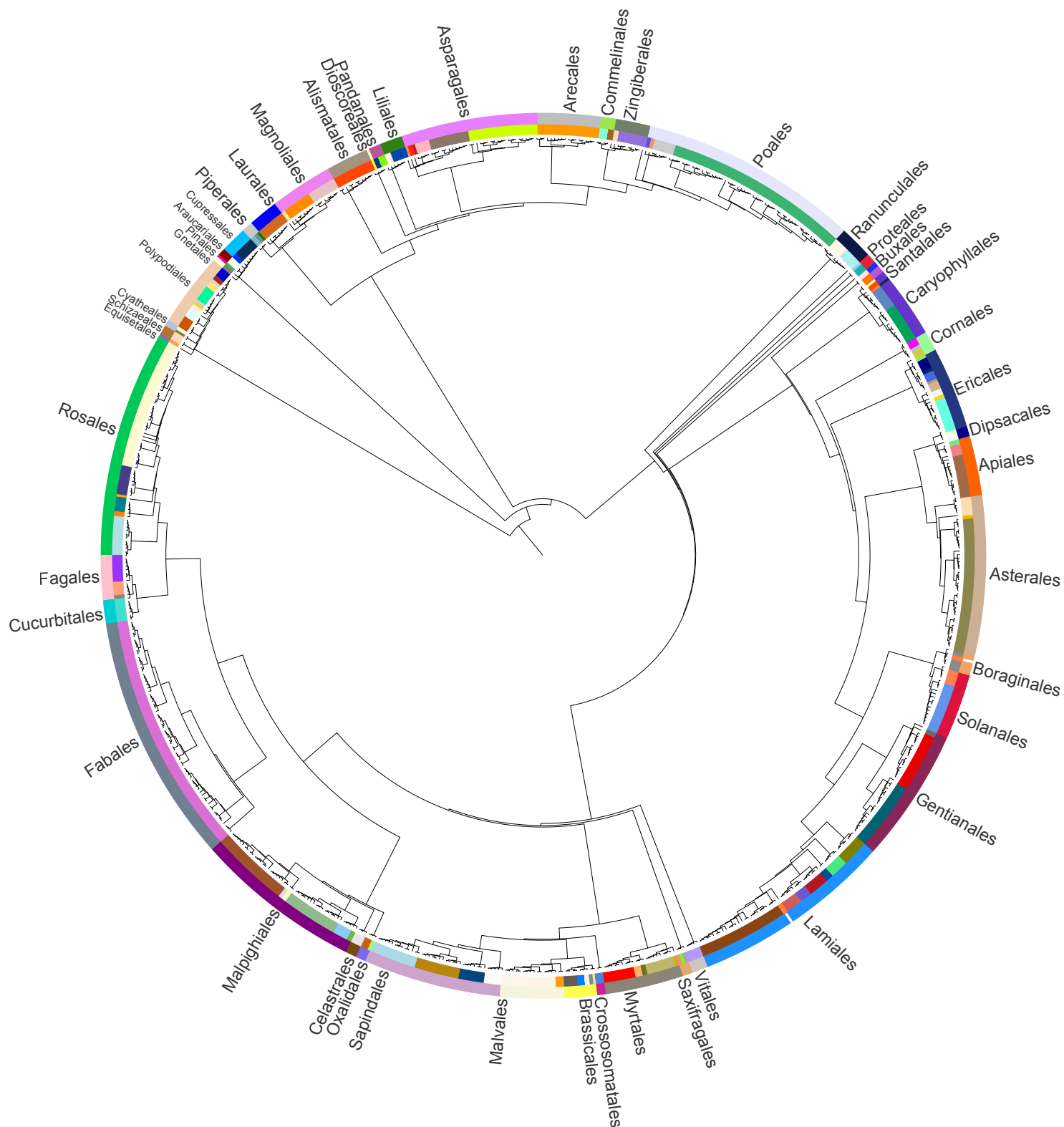

Supplement: Supplemental Files [file giz007_supplemental_files.zip › Figure S1.pdf]
