# Supplementary material for: Molecular digitization of a botanical garden: high-depth whole-genome sequencing of 689 vascular plant species from the Ruili Botanical Garden
Source: Gigascience. 2019 Jan 25;8(4):giz007. doi: 10.1093/gigascience/giz007 (PMC6441391; doi:10.1093/gigascience/giz007)
Supplement: Response_to_Reviewer_Comments_Original_Submission.pdf [file giz007_response_to_reviewer_comments_original_submission.pdf]

Author's Response To Reviewer Comments

Close

Reviewer reports:

Reviewer #1: The authors present an awesome quantity of whole genome sequence data representing hundreds of plant species. That the data is being made openly available represents a substantial contribution to the research community. Clearly the analysis of the data is a work in progress. Only 17 of the samples are subjected to whole genome assembly; for 738 just the chloroplast genomes were assembled. The sampling strategy was not exactly ideal: the plants were collected from a single botanic garden, and as such you might expect a somewhat arbitrary selection of species; given the numbers involved and the intent of the study this is perhaps not so important. What is more surprising is that the identifications of the plants is so limited. If these are part of a curated collection, you might expect them to be determined at least to genus; if it represents wild-collected specimens, you would ideally consult (local) botanical expertise for more precise determinations. Certainly for this kind of study it would increase the value of the data markedly to know from what organisms the sequences originated. Since there are voucher specimens lodged in herbaria, and it seems considerable resources will be available - images etc. - the identifications can presumably still be achieved and the database updated. It just seems a bit odd; but if the paper is viewed as the presentation of a vast quantity of usable semi-raw data then perhaps this is OK too. I have not assessed the data itself and have no great insight to provide into the bioinformatic methods - they look superficially pretty standard. The authors have compared previously known genome sizes to that inferred from their data and it appears similar; although only 17 of the genomes were assembled (those with lower heterozygosity and repeats; apparently assemblies of more will be added later) those each included around 90% of benchmarking universal single-copy orthologs. Since the data, from raw reads
onwards should be available, the community will be in a position to assess it in detail post publication.

1. "We then compared these assembled chloroplast genomes and constructed the phylogenetic tree using the gene trees and translated to the species tree"
You have used astral to infer a species tree from the plastid data? This seems inappropriate in principle; the data ought to be linked and could be combined e.g. under ML to infer a tree without using a coalescence-based method. Plus, if there is conflict in this dataset, coalescence is not the best way to model it I would argue.
Response:
Thank you for the nice suggestion. Yes, we used ASTRAL to infer a species tree in the earlier version of this article. We also combined all genes to super data and used two methods for tree construction, one is by RAxML using GTRCAT model, another one is by IQ_TREE using the best model (GTR+F+R10) to construct the species tree. We have compared these two results in the revised article.

2. The conclusion towards the end of the data description (although it instead feels like discussion material): "deep learning can be applied to develop plant identification using this

dataset as a good training set" seems a little far-fetched in this context. Huge as it is, I'm not convinced that this dataset represents useful input for this kind of application - the plants would need to be reliably determined, at the least, and I believe image-based IDs need a great deal more training data.

Response:
Thank you for the suggestion. We have now revised the sentence as per your suggestion as follows:

3.Discussion:
"The evolution of vasculature was a major event in plant history."
This is not directly relevant - I'd suggest to consider an alternative leading sentence here.

Response:
Thank you for the suggestion. We have removed this sentence from the main text and replaced with “The current understanding on the evolution of plants and its diversity in a phylogenomic context is limited due to the non-availability of genome-scale information across phylogenetically diverse species.”

4. "We only constructed a single short insert size library for each of the species and generated ~60 Gb whole genome sequencing data. It would be insufficient to assemble good draft genomes for majority of the species just based on single library data, because previous efforts to assemble reference genomes based on second generation sequencing data would require multiple short insert size libraries and also mate pair (large insert size) libraries. However,"
I don't understand what you're saying here. Are you saying that it would not have been possible to assemble genomes from much of the current data (surely not?)? Or are you suggesting that something has changed and that although in the past it would not have been possible, now it is? The lack of long reads marks a difference between this effort and that proposed in the 10KP project; how are the data likely to be comparable?
Response:
Yes, we do agree with your comments. Though a single library can be used to assemble a genome, but may not yield a high-quality genome assembly. However, the current data was potentially used for several analyses such as gene finder, plastid and mitochondrial assembly. At present, we are using these data in combination with 10x genomics to get high quality genome data.

5. "With particular relevance for the 10KP, this study provided experiences for plant sampling, sample logistics, sample management, DNA extraction, sequencing library preparation, sequencing, data analysis and data management."
What exactly was learnt about all this? Qualification needed here - I have no doubt the authors gained a great deal of insight in the process of generating this huge dataset and it would be well worth sharing.
Response:
We have optimized the DNA extraction protocol and also published the protocol (Wu and Yang, 2018). Soon we will also launch the DNA extraction kit.

Wu C and Yang T. DNA Extraction for plant samples by CTAB. Gigascience. 2018; doi:10.17504/protocols.io.pzqdp5w

We also have just finished a guideline about sample submission for 10KP which including sample preparation (fresh sample, DNA sample and RNA sample), sample packing and shipping. The specific guidelines will be soon available in our 10KP website (https://db.cngb.org/10kp/).

6. "DNA Extraction [27] and BGISEQ-500 WGS library construction protocols can be found in protocols.io [28]."
Are these the appropriate references...?
Response: Yes, these are valid and appropriate references.

Table 1: does it really make sense to summarize this by order? Surely the quantities of raw data are only comparable if you break that down to families/genera/species/individuals?
Response:
Thank you for raising this question. For every sample, the sequencing quality and results were highly similar. Hence, we summarized the data by "Order" in Table 1. However, the detailed breakdown of the data at families/genera/species/ level will presented in our subsequent manuscript with higher quality genome assemblies for the selected species.

Reviewer #2: This paper sequences and assembles the genomes of diverse plant species found at the Ruili Botanical Garden, China. The authors generate approximately 60 Gb short sequence read data from 760 samples, and perform a range of analyses suitable for low-coverage draft genomes such as repeat content characterization and plastid assembly. These analyses show considerable variation in many genomic properties across plants. While the paper is descriptive, and is limited by using the same sequencing approach and assembly parameters for all samples, I do think it is impressive in terms of the scale of sequencing, and will serve as a good test case for large-scale sequencing of diverse sample sets.

Major comments
1. This paper is likely to be used as a test case for sequencing diverse samples across a flora. As such, I'm sure the reader would like to know the relationship between coverage and contiguity in the assemblies. Also, were mitochondrial genome assemblies attempted, and were these successful? More generally, it would be useful to explain why the same sequencing effort was allocated to all samples regardless of known genome size (and would you suggest others follow this approach in the future?).
Response
In this article, we have mentioned that ~70 Gb of raw sequencing data was generated for each of the sample. Combined with estimated genome size by GCE and kmergenie we can infer the genome coverage. For mitochondrial genome, we completed the initial test run, and successfully assembled the mitochondrial genome of five Species. Based on these positive assemblies, we have now actually initiated the mitochondrial genome assembly of remaining species.

To calculate the genome size, only 10-30X data is usually enough, that’s why we sequenced ~70 Gb as the preliminary step. Of course, for some samples with large genome size, we couldn’t estimate the genome size, but their sequencing data was used in assembling the chloroplast genomes, as well as some conserved nuclear genes. Hence, once we obtained the genome size information, then we selected the appropriate method for the genome assembly. Overall, based on our experience, the sequencing method employed in this study can be recommended for the smaller genomes only.

2. As far as I'm aware, Astral is a species tree approach that should be used on many independent loci. I don't think it's suitable for the plastid as all loci are linked. An alternative phylogenetic approach should be used (such as partitioned analysis in IQ-TREE).
Response
Yes, we agree with your suggestion that Astral is not a suitable method for our analysis. We now combined all genes to super data and used two different methods for the tree construction, one is by RAxML using GTRCAT model, another one is by IQ_TREE using the best model to construct the species tree. We have compared these two results in the revised article.

3. The quality of the writing and clarity of some sentences could be improved.
Response:
Thank you for the suggestion. We have thoroughly revised the manuscript, and also followed the specific suggestions given by Reviewer#3 on writing part. In addition, our manuscript was proof read by a native English speaker.

4. This is the first time I've seen repeat content and heterozygosity summarised across such a broad sample set, and I'd be interested in more interpretation of these results. Presumably you'd consider these to be extremely labile across plants? Given that lability, I wonder the suitability of summarising this across species within a family (as shown in the box plots)?
Response:
We have summarized the information on repeat content and heterozygosity for every individual, both in the result section as well in the Table S1.

Minor comments

5. Line 52. Next generation sequencing technologies?
Response: We have revised the sentence as “With the advent of next generation sequencing technologies, enormous efforts have been made to sequence whole genomes of plant species, thereby providing new insights on plant evolution [1]”

6. Line 81. Genome size and repeat content estimates are possible from genome skim data (for example, see RepeatExplorer), so this critique doesn't seem fair.
Response: Thank you for the suggestion. We do agree with your comment that “repeat content estimates are possible from the genome skim data via RepeatExplorer program”. However, Genome size estimation is only possible for the plants with small genome size. Anyway, we have revised the sentence to avoid the confusion as follows:
“However, previous genome skimming studies have only generated a small amount of sequencing data for the individual species, precluding the re-use of the data to reveal more

detailed genome features including genome sizes (for plant with large genome size), ploidy level etc., or its direct usage in the further de novo genome assembly.”

7. Line 85: A major challenge for diverse genome sequencing projects like this one is choosing whether to sequence samples in accordance to their genome size, or whether to use a 'one size fits all' approach with similar amounts of data for all samples. It would be valuable to justify this approach, and say the range of coverage this amount of data generates (see main comment 1, above).
Response: It’s really a good question indeed. As you mentioned, it was a major question cum challenge for us to select the best sequencing approach for all the samples. In continuation to our response to the “comment 1”, this project is part of 10KP project, and being the starting point of this mega project, we wanted to first evaluate our existing sequencing strategy according to 'one size fits all' approach. On an average we generated 60X data per sample.

8. Line 106: The details of DNA extraction are very brief. Was the same DNA extraction protocol used for all species? Were there any modifications?
Response: We first optimized the CTAB DNA extraction protocol and found it suitable for most of the tested species. That’s the reason we used the same DNA extraction (Wu and Yang, 2018) method for all the species. The detailed protocol is available via GigaScience protocols.

Wu C and Yang T. DNA Extraction for plant samples by CTAB. GigaScience. 2018; doi:10.17504/protocols.io.pzqdp5w

9. Line 107: BGISEQ is not a very established sequencing platform relative to other platforms (such as Illumina). A brief description of this technology would be useful.
Response: BGISEQ-500 is a desktop sequencer developed by our Institute BGI-Shenzhen in 2015. Using DNA nanoball and combinational probe anchor synthesis developed from Complete Genomics™ sequencing technologies, it generates short reads at a large scale. The sequencing outputs are comparable with the Illumina series (Mak et al 2017), and has been successfully utilized to sequence the plant genome (Chang et al 2018), human genome (Huang et al 2017), and metagenomes (Fang et al 2017)

Mak, S. S. T., Gopalakrishnan, S., Carøe, C., Geng, C., Liu, S., Sinding, M. H. S., ... & Germonpré, M. (2017). Comparative performance of the BGISEQ-500 vs Illumina HiSeq2500 sequencing platforms for palaeogenomic sequencing. GigaScience, 6(8), 1-13.

Huang, J., Liang, X., Xuan, Y., Geng, C., Li, Y., Lu, H., ... & Sun, N. (2017). A reference human genome dataset of the BGISEQ-500 sequencer. Gigascience, 6(5), 1-9.

Fang, C., Zhong, H., Lin, Y., Chen, B., Han, M., Ren, H., ... & Stein, S. (2017). Assessment of the cPAS-based BGISEQ-500 platform for metagenomic sequencing. GigaScience, 7(3), gix133.

10. Line 108: What type of libraries were prepared? What was the insert size?
Response: The 100bp pair end libraries were prepared for the sequencing, and the insert size was 200 bp.

11. Line 110. Repetition of 100bp PE from the previous line.
Response: We have now deleted the repetitive information.

12. Line 166: Define which repeats are being counted.
Response: We didn't assemble all the genomes, and doesn't used the RepeatExplorer program for our analysis. That's why we just used the Kmer data plot to identify the repeat ratio in the sequenced genomes. The specific repeat types will be classified in our subsequent manuscript, once we finish the assembly of all the genomes.

Line 183: It's unclear to me how heterozygosity was scored. Were reads mapped back to the initial assembly then heterozygous sites scored?
Response: The heterozygosity was scored based on the Kmer distribution plot according to the method described by Liu et al (2013). The same description is now mentioned in the main text also.

13. Line 255: Evolution of vasculature is not really relevant to this study.
Figure legends lack detail, e.g. details of the phylogenetic reconstruction used in Figure 2.
Response:
The ordinal phylogeny in Figure 2 was "drop-tips" from Figure 3.

14. Reference 4 seems incomplete.
Response: Thanks for your careful observation. We have now updated the reference for the book chapter.

Reviewer #3: This manuscript reports the whole genome sequencing of more than 600 hundred vascular plant samples collected at the Ruili Botanical Garden in China. The data generated here makes an enormous contribution to the study of plant genomes of non-model plants by generating raw sequencing data, along with images, voucher information, complete chloroplast genomes and several partial nuclear genomes assembled. The supplementary data is essential to understand the scope of this project, and make processed data (i.e. genome assemblies) available.
My main comments, suggestions and edits are included in the attached word document. They include a few questions to clarify the analyses performed, as well as suggestions to make the language clearer.

Response:
Thank you for your great suggestions and kind recommendation. We have thoroughly updated the manuscript (with track changes) as per your suggestions. Kindly refer the enclosed manuscript.

Close
