# Supplementary material for: Molecular digitization of a botanical garden: high-depth whole-genome sequencing of 689 vascular plant species from the Ruili Botanical Garden
Source: Gigascience. 2019 Jan 25;8(4):giz007. doi: 10.1093/gigascience/giz007 (PMC6441391; doi:10.1093/gigascience/giz007)
Supplement: Response_to_Reviewer_Comments_Revision_1.pdf [file giz007_response_to_reviewer_comments_revision_1.pdf]

Author's Response To Reviewer Comments

Close

Dear Dr. Scott,

We are glad to submit the thoroughly revised version of our manuscript entitled "Molecular Digitization of a Botanical Garden: High-depth whole genome sequencing of 689 vascular plants from the Ruili Botanical Garden".

The comments of the reviewers were highly insightful and enabled us to greatly improve the quality of our manuscript. According to their advice, we have carefully revised our manuscript. We present the point-by-point responses to each of the comments and suggestions of the reviewer, we also made a substantial revision in the manuscript with track changes. However, we have uploaded both clean and track changes version for your kind perusal. We strongly believe that these revisions in the manuscript and our accompanying responses are sufficient to make our manuscript suitable for publication in GigaScience.

Reviewer reports:
Reviewer #1:
1) I reviewed the previous version of this submission and am pleased to see that most of the reviewers' comments have been used effectively. My only reservations would remain the low proportion of precise identifications of the samples used, and I trust that the available resources - images; specimens - will be sufficient to address this as the data are further processed and used.
Response: Your comments in the earlier version of the manuscript were really useful, and enabled us to greatly improve the quality of our manuscript. Yes, the genomics data generated in this study makes an enormous contribution to the study of plant genomes of non-model plants along with images, voucher information, complete chloroplast genomes and nuclear genomes. We agree with your concerns, but as mentioned in the manuscript, all the information will be continuously updated and linked to the GigaDB repository as new assemblies are completed.

2) I have been through the text and made a number of suggestions for minor revisions and a few comments which are included in a tracked changes version of the word document. I hope these will be useful and look forward to seeing this impressive piece of work published in due course.

Response:
Thank you for your great suggestions and kind recommendation. We have thoroughly updated the manuscript (with track changes) as per your suggestions. Kindly refer the enclosed manuscript.

Reviewer #2:
The authors have done the right thing and removed the Astral analysis, which was inappropriate for this data. Their new analysis is better, though personally I would raise the following questions:

1. It is very unusual that only 18 conserved genes are found across plastids. In general plastid gene content is highly conserved across plant species. I would suggest the few samples without a standard plastid gene complement are removed and the phylogeny rerun with as many loci as possible. Alternatively, more details could be given as to this issue, perhaps in the Supplementary Information. For example, is it actually gene loss, or are there issues aligning divergent loci?
Response：Yes, we do agree that the plastid gene content of green plant is highly conserved, but the gene loss was observed in several clades especially in parasitic plants and gymnosperm. For instance, in our study, Gnetales, Pinales lost all ndh and rps genes, Gnetaceae only contained 45 genes. To avoid the confusion, we have now changed the sentence "only 18 conserved genes are found across plastids" to "only 18 genes were found to be consistently present among all the plastid genomes". The gene content information is listed in supplementary table 3.

2. A single model of molecular evolution is used across loci. It would be much better to use model testing on each locus and rerun the analysis using partitions.

Response: To refer the phylogeny of green plants, Brad et al. (2014) [1] used 360 plastid genomes and partitioned the nucleotide data into four data sets with the GTR+Γ model. They found that the average support values among all internal nodes in the ML trees were slightly higher in the ntAll phylogeny, and ntAll phylogeny also had the most clades resolved with ≥70% BS. In our study, we used IQ-TREE with best model GTR+F+R10 to construct the tree. According to your advice, we partitioned all nt data set to 76 groups of sites based on gene content, IQ-TREE edge-linked-equal partition model. But, separate models between partitions were used with command -m "GTR+I+G". However, to our surprise, the phylogenetic tree was the same as our previous result. Please see the enclosed phylogenetic tree for your kind perusal.

We look forward to hearing from you at your earliest convenience.
Yours sincerely,
Xin Liu

Close
