# Supplementary material for: Molecular digitization of a botanical garden: high-depth whole-genome sequencing of 689 vascular plant species from the Ruili Botanical Garden
Source: Gigascience. 2019 Jan 25;8(4):giz007. doi: 10.1093/gigascience/giz007 (PMC6441391; doi:10.1093/gigascience/giz007)
Supplement: Response_to_Reviewer_Comments_Revision_2.pdf [file giz007_response_to_reviewer_comments_revision_2.pdf]

Author's Response To Reviewer Comments

Close

Dear Dr. Scott,

We are glad to submit the thoroughly revised version of our manuscript entitled “Molecular Digitization of a Botanical Garden: High-depth whole genome sequencing of 689 vascular plant species from the Ruili Botanical Garden”

We look forward to hearing from you at your earliest convenience.
Yours sincerely,
Xin Liu

Note: No comments by the reviewers

Close
