# Supplementary material for: Molecular digitization of a botanical garden: high-depth whole-genome sequencing of 689 vascular plant species from the Ruili Botanical Garden
Source: Gigascience. 2019 Jan 25;8(4):giz007. doi: 10.1093/gigascience/giz007 (PMC6441391; doi:10.1093/gigascience/giz007)
Supplement: Reviewer_1_Report_Original_Submission -- Michael Pirie — 9/27/2018 Reviewed [file giz007_reviewer_1_report_original_submission.pdf]

Reviewer Report

Title: Molecular Digitization of a Botanical Garden: High-depth whole genome sequencing of 689 vascular plant species from the Ruili Botanical Garden

Version: Original Submission **Date:** 9/27/2018

Reviewer name: Michael Pirie

Reviewer Comments to Author:

The authors present an awesome quantity of whole genome sequence data representing hundreds of plant species. That the data is being made openly available represents a substantial contribution to the research community. Clearly the analysis of the data is a work in progress. Only 17 of the samples are subjected to whole genome assembly; for 738 just the chloroplast genomes were assembled. The sampling strategy was not exactly ideal: the plants were collected from a single botanic garden, and as such you might expect a somewhat arbitrary selection of species; given the numbers involved and the intent of the study this is perhaps not so important. What is more surprising is that the identifications of the plants is so limited. If these are part of a curated collection, you might expect them to be determined at least to genus; if it represents wild-collected specimens, you would ideally consult (local) botanical expertise for more precise determinations. Certainly for this kind of study it would increase the value of the data markedly to know from what organisms the sequences originated. Since there are voucher specimens lodged in herbaria, and it seems considerable resources will be available - images etc. - the identifications can presumably still be achieved and the database updated. It just seems a bit odd; but if the paper is viewed as the presentation of a vast quantity of usable semi-raw data then perhaps this is OK too. I have not assessed the data itself and have no great insight to provide into the bioinformatic methods - they look superficially pretty standard. The authors have compared previously known genome sizes to that inferred from their data and it appears similar; although only 17 of the genomes were assembled (those with lower heterozygosity and repeats; apparently assemblies of more will be added later) those each included around 90% of benchmarking universal single-copy orthologs. Since the data, from raw reads onwards should be available, the community will be in a position to assess it in detail post publication.
Some detailed comments:
"We then compared these assembled chloroplast genomes and constructed the phylogenetic tree using the gene trees and translated to the species tree"
You have used astral to infer a species tree fom the plastid data? This seems inappropriate in principle; the data ought to be linked and could be combined e.g. under ML to infer a tree without using a coalescence-based method. Plus, if there is conflict in this dataset, coalescence is not the best way to model it I would argue.
The conclusion towards the end of the data description (although it instead feels like discussion material): "deep learning can be applied to develop plant identification using this dataset as a good training set" seems a little far-fetched in this context. Huge as it is, I'm not convinced that this dataset represents useful input for this kind of application - the plants would need to be reliably determined, at

the least, and I believe image-based IDs need a great deal more training data.
Discussion:
"The evolution of vasculature was a major event in plant history."
This is not directly relevant - I'd suggest to consider an alternative leading sentence here.
"We only constructed a single short insert size library for each of the species and generated ~60 Gb whole genome sequencing data. It would be insufficient to assemble good draft genomes for majority of the species just based on single library data, because previous efforts to assemble reference genomes based on second generation sequencing data would require multiple short insert size libraries and also mate pair (large insert size) libraries. However,"
I don't understand what you're saying here. Are you saying that it would not have been possible to assemble genomes from much of the current data (surely not?)? Or are you suggesting that something has changed and that although in the past it would not have been possible, now it is? The lack of long reads marks a difference between this effort and that proposed in the 10KP project; how are the data likely to be comparable?
"With particular relevance for the 10KP, this study provided experiences for plant sampling, sample logistics, sample management, DNA extraction, sequencing library preparation, sequencing, data analysis and data management."
What exactly was learnt about all this? Qualification needed here - I have no doubt the authors gained a great deal of insight in the process of generating this huge dataset and it would be well worth sharing.
"DNA Extraction [27] and BGISEQ-500 WGS library construction protocols can be found in protocols.io [28]."
Are these the appropriate references...?
Table 1: does it really make sense to summarie this by order? Surely the quantities of raw data are only comparable if you break that down to families/genera/species/individuals?

Methods

Are the methods appropriate to the aims of the study, are they well described, and are necessary controls included? Choose an item.

Conclusions

Are the conclusions adequately supported by the data shown? Choose an item.

Reporting Standards

Does the manuscript adhere to the journal's guidelines on minimum standards of reporting? Choose an item.

Choose an item.

Statistics

Are you able to assess all statistics in the manuscript, including the appropriateness of statistical tests used? Choose an item.

Quality of Written English

Please indicate the quality of language in the manuscript: Choose an item.

Declaration of Competing Interests

Please complete a declaration of competing interests, considering the following questions:

- Have you in the past five years received reimbursements, fees, funding, or salary from an organisation that may in any way gain or lose financially from the publication of this manuscript, either now or in the future?
- Do you hold any stocks or shares in an organisation that may in any way gain or lose financially from the publication of this manuscript, either now or in the future?
- Do you hold or are you currently applying for any patents relating to the content of the manuscript?
- Have you received reimbursements, fees, funding, or salary from an organization that holds or has applied for patents relating to the content of the manuscript?
- Do you have any other financial competing interests?
- Do you have any non-financial competing interests in relation to this paper?

If you can answer no to all of the above, write 'I declare that I have no competing interests' below. If your reply is yes to any, please give details below.

I declare that I have no competing interests

I agree to the open peer review policy of the journal. I understand that my name will be included on my report to the authors and, if the manuscript is accepted for publication, my named report including any attachments I upload will be posted on the website along with the authors' responses. I agree for my report to be made available under an Open Access Creative Commons CC-BY license (http://creativecommons.org/licenses/by/4.0/). I understand that any comments which I do not wish to be included in my named report can be included as confidential comments to the editors, which will not be published.

I agree to the open peer review policy of the journal

To further support our reviewers, we have joined with Publons, where you can gain additional credit to further highlight your hard work (see: https://publons.com/journal/530/gigascience). On publication of this paper, your review will be automatically added to Publons, you can then choose whether or not to claim your Publons credit. I understand this statement.

Yes Choose an item.
