# Supplementary material for: Molecular digitization of a botanical garden: high-depth whole-genome sequencing of 689 vascular plant species from the Ruili Botanical Garden
Source: Gigascience. 2019 Jan 25;8(4):giz007. doi: 10.1093/gigascience/giz007 (PMC6441391; doi:10.1093/gigascience/giz007)
Supplement: Reviewer_2_Report_Original_Submission -- Alex Twyford — 10/2/2018 Reviewed [file giz007_reviewer_2_report_original_submission.pdf]

Reviewer Report

Title: Molecular Digitization of a Botanical Garden: High-depth whole genome sequencing of 689 vascular plant species from the Ruili Botanical Garden

Version: Original Submission **Date:** 10/2/2018

Reviewer name: Alex Twyford

Reviewer Comments to Author:

This paper sequences and assembles the genomes of diverse plant species found at the Ruili Botanical Garden, China. The authors generate approximately 60 Gb short sequence read data from 760 samples, and perform a range of analyses suitable for low-coverage draft genomes such as repeat content characterisation and plastid assembly. These analyses show considerable variation in many genomic properties across plants. While the paper is descriptive, and is limited by using the same sequencing approach and assembly parameters for all samples, I do think it is impressive in terms of the scale of sequencing, and will serve as a good test case for large-scale sequencing of diverse sample sets.

Major comments

1. This paper is likely to be used as a test case for sequencing diverse samples across a flora. As such, I'm sure the reader would like to know the relationship between coverage and contiguity in the assemblies. Also, were mitochondrial genome assemblies attempted, and were these successful? More generally, it would be useful to explain why the same sequencing effort was allocated to all samples regardless of known genome size (and would you suggest others follow this approach in the future?).
2. As far as I'm aware, Astral is a species tree approach that should be used on many independent loci. I don't think it's suitable for the plastid as all loci are linked. An alternative phylogenetic approach should be used (such as partitioned analysis in IQ-TREE).
3. The quality of the writing and clarity of some sentences could be improved.
4. This is the first time I've seen repeat content and heterozygosity summarised across such a broad sample set, and I'd be interested in more interpretation of these results. Presumably you'd consider these to be extremely labile across plants? Given that lability, I wonder the suitability of summarising this across species within a family (as shown in the box plots)?

Minor comments

Line 52. Next generation sequencing technologies?

Line 81. Genome size and repeat content estimates are possible from genome skim data (for example, see RepeatExplorer), so this critique doesn't seem fair.

Line 85: A major challenge for diverse genome sequencing projects like this one is choosing whether to sequence samples in accordance to their genome size, or whether to use a 'one size fits all' approach with similar amounts of data for all samples. It would be valuable to justify this approach, and say the range of coverage this amount of data generates (see main comment 1, above).

Line 106: The details of DNA extraction are very brief. Was the same DNA extraction protocol used for all species? Were there any modifications?

Line 107: BGISEQ is not a very established sequencing platform relative to other platforms (such as

Illumina). A brief description of this technology would be useful.
Line 108: What type of libraries were prepared? What was the insert size?
Line 110. Repetition of 100bp PE from the previous line.
Line 166: Define which repeats are being counted.
Line 183: It's unclear to me how heterozygosity was scored. Were reads mapped back to the initial assembly then heterozygous sites scored?
Line 255: Evolution of vasculature is not really relevant to this study.
Figure legends lack detail, e.g. details of the phylogenetic reconstruction used in Figure 2.
Reference 4 seems incomplete.

Methods

Are the methods appropriate to the aims of the study, are they well described, and are necessary controls included? Choose an item.

Conclusions

Are the conclusions adequately supported by the data shown? Choose an item.

Reporting Standards

Does the manuscript adhere to the journal's guidelines on minimum standards of reporting? Choose an item.

Choose an item.

Statistics

Are you able to assess all statistics in the manuscript, including the appropriateness of statistical tests used? Choose an item.

Quality of Written English

Please indicate the quality of language in the manuscript: Choose an item.

Declaration of Competing Interests

Please complete a declaration of competing interests, considering the following questions:

- Have you in the past five years received reimbursements, fees, funding, or salary from an organisation that may in any way gain or lose financially from the publication of this manuscript, either now or in the future?
- Do you hold any stocks or shares in an organisation that may in any way gain or lose financially from the publication of this manuscript, either now or in the future?

- Do you hold or are you currently applying for any patents relating to the content of the manuscript?
- Have you received reimbursements, fees, funding, or salary from an organization that holds or has applied for patents relating to the content of the manuscript?
- Do you have any other financial competing interests?
- Do you have any non-financial competing interests in relation to this paper?

If you can answer no to all of the above, write 'I declare that I have no competing interests' below. If your reply is yes to any, please give details below.

I declare that I have no competing interests

I agree to the open peer review policy of the journal. I understand that my name will be included on my report to the authors and, if the manuscript is accepted for publication, my named report including any attachments I upload will be posted on the website along with the authors' responses. I agree for my report to be made available under an Open Access Creative Commons CC-BY license (http://creativecommons.org/licenses/by/4.0/). I understand that any comments which I do not wish to be included in my named report can be included as confidential comments to the editors, which will not be published.

I agree to the open peer review policy of the journal

To further support our reviewers, we have joined with Publons, where you can gain additional credit to further highlight your hard work (see: https://publons.com/journal/530/gigascience). On publication of this paper, your review will be automatically added to Publons, you can then choose whether or not to claim your Publons credit. I understand this statement.

Yes Choose an item.
